# Rhodium‐Catalyzed Asymmetric Arylation of Cyclobutenone Ketals

**DOI:** 10.1002/anie.202217381

**Published:** 2023-02-17

**Authors:** David Egea‐Arrebola, F. Wieland Goetzke, Stephen P. Fletcher

**Affiliations:** ^1^ Department of Chemistry Chemistry Research Laboratory Department of Chemistry University of Oxford 12 Mansfield Road Oxford OX1 3TA UK

**Keywords:** Arylboronic Acids, Asymmetric Catalysis, Complex Cyclobutanes, Cyclobutenone, Rhodium

## Abstract

Complex cyclobutanes are important motifs in both bioactive molecules and natural products, yet their enantioselective preparation has not been widely explored. In this work, we describe rhodium‐catalyzed enantioselective additions of aryl and vinyl boronic acids to cyclobutenone ketals. This transformation involves enantioselective carbometalation to give cyclobutyl‐rhodium intermediates, followed by β‐oxygen elimination to afford enantioenriched enol ethers. Overall, this addition serves as a surrogate for Rh‐catalyzed 1,4‐additions to cyclobutenone.

## Graphical abstract

We report bench‐stable cyclobutenone surrogates that undergo Rh‐catalyzed addition of aryl‐ and vinylboronic acids to yield cyclobutenyl enol ethers in high yields and excellent enantioselectivities. This transformation proceeds via enantioselective carbometalation to give cyclobutyl‐rhodium intermediates, followed by β‐oxygen elimination. The reaction is fast and proceeds under mild conditions.

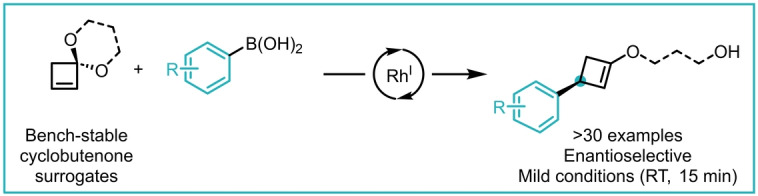

Cyclobutane rings can be found in natural products with remarkable biological activity (Figure 1, top row),[^1^, ^2^, ^3^] and the introduction of this small carbocycle is a powerful strategy in the preparation of biologically active molecules (Figure 1, bottom row).^[4]^ Many of the cyclobutane motifs reported in the study of synthetic biologically active materials do not introduce many, if any, stereogenic centers, and the lack of complexity in synthetic cyclobutane‐containing molecules is likely caused by a scarcity of methods for their stereocontrolled synthesis.

**Figure 1:**
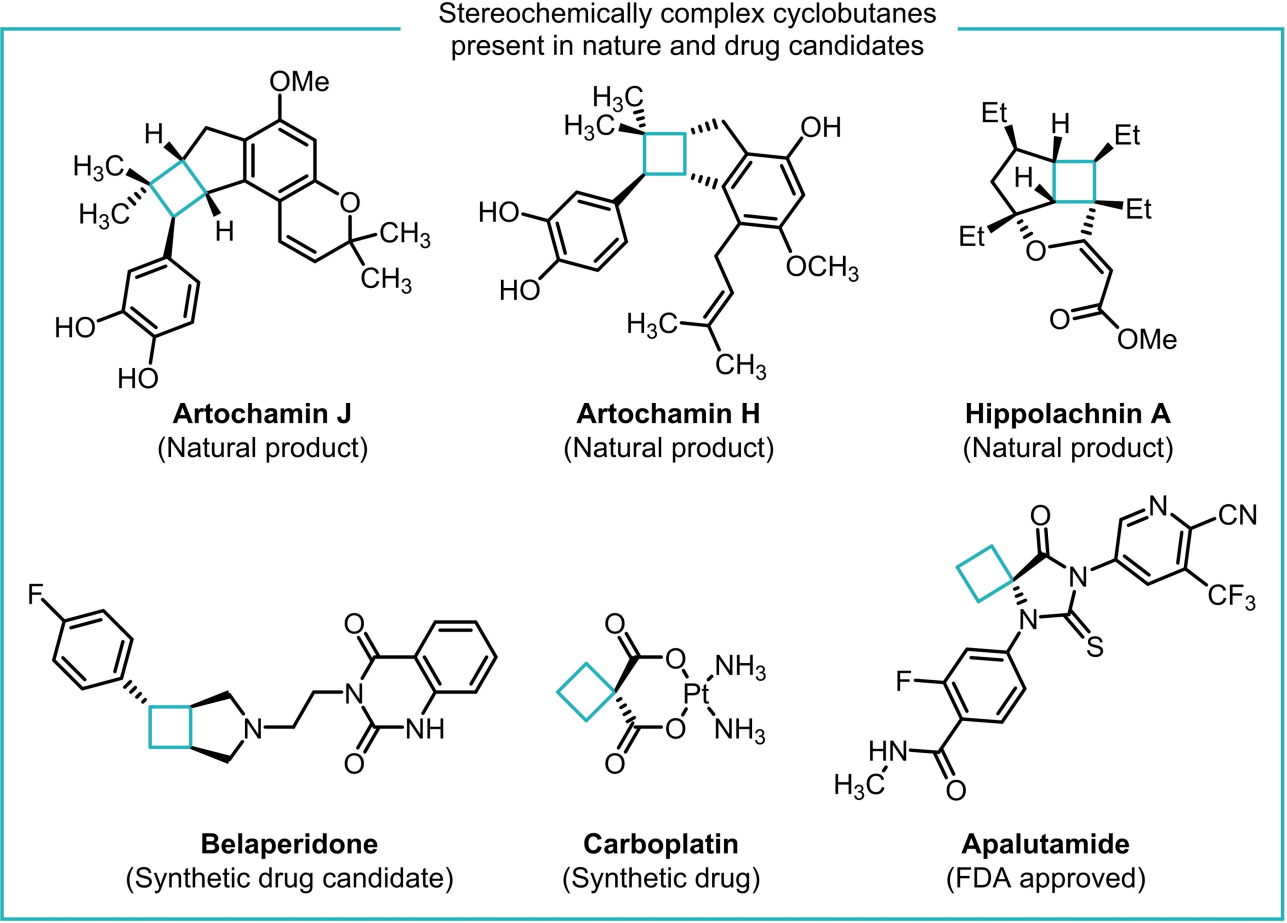
Examples of stereochemically complex cyclobutanes found both in nature and in drug candidates.

The most developed strategies for the enantioselective preparation of four‐member‐ring cores are [2+2] cycloadditions and skeletal rearrangements (i.e. ring expansion or contraction) but the enantioselective functionalization of pre‐existing cyclobutanes or cyclobutenes has recently emerged as a complementary approach.[^3^, ^5^, ^6^, ^7^]

Tortosa and co‐workers described a copper‐catalyzed desymmetrization for the enantioselective synthesis of cyclobutylboronates. This approach yields 1,2,3‐trisubstituted cyclobutanes that can readily undergo further modifications.^[8]^ The same group also reported asymmetric Pt‐catalyzed diborylation of spirocyclic cyclobutenes^[9]^ and copper‐catalyzed monoboration of spirocyclic cyclobutenes to yield achiral 3‐substituted spirocyclobutanes (Figure 2A).^[10]^

**Figure 2:**
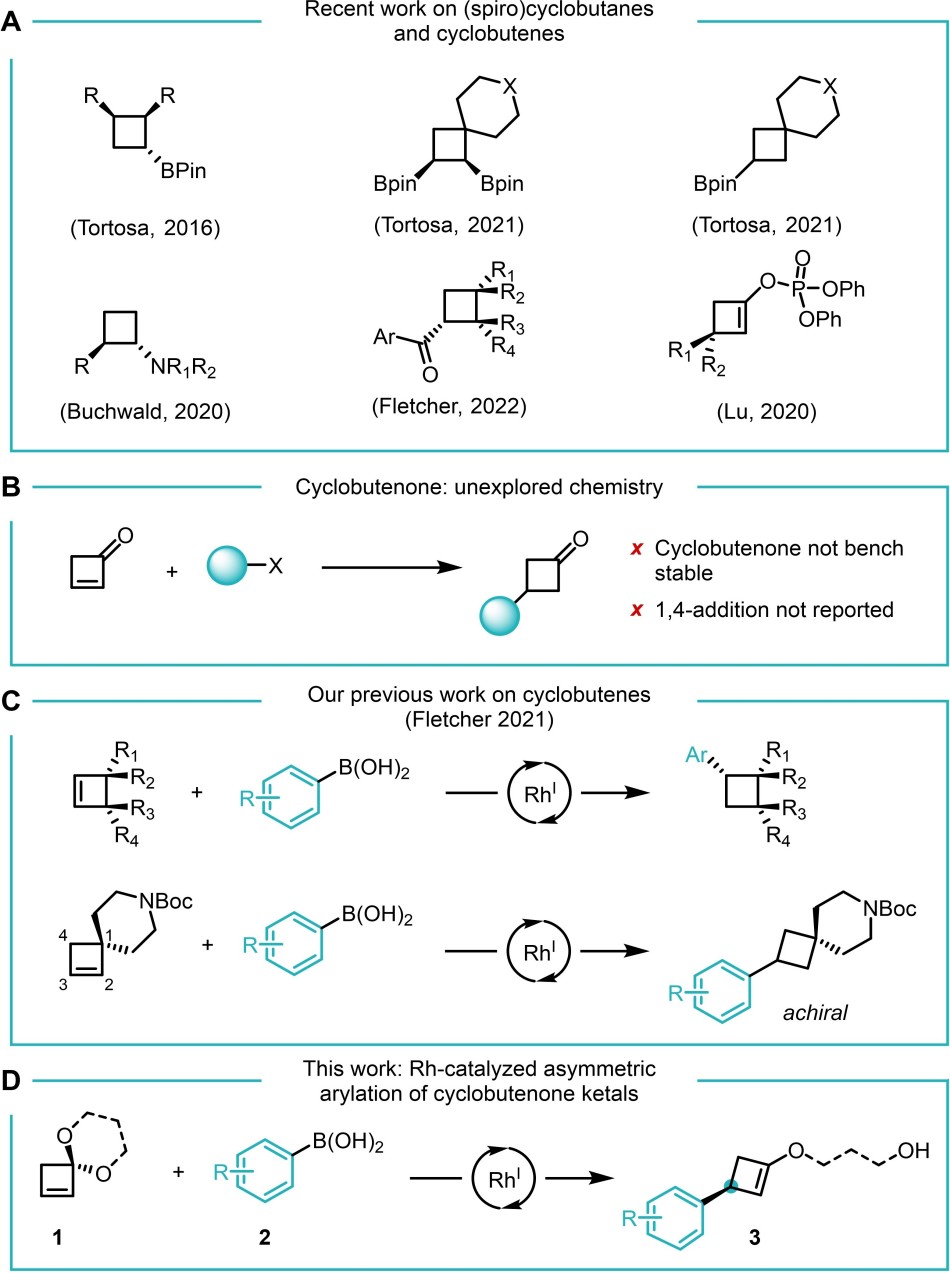
A) Recent work on (spiro)cyclobutanes and cyclobutenes by the Tortosa, Buchwald, Fletcher and Lu groups. B) Asymmetric conjugate addition to cyclobutenone has not been reported, presumably because cyclobutene has poor stability. C) Use of catalytic asymmetric arylation to synthesize complex cyclobutanes. D) This work: **1** serves as a bench‐stable surrogate for cyclobutenone that can undergo carbometalation initiated transformations to access enantioenriched enol ethers.

Buchwald and co‐workers developed a CuH‐catalyzed enantioselective hydroamination of 1‐substituted cyclobutenes to give enantioenriched *trans*‐1,2‐disubstituted aminocyclobutanes (Figure 2A),^[11]^ and Rh‐catalyzed asymmetric hydroacylation of cyclobutenes^[12]^ has also been reported, with both of these reactions believed to occur via a key asymmetric hydrometalation step.

1,4‐addition to α,β‐unsaturated ketones is one of the most well explored strategies for the enantioselective formation of C−C bonds.[^13^, ^14^, ^15^, ^16^] While a large variety of linear and cyclic substrates can undergo metal catalyzed asymmetric conjugate addition, a key exception is cyclobutenone. Cyclobutenone itself is intrinsically unstable, as it rapidly polymerizes even at cryogenic temperatures.^[17]^ Furthermore, the ketone that would arise from the simple 1,4‐addition‐protonation sequence that is normally first developed is achiral (Figure 2B), although enolates arising from addition would be chiral. The Lu group recently reported copper‐catalyzed asymmetric additions to 3‐substituted cyclobutenones which allowed a variety of enantioenriched cyclobutenes to be obtained by trapping chiral intermediates with a suitable electrophile (Figure 2A).^[18]^

Our group reported a series of asymmetric arylations of cyclobutenes that proceed via a common asymmetric carbometalation step.^[19]^ After initial carborhodation, different reaction pathways—depending on the substituents on the ring and reaction conditions—can give rise to a variety of different products. For some substrates, we observed β‐hydrogen elimination pathways followed by hydrometalation (i.e. Rh chain walking), a process that was exploited to yield remote elimination reactions. As part of this study, we also observed that cyclobutenes featuring spirocycles underwent regioselective reductive‐Heck‐reactions at the 3‐position to give achiral cyclobutanes (Figure 2C).

Rhodium's ability to eliminate β‐heteroatoms made us consider ways to break the symmetry of spirocyclic cyclobutenes. In particular, we wondered if cyclobutenes containing a spirocyclic ketal would undergo highly regio‐ and enantioselective carborhodation, followed by β‐oxygen elimination, and formally mimic the trapping of enol intermediates obtained after 1,4‐addition to cyclobutenone. Here, we present a Rh‐catalyzed desymmetrization of cyclobutenone ketals, providing an effective surrogate for an asymmetric conjugate addition to cyclobutenone (Figure 2D).

Initially, we developed a multi‐gram synthesis of cyclobutenone ketal **1 a** (Scheme 1) starting from commercially available cyclobutanone. α‐Bromination, ketalization and then elimination gave **1 a**.

**Scheme 1:**
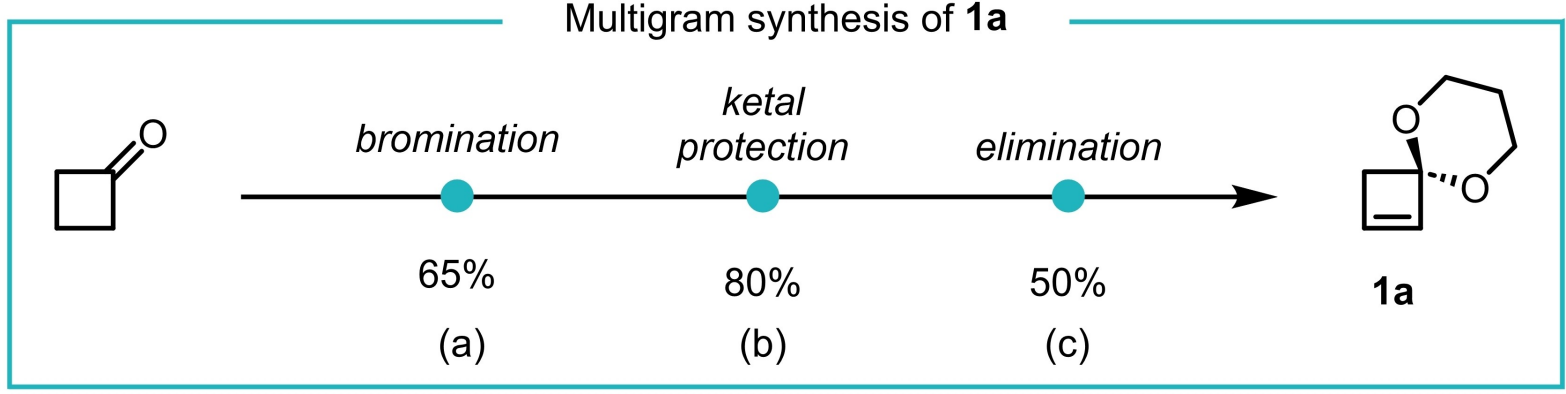
Multi‐gram preparation of cyclobutenone ketal **1 a**. Conditions: a) NBS, CH_2_Cl_2_, *p*TsOH_(cat)_, 2 days, reflux. b) 1,3‐propanediol, *p*TsOH_(cat)_, C_6_H_6_, 4 h, Dean–Stark. c) ^t^BuOK, DMSO, 1 h, r.t.

With cyclobutenone ketal **1 a** in hand, we started exploring Rh‐catalyzed aryl additions. After screening of a diverse set of reaction parameters, we found that compound **3 aa** could be obtained in 85 % yield with 95 % enantiomeric excess under mild conditions at room temperature in 15 minutes (Table 1).

**Table 1:** Deviation from standard conditions.

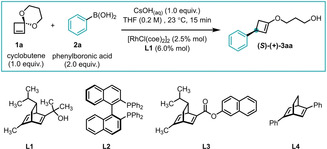			
Entry	Deviation from standard conditions	Yield [%]^[a]^	ee [%]^[b]^
1	None	85	95
2	No Rh	0	N/A
3	No ligand^[c]^	68	0
4	**L2** instead of **L1** ^[c,d]^	63	29
5	Cyclooctadiene instead of **L1** ^[e]^	79	0
6	**L3** instead of **L1**	74	−24
7	**L4** instead of **L1**	34	82
8	Toluene instead of THF	77	89
9	No base^[f]^	2	87
10	Cs_2_CO_3_ instead of CsOH_(aq)_	0	N/A

All experiments were performed on 0.2 mmol scale. [a] Isolated yield. [b] Determined by SFC using chiral non‐racemic stationary phase. [c] Reaction stirred for 16 h. [d] Reaction stirred at 60 °C. [e] Using [Rh(cod)(OH)]_2_ as pre‐catalyst. [f] 35 μL water added.

We found that enol ether **3 aa** is stable at temperatures of less than 80 °C and to base (e.g., NEt_3_, NaH, imidazole). The compound is sensitive to acid, and so traces of acid in NMR solvents or SiO_2_ need to be neutralized as **3 aa** readily tautomerizes to the corresponding achiral spirocyclobutane under acid conditions.

Diene ligand **L1** allows addition of **2 a** to **1 a** to give **3 aa** in 85 % yield and 95 % enantiomeric excess (Table 1, entry 1). The reaction does not proceed without rhodium (Table 1, entry 2) and gives racemic product without chiral ligand (Table 1, entry 3). During optimization, we found using C2‐symmetrical chiral bisphosphines such as **L2**, which had been previously successful in enantioselective additions of arylboronic acids to cyclobutenes,^[19]^ were not able to induce high levels of ee in the formation of **3 aa** in (Table 1, entry 4 and Supporting Information).

Other diene ligands examined gave diminished enantioselectivity and/or yields (Table 1 entries 5–7) and toluene as solvent gave lower enantioselectivity (Table 1, entry 8). The presence of base and water seems to be required for catalyst turnover (Table 1, entries 9 and 10).

The corresponding cyclopentene and cyclohexene ketal analogues of **1 a** showed no desired reactivity under related reaction conditions even at elevated temperatures.

We explored various arylboronic acids as coupling partners for this transformation (Scheme 2). Several different *meta*‐ and *para‐*substituted arylboronic acids gave the desired enol ethers in good to excellent enantioselectivities and yields. The reaction with **1 a** is compatible with a wide range of functional groups, such as arylboronic acids bearing halogens (**3 ac**, **3 ai**, **3 aj**–**l**, **3 am**, **3 aq**) (thio)‐alkoxy groups (**3 ab**, **3 af**, **3 as**), acetyl and esters (**3 ag**, **3 at**), carbamate‐protected amines (**3 ar**) and silanes (**3 ap**).

**Scheme 2:**
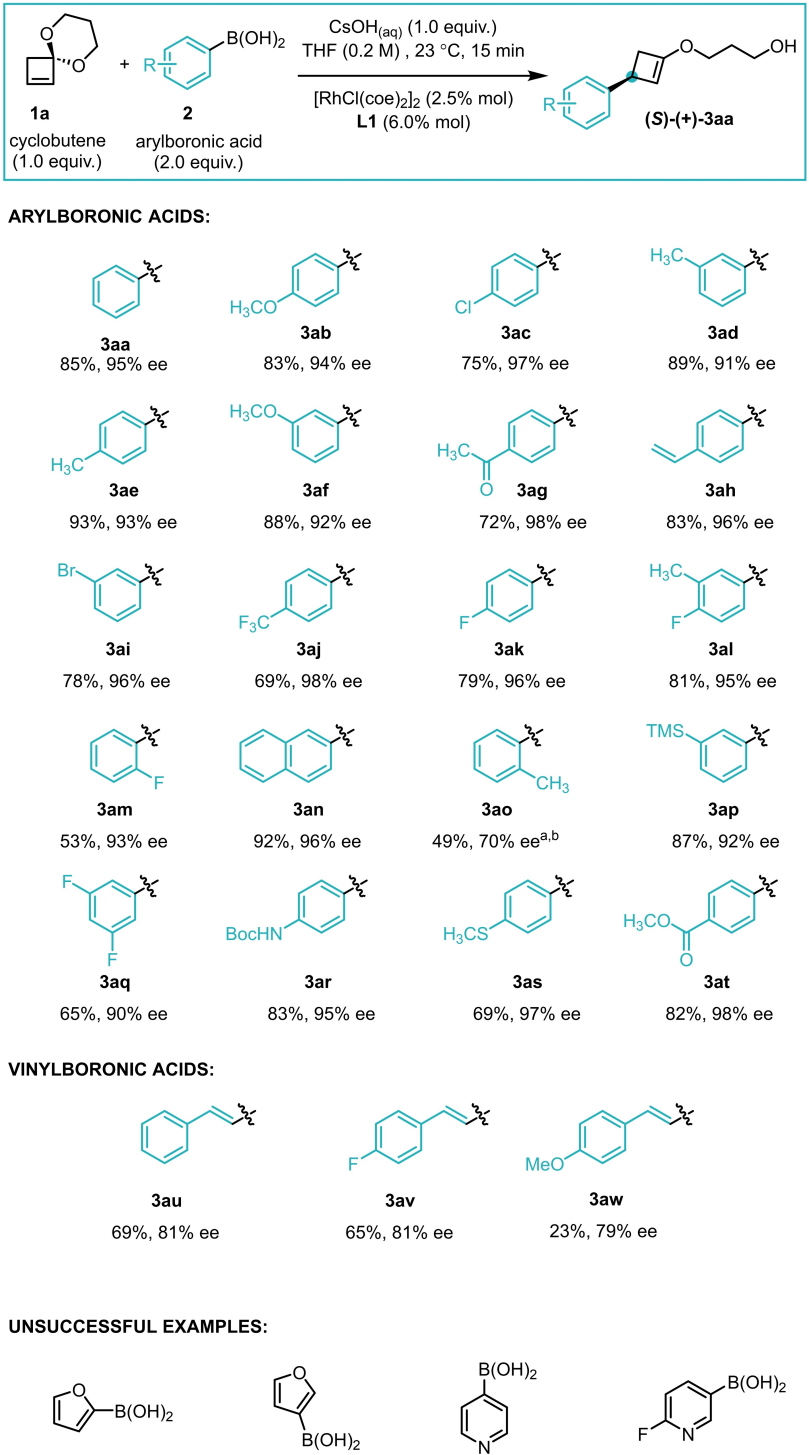
Scope of the reaction between **1 a** and a range of boronic acids. All experiments were performed on a 0.4 mmol scale for **1 a**. Reported yields are isolated yields. Enantiomeric ratios were determined by SFC using a chiral non‐racemic stationary phase. a) Reaction performed at 60 °C. b) **L4** used instead of **L1**.

O*rtho*‐substituted boronic acids are generally challenging coupling partner in transition metal catalyzed 1,4‐additions and related reactions and suffer often from low yields and enantioselectivities.^[20]^ While 2‐fluorophenylboronic acid gave good results (**3 am**, 53 %, 93 % ee), sterically more hindered 2‐methylphenylboronic acid addition gave only 58 % ee. In the latter case, using ligand **L4** increased the enantioselectivity to 70 % ee (**3 ao**), suggesting that it may be possible to fine‐tune reaction conditions for this particular boronic acid. A small set of *trans*‐styreneboronic acids was examined. The corresponding 1,4‐dienes were obtained in good yields, albeit with moderate enantioselectivities. Heteroarylboronic acids are not compatible with the current methodology.

Additionally, three other spirocyclic cyclobutenes with different substituents in the ketal, as well as different ring sizes, were tested in the addition/elimination sequence with different arylboronic acids (Scheme 3). Excellent results were also obtained with these ketals **1 b**–**d**. Cyclobutenes **1 b** and **1 c** yielded the corresponding products with high enantioselectivities. On the other hand, when using the 5‐membered acetal cyclobutene **1 d**, lower ee's were observed.

**Scheme 3:**
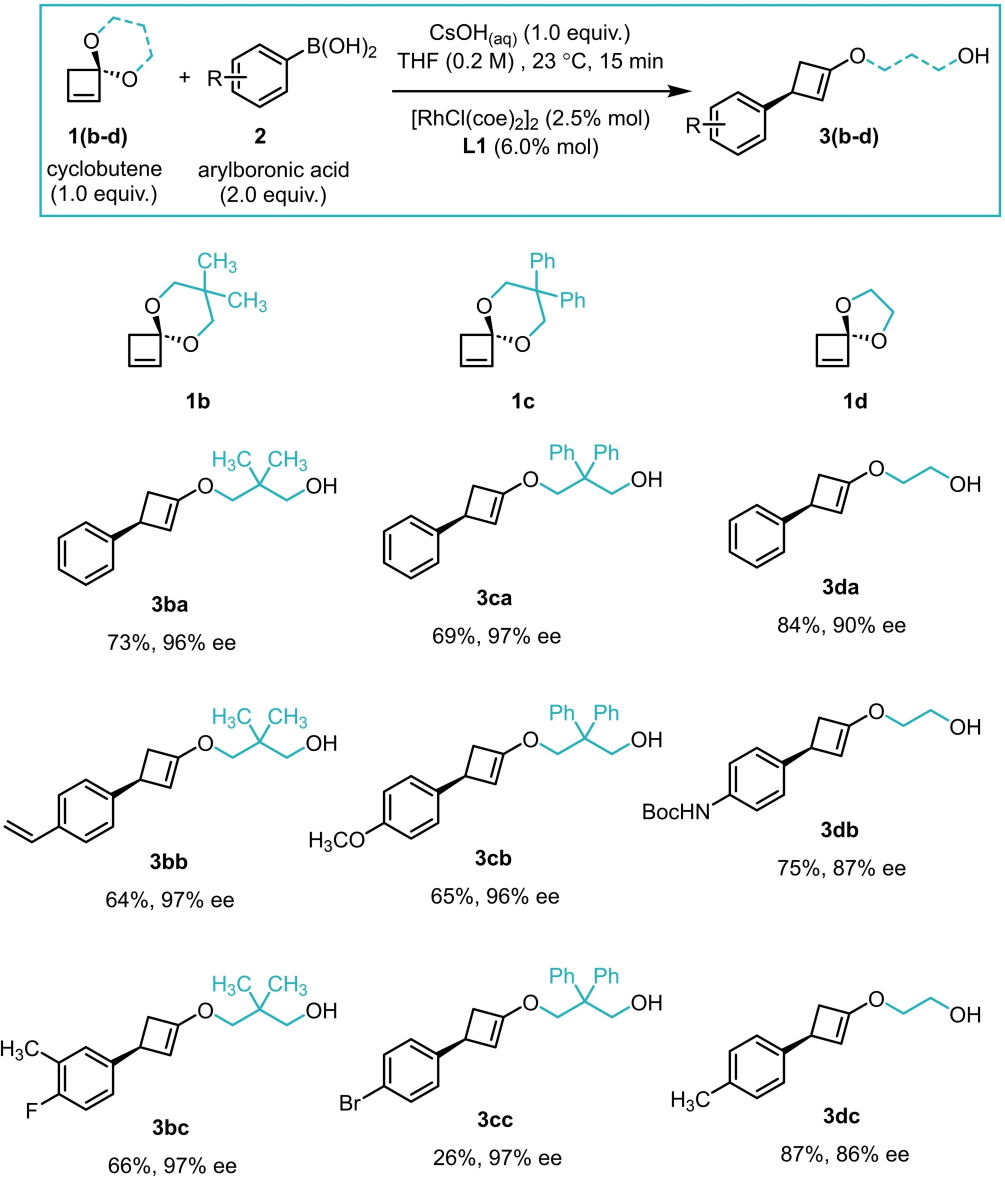
Cyclobutene scope. All experiments were performed on a 0.4 mmol scale for **1(b**–**d)**. Reported yields are isolated yields. Enantiomeric ratios were determined by SFC using a chiral non‐racemic stationary phase.

We propose the following mechanism based on the products formed, analysis of a by‐product and our previous studies (Scheme 4). The rhodium(I)‐hydroxide complex **I** undergoes transmetalation with boronic acid **2** to intermediate **II**.[^21^, ^22^] Then, **II** and alkene **1 a** react via carbometalation, generating intermediate **III**. The regioselectivity of the carbometalation step is likely not controlled by any directing effect of the ketal but fully under steric control.^[19]^ **III** then undergoes β‐hydroxy elimination to open the ketal and gives **IV**. Catalyst **I** is finally regenerated by hydrolysis of **IV**, a process that at the same time yields product **3 a**. Intermediate **III** can also undergo a side reaction to give what is likely by‐product **4 a**. **VI** would be obtained after oxidative addition into the *ortho*‐H of the aryl‐ring via a 1,4‐Rh shift.^[23]^ For some electron‐withdrawing aryl substituents, the rate of this off‐cycle oxidative addition is considerably increased, giving small amounts of side‐product **4 a** (e.g. for **3 ak**, **3 ar**).

**Scheme 4:**
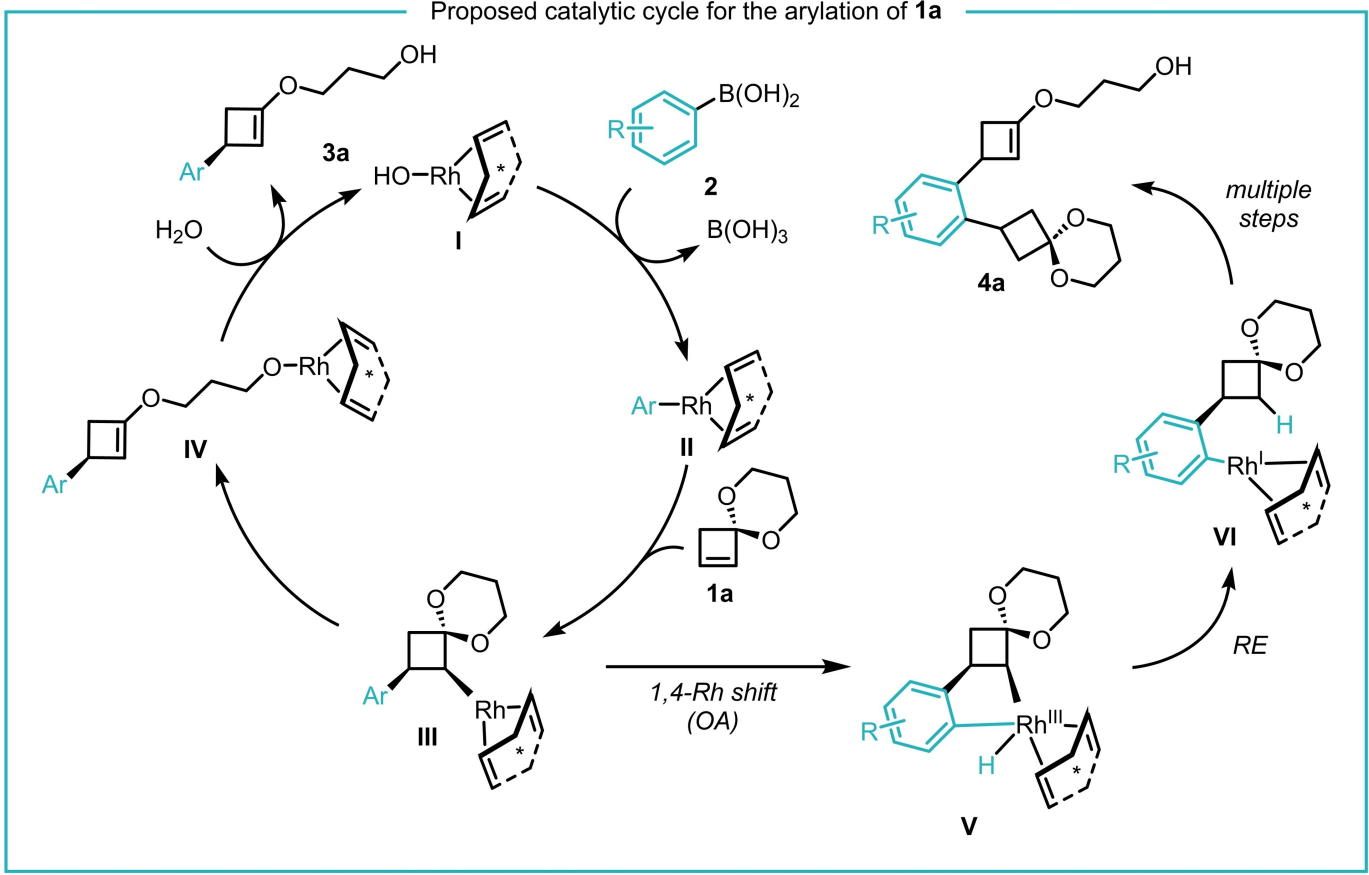
Proposed catalytic cycle for the arylation of **1 a** and the possible formation of byproduct **4 a**.

To show the synthetic utility of this method, we performed a 10 mmol scale reaction with decreased catalyst loading (2.5 mol % Rh, 3.0 mol % ligand) between **1 a** and **2 a** which afforded 1.43 g of **3 aa** (73 %, 93 % ee, Scheme 5).

**Scheme 5:**
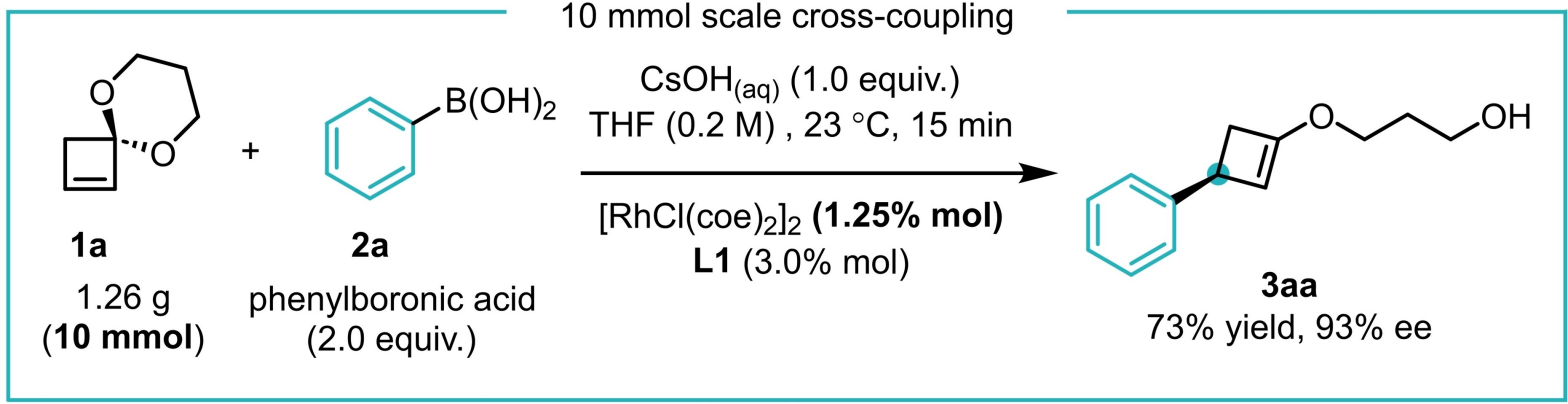
10 mmol scale addition between **1 a** and **2 a** with reduced catalyst loading.

Alkyl enol ether **3 aa** can be further elaborated into a range of different products (Scheme 6). Hydrolysis of racemic **3 aa** yields 3‐phenylcyclobutanone (**5**), showing cyclobutenone ketal **1 a** is a stable surrogate for 1,4‐addition to cyclobutenone with arylboronic acids. Heating cyclobutene **3 aa** triggers electrocyclic ring opening to the electron‐rich diene **6**.

**Scheme 6:**
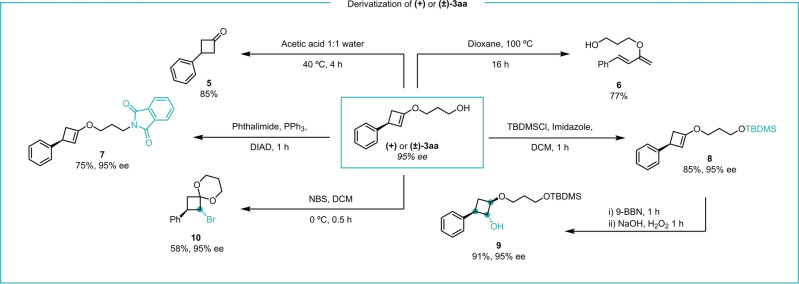
Synthetic utility of enantioenriched **(+)** or **(±)‐3 aa**.

The terminal hydroxy group can be used a handle for subsequent functionalizations. E.g., a Mitsunobu‐type substitution with phthalimide gave **7**. The activated double bond in the carbocycle can be used to produce a variety of products, although some care must be taken because of the sensitively of cyclobutenes **3**. The alkyl enol ether can be α‐functionalized with *N*‐bromosuccinimide to give bromoketal **10**. Borylation‐oxidation of *tert*‐butyldimethylsilyl (TBDMS) silyl‐protected **8** leads to **9**, a cyclobutane ring with 3 contiguous stereogenic centers. Importantly, these transformations install functional groups that can be further elaborated. The Mosher esters derived from **9** were used to assign the absolute stereochemistry of the products.^[24]^

In summary, we have developed an efficient and selective rhodium catalyzed arylation of cyclobutenone ketal **1 a**. The reaction uses a combination of asymmetric carbometalation and β‐oxy‐elimination to give chiral enol ethers from achiral ketals. The reaction is a surrogate for the asymmetric addition to cyclobutanone. The reaction occurs rapidly at room temperature using a chiral diene ligand and the products can be further modified to access more complex cyclobutanes.

## Conflict of interest

The authors declare no conflict of interest.

## Supporting information

As a service to our authors and readers, this journal provides supporting information supplied by the authors. Such materials are peer reviewed and may be re‐organized for online delivery, but are not copy‐edited or typeset. Technical support issues arising from supporting information (other than missing files) should be addressed to the authors.

Supporting Information

## Data Availability

The data that support the findings of this study are available in the Supporting Information of this article.
