# Supplementary material for: Rhodium‐Catalyzed Asymmetric Arylation of Cyclobutenone Ketals
Source: Angew Chem Int Ed Engl. 2023 Feb 17;62(13):e202217381. doi: 10.1002/anie.202217381 (PMC10946970; doi:10.1002/anie.202217381)
Supplement: Supplementary file 1 — Supporting Information [file ANIE-62-0-s001.pdf]

Angewandte Chemie
Eine Zeitschrift der Gesellschaft Deutscher Chemiker

Supporting Information

Rhodium-Catalyzed Asymmetric Arylation of Cyclobutenone Ketals

*D. Egea-Arrebola, F. W. Goetzke, S. P. Fletcher**

List of Contents

1 General Methods **2**

2 Detailed ligand screening **4**

3 Experimental procedures **6**

3.1 General method for the preparation of racemates 19

3.2 General methods for the preparation of compounds **3aa-w**, **3ba-c**, **3ca-c** and **3da-c** 19

4 Further transformations **92**

5 Mosher ester analysis for the determination of absolute configuration **116**

5.1 Determination of relative configuration in compound **9** 116

5.2 Determination of absolute configuration in compound **9** 120

6 SFC traces **122**

1 General Methods

All reactions were carried out in anhydrous solvents with continuous magnetic stirring under an inert argon atmosphere, unless otherwise specified. Heating was performed using DrySyn heating blocks.

Nuclear magnetic resonance (NMR) spectroscopy measurements were carried out at room temperature. ^{1}H NMR, ^{13}C NMR, ^{19}F NMR, COSY, HSQC, HMBC and NOESY experiments were carried out using Bruker AVIII HD 400 (400/101 MHz) or AVIII HD 400 (500/125 MHz) spectrometers. Chemical shifts (δ) are reported in ppm relative to the residual solvent peak with corresponding coupling constants (*J*) in Hertz (Hz) and multiplicities (s: singlet, d: doublet, t: triplet, q: quartet, p: quintuplet, m: multiplet and combinations of these and app.: apparent multiplicities). Assignment follows HSQC, HMBC or/and COSY spectra, chemical shift and coupling constant analysis.

Optical rotations ($[\alpha]_{D}^{25}$) were recorded using a Perkin Elmer-241 Polarimeter. Concentrations (c) are reported in g/100 mL.

Infrared (IR, neat): spectroscopy was carried out on a Bruker Tensor 27 FT–IR spectrometer with an internal calibration range of 4000–600 cm^{-1}. The samples are reported as absorption maxima in cm^{-1} with corresponding relative intensities described as br (broad), s (strong), m (medium) and w (weak).

Chiral SFC (supercritical fluid chromatography) separations were conducted on a Waters Acquity UPC2 system using Waters Empower software. Chiralpak® columns (150x3 mm, particle size 3 µm) were used as specified in the text. Solvents used were of HPLC grade (Fisher Scientific, Sigma Aldrich or Rathburn).

High Resolution Mass Spectra were carried out by internal service at the University of Oxford. Electron spray ionisation (ESI) was recorded on a Fisons Platform II.

Commercially available reagents and ligands were purchased from Sigma Aldrich, Alfa Aesar, Acros Organics, Fluorochem and Strem Chemicals and unless otherwise stated were used without further purification. $[Rh(cod)(OH)]_2$ and $[RhCl(coe)_2]_2$ were bought from Sigma Aldrich. Dry solvents were collected fresh from an mBraun SPS–800 solvent purification system after having passed

through anhydrous alumina columns, with the exception of anhydrous THF, which was supplied by ACROS. Deuterated solvents were purchased from Sigma Aldrich.

2 Detailed ligand screening

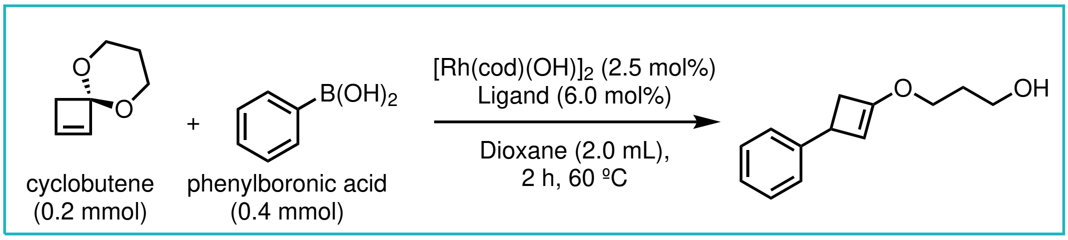

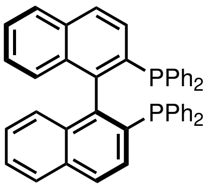

BINAP
33% ee

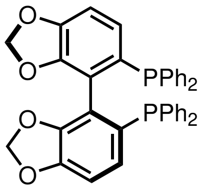

SEGPHOS
60% ee

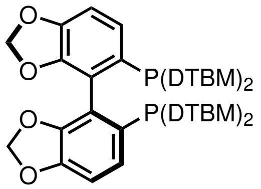

DTBM-SEGPHOS
0% ee

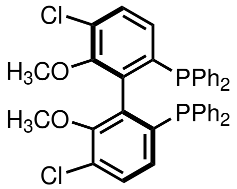

Cl-MeO-BIPHEP
60% ee

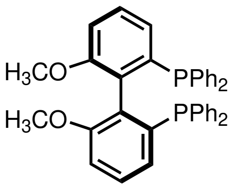

MeO-BIPHEP
54% ee

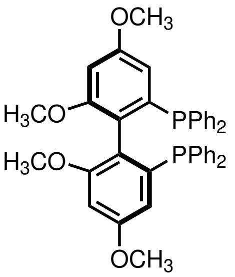

Ph-Garphos
15% ee

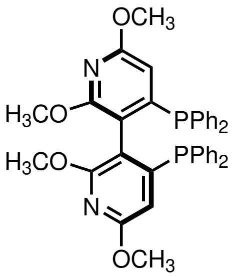

P-Phos
58% ee

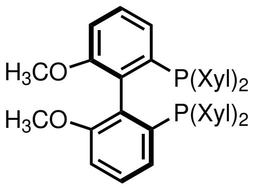

Xyl-MeO-BIPHEP
43% ee

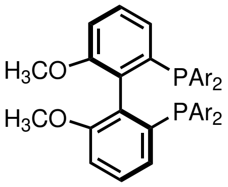

3,4,5-MeO-BIPHEP
22% ee

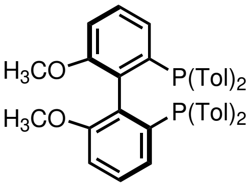

Tol-MeO-BIPHEP
37% ee

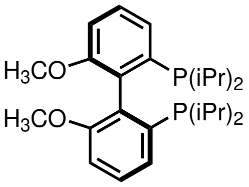

iPr-MeO-BIPHEP
0% ee

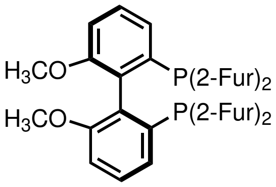

Furanyl-MeO-BIPHEP
0% ee

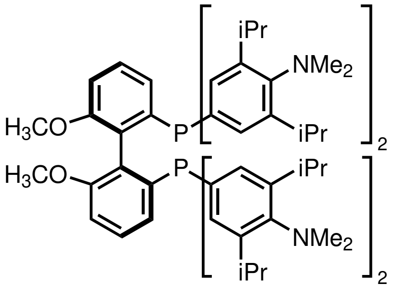

di-iProp-4-dimethylaminophenyl-MeO-BIPHEP
0% ee

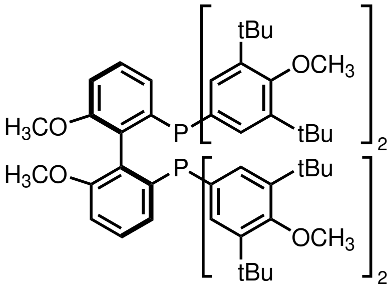

3,5-tBu-4-MeO-MeOBIPHEP
6% ee

Figure 1: Detailed ligand screening (1/2). ee determined by SFC analysis of the crude mixture.

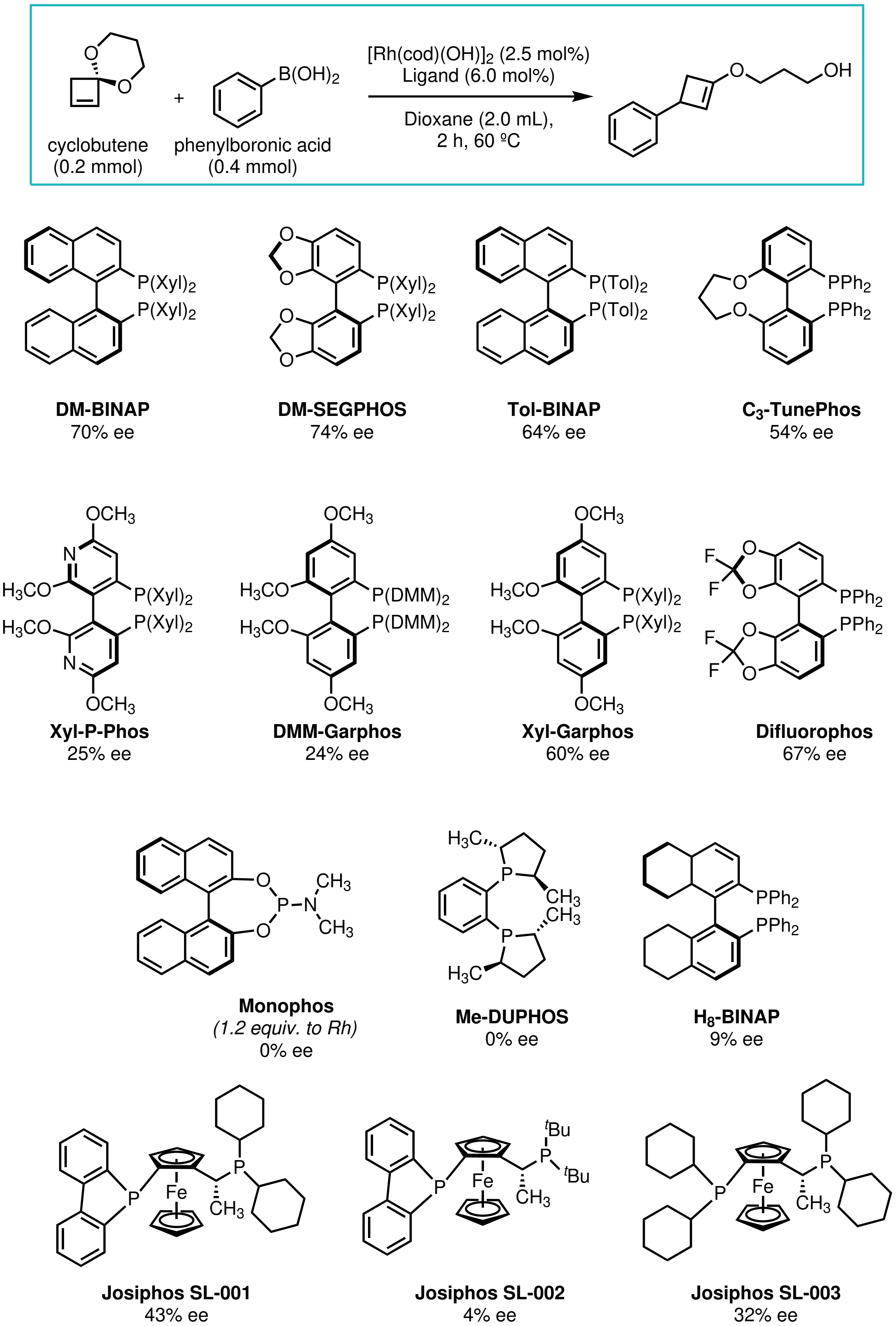

Figure 2: Detailed ligand screening (2/2). ee determined by SFC analysis of the crude mixture.

3 Experimental procedures

Synthesis of 2-bromocyclobutanone, S1

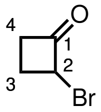

A solution of cyclobutanone (48.0 g, 686 mmol, 1.0 equiv.) in CH_2Cl_2 (96 mL) was added to a suspension of *N*-bromosuccinimide (146.4 g, 822 mmol, 1.2 equiv.) and *p*-TsOH · H_2O (12.8 g, 67.4 mmol, 0.1 equiv.) in CH_2Cl_2 (400 mL) at 0 °C under a nitrogen atmosphere. The reaction mixture was stirred under reflux.
After 2 days, the mixture was allowed to reach room temperature and it was filtered through a cotton plug. The filtrate was washed with 50 mL of a saturated $NaHCO_3$ solution followed by 50 mL of brine. The organic layer was dried over Na_2SO_4, filtered and concentrated under reduced pressure. The resulting yellow oil was distilled under reduced pressure (12 mbar) to yield a colorless oil identified as **S1** (66.4 g, 446 mmol, 65% yield; bp (12 mbar) = 62-65 °C). The liquid is stored at -20 °C, under an argon atmosphere and protected from light.

^{1}**H NMR** ($CDCl_3$, 400 MHz): δ (ppm) 4.98 – 4.92 (m, 1H, H-2), 3.23 – 3.00 (m, 2H, H-4), 2.80 – 2.56 (m, 1H, H-3), 2.29 – 2.10 (m, 1H, H-3).

^{13}**C NMR** ($CDCl_3$, 101 MHz): δ (ppm) 200.4 (C(1)), 49.4 (C(2)), 44.9 (C(4)), 22.9 (C(3)).

IR (neat): 1792 (s), 1393 (w), 1212 (w), 1065 (m), 965 (w), 894 (w), 864 (w), 688 (w) cm^{-1}.

HRMS (ESI/APCI): Not found.
Note: We were not able to detect the molecular ion or a characteristic fragment for this molecule.

Compound **S1** was isolated along with traces of 2,2-dibromocyclobutanone:

^{1}**H NMR** ($CDCl_3$, 400 MHz): δ (ppm) 3.4 (dd, J = 9.7, 8.4 Hz, 2H), 3.1 (dd, J = 9.6, 8.4 Hz, 2H).

^{13}**C NMR** ($CDCl_3$, 101 MHz): δ (ppm) 192.9, 59.6, 42.2, 38.3.

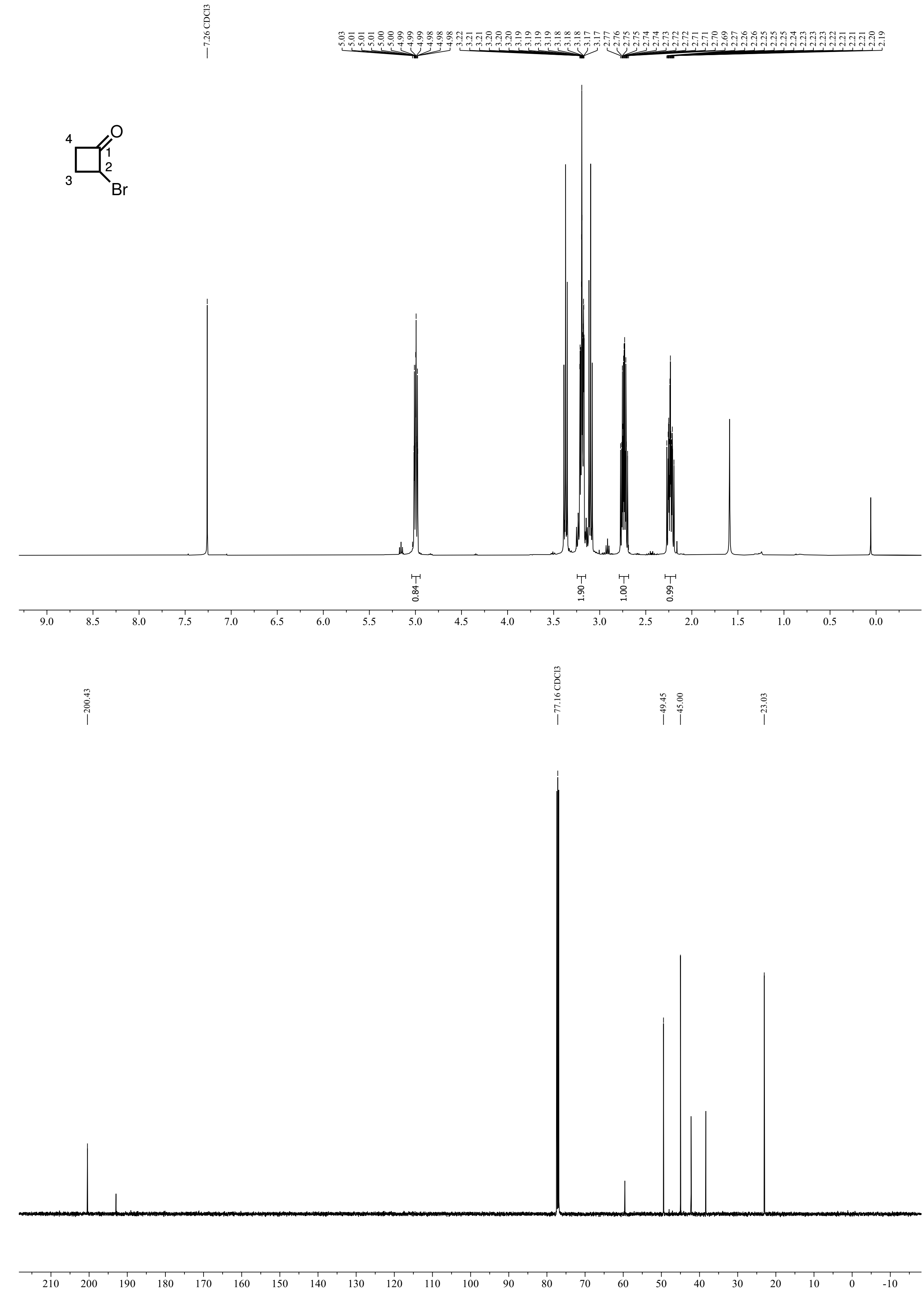

Figure 3: ^{1}H NMR (500 MHz, $CDCl_3$, top) and ^{13}C NMR (126 MHz, $CDCl_3$, bottom) for **S1**. Contains minor impurities of 2,2-dibromocyclobutanone.

Synthesis of S2

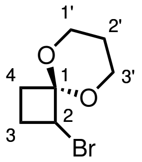

A solution of 1,3-propanediol (5.11 g, 67.0 mmol, 2 equiv.), *p*-TsOH · H_2O (646 mg, 3.4 mmol, 0.1 equiv.) and 2-bromocyclobutanone **S1** (5.0 g, 33.5 mmol) in 150 mL of benzene was stirred under reflux overnight, with a Dean-Stark trap attached to the reaction flask.
The reaction was allowed to cool down to room temperature and the mixture was washed with 50 mL of a saturated $NaHCO_3$ solution, followed by 50 mL of brine. The organic layer was dried over anhydrous Na_2SO_4, filtered and concentrated under reduced pressure. The resulting oil was distilled under reduced pressure (12 mbar) to yield a colorless oil identified as **S2** (5.55 g, 26.8 mmol, 80% yield; bp (12 mbar) = 75-80 °C) containing minor impurities.

^{1}**H NMR** ($CDCl_3$, 400 MHz): δ (ppm) 4.36 (q, *J* = 7.1 Hz, 1H, C(2)-H), 4.12 (dt, *J* = 9.4, 5.2 Hz, 1H, C(1' or 3')-H), 3.99 – 3.72 (m, 3H, C(1' or 3')-H), 2.56 (dq, *J* = 13.3, 7.3, 6.4 Hz, 1H, C(4)-H), 2.39 (dtd, *J* = 10.9, 8.2, 5.0 Hz, 1H, C(4)-H), 2.13 – 1.87 (m, 3H, C(2')-H, C(3)-H), 1.56 (dt, *J* = 12.7, 4.0 Hz, 1H, C(3)-H).

^{13}**C NMR** ($CDCl_3$, 101 MHz): δ (ppm) 100.5 (C(1)), 61.7 (C(1' or 3')), 61.6 (C(1' or 3')), 48.0 (C(2)), 30.8 (C(4)), 25.5 (C(4 or 2')), 25.4 (C(4 or 2')).

IR (neat): 2960 (w), 2866 (w), 1275 (s), 1252 (m), 1155 (s), 1066 (s), 874 (w) cm^{-1}.

HRMS (ESI/APCI): Not found.
Note: We were not able to detect the molecular ion or a characteristic fragment for this molecule.

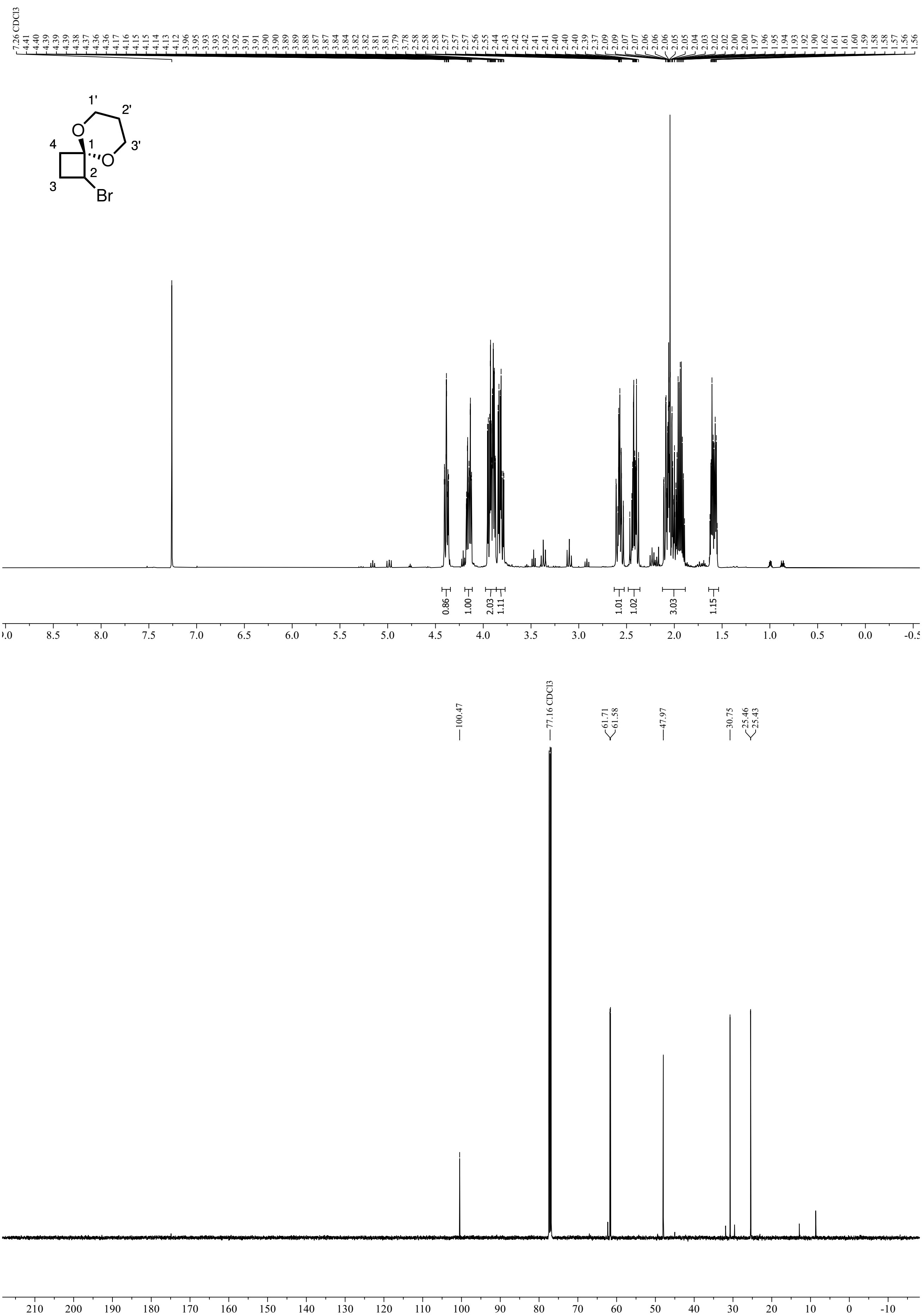

Figure 4: ^1H NMR (400 MHz, $CDCl_3$, top) and ^{13}C NMR (101 MHz, $CDCl_3$, bottom) for **S2** containing minor impurities.

Synthesis of cyclobutenone ketal, 1a

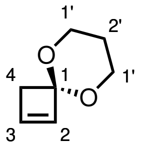

A solution of **S2** (31.73g, 153 mmol, 1.0 equiv.) in DMSO (32 mL) was added dropwise to a solution of potassium *tert*-butoxide (25.8 g, 230 mmol, 1.5 equiv.) in DMSO (60 mL) at 0 °C under an argon atmosphere. After the addition, the ice-bath was removed and the reaction mixture was stirred at room temperature for 1 h.
Then, a saturated solution of $NaHCO_3$ (30 mL) was added and the aqueous layer was extracted 10 times with cold Et_2O (10x20 mL). The combined organic layers were then washed 10 times with ice-cold brine (10x20 mL) and dried over Na_2SO_4, concentrated under reduced pressure (25 °C, 300 mbar) and purified by distillation under reduced pressure (13 mbar) to yield a colorless oil identified as **1a** (9.67 g, 77 mmol, 50% yield; bp (13 mbar) = 58 °C.).

^{1}**H NMR** ($CDCl_3$, 400 MHz): δ 6.50 (dt, *J* = 3.2, 1.0 Hz, 1H, C(3)-H), 6.33 (d, *J* = 3.1 Hz, 1H, C(2)-H), 4.05 – 3.79 (m, 4H, C(1')-H), 2.68 (d, *J* = 1.1 Hz, 2H, C(H)-4), 1.93 – 1.76 (m, 1H, C(2')-H), 1.72 – 1.59 (m, 1H, H-5 C(2')-H).

^{13}**C NMR** ($CDCl_3$, 101 MHz): δ (ppm) 140.6 (C(3)), 136.1 (C(2)), 102.5 (C(1)), 63.8 (C(2')), 42.7 (C(4)), 25.6 (C(3')).

IR (neat): 3658 (w), 2980 (s), 2887 (w), 1462 (w), 1382 (m), 1324 (m), 1290 (m), 1246 (m), 1229 (m), 1156 (m), 1136 (m), 1079 (s), 1043 (m), 957 (m), 869 (m), 718 (w) cm^{-1}.

HRMS (ESI): m/z calculated for $C_7H_{11}O_2^+$ $[M+H]^+$ = 127.0755; found = 127.0754.

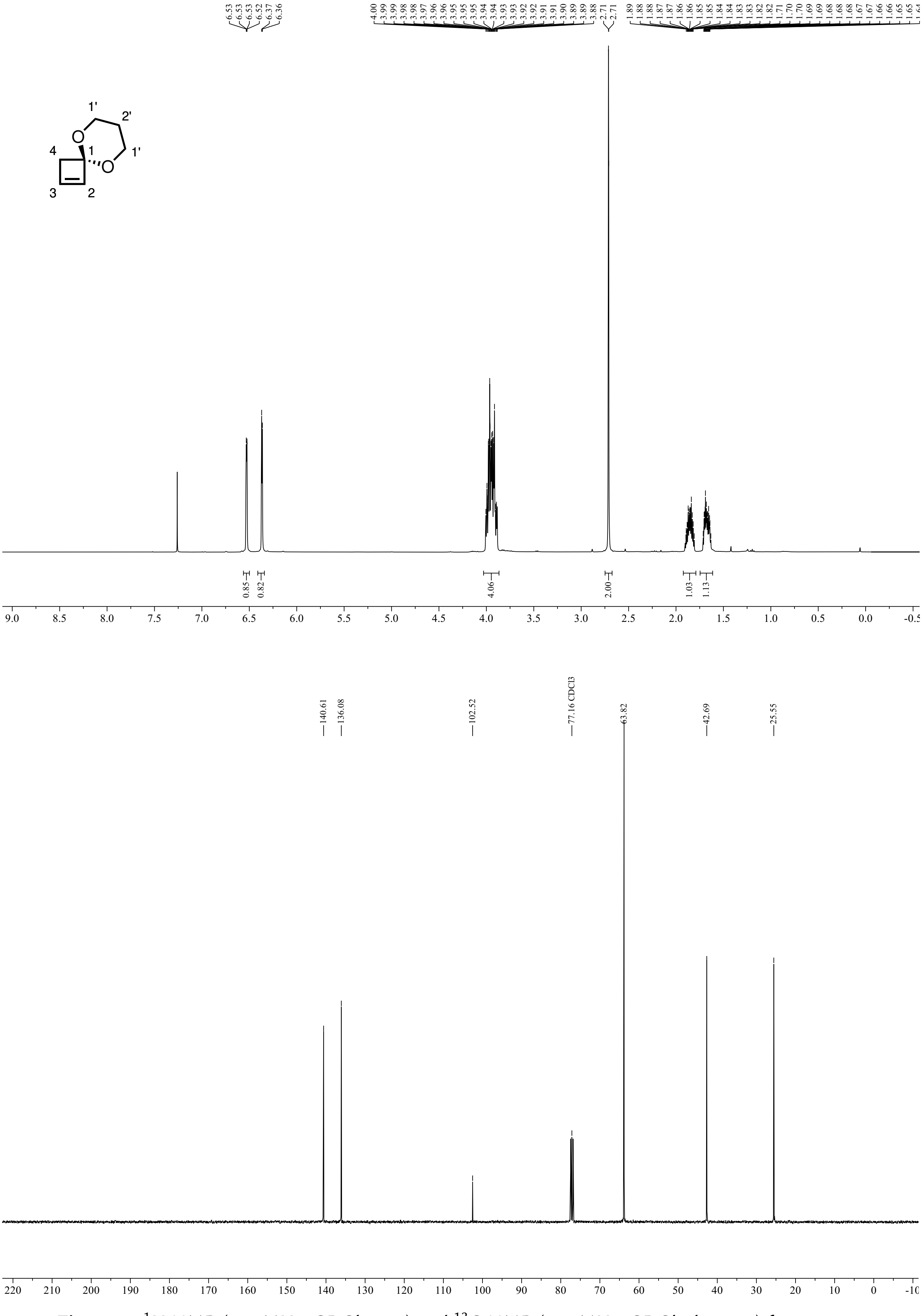

Figure 5: ^{1}H NMR (400 MHz, $CDCl_3$, top) and ^{13}C NMR (101 MHz, $CDCl_3$, bottom) for **1a**.

Synthesis of 1b

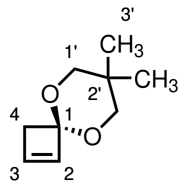

A solution of 2,2-dimethyl-1,3-propanediol (6.98 g, 67.0 mmol, 2 equiv.), *p*-TsOH · H_2O (646 mg, 3.4 mmol, 0.1 equiv.) and 2 bromocyclobutanone **S1** (5.0 g, 33.5 mmol) in 150 mL of benzene was stirred under reflux for 4 h, with a Dean-Stark trap attached to the reaction flask.
The reaction was then allowed to cool down to room temperature and the mixture was washed with 50 mL of a saturated $NaHCO_3$ solution, followed by 50 mL of brine. The organic layer was dried over anhydrous Na_2SO_4, filtered and concentrated under reduced pressure. The mixture was used without further purification in the next step.

A solution of the the product from the previous step (*vide supra*) (5.0 g, ≤21.3 mmol, 1 equiv.) in DMSO (5 mL) was added dropwise to a solution of potassium *tert*-butoxide (3.57 g, 31.9 mmol, 1.5 equiv.) in DMSO (10 mL) at 0°C under an argon atmosphere. After complete addition, the ice-bath was removed and the reaction mixture was stirred at room temperature for 1 h. Then, a saturated solution of $NaHCO_3$ (10 mL) was added and the aqueous layer was extracted 10 times with cold Et_2O (10x10 mL). The combined organic layers were then washed 10 times with ice-cold brine (10x10 mL) and dried over Na_2SO_4, concentrated under reduced pressure (25° C, 300 mbar) and purified by distillation under reduced pressure (13 mbar) to yield a colorless oil identified as **1b** (2.1 g, 13.8 mmol, 45% yield over 2 steps; bp (13 mbar) = 70 °C).

^{1}H NMR ($CDCl_3$, 400 MHz): δ (ppm) 6.53 (dq, J = 2.9, 0.9 Hz, 1H, C(3)-H), 6.39 – 6.33 (m, 1H, C(2)-H), 3.54 (d, J = 1.1 Hz, 4H, C(1')-H), 2.70 (d, J = 1.1 Hz, 2H, C(4)-H), 1.06 (s, 3H, C(3')-H), 0.93 (s, 3H, C(3')-H).

^{13}C NMR ($CDCl_3$, 101 MHz): δ (ppm) 140.9 (C(3)), 135.8 (C(2)), 102.4 (C(1)), 74.3 (C(1')), 42.5 (C(4)), 30.0 (C(2')), 22.47 (C(3')), 22.46 (C(3')).

IR (neat): 2959 (w), 1473 (w), 1329 (m), 1309 (m), 1292 (m), 1255 (m), 1230 (w), 1137 (s), 1137 (m), 1283 (s), 1048 (m), 1005 (w), 913 (s), 887 (s), 739 (s), 645 (w) cm^{-1}.

HRMS (ESI): m/z calculated for $C_9H_{15}O_2^+$ $[M+H]^+$ = 155.1067; found = 155.1065.

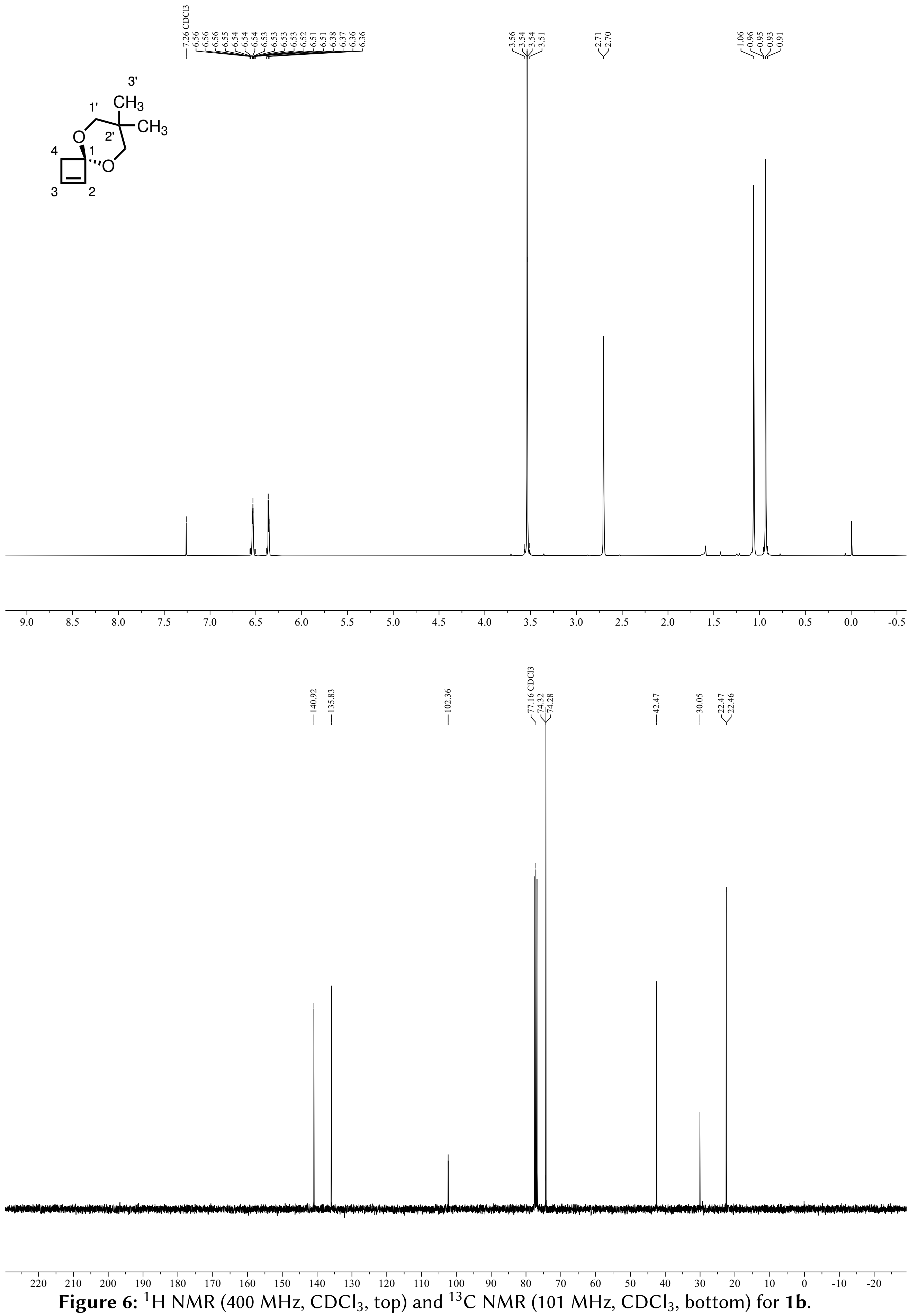

Figure 6: ^{1}H NMR (400 MHz, $CDCl_3$, top) and ^{13}C NMR (101 MHz, $CDCl_3$, bottom) for **1b**.

Synthesis of 1c

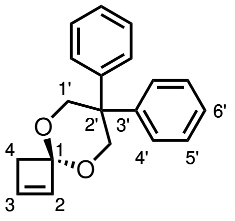

A solution of 2,2-diphenyl-1,3-propanediol[1] (11.5 g, 50.25 mmol, 1.5 equiv.), *p*-TsOH · H_2O (646 mg, 3.4 mmol, 0.1 equiv.) and 2 bromocyclobutanone **S1** (5.0 g, 33.5 mmol) in 150 mL of benzene was stirred under reflux for 4 h, with a Dean-Stark trap attached to the reaction flask.
The reaction mixture was then allowed to cool down to room temperature and the mixture was washed with 50 mL of a saturated $NaHCO_3$ solution, followed by 50 mL of brine. The organic layer was dried over anhydrous Na_2SO_4, filtered and concentrated under reduced prressure. The mixture was used without further purification in the next step.

A solution of the product from the previous step (*vide supra*) (5.0 g, $\leq$13.9 mmol, 1 equiv.) in DMSO (5 mL) was added dropwise to a solution of potassium *tert*-butoxide (2.4 g, 20.9 mmol, 1.5 equiv.) in DMSO (10 mL) at 0 °C under an argon atmosphere. After complete addition, the ice-bath was removed and the reaction mixture was stirred at room temperature for 1 h. Then, a saturated solution of $NaHCO_3$ (10 mL) was added and the aqueous layer was extracted 10 times with cold Et_2O (10x10 mL). The combined organic layers were washed 10 times with ice-cold brine (10x10 mL) and dried over Na_2SO_4, concentrated under reduced and purified by manual flash chromatography (Pentane 95:5 Et_2O) to yield a white solid identified as **1c** (3.28 g, 11.8 mmol, 55% yield over two steps).

^{1}**H NMR** ($CDCl_3$, 400 MHz): δ (ppm) 7.36 – 7.06 (m, 10H, C(Ar)-H), 6.50 (dt, J = 3.1, 1.0 Hz, 1H, C(3)-H), 6.29 (d, J = 3.1 Hz, 1H, C(2)-H), 4.52 – 4.44 (m, 2H, C(1')-H), 4.41 – 4.31 (m, 2H, C(1')-H), 2.72 (d, J = 1.0 Hz, 2H, C(4)-H).

^{13}**C NMR** ($CDCl_3$, 101 MHz): δ (ppm) 143.9 (C(Ar)), 141.1 (C(3)), 135.7 (C(2)), 128.5 (C(Ar)), 128.4 (C(Ar)), 128.1 (C(Ar)), 127.9 (C(Ar)), 127.2 (C(Ar)), 126.9 (C(Ar)), 126.7 (C(Ar)), 126.5 (C(Ar)), 102.6 (C(1)), 71.8 (C(1')), 44.4 (C(4)), 42.5 (C(4)).

[1] *Synthesis* **2015**; 47(15), 2217-2222.

IR (neat): 2981 (s), 2885 (w), 1497 (w), 1383 (m), 1329 (m), 1292 (m), 1254 (m), 1228 (m), 1152 (m), 1123 (m), 1059 (m), 914 (m), 890 (m), 739 (s), 701 (s), 660 (w) cm^{-1}.

HRMS (ESI): m/z calculated for $C_{19}H_{18}O_2Na^+$ $[M+Na]^+$ = 301.1199; found =301.1202.

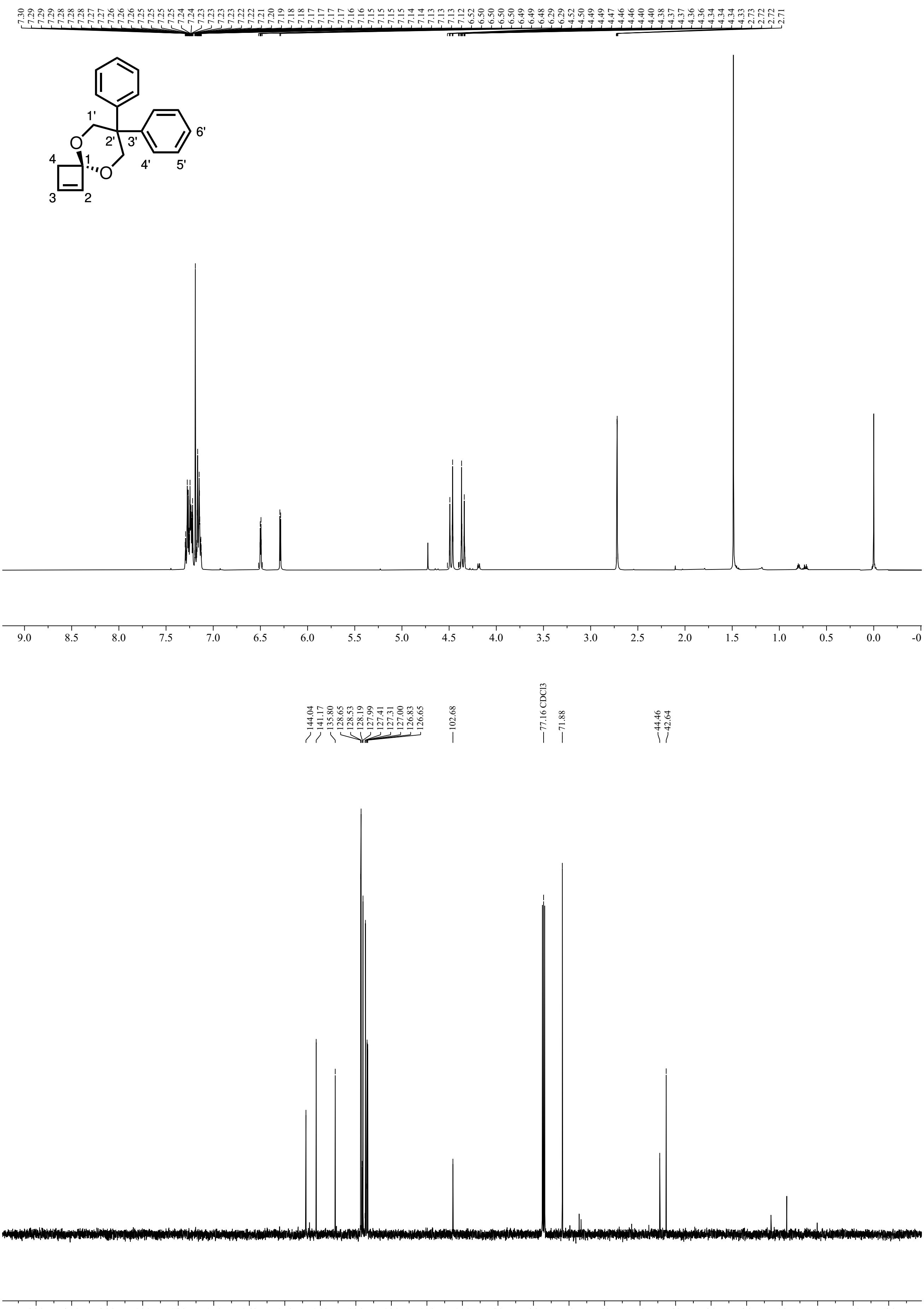

Figure 7: ^{1}H NMR (400 MHz, $CDCl_3$, top) and ^{13}C NMR (101 MHz, $CDCl_3$, bottom) for **1c**.

Synthesis of 1d

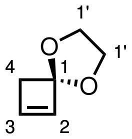

A solution of ethylene glycol (3.12 g, 50.25 mmol, 1.5 equiv.), *p*-TsOH · H_2O (646 mg, 3.4 mmol, 0.1 equiv.) and 2 bromocyclobutanone **S1** (5.0 g, 33.5 mmol) in 150 mL of benzene was stirred under reflux for 4 h, with a Dean-Stark trap attached to the reaction flask.
The reaction mixture was then allowed to cool down to room temperature and the mixture was washed with 50 mL of a saturated $NaHCO_3$ solution, followed by 50 mL of brine. The organic layer was dried over anhydrous Na_2SO_4, filtered and concentrated under reduced pressure. The mixture was used without further purification in the next step.

A solution of the product from the previous step (*vide supra*) (5.0 g, $\leq$25.9 mmol, 1 equiv.) in DMSO (5 mL) was added dropwise to a solution of potassium *tert*-butoxide (4.35 g, 38.8 mmol, 1.5 equiv.) in DMSO (10 mL) at 0° under argon an argon atmosphere. After complete addition, the ice-bath was removed and the reaction mixture was stirred at room temperature for 1 h. Then, a saturated solution of $NaHCO_3$ (10 mL) was added and the aqueous layer was extracted 10 times with cold Et_2O (10x10 mL). The combined organic layers were washed 10 times with ice-cold brine and dried over Na_2SO_4, concentrated under reduced pressure (25°, 300 mbar) and purified by distillation under reduced pressure (13 mabar) to yield a colorless oil identified as **1b** (1.0 g, 9.0 mmol, 23% yield over 2 steps; bp (13 mbar) = 57 °C).

^{1}**H NMR** ($CDCl_3$, 400 MHz): δ (ppm) 6.51 (dt, J = 3.0, 1.0 Hz, 1H, C(3)-H), 6.01 (d, J = 3.0 Hz, 1H, C(2)-H), 4.06 – 3.89 (m, 4H, C(1')-H), 2.80 (d, J = 1.0 Hz, 2H, C(4)-H).

^{13}**C NMR** ($CDCl_3$, 101 MHz): δ (ppm) 141.5 (C(3)), 135.9 (C(2)), 109.5 (C(1)), 64.7 (C(1')), 46.8 (C(4)).

IR (neat): 2928 (w), 1317 (w), 1236 (m), 1050 (m), 912 (m), 738 (s), 650 (w) cm^{-1}.

HRMS (ESI): m/z calculated for $C_6H_9O_2^+$ $[M+H]^+$ = 113.0597; found = 113.0592.

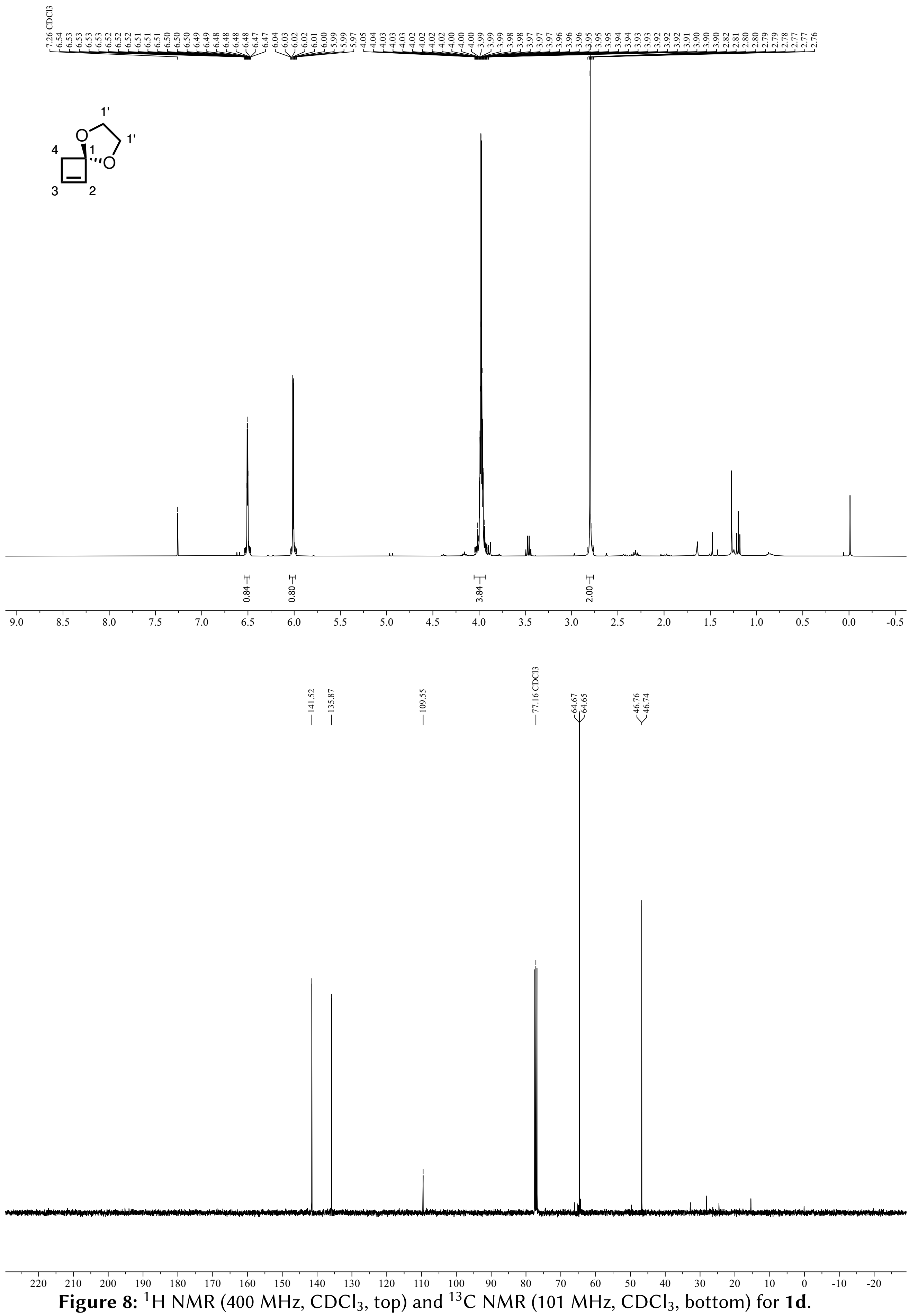

Figure 8: ^{1}H NMR (400 MHz, $CDCl_3$, top) and ^{13}C NMR (101 MHz, $CDCl_3$, bottom) for **1d**.

3.1 General method for the preparation of racemates

A 5 mL flamed-dried round bottom flask was charged with $[Rh(cod)(OH)]_2$ (2.3 mg, 0.005 mmol, 2.5 mol%) and the corresponding aryl boronic acid (0.4 mmol, 2.0 equiv.). The flask was fitted with a rubber septum, connected to an argon-vacuum manifold, evacuated for 1 min and was carefully back-filled with argon (3 vacuum/Ar cycles). Then, dry THF (1.0 mL), CsOH (aqueous solution 50 wt%, 35 µL, 0.40 mmol, 1.0 equiv.) and compound **1** (0.20 mmol, 1.0 equiv.) were added consecutively via syringe to the flask. The resulting mixture was stirred at 40 °C for 15 min. Purification by manual flash chromatography (SiO_2; neutralized with Pentane 95:5 NEt_3) (Pentane 80:20 Et_2O) afforded compound (±)**3**.

3.2 General methods for the preparation of compounds 3aa-w, 3ba-c, 3ca-c and 3da-c

General Procedure A

A 5 mL flamed-dried round bottom flask was charged with $[RhCl(coe)_2]_2$ (7.2 mg, 0.01 mmol, 2.5 mol%) and the corresponding aryl boronic acid (0.8 mmol, 2.0 equiv.). The flask was fitted with a rubber septum, connected to an argon-vacuum manifold, evacuated for 1 min and was carefully back-filled with argon (3 vaccum/Ar cycles). Then, a solution of diene ligand **L1**[2] (5.3 mg, 0.024 mmol, 6.0 mol%) in dry THF (2.0 mL) was added via syringe to the flask. The mixture was let to stir at room temperature for 5 min. Then, CsOH (50 wt%, 70 µL, 0.40 mmol, 1.0 equiv.) was added via syringe and the mixture was let to stir at room temperature for 5 min.Then, compound **1a** (47 µL, 0.40 mmol, 1.0 equiv.) was added via syringe into the flask. After stirring the resulting mixture at room temperature for 15 min, a mixture of Et_2O and NEt_3 (1.0 mL, Et_2O 99:1 NEt_3), and Celite ® (c.a. 1 g) were added, and the solvent was removed under reduced pressure. Purification by manual flash chromatography (SiO_2; neutralized with Pentane 95:5 NEt_3) afforded the desired coupling products.

General Procedure B

Analogous to General Procedure A, but using instead diene ligand **L2** instead of **L1** and stirring the reaction mixture at 60 °C after the addition of **1a** for 15 min.

[2] *Org. Lett.* **2008**, 10, 19, 4387–4389.

General Procedure C

Analogous to General Procedure A, but using compound **1b** instead of **1a**.

General Procedure D

Analogous to General Procedure A, but using compound **1c** instead of **1a**.

General Procedure E

Analogous to General Procedure A, but using compound **1e** instead of **1a**.

Upscale

Larger-scale experiments were performed in direct analogy to General Procedure A with decreased catalyst (1.25 mol% $[RhCl(coe)_2]_2$) and ligand (3 mol% **L1**) loading.

Synthesis of 3aa

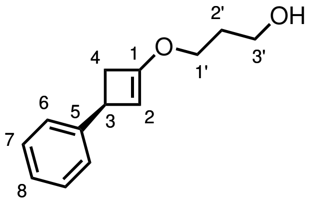

The corresponding compound was prepared following General Procedure A, using phenylboronic acid. Purification by manual flash chromatography (Pentane 80:20 Et_2O) afforded a colorless oil identified as **3aa** (69.4 mg, 85% yield). SFC analysis showed an enantiomeric excess of 95%.

^{1}H NMR (Acetone-d_6, 400 MHz): δ (ppm) 7.31 – 7.24 (m, 4H; C(6)-H, C(7)-H), 7.17 (m, 1H; C(8)-H), 4.83 (d, *J* = 0.9 Hz, 1H; C(2)-H), 4.08 – 3.93 (m, 2H; C(1')-H), 3.68 (td, *J* = 6.2, 5.0 Hz, 2H; C(3')-H), 3.61 – 3.58 (m, 2H; C(3)-H, OH), 3.08 (dd, *J* = 12.8, 4.6 Hz, 1H; C(4)-H), 2.29 (dd, *J* = 12.7, 1.6 Hz, 1H; C(4)-H), 1.91 (p, *J* = 6.3 Hz, 2H; C(2')-H).

^{13}C NMR (Acetone-d_6, 101 MHz): δ (ppm) 154.5 (C(1)), 145.7(C(5)), 129.0 (C(7)), 127.3 (C(6)), 126.9 (C(8)), 99.5 (C(2)), 66.2 (C(1')), 59.0 (C(3')), 41.8 (C(4)), 37.8 (C(3)), 33.0 (C(2')).

IR (neat): 3334 (br), 2925 (w), 1629 (s), 1492 (w), 1452 (w), 1300 (s), 1216 (m), 1195 (w), 1055 (m), 1003 (m), 941 (w), 789 (w), 757 (m), 689 (s) cm^{-1}.

HRMS (ESI): m/z calculated for $C_{13}H_{17}O_2^+$ $[M+H]^+$ = 205.1223; found = 205.1223.

SFC Chiralpak® IF; 1500 psi, 30 °C; flow 1.5 mL/min; from 1% to 30% MeOH in 5 min; 95% ee (minor enantiomer t_R = 2.90 min; major enantiomer t_R = 3.06 min).

$[\alpha]_D^{25}$ = +42.5 (c = 1.00, CH_2Cl_2).

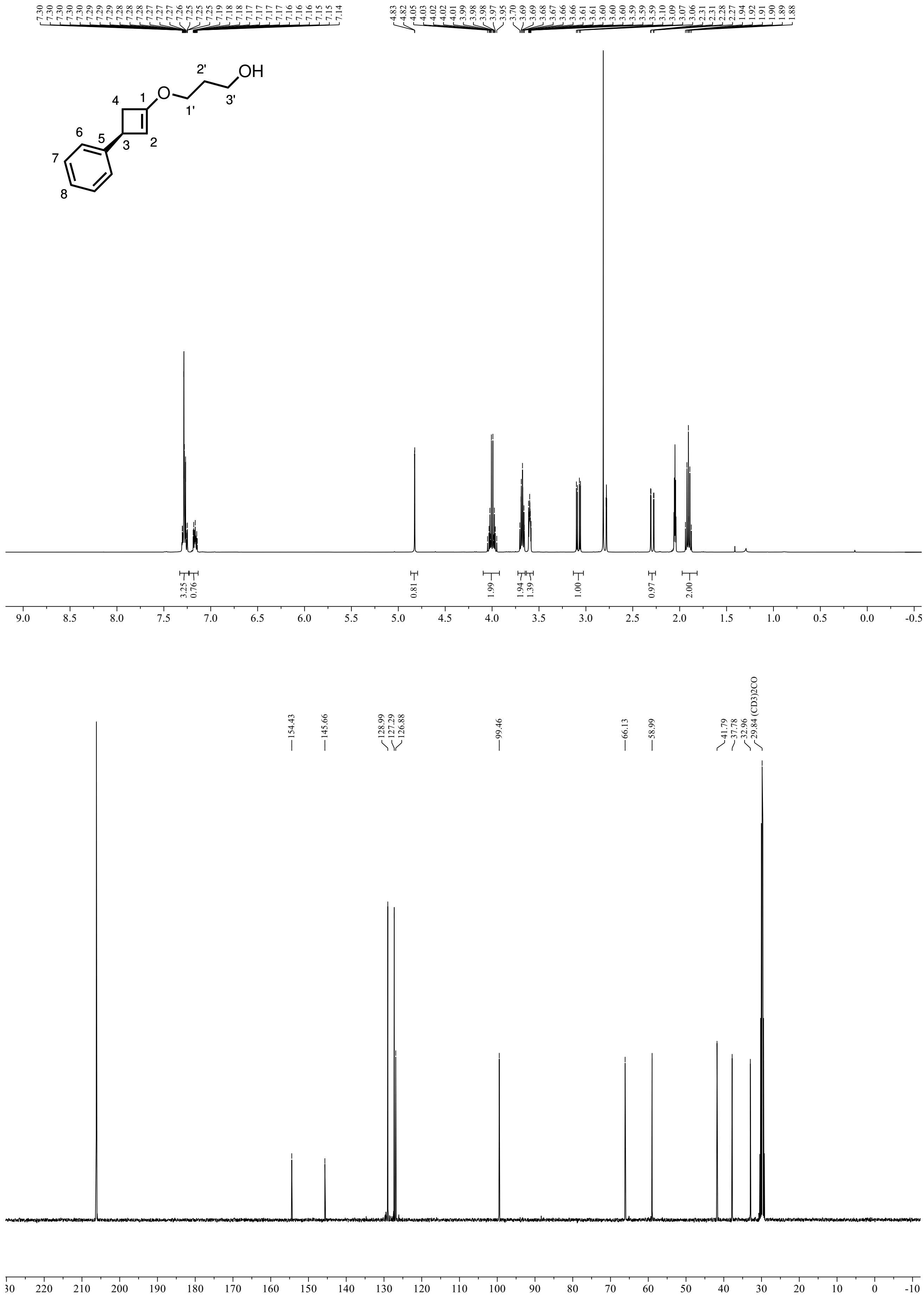

Figure 9: ^{1}H NMR (400 MHz, acetone-d_6, top) and ^{13}C NMR (101 MHz, acetone-d_6, bottom) for **3aa**.

Synthesis of 3ab

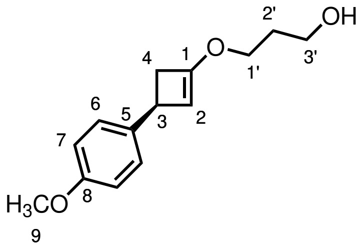

The corresponding compound was prepared following General Procedure A, using 4-methoxyphenylboronic acid. Purification by manual flash chromatography (Pentane 80:20 Et_2O) afforded a colorless oil identified as **3ab** (81.1 mg, 83% yield). SFC analysis showed an enantiomeric excess of 94%.

^{1}H NMR (Acetone-d_6, 400 MHz): δ 7.24 – 7.17 (m, 2H; C(6)-H), 6.88 – 6.82 (m, 2H; C(7)-H), 4.79 (d, *J* = 0.9 Hz, 1H; C(2)-H), 4.06 – 3.93 (m, 2H; C(1')-H), 3.76 (s, 3H; C(9)-H), 3.68 (td, *J* = 6.2, 5.2 Hz, 2H; C(3')-H), 3.60 – 3.52 (m, 2H; C(3)-H, OH), 3.04 (dd, *J* = 12.7, 4.5 Hz, 1H; C(4)-H), 2.24 (dd, *J* = 12.7, 1.6 Hz, 1H; C(4)-H), 1.90 (p, *J* = 6.3 Hz, 2H; C(2')-H).

^{13}C NMR (Acetone-d_6, 101 MHz): δ 159.2 (C(8)), 154.3 (C(1)), 137.5 (C(5)), 128.2 (C(6)), 114.4 (C(7)), 99.7 (C(2)), 66.1 (C(1')), 59.1 (C(3')), 55.5 (C(9)), 42.0 (C(4)), 37.1 (C(3)), 33.0 (C(2')).

IR (neat): 3371 (br), 2924 (w), 1630 (s), 1611 (w), 1582 (w), 1510 (s), 1465 (w), 1298 (s), 1243 (s), 1215 (m), 1196 (m), 1174 (m), 1056 (m), 1035 (s), 1004 (s), 942 (m), 804 (m), 777 (m), 688 (w) cm^{-1}.

HRMS (ESI): m/z calculated for $C_{14}H_{19}O_3^+$ $[M+H]^+$ = 235.1329; found = 235.1331.

SFC Chiralpak ® IF; 1500 psi, 30 °C; flow 1.5 mL/min; from 1% to 30% MeOH in 5 min; 94% ee (minor enantiomer t_R = 3.56 min; major enantiomer t_R = 3.89 min).

$[\alpha]_D^{25}$ = +36.7 (c = 1.00, CH_2Cl_2).

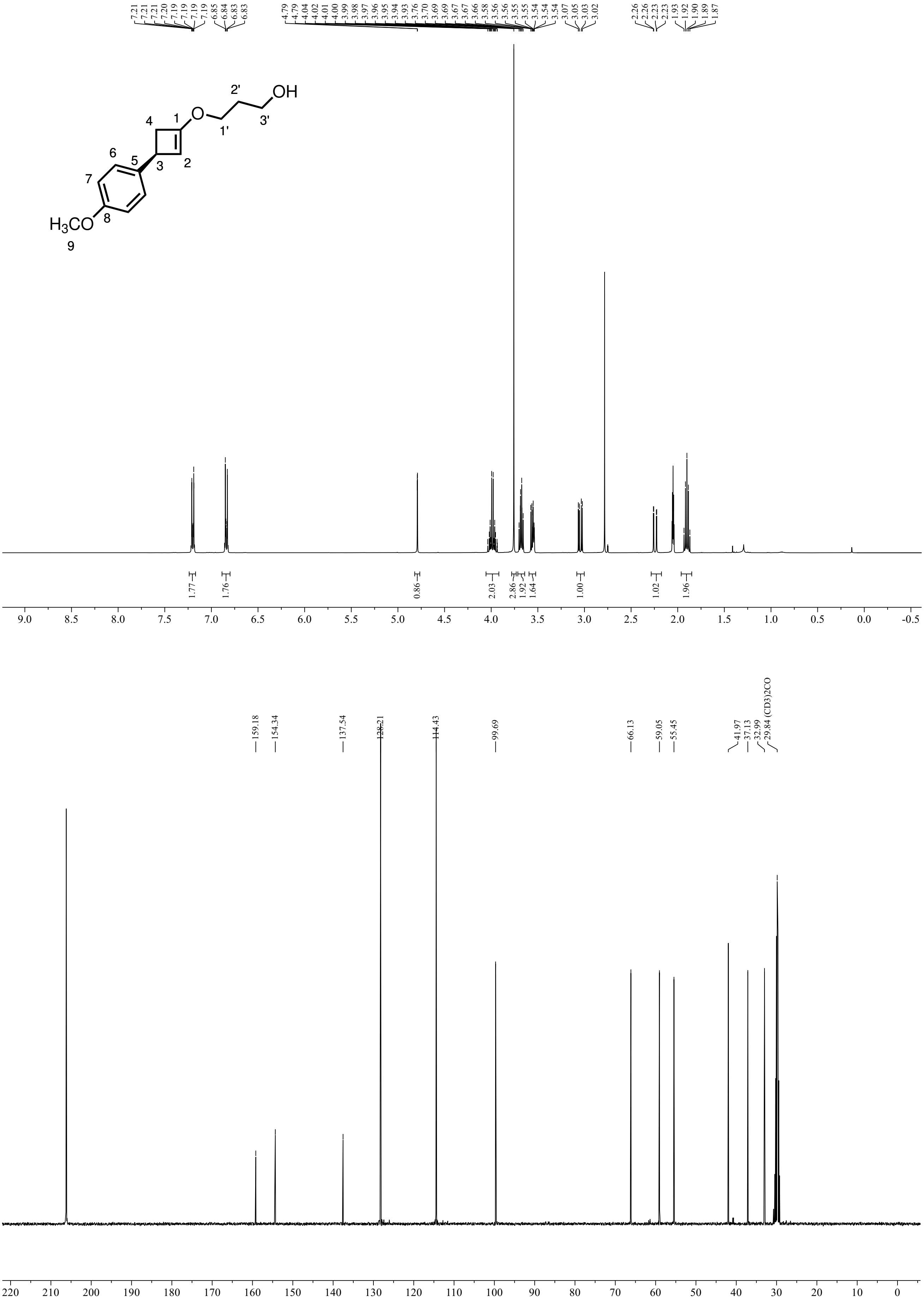

Figure 10: ^{1}H NMR (400 MHz, acetone-d_6, top) and ^{13}C NMR (101 MHz, acetone-d_6, bottom) for **3ab**.

Synthesis of 3ac

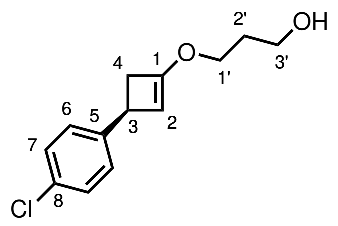

The corresponding compound was prepared following General Procedure A, using 4-chlorophenylboronic acid. Purification by manual flash chromatography (Pentane 80:20 Et_2O) afforded a colorless oil identified as **3ac** (71.8 mg, 75% yield). SFC analysis showed an enantiomeric excess of 97%.

^{1}H NMR (Acetone-d_6, 400 MHz): δ (ppm) 7.30 (d, *J* = 0.8 Hz, 4H; C(6)-H, C(7)-H), 4.81 (d, *J* = 0.9 Hz, 1H; C(2)-H), 4.11 – 3.92 (m, 2H; C(1')-H), 3.68 (td, *J* = 6.2, 5.1 Hz, 2H; C(3')-H), 3.64 – 3.58 (m, 2H; C(3)-H, OH), 3.09 (dd, *J* = 12.8, 4.6 Hz, 1H; C(4)-H), 2.28 (dd, *J* = 12.8, 1.6 Hz, 1H; C(4)-H), 1.90 (p, *J* = 6.3 Hz, 2H; C(2')-H).

^{13}C NMR (Acetone-d_6, 101 MHz): δ (ppm) 154.7 (C(1)), 144.8 (C(5)), 132.1 (C(8)), 129.1 (C(6), C(7)), 99.3 (C(2)), 66.3 (C(1')), 59.0 (C(3')), 41.8 (C(4)), 37.2 (C(3)), 33.0 (C(2')).

IR (neat): 3352 (br), 2924 (w), 1631 (s), 1469 (w), 1298 (s), 1213 (m), 1156 (w), 1057 (m), 1004 (w), 943 (w), 776 (m), 703 (m) cm^{-1}.

HRMS (ESI): m/z calculated for $C_{13}H_{16}O_2^+Cl$ $[M+H]^+$ = 239.0833; found = 239.0834.

SFC Chiralpak ® IF; 1500 psi, 30 °C; flow 1.5 mL/min; from 1% to 30% MeOH in 5 min; 97% ee (minor enantiomer t_R = 3.39 min; major enantiomer t_R = 3.59 min).

$[\alpha]_D^{25}$ = +29.0 (c = 1.00, CH_2Cl_2).

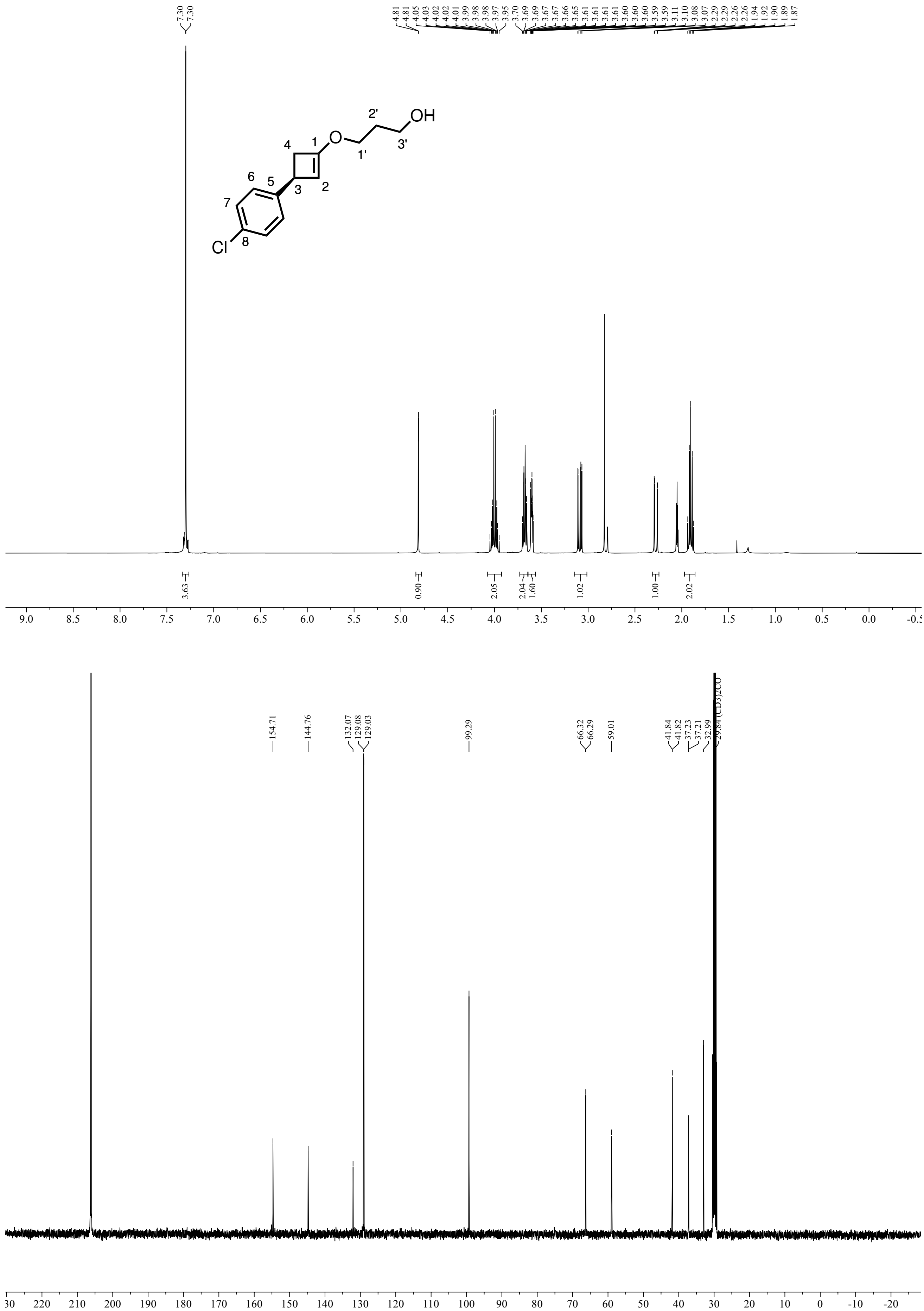

Figure 11: ^{1}H NMR (400 MHz, acetone-d$_6$, top) and ^{13}C NMR (101 MHz, acetone-d$_6$, bottom) for **3ac**.

Synthesis of 3ad

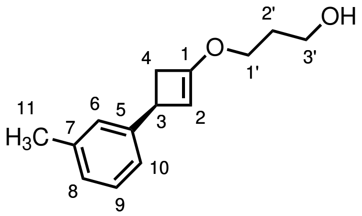

The corresponding compound was prepared following General Procedure A, using *m*-tolylboronic acid. Purification by manual flash chromatography (Pentane 80:20 Et_2O) afforded a colorless oil identified as **3ad** (77.6 mg, 89% yield). SFC analysis showed an enantiomeric excess of 91%.

^{1}H NMR (Acetone-d_6, 400 MHz): δ (ppm) 7.07 – 6.91 (m, 3H; C(8)-H, C(9)-H, C(10)-H), 6.87 – 6.83 (m, 1H; C(6)-H), 4.68 (d, *J* = 0.9 Hz, 1H; C(2)-H), 3.95 – 3.78 (m, 2H; C(1')-H), 3.55 (td, *J* = 6.2, 5.1 Hz, 2H; C(3')-H), 3.48 – 3.40 (m, 2H; C(3)-H, OH), 2.93 (dd, *J* = 12.7, 4.6 Hz, 1H; C(4)-H), 2.31 – 2.07 (m, 4H; C(4)-H, C(11)-H), 1.77 (p, *J* = 6.3 Hz, 2H; C(2')-H).

^{13}C NMR (Acetone-d_6, 101 MHz): δ (ppm) 154.3 (C(1)), 145.6 (C(5)), 138.3 (C(7)), 128.9 (C(Ar)), 128.0 (C(Ar)), 127.6 (C(Ar)), 124.4 (C(Ar)), 99.5 (C(2)), 66.1 (C(1')), 59.0 (C(3')), 41.7 (C(4)), 37.7 (C(3)), 33.0 (C(2')), 21.4 (C(9)).

IR (neat): 3350 (br), 2924 (w), 2360 (w), 1632 (s), 1469 (w), 1299 (m), 1213 (w), 1058 (m), 944 (w), 777 (w), 703 (w) cm^{-1}.

HRMS (ESI): m/z calculated for $C_{14}H_{19}O_2^+$ $[M+H]^+$ = 219.1380; found = 219.1381.

SFC Chiralpak ® IF; 1500 psi, 30 °C; flow 1.5 mL/min; from 1% to 30% MeOH in 5 min; 91% ee (minor enantiomer t_R = 2.84 min; major enantiomer t_R = 2.91 min).

$[\alpha]_D^{25}$ = +42.0 (c = 0.15, CH_2Cl_2).

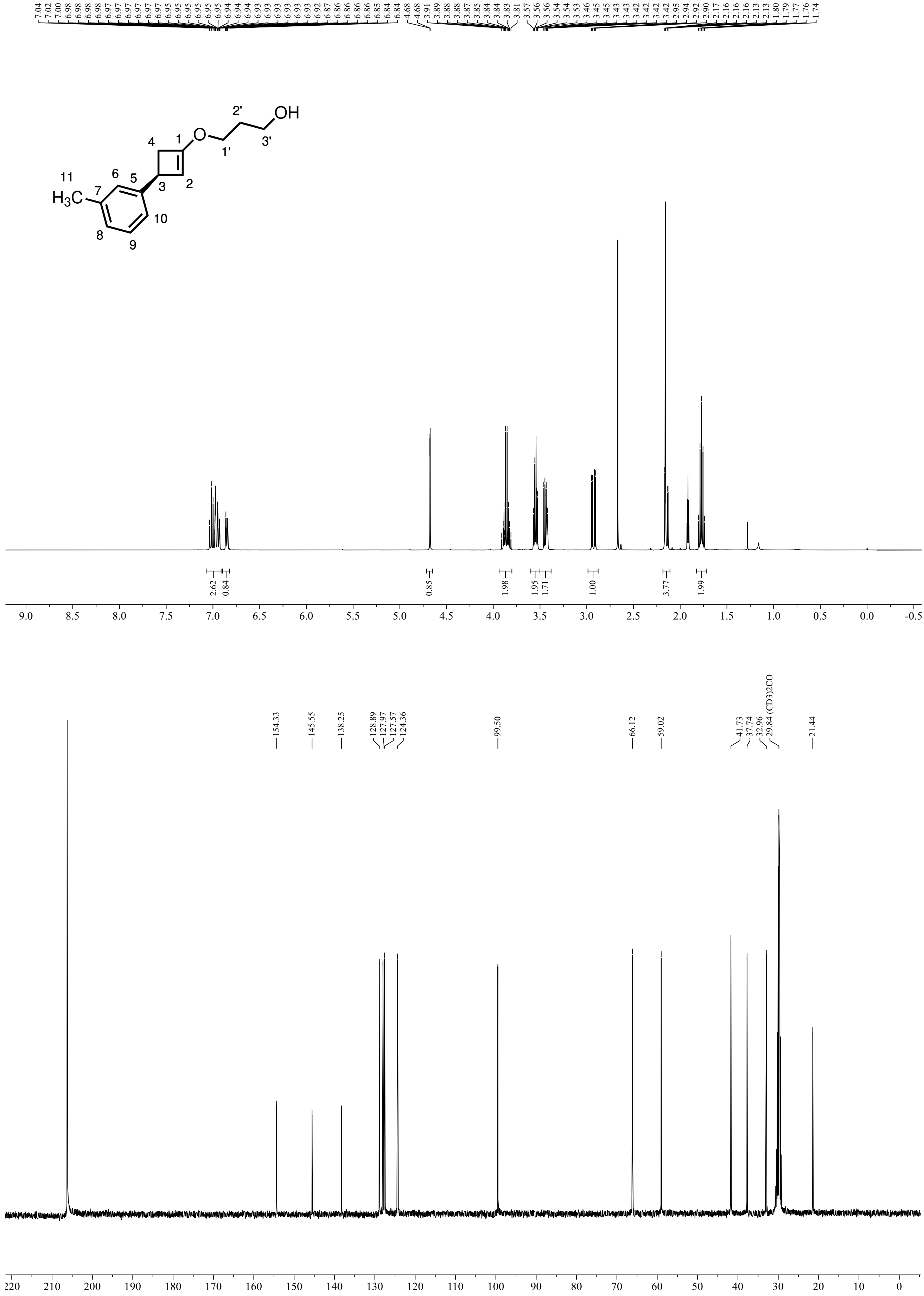

Figure 12: ^{1}H NMR (400 MHz, acetone-d_6, top) and ^{13}C NMR (101 MHz, acetone-d_6, bottom) for **3ad**.

Synthesis of 3ae

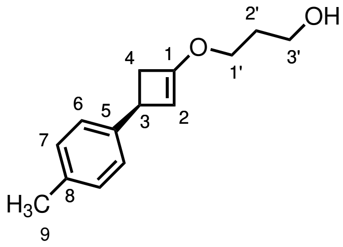

The corresponding compound was prepared following General Procedure A, using *p*-tolylboronic acid. Purification by manual flash chromatography (Pentane 80:20 Et_2O) afforded a colorless oil identified as **3ae** (80.8 mg, 93% yield). SFC analysis showed an enantiomeric excess of 93%.

^{1}H NMR (Acetone-d_6, 400 MHz): δ (ppm) 7.19 – 7.15 (m, 2H; C(6)-H), 7.10 – 7.07 (m, 2H; C(7)-H), 4.80 (d, *J* = 0.9 Hz, 1H; C(2)-H), 4.06 – 3.92 (m, 2H; C(1')-H), 3.68 (td, *J* = 6.2, 5.1 Hz, 2H; C(3')-H), 3.61 – 3.53 (m, 2H; C(3)-H, OH), 3.05 (dd, *J* = 12.7, 4.6 Hz, 1H, C(4)-H), 2.33 – 2.22 (m, 4H, C(4)-H, C(9)-H), 1.90 (p, *J* = 6.3 Hz, 2H; C(2')-H).

^{13}C NMR (Acetone-d_6, 101 MHz): δ (ppm) 154.4 (C(1)), 142.6 (C(5)), 136.1 (C(8)), 129.6 (C(6)), 127.2 (C(7)), 99.6 (C(2)), 66.1 (C(1')), 59.0 (C(3')), 41.8 (C(4)), 37.5 (C(3)), 33.0 (C(2')), 21.1 (C(9)).

IR (neat): 3332 (br), 2923 (w), 1630 (s), 1513 (w), 1430 (w), 1305 (s), 1293 (s), 1215 (m), 1193 (w), 1056 (m), 1005 (m), 939 (w), 815 (m), 775 (m), 718 (w), 690 (w) cm^{-1}.

HRMS (ESI): m/z calculated for $C_{14}H_{19}O_2^+$ $[M+H]^+$ = 219.1380; found = 219.1382.

SFC Chiralpak ® IF; 1500 psi, 30 °C; flow 1.5 mL/min; from 1% to 30% MeOH in 5 min; 93% ee (minor enantiomer t_R = 3.09 min; major enantiomer t_R = 3.30 min).

$[\alpha]_D^{25}$ = +37.0 (c = 1.00, CH_2Cl_2).

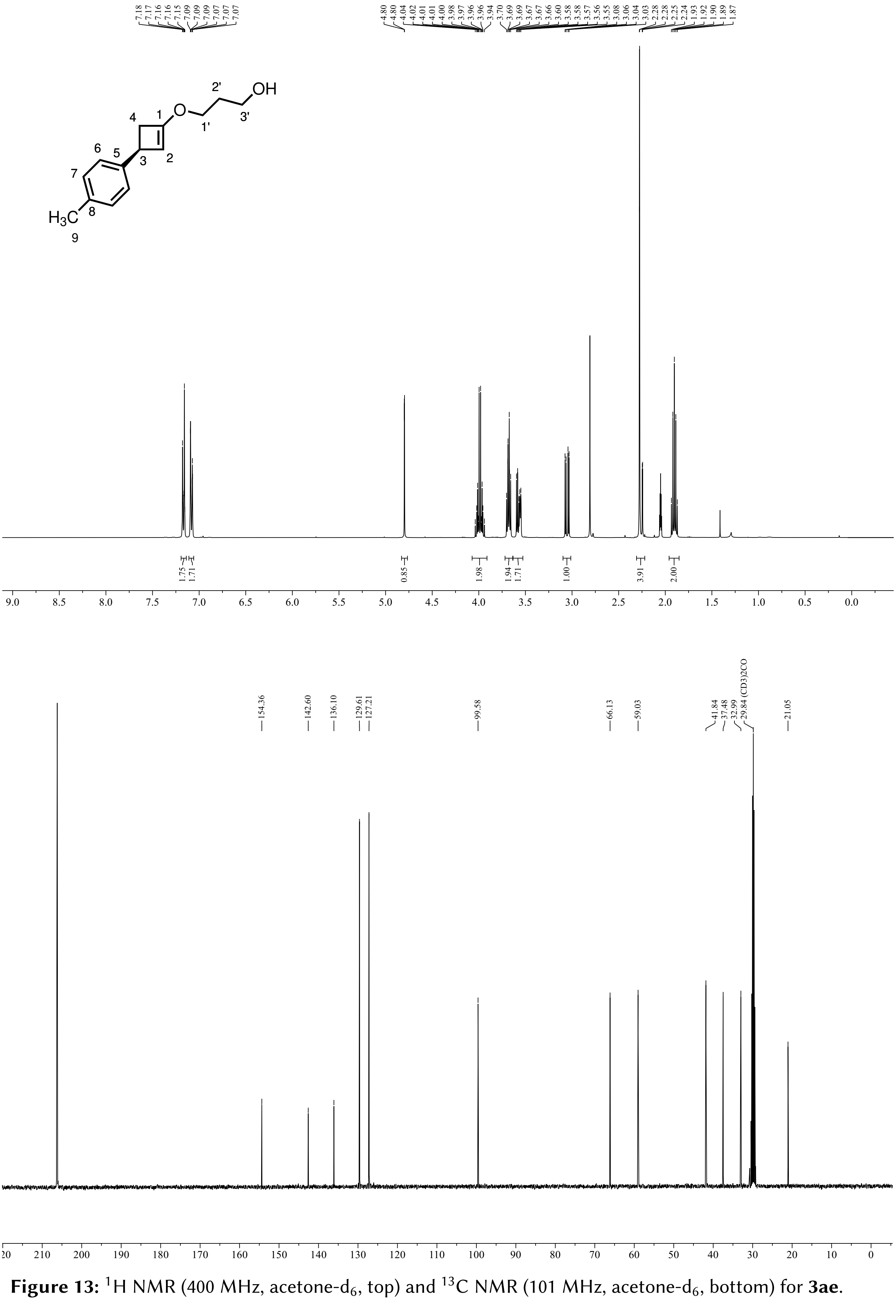

Figure 13: ^{1}H NMR (400 MHz, acetone-d$_6$, top) and ^{13}C NMR (101 MHz, acetone-d$_6$, bottom) for **3ae**.

Synthesis of 3af

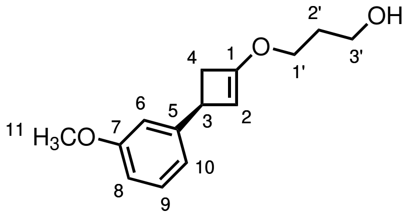

The corresponding compound was prepared following General Procedure A, using 3-methoxyphenylboronic acid. Purification by manual flash chromatography (Pentane 80:20 Et2O) afforded a colorless oil identified as **3af** (81.7 mg, 87% yield). SFC analysis showed an enantiomeric excess of 92%.

^{1}H NMR (Acetone-d_6, 400 MHz): δ (ppm) 7.22 – 7.16 (m, 1H; C(Ar)-H), 6.91 – 6.84 (m, 2H; C(Ar)-H), 6.78 – 6.69 (m, 1H; C(Ar)-H), 4.81 (d, *J* = 0.9 Hz, 1H; C(2)-H), 4.09 – 3.89 (m, 2H; C(1')-H), 3.77 (s, 3H; C(11)-H), 3.68 (td, *J* = 6.3, 5.1 Hz, 2H; C(3')-H), 3.62 – 3.56 (m, 2H; C(3)-H, OH), 3.06 (dd, *J* = 12.7, 4.6 Hz, 1H; C(4)-H), 2.29 (dd, *J* = 12.7, 1.6 Hz, 1H; C(4)-H), 1.90 (p, *J* = 6.3 Hz, 2H; C(2')-H).

^{13}C NMR (Acetone-d_6, 101 MHz): δ (ppm) 160.8 (C(7)), 154.5 (C(1)), 147.4 (C(5)), 130.0 (C(9)), 119.6 (C(10)), 113.0 (C(6)), 112.3 (C(8)), 99.4 (C(2)), 66.2 (C(1')), 59.0 (C(3')), 55.4 (C(11)), 41.7 (C(4)), 37.9 (C(3)), 33.0 (C(2')).

IR (neat): 3395 (br), 2923 (w), 1631 (s), 1601 (w), 1485 (w), 1466 (w), 1434 (w), 1301 (s), 1284 (m), 1214 (m), 1154 (w), 1050 (m), 1008 (m), 864 (w), 774 (m), 699 (m) cm^{-1}.

HRMS (ESI): m/z calculated for $C_{14}H_{19}O_3^+$ $[M+H]^+$ = 235.1329; found = 235.1331.

SFC Chiralpak ® IF; 1500 psi, 30 °C; flow 1.5 mL/min; from 1% to 30% MeOH in 5 min; 92% ee (minor enantiomer t_R = 3.51 min; major enantiomer t_R = 3.75 min).

$[\alpha]_D^{25}$ = +38.9 (c = 1.03, CH_2Cl_2).

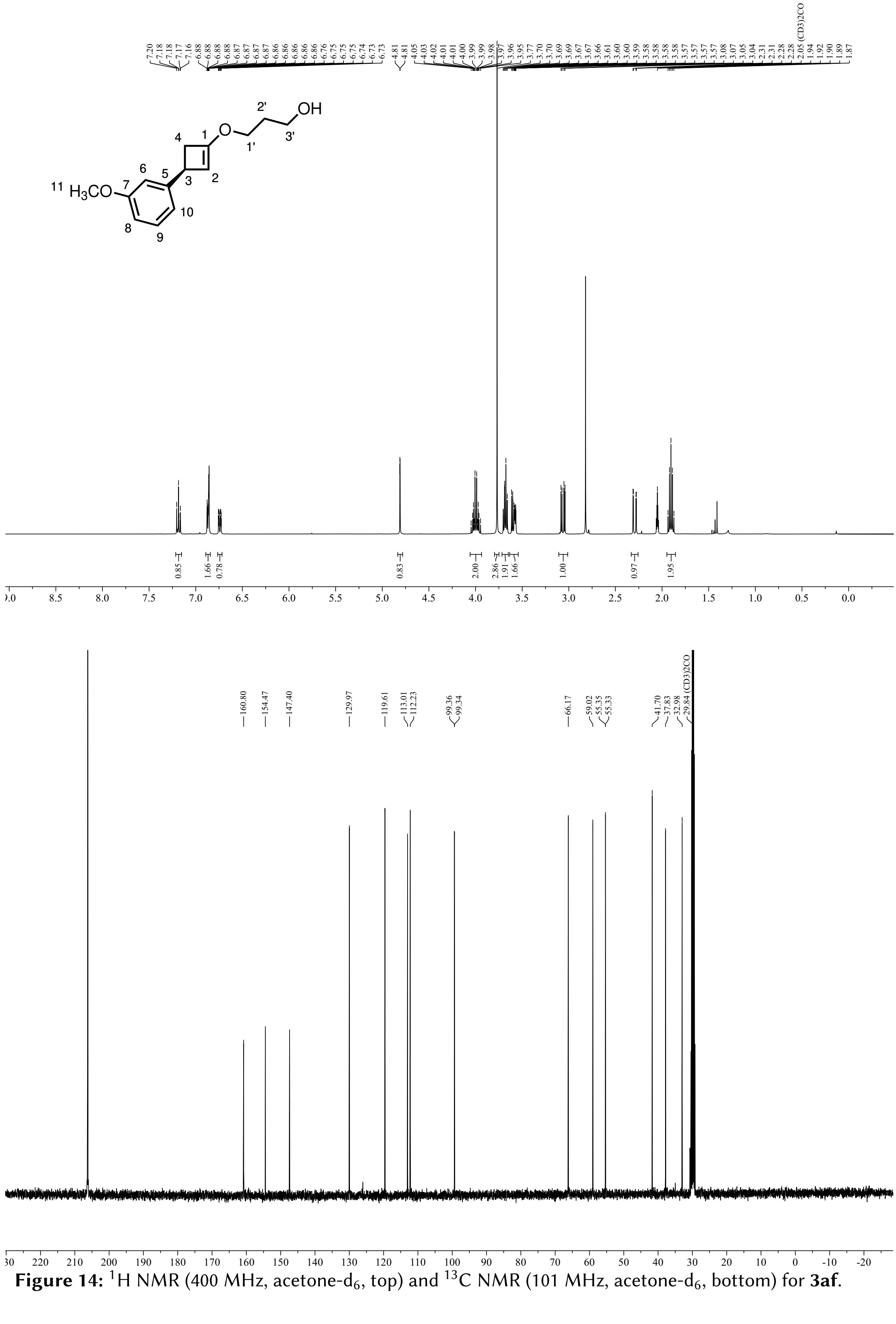

Figure 14: ^{1}H NMR (400 MHz, acetone-d_6, top) and ^{13}C NMR (101 MHz, acetone-d_6, bottom) for **3af**.

Synthesis of 3ag

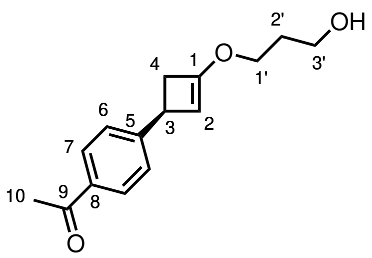

The corresponding compound was prepared following General Procedure A, using 4-acetyl-phenylboronic acid. Purification by manual flash chromatography (Pentane 80:20 Et_2O) afforded a colorless oil identified as **3ag** (71.3 mg, 72% yield). SFC analysis showed an enantiomeric excess of 98%.

^{1}H NMR (Acetone-d_6, 400 MHz): δ (ppm) 7.95 – 7.89 (m, 2H; C(6)-H), 7.47 – 7.39 (m, 2H; C(7)-H), 4.86 (d, *J* = 1.0 Hz, 1H; C(2)-H), 4.08 – 3.95 (m, 2H; C(1')-H), 3.69 (td, *J* = 6.2, 5.1 Hz, 3H; C(3')-H, OH), 3.59 (dd, *J* = 5.5, 4.8 Hz, 1H; C(3)-H), 3.13 (dd, *J* = 12.9, 4.6 Hz, 1H; C(4)-H), 2.55 (s, 3H; C(10)-H), 2.33 (dd, *J* = 12.9, 1.5 Hz, 1H; C(4)-H), 1.91 (p, *J* = 6.3 Hz, 2H; C(2')-H).

^{13}C NMR (Acetone-d_6, 101 MHz): δ (ppm) 197.5 (C(9)), 154.8 (C(1)), 151.8 (C(5)), 136.6 (C(8)), 129.2 (C(6)), 127.6 (C(7)), 99.3 (C(2)), 66.3 (C(1')), 59.0 (C(3')), 41.7 (C(4)), 37.8 (C(3)), 33.0 (C(2')), 26.6 (C(10)).

IR (neat): 3412 (br), 2923 (w), 1679 (m), 1631 (s), 1605 (m), 1568 (w), 1413 (w), 1359 (w), 1305 (s), 1270 (s), 1218 (m), 1180 (w), 1057 (m), 1006 (w), 959 (w), 830 (w), 791 (w), 764 (w), 729 (w) cm^{-1}.

HRMS (ESI): m/z calculated for $C_{15}H_{19}O_3^+$ $[M+H]^+$ = 247.1329; found = 247.1325.

SFC Chiralpak ® IF; 1500 psi, 30 °C; flow 1.5 mL/min; from 1% to 30% MeOH in 8 min; 98% ee (minor enantiomer t_R = 6.19 min; major enantiomer t_R = 6.96 min).

$[\alpha]_D^{25}$ = +32.0 (c = 1.04, CH_2Cl_2).

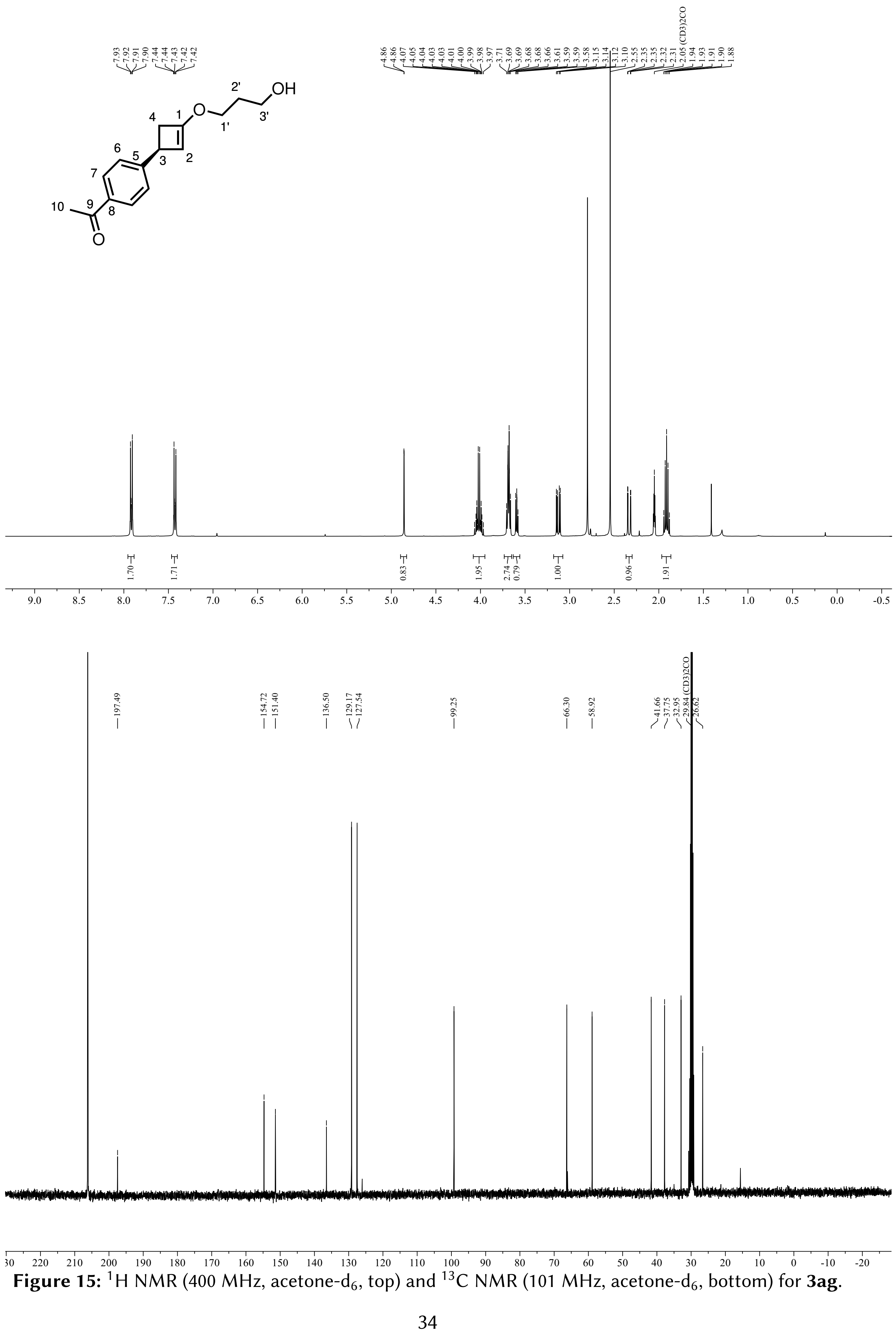

Figure 15: ^{1}H NMR (400 MHz, acetone-d$_6$, top) and ^{13}C NMR (101 MHz, acetone-d$_6$, bottom) for **3ag**.

Synthesis of 3ah

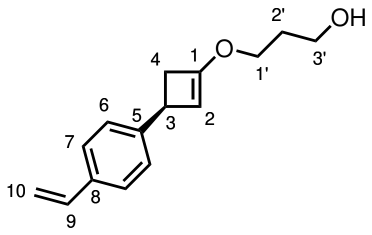

The corresponding compound was prepared following General Procedure A, using 4-vinyl-phenyl-boronic acid. Purification by manual flash chromatography (Pentane 80:20 Et_2O) afforded a colorless oil identified as **3ah** (76.5 mg, 83% yield). SFC analysis showed an enantiomeric excess of 96%.

^{1}H NMR (Acetone-d_6, 400 MHz): δ (ppm) 7.40 – 7.36 (m, 2H; C(7)-H), 7.28 – 7.24 (m, 2H; C(6)-H), 6.72 (dd, *J* = 17.6, 10.9 Hz, 1H; C(9)-H), 5.74 (dd, *J* = 17.7, 1.1 Hz, 1H; C(10)-H), 5.17 (dd, *J* = 10.9, 1.1 Hz, 1H; C(10)-H), 4.82 (d, *J* = 0.9 Hz, 1H; C(2)-H), 4.08 – 3.93 (m, 2H; C(1')-H), 3.68 (td, *J* = 6.2, 5.1 Hz, 2H; C(3')-H), 3.59 (m, 2H; C(3')-H, OH), 3.08 (dd, *J* = 12.8, 4.6 Hz, 1H; C(4)-H), 2.29 (dd, *J* = 12.7, 1.6 Hz, 1H; C(4)-H), 1.91 (p, *J* = 6.3 Hz, 2H; C(2')-H).

^{13}C NMR (Acetone-d_6, 101 MHz): δ (ppm) 154.5 (C(1)), 145.6 (C(5)), 137.8 (C(9)), 136.5 (C(8)), 127.6 (C(7)), 127.0 (C(6)), 113.2 (C(10)), 99.5 (C(2)), 66.2 (C(1')), 59.0 (C(3')), 41.8 (C(4)), 37.6 (C(3)), 33.0 (C(2')).

IR (neat): 3309 (br), 2916 (w), 1630 (s), 1511 (w), 1468 (w), 1407 (w), 1302 (s), 1216 (w), 1056 (m), 907 (m), 838 (m) cm^{-1}.

HRMS (ESI): m/z calculated for $C_{15}H_{19}O_2^+$ $[M+H]^+$ = 231.1380; found = 231.1380.

SFC Chiralpak ® IF; 1500 psi, 30 °C; flow 1.5 mL/min; from 1% to 30% MeOH in 5 min; 96% ee (minor enantiomer t_R = 3.56 min; major enantiomer t_R = 3.92 min).

$[\alpha]_D^{25}$ = +26.3 (c = 0.99, CH_2Cl_2).

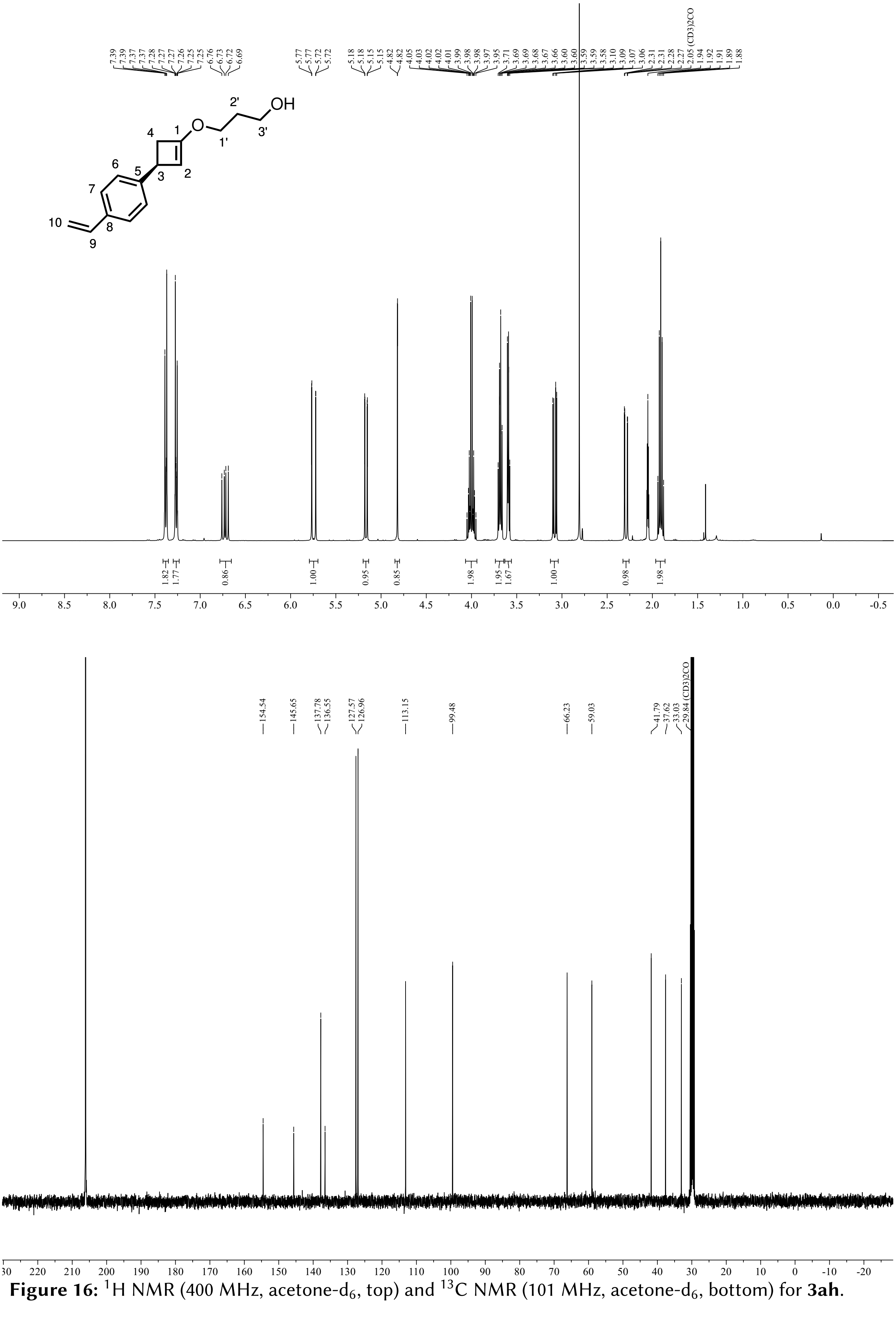

Figure 16: ^{1}H NMR (400 MHz, acetone-d$_6$, top) and ^{13}C NMR (101 MHz, acetone-d$_6$, bottom) for **3ah**.

Synthesis of 3ai

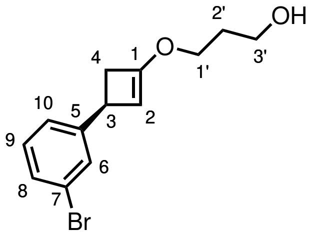

The corresponding compound was prepared following General Procedure A, using 3-bromophenylboronic acid. Purification by manual flash chromatography (Pentane 80:20 Et_2O) afforded a colorless oil identified as **3ai** (88.4 mg, 78% yield). SFC analysis showed an enantiomeric excess of 96%.

^{1}H NMR (Acetone-d$_6$, 400 MHz): δ (ppm) 7.49 – 7.44 (m, 1H; C(6)-H), 7.36 (ddd, *J* = 7.7, 2.0, 1.2 Hz, 1H; C(10)-H), 7.32 – 7.28 (m, 1H; C(8)-H), 7.23 (t, *J* = 7.7 Hz, 1H; C(9)-H), 4.83 (d, *J* = 0.9 Hz, 1H; C(2)-H), 4.09 – 3.93 (m, 2H; C(1')-H), 3.68 (td, *J* = 6.3, 5.1 Hz, 2H; C(3')-H), 3.63 – 3.55 (m, 2H; C(3)-H, OH), 3.10 (dd, *J* = 12.9, 4.6 Hz, 1H; C(4)-H), 2.30 (dd, *J* = 12.9, 1.5 Hz, 1H; C(4)-H), 1.91 (p, *J* = 6.3 Hz, 2H; C(2')-H).

^{13}C NMR (Acetone-d$_6$, 101 MHz): δ (ppm) 154.7 (C(1)), 148.7 (C(5)), 131.0 (C(6)), 130.2 (C(9)), 129.9 (C(8)), 126.4 (C(10)), 122.8 (C(x)7, 99.0 (C(2)), 66.3 (C(1')), 59.0 (C(3')), 41.8 (C(4)), 37.4 (C(3)), 32.9 (C(2')).

IR (neat): 3329 (br), 2925 (w), 1630 (s), 1592 (w), 1563 (w), 1472 (w), 1421 (w), 1300 (s), 1216 (w), 1193 (w), 1056 (m), 996 (m), 943 (w), 876 (w), 762 (m), 693 (m) cm^{-1}.

HRMS (ESI): m/z calculated for $C_{13}H_{16}O_2[^{79}Br]^+$ $[M+H]^+$ = 283.0328; found = 283.0329. m/z calculated for $C_{13}H_{16}O_2[^{81}Br]^+$ $[M+H]^+$ = 285.0308; found = 285.0309.

SFC Chiralpak ® IF; 1500 psi, 30 °C; flow 1.5 mL/min; from 1% to 30% MeOH in 5 min; 96% ee (minor enantiomer t_R = 3.41 min; major enantiomer t_R = 3.53 min).

$[\alpha]_D^{25}$ = +29.4 (c = 0.98, CH_2Cl_2).

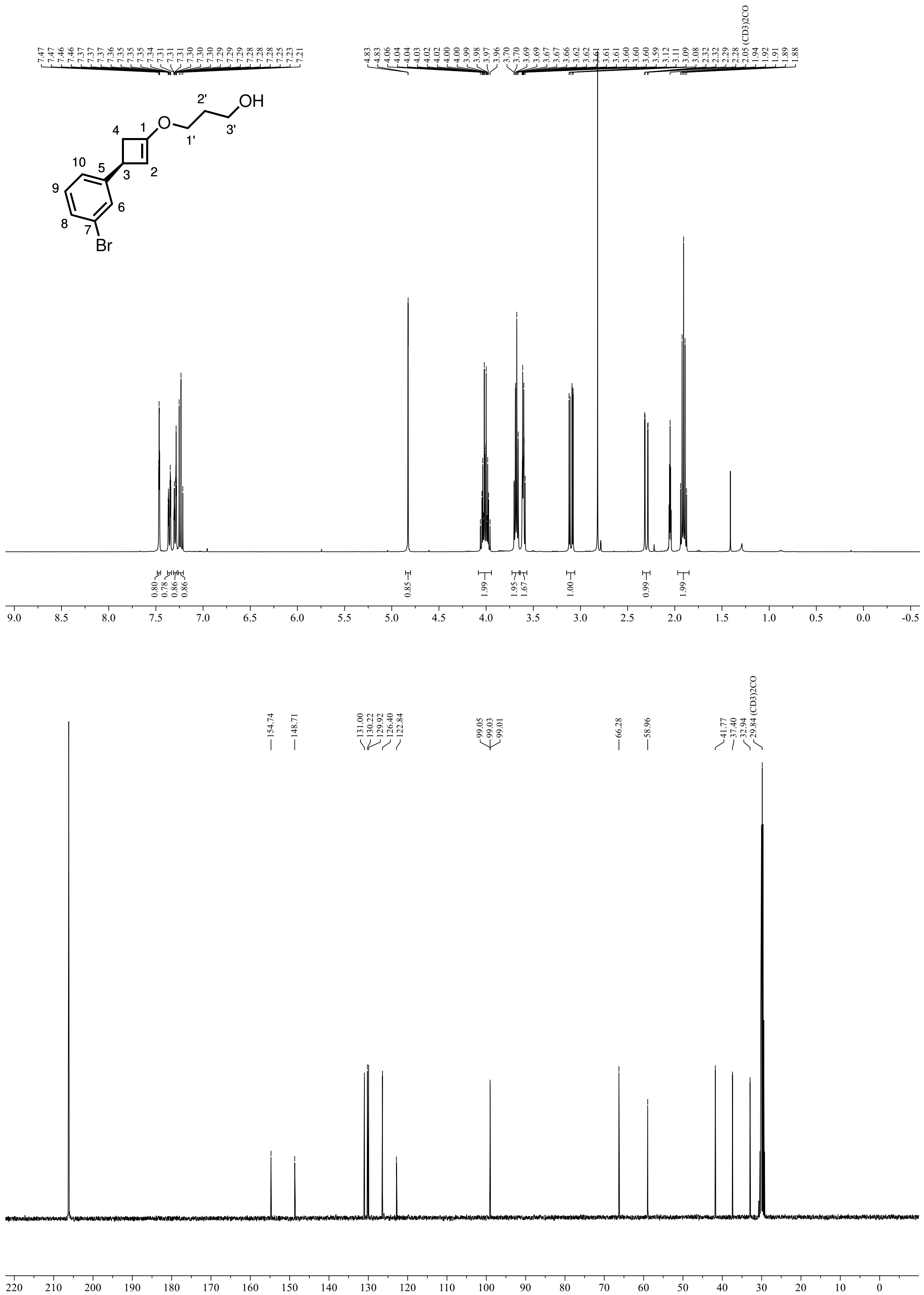

Figure 17: ^{1}H NMR (400 MHz, acetone-d_6, top) and ^{13}C NMR (101 MHz, acetone-d_6, bottom) for **3ai**.

Synthesis of 3aj

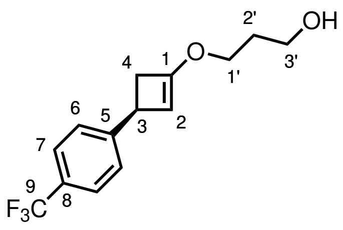

The corresponding compound was prepared following General Procedure A, using 4-(trifluoromethyl)phenylboronic acid. Purification by manual flash chromatography (Pentane 80:20 Et_2O) afforded a colorless oil identified as **3j** (75.0 mg, 69% yield). SFC analysis showed an enantiomeric excess of 98%.

^{1}H NMR (Acetone-d_6, 400 MHz): δ (ppm) 7.69 – 7.59 (m, 2H; C(7)-H), 7.56 – 7.48 (m, 2H; C(6)-H), 4.86 (d, *J* = 0.9 Hz, 1H; C(2)-H), 4.09 – 3.95 (m, 2H; C(1')-H), 3.74 – 3.66 (m, 3H; C(3')-H, OH), 3.61 (dd, *J* = 5.6, 4.7 Hz, 1H; C(3)-H), 3.14 (dd, *J* = 12.9, 4.6 Hz, 1H; C(4)-H), 2.34 (dd, *J* = 13.0, 1.6 Hz, 1H; C(4)-H), 1.91 (p, *J* = 6.3 Hz, 2H; C(2')-H).

^{13}C NMR (Acetone-d_6, 101 MHz): δ (ppm) 154.9 (C(1)), 150.7 (C(5)), 128.7 (d, *J* =31.8 Hz, C(8)), 128.1 (C(6)), 125.9 (q, *J* =3.8 Hz, C(7)), 125.6 (d, *J* =272.7 Hz, C(9)), 99.2 (C(2)), 66.4 (C(1')), 59.0 (C(3')), 41.7 (C(4)), 37.6 (C(3)), 33.0 (C(2')).

^{19}F NMR (Acetone-d_6, 376 MHz): δ (ppm) -62.73 (br. s; CF3).

IR (neat): 3327 (br), 2930 (w), 1633 (m), 1417 (s), 1303 (m), 1219 (w), 1163 (m), 1122 (m), 1067 (m), 1017 (w), 837 (w), 795 (w), 794 (w), 773 (w) cm^{-1}.

HRMS (ESI): m/z calculated for $C_{14}H_{16}O_2F_3^+$ $[M+H]^+$ = 273.1097; found = 273.1096.

SFC Chiralpak ® IF; 1500 psi, 30 °C; flow 1.5 mL/min; from 1% to 30% MeOH in 5 min; 98% ee (minor enantiomer t_R = 2.40 min; major enantiomer t_R = 2.57 min).

$[\alpha]_D^{25}$ = +29.2 (c = 0.98, CH_2Cl_2).

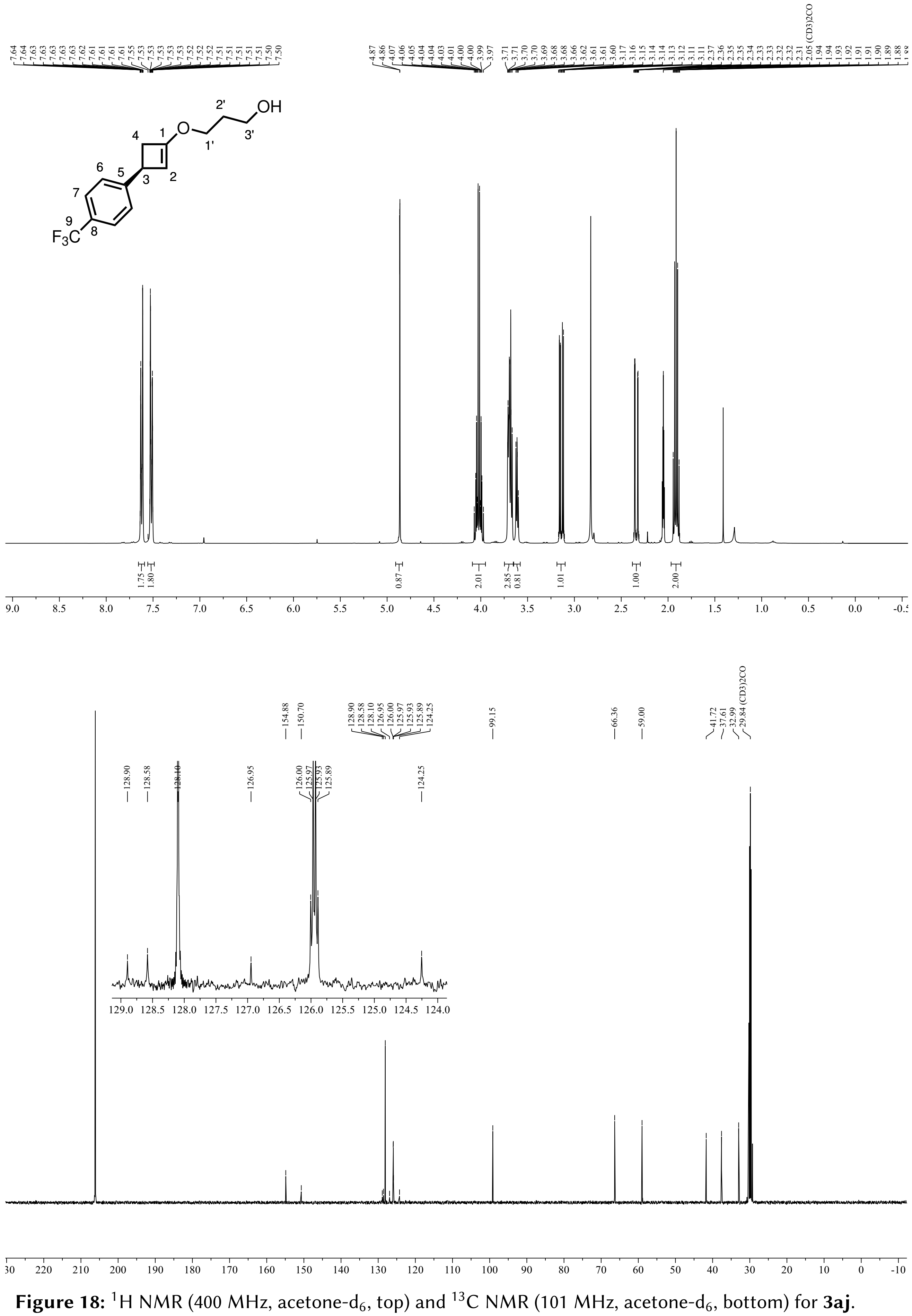

Figure 18: ^{1}H NMR (400 MHz, acetone-d_6, top) and ^{13}C NMR (101 MHz, acetone-d_6, bottom) for **3aj**.

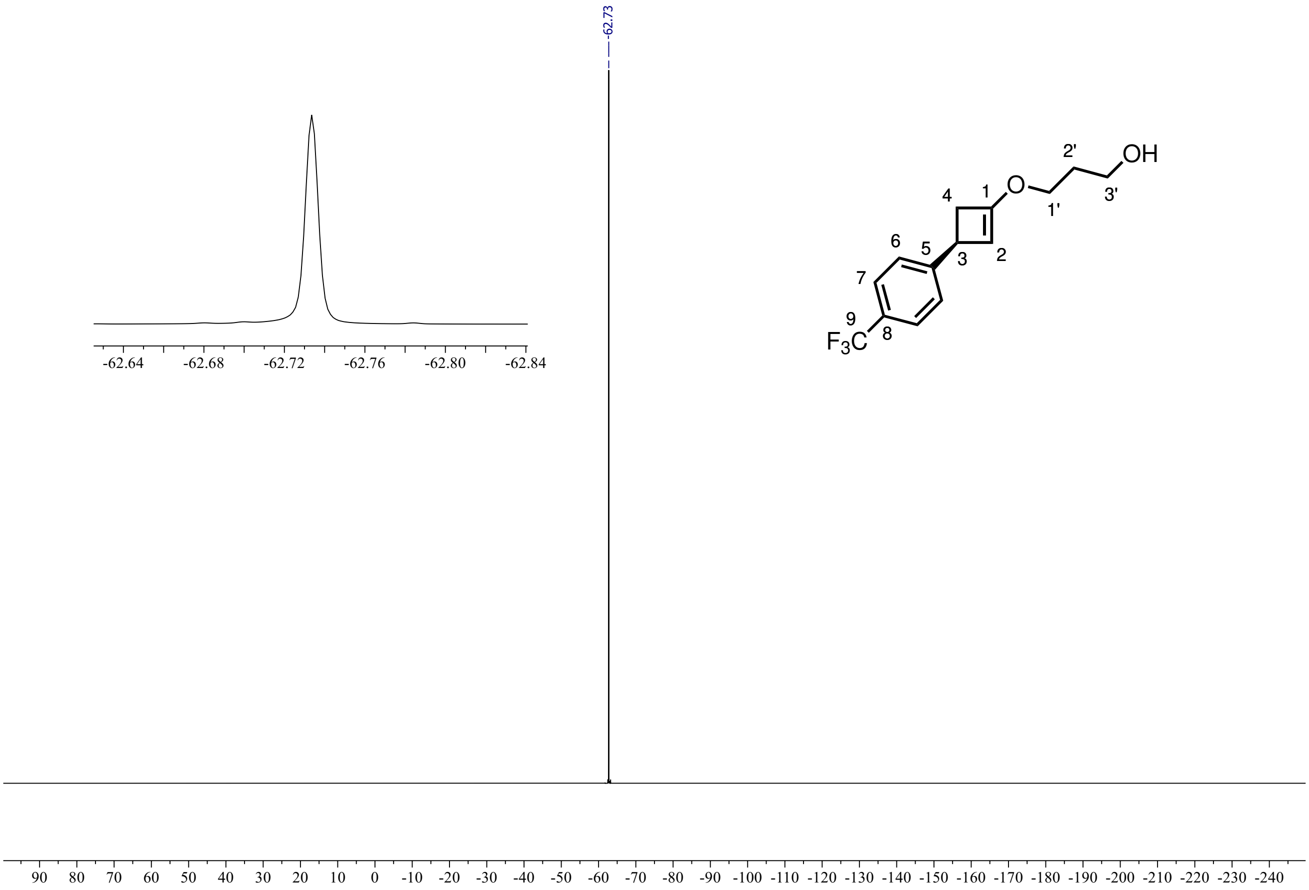

Figure 19: ^{19}F NMR (Acetone-d6, 376 MHz) for **3aj**.

Synthesis of 3ak

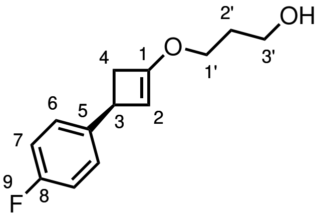

The corresponding compound was prepared following General Procedure A, using 4-fluorophenylboronic acid. Purification by manual flash chromatography (Pentane 80:20 Et_2O) afforded a colorless oil identified as **3ak** (70.6 mg, 79% yield). SFC analysis showed an enantiomeric excess of 96%.

^{1}H NMR (Acetone-d$_6$, 400 MHz): δ (ppm) 7.31 (ddd, *J* = 8.8, 5.5, 2.6 Hz, 2H; C(6)-H), 7.08 – 6.98 (m, 2H; C(7)-H), 4.82 (d, *J* = 0.9 Hz, 1H; C(2)-H), 4.07 – 3.92 (m, 2H; C(1')-H), 3.72 – 3.56 (m, 4H; C(3')-H, C(3)-H, OH), 3.08 (dd, *J* = 12.8, 4.5 Hz, 1H; C(4)-H), 2.26 (dd, *J* = 12.8, 1.5 Hz, 1H; C(4)-H), 1.90 (p, *J* = 6.2 Hz, 2H; C(2')-H).

^{13}C NMR (Acetone-d$_6$, 101 MHz): δ (ppm) 162.3 (d, *J* =241.7 Hz, C(8)), 154.6 (C(1)), 141.7 (d, *J* =3.1 Hz, C(5)), 129.0 (d, *J* =7.9 Hz, C(6)), 115.6 (d, *J* =21.2 Hz, C(7)), 99.5 (C(2)), 66.2 (C(1')), 59.0 (C(3')), 41.9 (C(4)), 37.1 (C(3)), 33.0 (C(2')).

^{19}F NMR (Acetone-d$_6$, 376 MHz): δ (ppm) -118.88 (tt, *J* = 9.0, 5.5 Hz).

IR (neat): 3334 (br), 2918 (m), 2849 (w), 1632 (s), 1600 (w), 1508 (s), 1472 (w), 1303 (s), 1213 (s), 1156 (w), 1057 (w), 1057 (m), 943 (w), 833 (m), 817 (m), 750 (w) cm^{-1}.

HRMS (ESI): m/z calculated for $C_{13}H_{16}O_2F^+$ $[M+H]^+$ = 223.1129; found = 223.1131.

SFC Chiralpak ® IF; 1500 psi, 30 °C; flow 1.5 mL/min; from 1% to 30% MeOH in 5 min; 96% ee (minor enantiomer t_R = 2.72 min; major enantiomer t_R = 2.76 min).

$[\alpha]_D^{25}$ = +33.5 (c = 0.36, CH_2Cl_2).

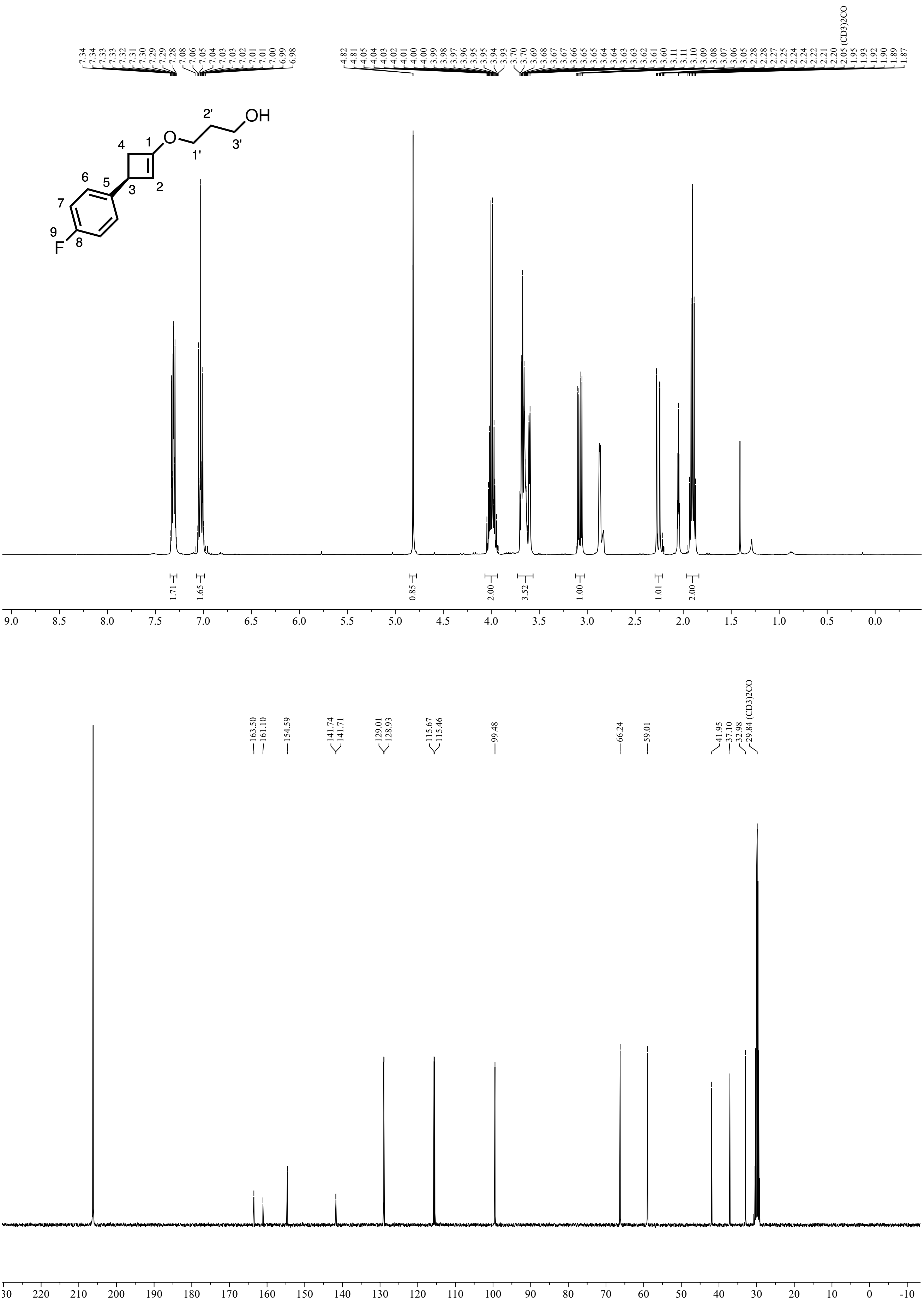

Figure 20: ^{1}H NMR (400 MHz, acetone-d_6, top) and ^{13}C NMR (101 MHz, acetone-d_6, bottom) for **3ak**.

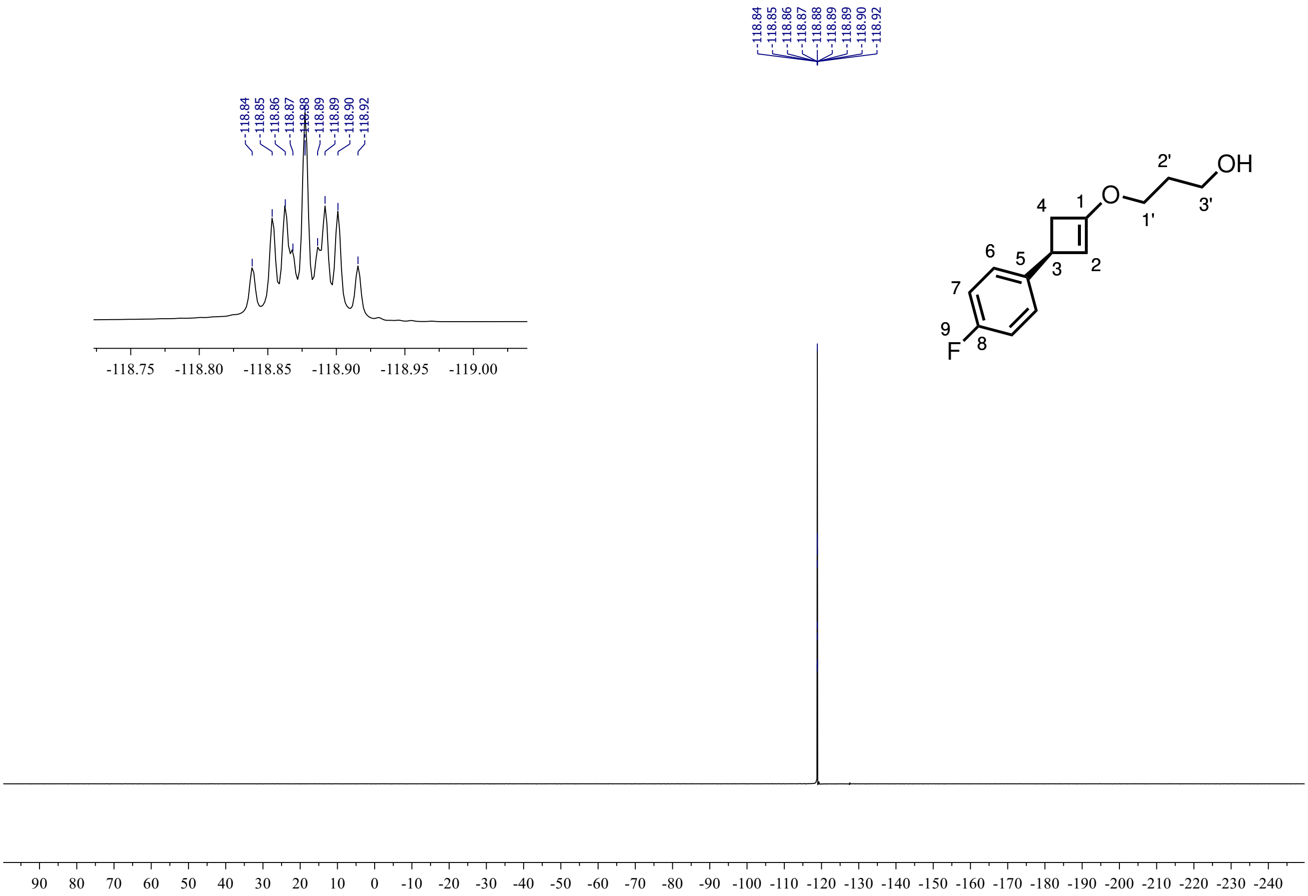

Figure 21: ^{19}F NMR (Acetone-d_6, 376 MHz) for **3ak**.

Synthesis of 3al

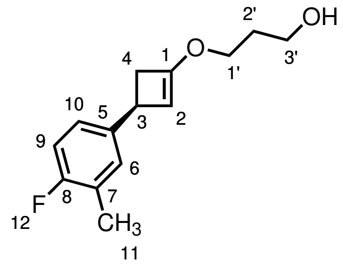

The corresponding compound was prepared following General Procedure A, using 4-Fluoro-3-methylphenylboronic acid. Purification by manual flash chromatography (Pentane 80:20 Et_2O) afforded a colorless oil identified as **3al** (76.5 mg, 81% yield). SFC analysis showed an enantiomeric excess of 95%.

^{1}H NMR (Acetone-d_6, 400 MHz): δ (ppm) 7.18 – 7.14 (m, 1H, C(Ar)-H), 7.13 – 7.08 (m, 1H, C(Ar)-H), 6.94 (dd, *J* = 9.8, 8.3 Hz, 1H, C(Ar)-H), 4.80 (d, *J* = 0.9 Hz, 1H, C(2)-H), 4.06 – 3.92 (m, 2H, C(1')-H), 3.72 – 3.59 (m, 3H, C(3')-H, C(3)-H), 3.59 – 3.52 (m, 1H, OH), 3.06 (dd, *J* = 12.8, 4.6 Hz, 1H, C(4)-H), 2.30 – 2.19 (m, 4H, C(4)-H, C(11)-H), 1.90 (p, *J* = 6.3 Hz, 2H, C(2')-H).

^{13}C NMR (Acetone-d_6, 101 MHz): δ (ppm) 160.7 (d, *J* =240.8 Hz C(8)), 154.5 (C(1)), 141.3 (d, *J* =3.3 Hz, C(5)), 130.4 (d, *J* =4.9 Hz, C(6)), 126.2 (d, *J* =7.9 Hz, C(10)), 124.8 (d, *J* =17.1 Hz, C(8)), 115.2 (d, *J* =22.2 Hz, C(9)), 99.5 (C(2)), 66.2 (C(1')), 59.0 (C(3')), 41.9 (C(4)), 37.1 (C(3)), 33.0 (C(2')), 14.5 (dd, *J* =3.6, 1.9 Hz, (C(11))).

^{19}F NMR (Acetone-d_6, 376 MHz): δ (ppm) -123.22 (dddd, *J* = 9.7, 7.5, 5.1, 2.2 Hz).

IR (neat): 3357 (br), 2926 (w), 1632 (s), 1502 (m), 1300 (s), 1245 (w), 1214 (m), 1144 (w), 1116 (m), 1057 (m), 1005 (w), 884 (w), 819 (m), 781 (m), 757 (m) cm^{-1}.

HRMS (ESI): m/z calculated for $C_{14}H_{18}O_2F^+$ $[M+H]^+$ = 237.1285; found = 237.1287.

SFC Chiralpak ® IF; 1500 psi, 30 °C; flow 1.5 mL/min; from 1% to 30% MeOH in 5 min; 95% ee (minor enantiomer t_R = 2.61 min; major enantiomer t_R = 2.71 min).

$[\alpha]_D^{25}$ = +29.4 (c = 1.09, CH_2Cl_2).

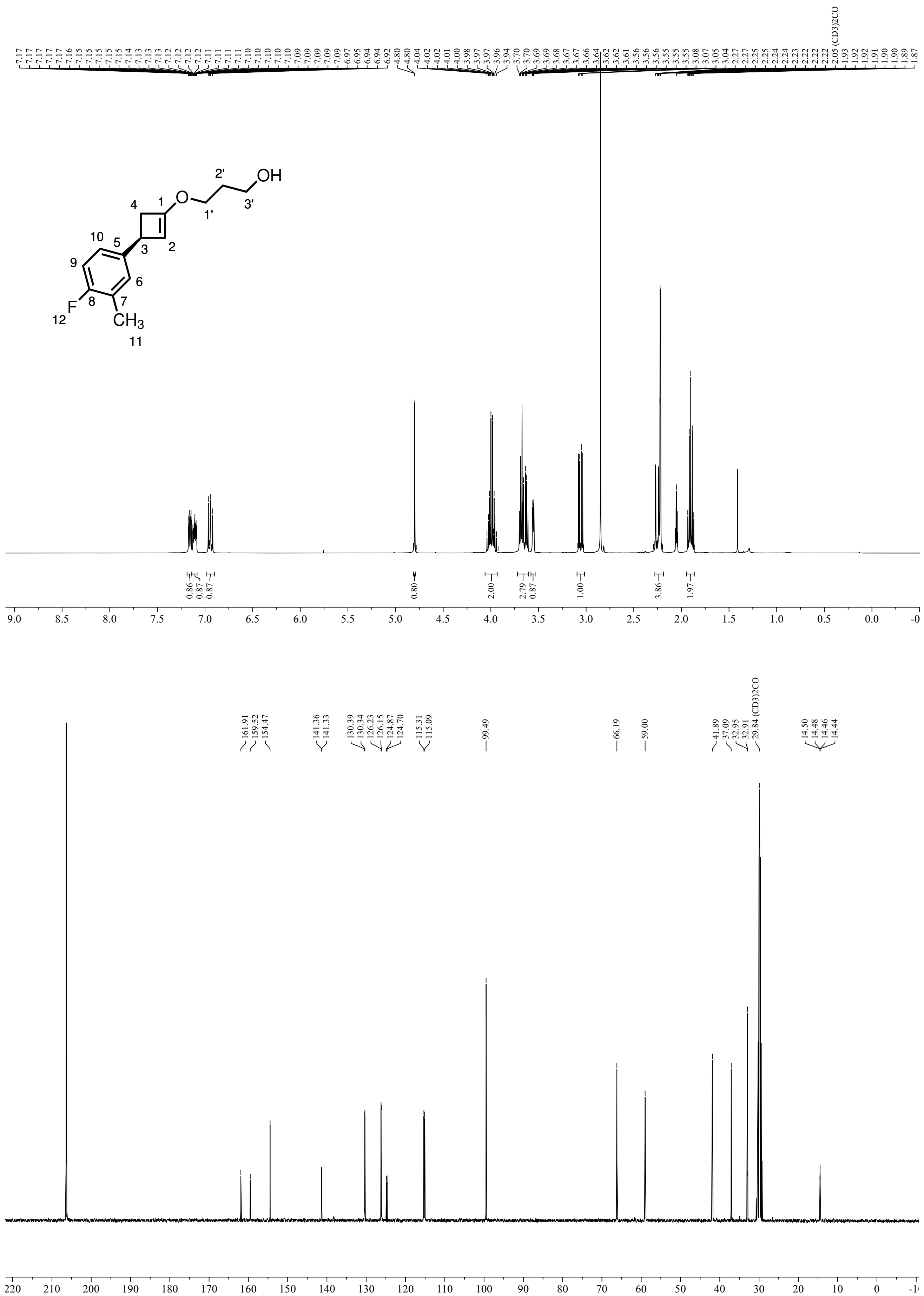

Figure 22: ^{1}H NMR (400 MHz, acetone-d$_6$, top) and ^{13}C NMR (101 MHz, acetone-d$_6$, bottom) for **3al**.

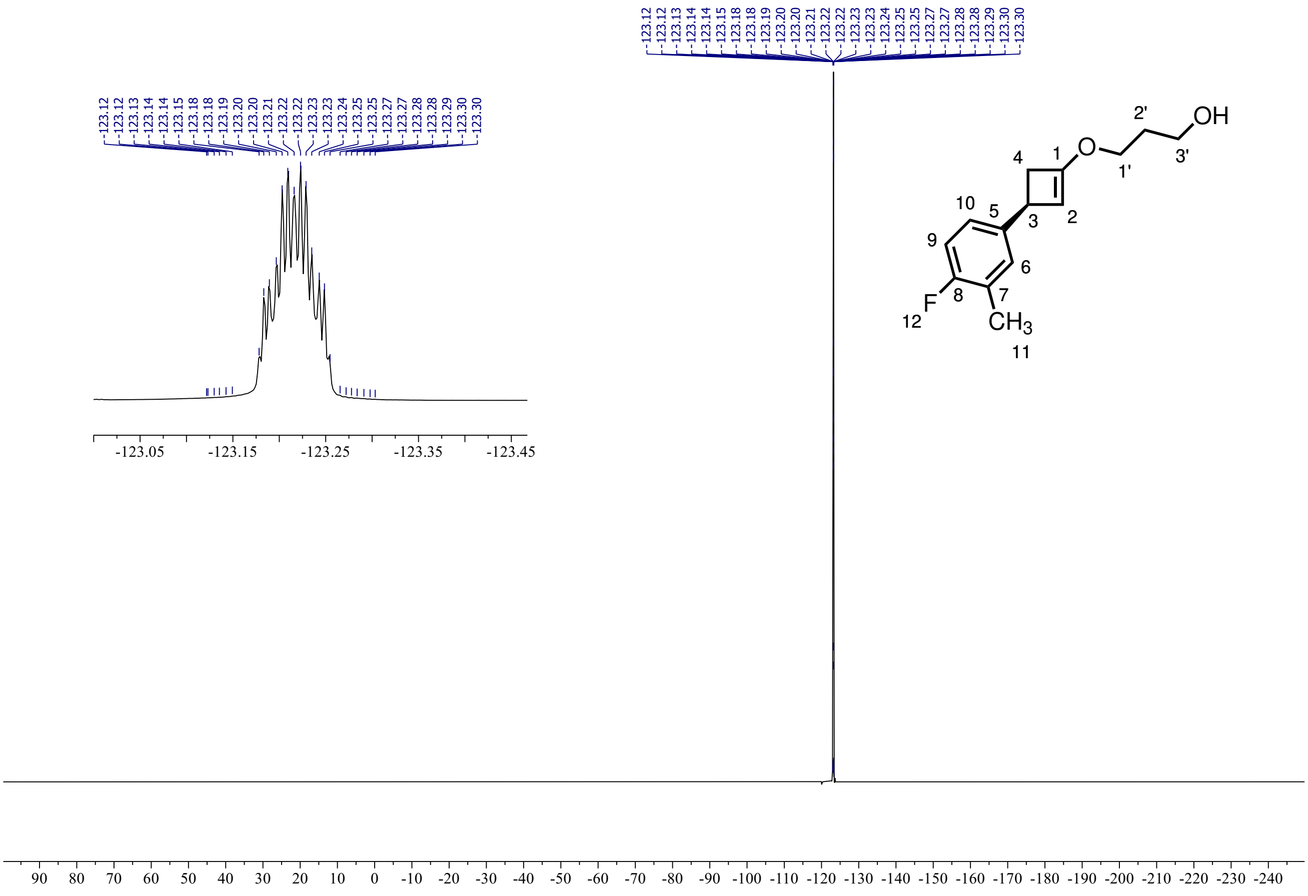

Figure 23: ^{19}F NMR (Acetone-d_6, 376 MHz) for **3al**.

Synthesis of 3am

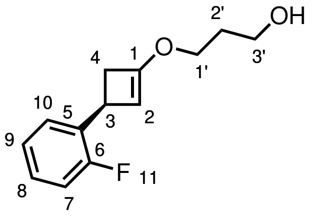

The corresponding compound was prepared following General Procedure A, using 2-fluorophenylboronic acid. Purification by manual flash chromatography (Pentane 80:20 Et_2O) afforded a colorless oil identified as **3am** (47.1 mg, 53% yield). SFC analysis showed an enantiomeric excess of 93%.

^{1}H NMR (Acetone-d$_6$, 400 MHz): δ (ppm) 7.38 (tdd, *J* = 7.8, 1.9, 0.7 Hz, 1H, C(Ar)-H), 7.23 (dddd, *J* = 8.2, 7.3, 5.3, 1.9 Hz, 1H, C(Ar)-H), 7.12 (td, *J* = 7.5, 1.3 Hz, 1H, C(Ar)-H), 7.04 (ddd, *J* = 10.5, 8.1, 1.2 Hz, 1H, C(Ar)-H), 4.83 (d, *J* = 0.9 Hz, 1H, C(2)-H), 4.08 – 3.94 (m, 2H, C(1')-H), 3.82 (d, *J* = 4.6 Hz, 1H, C(3)-H), 3.68 (td, *J* = 6.1, 4.5 Hz, 3H, C(3')-H, OH), 3.12 (dd, *J* = 12.9, 4.6 Hz, 1H, C(4)-H), 2.34 (ddd, *J* = 12.8, 1.6, 0.5 Hz, 1H, C(4)-H), 1.90 (p, *J* = 6.2 Hz, 2H, C(2')-H).

^{13}C NMR (Acetone-d$_6$, 101 MHz): δ 162.0 (d, *J* =243.3 Hz, C(6)), 154.3 (C(1)), 132.3 (d, *J* =15.0 Hz, (C(5))), 128.8 – 128.6 (m, C(8), C(9)), 124.9 (d, *J* =3.6 Hz, C(10)), 115.5 (d, *J* =22.1 Hz, C(7)), 97.4 (C(2)), 66.3 (C(1')), 59.0 (C(3')), 40.7 (C(4)), 33.0 (C(2')), 31.1 (d, *J* =3.7 Hz, (C(3))).

^{19}F NMR (Acetone-d$_6$, 376 MHz): δ (ppm) (ppm) -120.82 (ddd, *J* = 10.6, 7.9, 5.2 Hz).

IR (neat): 3341 (br), 2928 (w), 1632 (s), 1581 (w), 1488 (m), 1453 (w), 1304 (s), 1233 (m), 1209 (m), 1057 (m), 1005 (w), 944 (w), 818 (w), 755 (s) cm^{-1}.

HRMS (ESI): m/z calculated for $C_{13}H_{16}O_2F^+$ $[M+H]^+$ = 223.1129; found = 223.1130.

SFC Chiralpak ® IF; 1500 psi, 30 °C; flow 1.5 mL/min; from 1% to 30% MeOH in 5 min; 93% ee (minor enantiomer t_R = 2.81 min; major enantiomer t_R = 2.93 min).

$[\alpha]_D^{25}$ = +68.6 (c = 1.04, CH_2Cl_2).

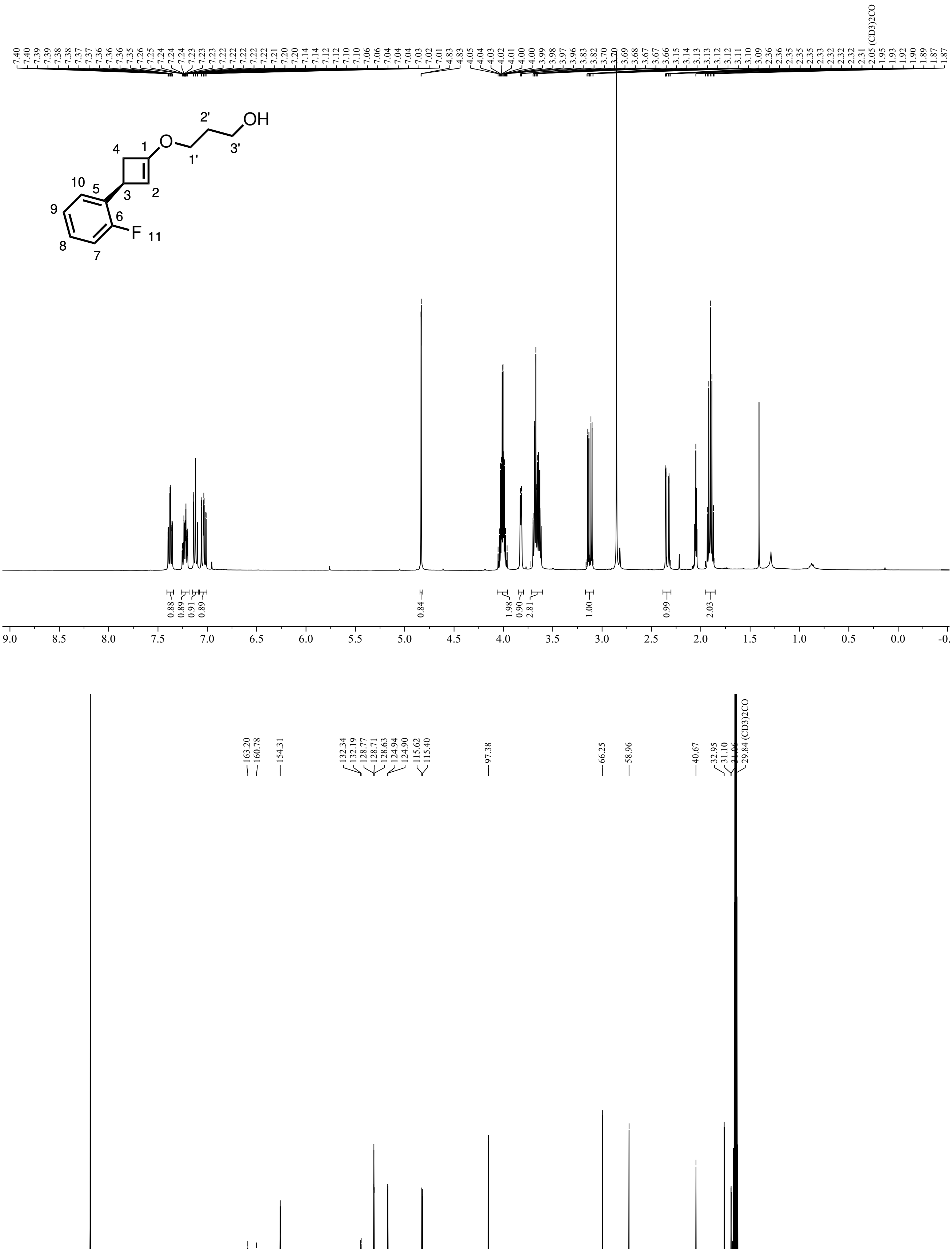

Figure 24: ^{1}H NMR (400 MHz, acetone-d_6, top) and ^{13}C NMR (101 MHz, acetone-d_6, bottom) for **3am**.

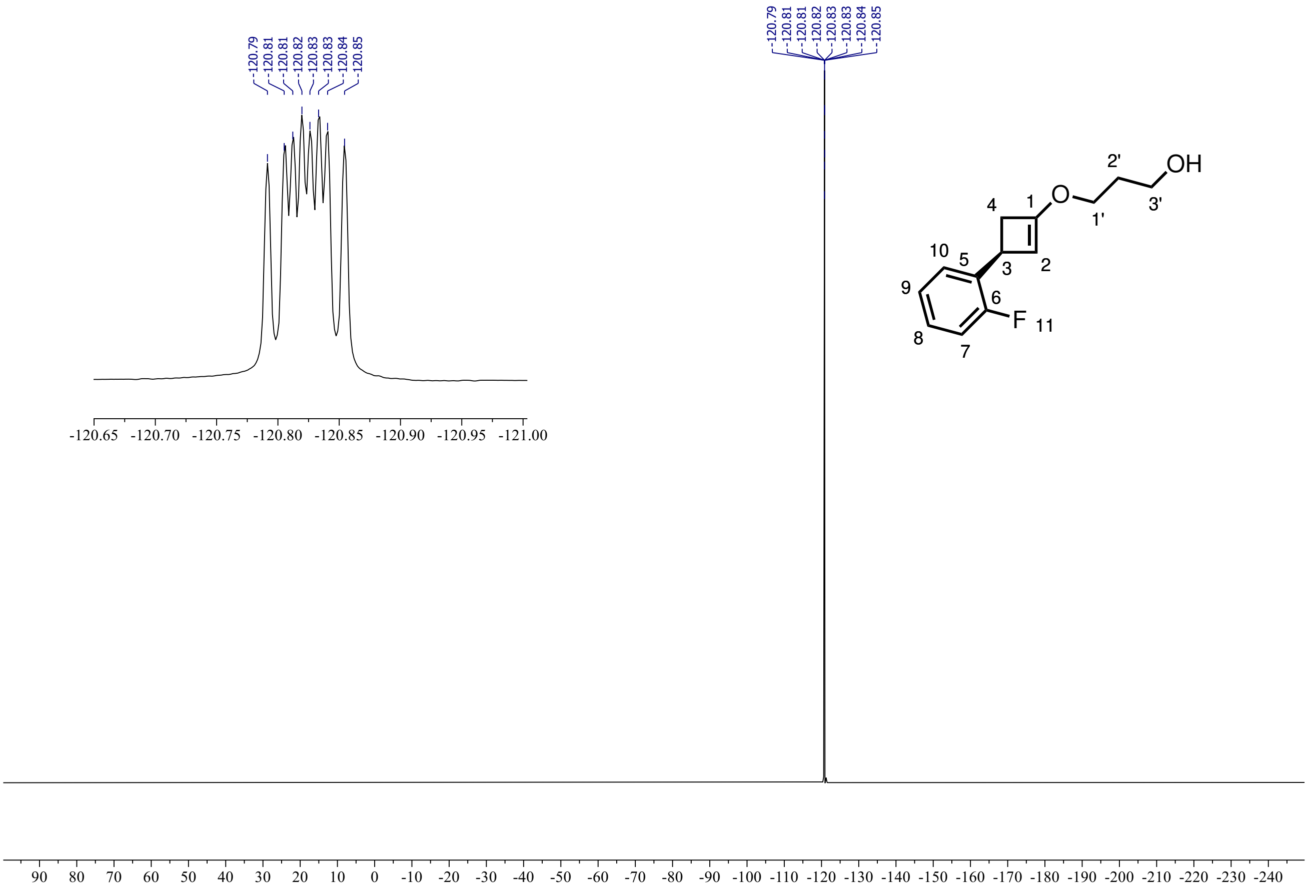

Figure 25: ^{19}F NMR (Acetone-d_6, 376 MHz) for **3am**.

Synthesis of 3an

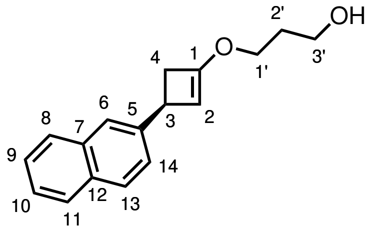

The corresponding compound was prepared following General Procedure A, using 2-naphthylboronic acid. Purification by manual flash chromatography (Pentane 80:20 Et_2O) afforded a colorless oil identified as **3an** (93.8 mg, 92% yield). SFC analysis showed an enantiomeric excess of 96%.

^{1}H NMR (Acetone-d_6, 400 MHz): δ (ppm) 7.88 – 7.79 (m, 3H, C(Ar)-H), 7.79 – 7.74 (m, 1H, C(Ar)-H), 7.50 – 7.38 (m, 3H, C(Ar)-H), 4.93 (d, *J* = 0.9 Hz, 1H, C(2)-H), 4.04 (qt, *J* = 10.0, 6.4 Hz, 2H, C(1')-H), 3.81 – 3.62 (m, 4H, C(3')-H, C(3)-H, OH), 3.15 (dd, *J* = 12.8, 4.6 Hz, 1H, C(4)-H), 2.39 (dd, *J* = 12.8, 1.6 Hz, 1H, C(4)-H), 1.93 (p, *J* = 6.3 Hz, 2H, C(2')-H).

^{13}C NMR (Acetone-d_6, 101 MHz): δ (ppm) 154.6 (C(1)), 143.4 (C(Ar)), 134.6 (C(Ar)), 133.4 (C(Ar)), 128.7 (C(Ar)), 128.4 (C(Ar)), 128.3 (C(Ar)), 126.7 (C(Ar)), 126.1 (C(Ar)), 126.0 (C(Ar)), 125.5 (C(Ar)), 99.6 (C(2)), 66.3 (C(1')), 59.0 (C(3')), 41.7 (C(4)), 38.0 (C(3)), 33.0 (C(2')).

IR (neat): 3343 (br), 2920 (w), 1630 (s), 1506 (w), 1469 (w), 1302 (m), 1217 (m0, 1057 (m), 956 (w), 818 (m), 779 (m) cm^{-1}.

HRMS (ESI): m/z calculated for $C_{17}H_{19}O_2^+$ $[M+H]^+$ = 255.1380; found = 255.1381.

SFC Chiralpak ® IF; 1500 psi, 30 °C; flow 1.5 mL/min; from 1% to 30% MeOH in 5 min; 96% ee (minor enantiomer t_R = 4.24 min; major enantiomer t_R = 4.47 min).

$[\alpha]_D^{25}$ = +15.9 (c = 1.00, CH_2Cl_2).

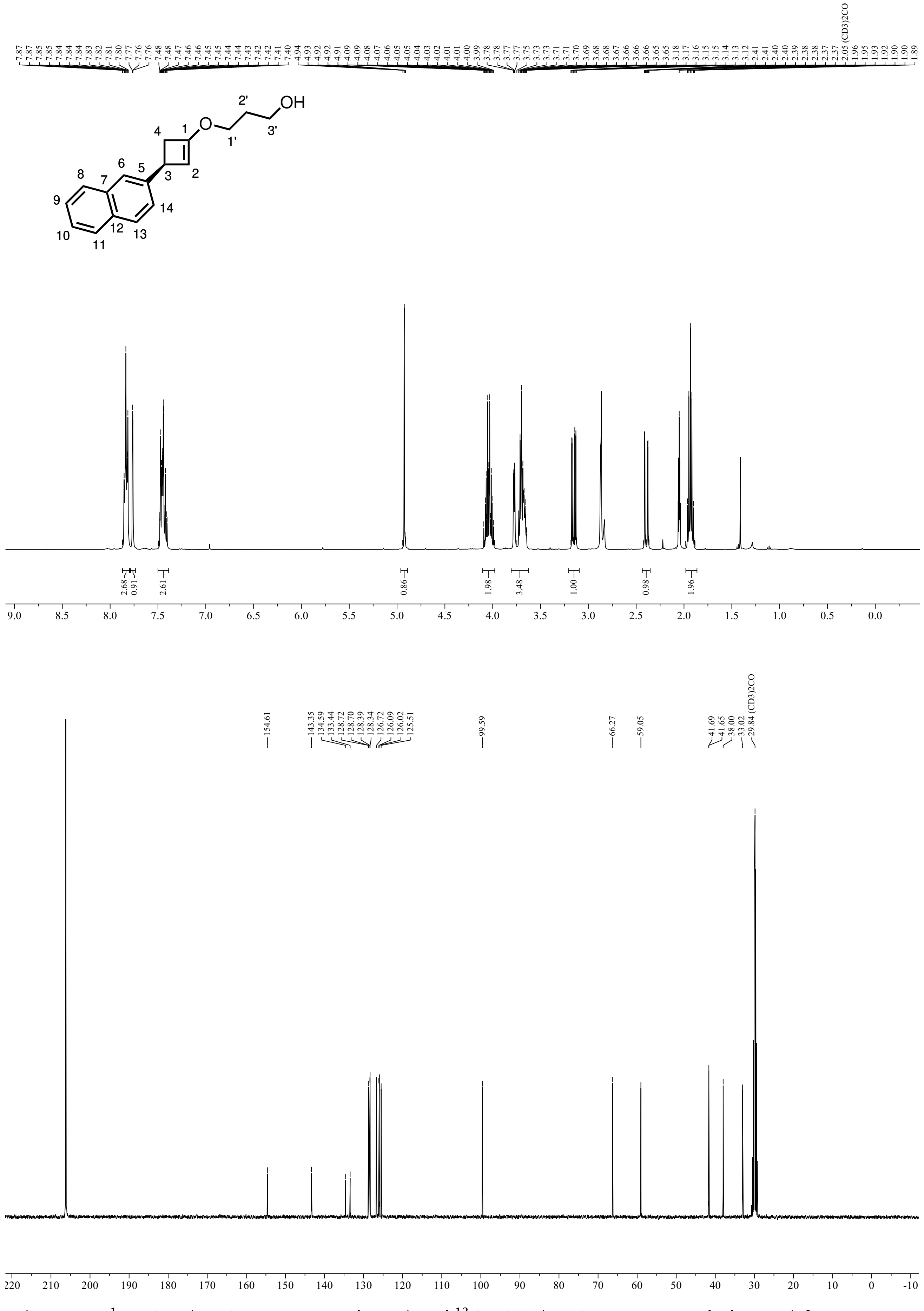

Figure 26: ^{1}H NMR (400 MHz, acetone-d$_6$, top) and ^{13}C NMR (101 MHz, acetone-d$_6$, bottom) for **3an**.

Synthesis of 3ao

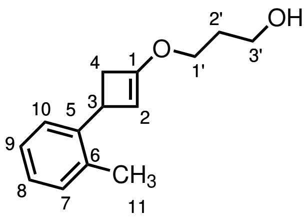

The corresponding compound was prepared following General Procedure B, using *o*-tolylboronic acid. Purification by manual flash chromatography (Pentane 80:20 Et_2O) afforded a colorless oil identified as **3ao** (42.5 mg, 49% yield). SFC analysis showed an enantiomeric excess of 70%.

^{1}H NMR (Acetone-d_6, 400 MHz): δ (ppm) 7.29 (dd, *J* = 7.8, 1.9 Hz, 1H, C(Ar)-H), 7.18 – 7.03 (m, 3H, C(Ar)-H), 4.90 (d, *J* = 0.9 Hz, 1H, C(2)-H), 4.08 – 3.94 (m, 2H, C(1')-H), 3.76 – 3.64 (m, 3H, C(3')-H, OH), 3.61 (dd, *J* = 5.6, 4.7 Hz, 1H, C(3)-H), 3.14 (dd, *J* = 12.5, 4.6 Hz, 1H, C(4)-H), 2.28 (s, 3H, C(11)-H), 2.17 (dd, *J* = 12.5, 1.7 Hz, 1H, C(4)-H), 1.91 (p, *J* = 6.3 Hz, 2H, C(2')-H).

^{13}C NMR (Acetone-d_6, 101 MHz): δ 153.7 (C(1)), 143.4 (C(5)), 136.8 (C(5)), 130.4 (C(10)), 126.8 (C(7)), 126.5 (C(8)), 126.3 (C(9)), 97.7 (C(2)), 66.2 (C(1')), 59.0 (C(2')), 40.5 (C(4)), 35.2 (C(3)), 33.0 (C(2')), 19.0 (C(11)).

IR (neat): 3359 (br), 2921 (w), 1632 (w), 1461 (w), 1380 (w), 1302 (s), 1228 (w), 1205 (w), 1057 (m), 1005 (w), 947 (w), 790 (w), 753 (s), 726 (w) cm^{-1}.

HRMS (ESI): m/z calculated for $_{14}H_{19}O_2^+$ $[M+H]^+$ = 219.1380; found = 219.1381.

SFC Chiralpak ® IF; 1500 psi, 30 °C; flow 1.5 mL/min; from 1% to 30% MeOH in 5 min; 70% ee (minor enantiomer t_R = 3.45 min; major enantiomer t_R = 3.12 min).

$[\alpha]_D^{25}$ = -105.8 (c = 0.71, CH_2Cl_2).

Note: Opposite sign for $[\alpha]_D^{25}$ was observed when using **L4** instead of **L1**. Absolute configuration hance not assigned.

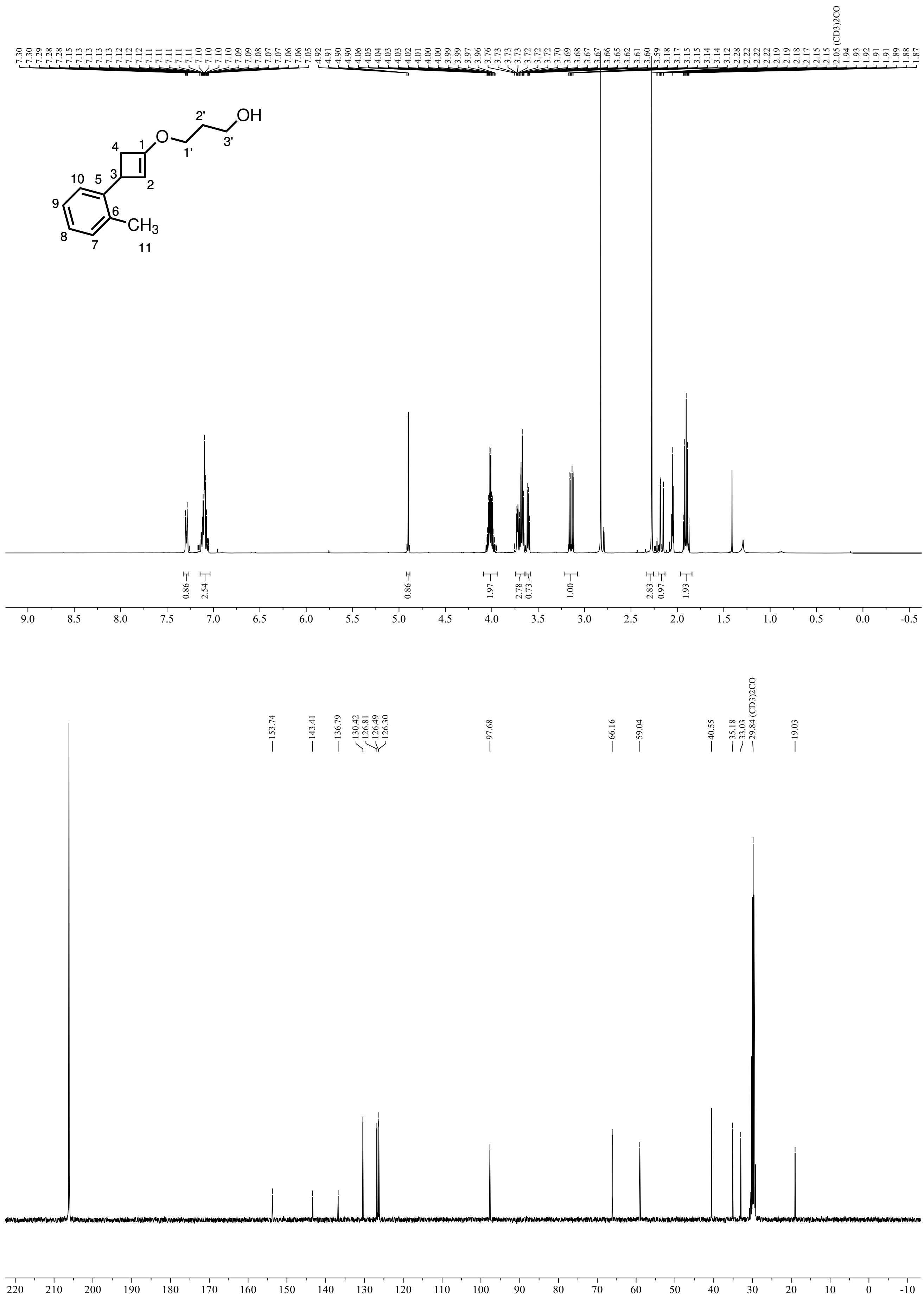

Figure 27: ^{1}H NMR (400 MHz, acetone-d$_6$, top) and ^{13}C NMR (101 MHz, acetone-d$_6$, bottom) for **3ao**.

Synthesis of 3ap

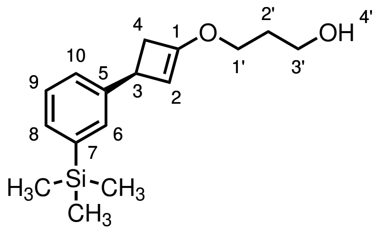

The corresponding compound was prepared following General Procedure A, using 3-(Trimethylsilyl)phenylboronic acid. Purification by manual flash chromatography (Pentane 80:20 Et_2O) afforded a colorless oil identified as **3ap** (96.2 mg, 87% yield). SFC analysis showed an enantiomeric excess of 92%.

^{1}H NMR (Acetone-d_6, 400 MHz): δ (ppm) 7.50 – 7.44 (m, 1H, C(Ar)-H), 7.39 – 7.24 (m, 3H, C(Ar)-H), 4.84 (d, J = 1.0 Hz, 1H, C(2)-H), 4.07 – 3.93 (m, 2H, C(1')-H), 3.73 – 3.58 (m, 4H, C(3')-H, C(3')-H, OH), 3.08 (dd, J = 12.8, 4.6 Hz, 1H, C(4)-H), 2.30 (dd, J = 12.8, 1.6 Hz, 1H, C(4)-H), 1.91 (p, J = 6.3 Hz, 2H, C(2')-H), 0.25 (s, 9H, C(TMS)-H).

^{13}C NMR (Acetone-d_6, 101 MHz): δ (ppm) 154.4 (C(1)), 144.7 (C(5)), 140.6 (C(7)), 132.4 (C(6)), 131.9 (C(10)), 128.5 (C(9)), 127.7 (C(8)), 99.6 (C(2)), 66.1 (C(1')), 59.0 (C(3')), 41.8 (C(4)), 37.9 (C(3)), 33.0 (C(2')), -1.0 (C(TMS)).

IR (neat): 3347 (br), 2955 (w), 1632 (s), 1474 (w), 1406 (w), 1311 (m), 1295 (m), 1248 (m), 1216 (w), 1117 (w), 1059 (m), 1007 (w), 852 (s), 859 (s), 791 (w), 753 (s), 705 (w), 620 (w) cm^{-1}.

HRMS (ESI): m/z calculated for $C_{16}H_{25}O_2Si^+$ $[M+H]^+$ = 277.1618; found = 277.1617.

SFC Chiralpak ® IG; 1500 psi, 30 °C; flow 0.5 mL/min; from 0% to 5% MeOH in 4 min, then constant 5% MeOH over 9 minutes; 92% ee (minor enantiomer t_R = 9.18 min; major enantiomer t_R = 8.71 min).

$[\alpha]_D^{25}$ = +42.5 (c = 0.84, CH_2Cl_2).

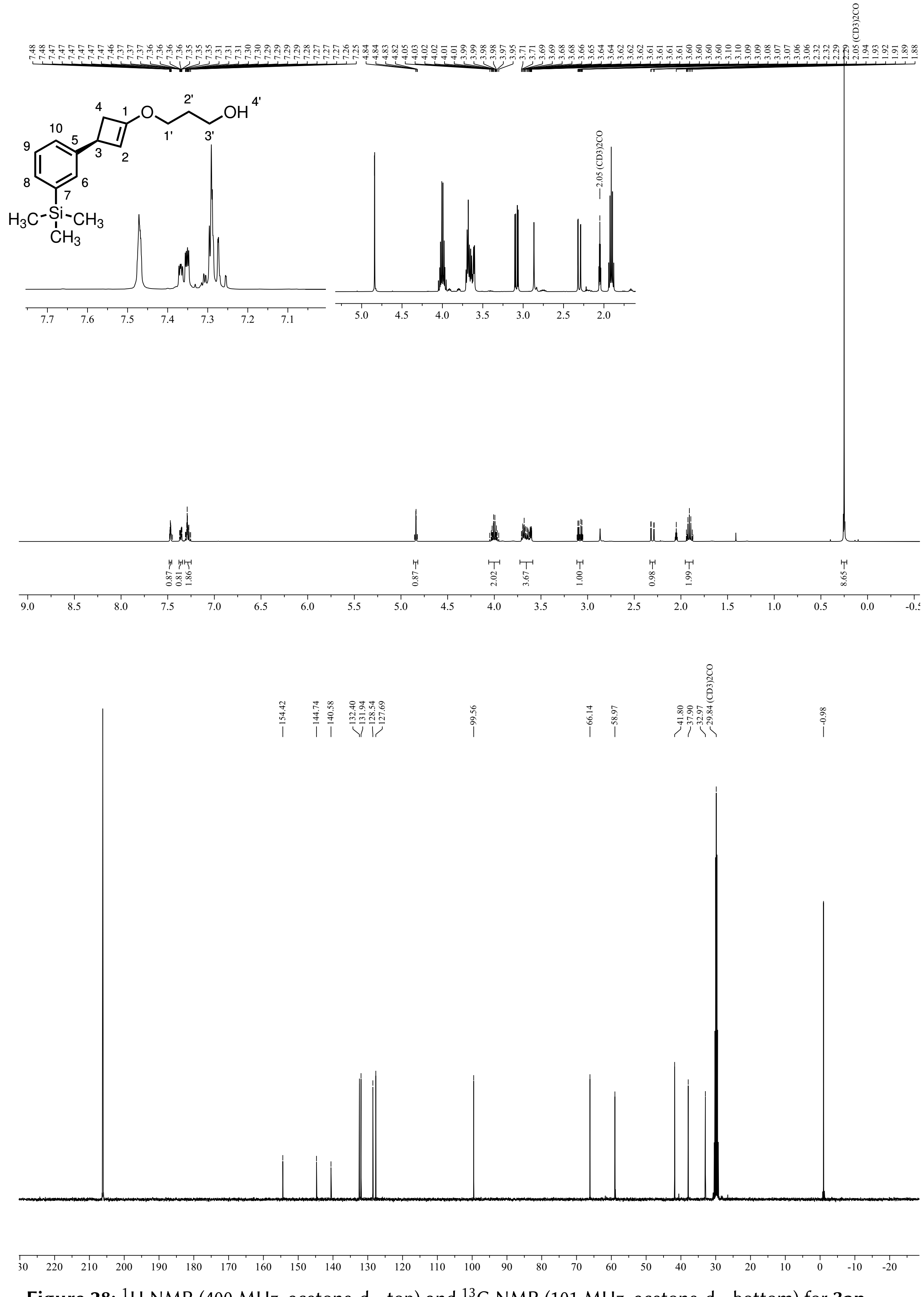

Figure 28: ^{1}H NMR (400 MHz, acetone-d_6, top) and ^{13}C NMR (101 MHz, acetone-d_6, bottom) for **3ap**.

Synthesis of 3aq

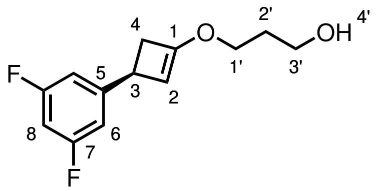

The corresponding compound was prepared following General Procedure A, using 3,5-Difluoro-phenylboronic acid. Purification by manual flash chromatography (Pentane 80:20 Et_2O) afforded a colorless oil identified as **3aq** (62.5 mg, 65% yield). SFC analysis showed an enantiomeric excess of 90%.

^{1}H NMR (Acetone-d_6, 400 MHz): δ (ppm) 6.98 – 6.88 (m, 2H), 6.80 (tt, J = 9.2, 2.4 Hz, 1H), 4.82 (d, J = 0.9 Hz, 1H), 4.01 (qt, J = 10.0, 6.4 Hz, 2H), 3.74 – 3.61 (m, 3H), 3.10 (dd, J = 12.9, 4.6 Hz, 1H), 2.31 (dd, J = 12.9, 1.6 Hz, 1H), 1.91 (p, J = 6.3 Hz, 2H).

^{13}C NMR (Acetone-d_6, 101 MHz): δ (ppm) 164.0 (dd, J = 246.1, 13.1 Hz, C(7)), 155.0 (C(1)), 151.0 (t, J = 8.6 Hz, C(5)), 110.4 – 109.9 (m, C(6)), 102.0 (t, J = 26.0 Hz, C(8)), 98.7 (C(2)), 66.3 (C(1')), 58.9 (C(3')), 41.6 (C(4)), 37.5 (C(3)), 32.9 (C(2)).

^{19}F NMR (Acetone-d_6, 376 MHz): δ (ppm) -112.12 (t, J = 8.8 Hz).

IR (neat): 3340 (br), 2930 (w), 1362 (s), 1595 (w), 1458 (w), 1305 (s), 1221 (w), 1207 (w), 1116 (m), 1058 (m), 959 (m), 859 (w), 842 (w), 773 (w), 688 (w) cm^{-1}.

HRMS (ESI): m/z calculated for $C_{13}H_{15}F_2O_2^+$ $[M+H]^+$ = 241.1035; found = 241.1037.

SFC Chiralpak ® IF; 1500 psi, 30 °C; flow 1.5 mL/min; from 1% to 30% MeOH in 5 min; 90% ee (minor enantiomer t_R = 2.49 min; major enantiomer t_R = 2.61 min).

$[\alpha]_D^{25}$ = +36.2 (c = 0.59, CH_2Cl_2).

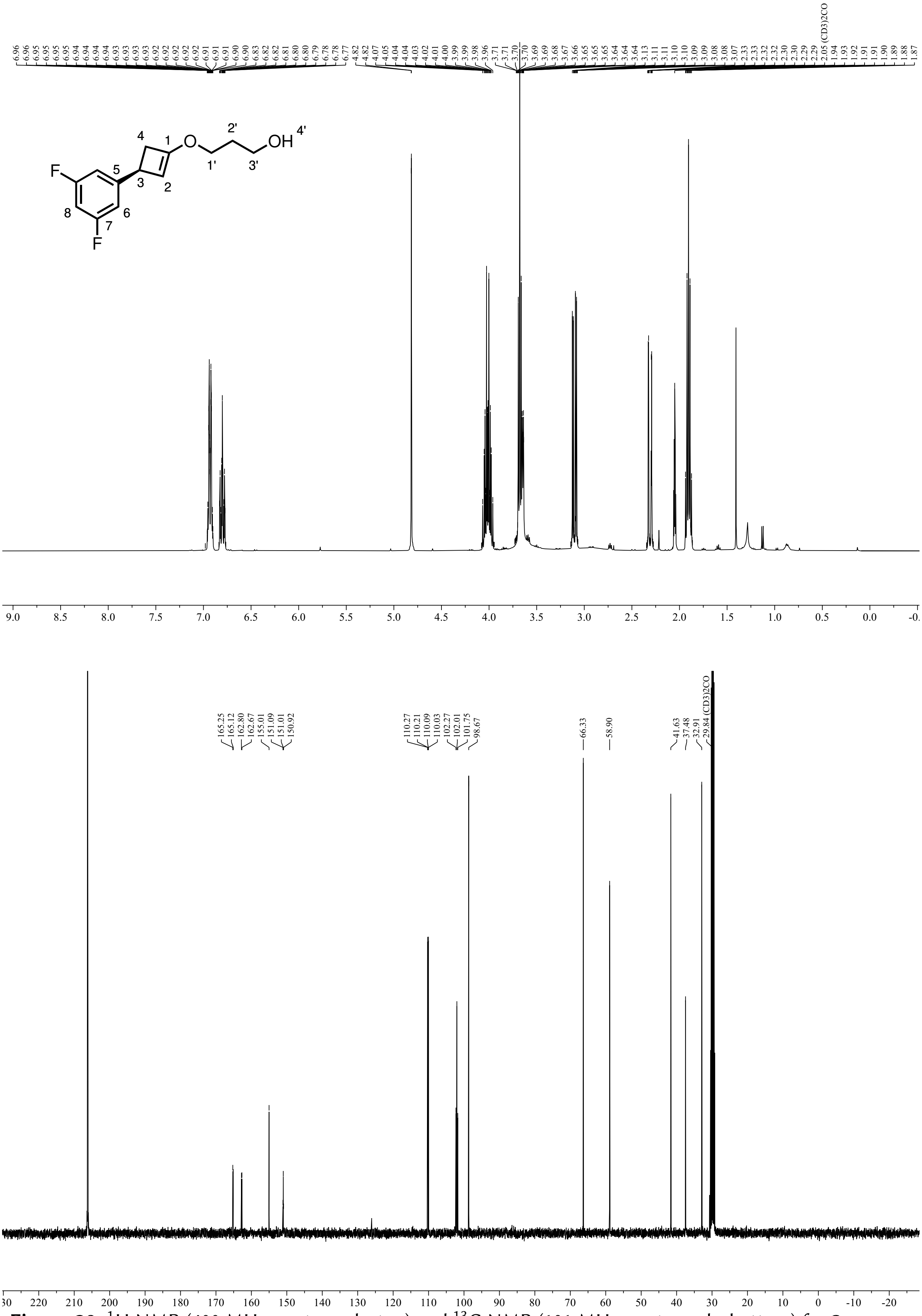

Figure 29: ^{1}H NMR (400 MHz, acetone-d$_6$, top) and ^{13}C NMR (101 MHz, acetone-d$_6$, bottom) for **3aq**.

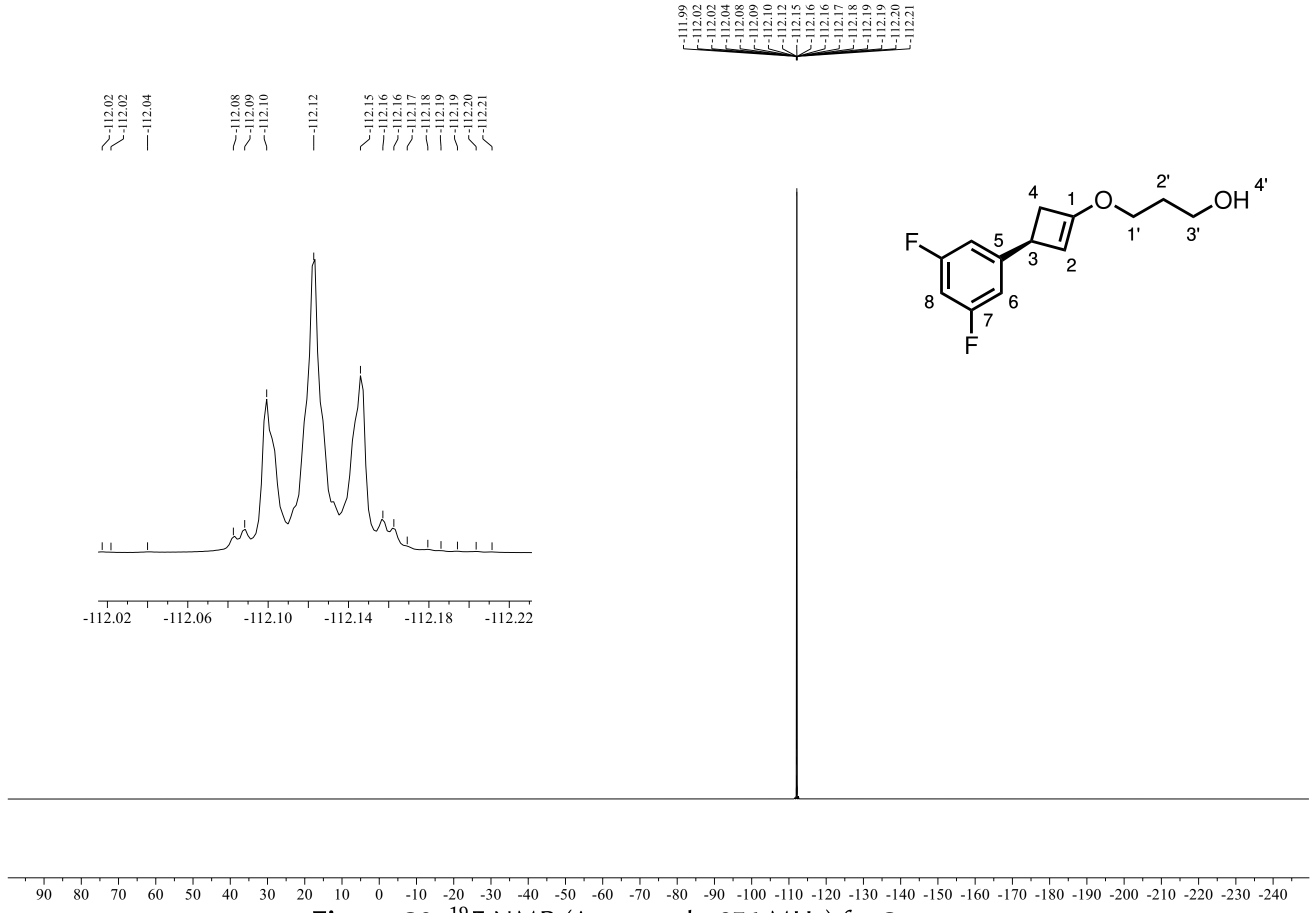

Figure 30: ^{19}F NMR (Acetone-d$_6$, 376 MHz) for **3am**.

Synthesis of 3ar

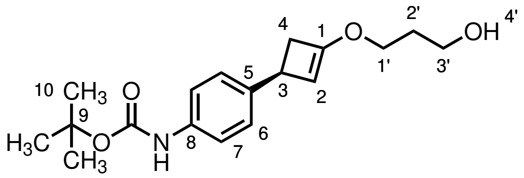

The corresponding compound was prepared following General Procedure A, using (4-Boc-Amino-phenyl)boronic Acid. Purification by manual flash chromatography (Pentane 80:20 Et_2O) afforded a colorless oil identified as **3ar** (106.0 mg, 83% yield). SFC analysis showed an enantiomeric excess of 95%.

^{1}H NMR (Acetone-d_6, 400 MHz): δ (ppm) 8.3 (s, 1H, NH), 7.5 – 7.4 (m, 2H, C(Ar)-H), 7.2 – 7.1 (m, 2H, C(Ar)-H), 4.8 (d, J = 0.9 Hz, 1H, C(2)-H), 4.1 – 3.9 (m, 2H, C(1')-H), 3.7 – 3.6 (m, 3H, C(3')-H, OH), 3.6 – 3.5 (m, 1H, C(3)-H), 3.0 (dd, J = 12.7, 4.6 Hz, 1H, C(4)-H), 2.3 (dd, J = 12.7, 1.5 Hz, 1H, C(4)-H), 1.9 (p, J = 6.3 Hz, 2H, C(2')-H), 1.5 (s, 9H, C(10)-H).

^{13}C NMR (Acetone-d_6, 101 MHz): δ (ppm) 154.4 (C(1)), 153.8 (C=O)), 139.5 (C(5)), 138.7 (C(8)), 127.5 (C(6)), 119.0 (C(7)), 99.6 (C(2)), 79.7 (C(9)), 66.1 (C(1')), 59.0 (C(3')), 41.9 (C(4)), 37.3 (C(3)), 33.0 (C(2')), 28.5 (C(10)).

IR (neat): 3326 (b), 2978 (w), 1699 (m), 1632 (m), 1595 (m), 1523 (w), 1412 (w), 1393 (m), 1367 (m), 1304 (m), 1242 (m), 1160 (s), 1056 (m), 833 (w), 786 (w) cm^{-1}.

HRMS (ESI): m/z calculated for $C_{18}H_{26}NO_4^+$ $[M+H]^+$ = 320.1856; found = 320.1857.

SFC Chiralpak ® IF; 1500 psi, 30 °C; flow 1.5 mL/min; from 1% to 50% MeOH in 10 min; 95% ee (minor enantiomer t_R = 7.18 min; major enantiomer t_R = 7.89 min).

$[\alpha]_D^{25}$ = +23.6 (c = 1.09, CH_2Cl_2).

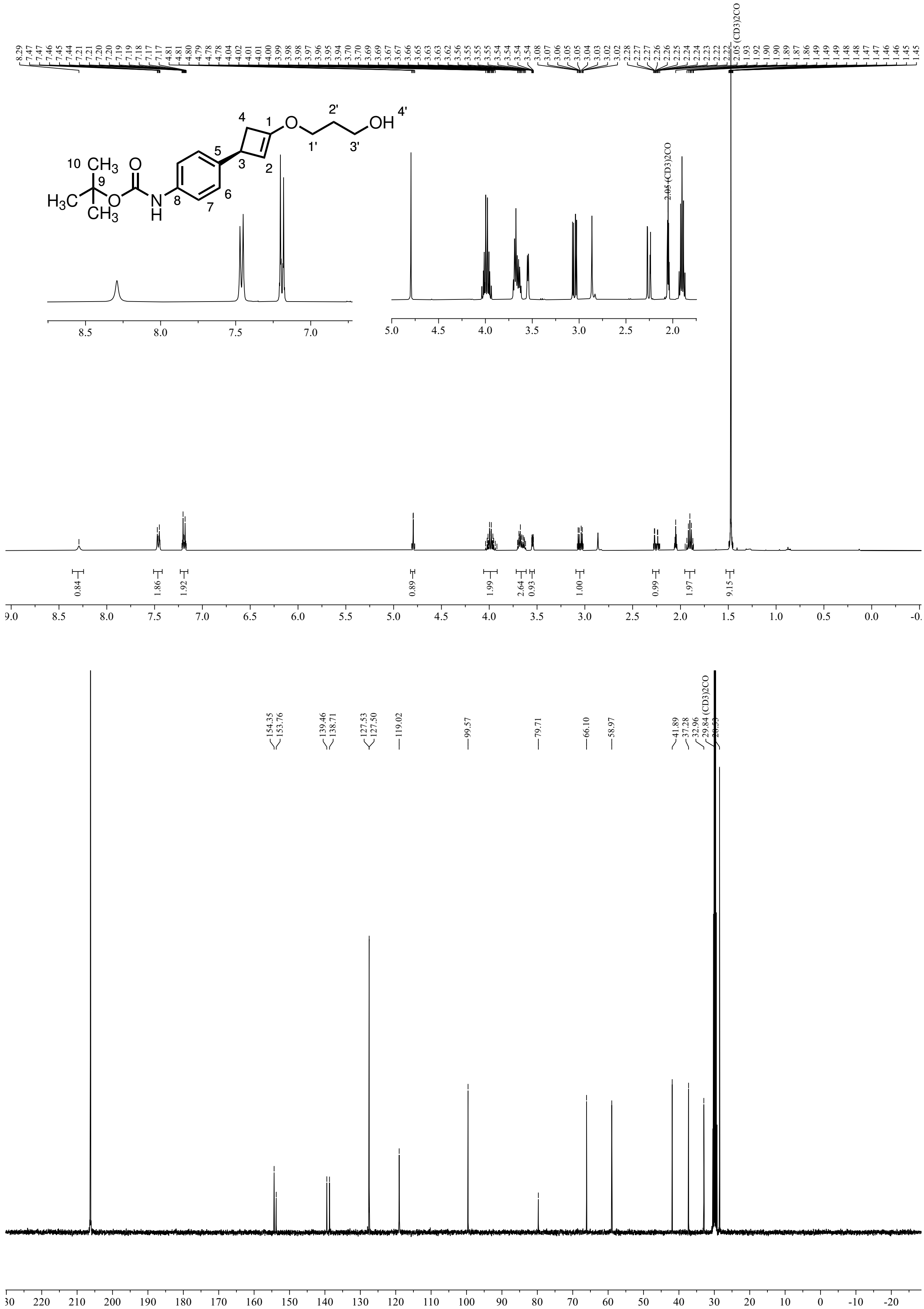

Figure 31: ^{1}H NMR (400 MHz, acetone-d_6, top) and ^{13}C NMR (101 MHz, acetone-d_6, bottom) for **3ar**.

Synthesis of 3as

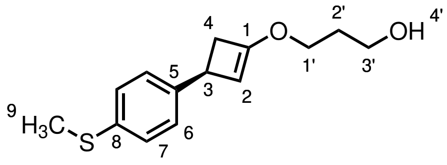

The corresponding compound was prepared following General Procedure A, using 4-(Methylthio)-phenylboronic acid. Purification by manual flash chromatography (Pentane 80:20 Et_2O) afforded a colorless oil identified as **3ar** (69.0 mg, 69% yield). SFC analysis showed an enantiomeric excess of 97%.

^{1}H NMR (Acetone-d_6, 400 MHz): δ (ppm) 7.28 – 7.17 (m, 4H, C(Ar)-H), 4.80 (d, J = 0.9 Hz, 1H, C(2)-H), 4.06 – 3.92 (m, 2H, C(1')-H), 3.72 – 3.61 (m, 3H, C(3')-H, OH), 3.57 (dd, J = 4.6, 1.4 Hz, 1H, C(3)-H), 3.07 (ddd, J = 12.7, 4.6, 0.7 Hz, 1H, C(4)-H), 2.46 (d, J = 0.7 Hz, 3H, C(9)-H), 2.26 (dt, J = 12.8, 1.2 Hz, 1H, C(4)-H), 1.90 (p, J = 6.2 Hz, 2H, C(2')-H).

^{13}C NMR (Acetone-d_6, 101 MHz): δ (ppm) 154.5 (C(1)), 142.8 (C(5)), 136.7 (C(8)), 128.0 (C(6)), 127.5 (C(7)), 99.4 (C(2)), 66.2 (C(1')), 59.0 (C(3')), 41.8 (C(4)), 37.3 (C(3)), 33.0 (C2')), 15.9 (C(9)).

IR (neat): 3389 (br), 2923 (s), 2853 (m), 1632 (m), 1494 (w), 1459 (w), 1459 (w), 1377 (m), 1304 (w), 1288 (w), 1216 (w), 1093 (w), 1057 (w), 818 (w), 789 (w), 757 (w) cm^{-1}.

HRMS (ESI): m/z calculated for $C_{14}H_{19}O_2S^+$ $[M+H]^+$ = 251.1100; found = 251.1092.

SFC Chiralpak ® IF; 1500 psi, 30 °C; flow 1.5 mL/min; from 1% to 30% MeOH in 8 min; 97% ee (minor enantiomer t_R = 5.73 min; major enantiomer t_R = 6.40 min).

$[\alpha]_D^{25}$ = +23.1 (c = 1.10, CH_2Cl_2).

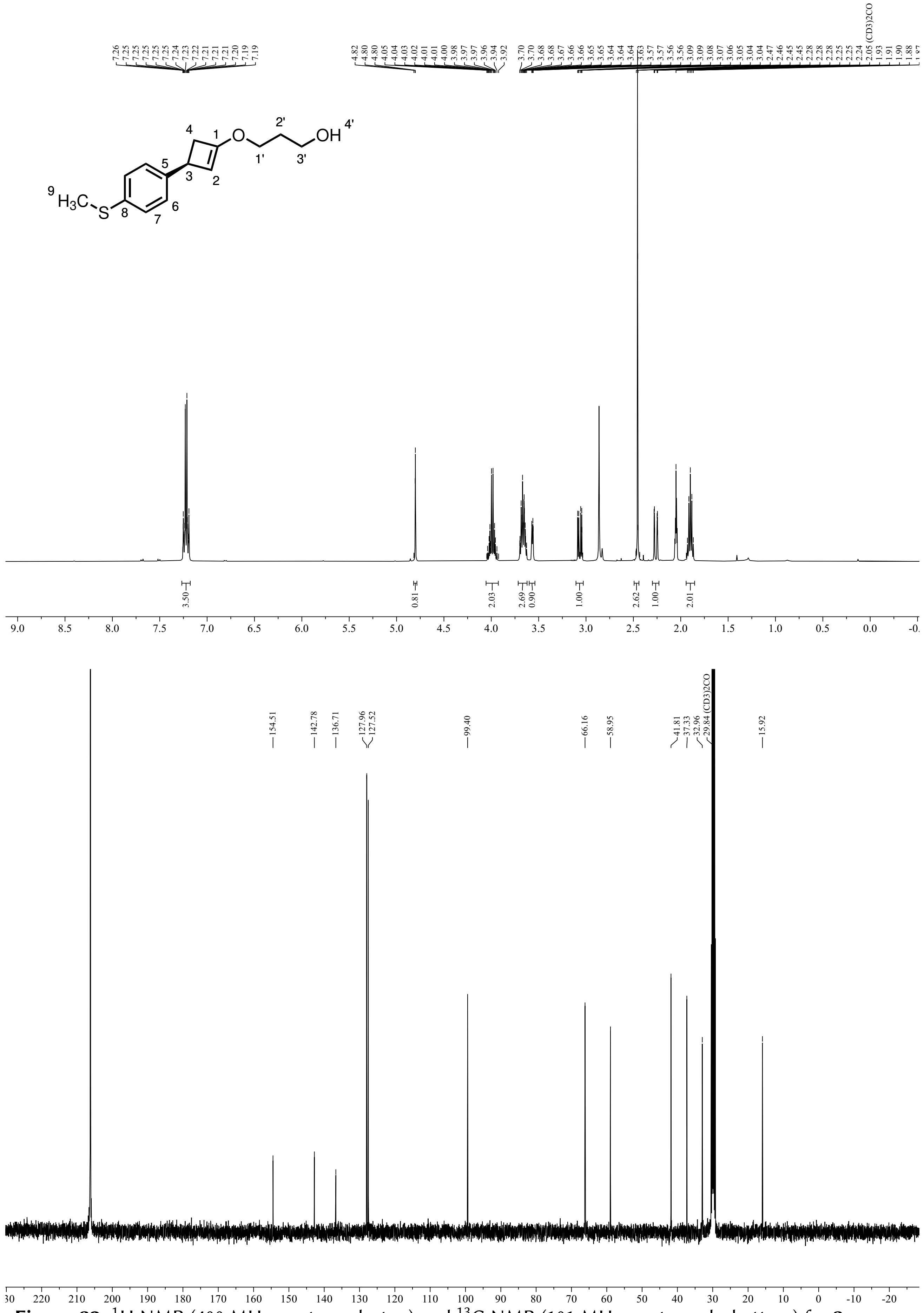

Figure 32: ^{1}H NMR (400 MHz, acetone-d_6, top) and ^{13}C NMR (101 MHz, acetone-d_6, bottom) for **3as**.

Synthesis of 3at

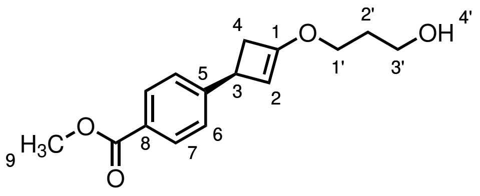

The corresponding compound was prepared following General Procedure A, using 4-Methoxy-carbonylphenylboronic acid. Purification by manual flash chromatography (Pentane 80:20 Et_2O) afforded a colorless oil identified as **3ar** (86.0 mg, 82% yield). SFC analysis showed an enantiomeric excess of 98%.

^{1}H NMR (Acetone-d$_6$, 400 MHz): δ (ppm) 7.97 – 7.88 (m, 2H, C(6)-H), 7.46 – 7.37 (m, 2H, C(7)-H), 4.85 (d, J = 0.9 Hz, 1H, C(2)-H), 4.08 – 3.94 (m, 2H, C(1')-H), 3.86 (s, 3H, C(9)-H), 3.68 (t, J = 5.0 Hz, 4H, C(3)-H, C(3')-H, OH), 3.12 (dd, J = 12.9, 4.6 Hz, 1H, C(4)-H), 2.32 (dd, J = 12.9, 1.5 Hz, 1H, C(4)-H), 1.91 (p, J = 6.1 Hz, 2H, C(2')-H).

^{13}C NMR (Acetone-d$_6$, 101 MHz): δ (ppm) 167.2 (C=O)), 154.7 (C(5)), 151.5 (C(1)), 130.2 (C(6)), 129.1, (C(8)), 127.5 (C(7)), 99.2 (C(2)), 66.2, (C(1')), 58.9 (C(3')), 52.1 (C(9)), 41.7 (C(4)), 37.7 (C(3)), 32.9 (C(2')).

IR (neat): 3491 (br), 2951 (w), 1788 (m), 1721 (s), 16611 (w), 1436 (w), 1279 (s), 1182 (w), 1151 (w), 1111 (w), 1059 (w), 1019 (w), 963 (w), 854 (w), 769 (w), 706 (w) cm^{-1}.

HRMS (ESI): m/z calculated for $C_{15}H_{19}O_4^+$ $[M+H]^+$ = 263.1278; found = 263.1274.

SFC Chiralpak ® IF; 1500 psi, 30 °C; flow 1.5 mL/min; from 1% to 30% MeOH in 8 min; 98% ee (minor enantiomer t_R = 6.79 min; major enantiomer t_R = 7.38 min).

$[\alpha]_D^{25}$ = +5.3 (c = 0.85, CH_2Cl_2).

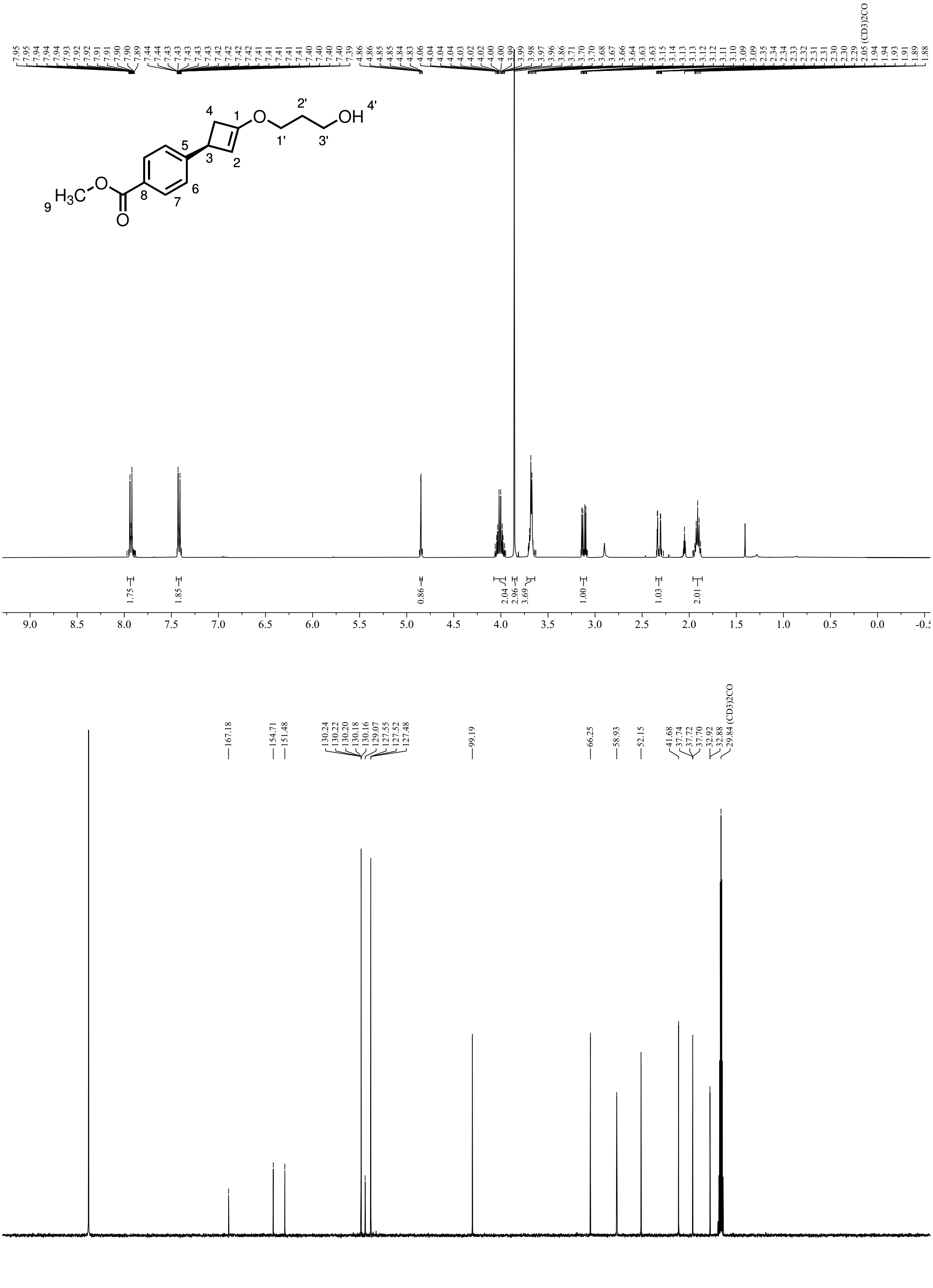

Figure 33: ^{1}H NMR (400 MHz, acetone-d_6, top) and ^{13}C NMR (101 MHz, acetone-d_6, bottom) for **3at**.

Synthesis of 3au

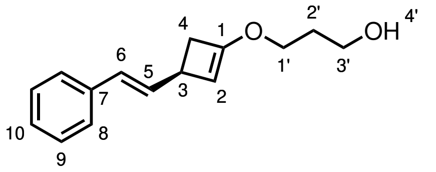

The corresponding compound was prepared following General Procedure A, using trans-2-Phenylvinylboronic acid. Purification by manual flash chromatography (Pentane 80:20 Et_2O) afforded a colorless oil identified as **3ar** (63.5 mg, 69% yield). SFC analysis showed an enantiomeric excess of 81%.

^{1}H NMR (Acetone-d_6, 400 MHz): δ (ppm) 7.43 – 7.36 (m, 2H, C(Ar)-H), 7.35 – 7.24 (m, 2H, C(Ar)-H), 7.24 – 7.14 (m, 1H, C(Ar)-H), 6.50 (d, J = 15.7 Hz, 1H, C(6)-H), 6.32 (dd, J = 15.8, 7.6 Hz, 1H, C(5)-H), 4.70 (s, 1H, C(2)-H), 3.94 (tt, J = 6.4, 3.3 Hz, 2H, C(1')-H), 3.71 – 3.57 (m, 3H, C(3')-H, OH), 3.21 – 3.13 (m, 1H, C(3)-H), 2.91 (dd, J = 12.9, 4.4 Hz, 1H, C(4)-H), 2.30 (dd, J = 12.9, 1.5 Hz, 1H, C(4)-H), 1.88 (p, J = 6.3 Hz, 2H, C(2')-H).

^{13}C NMR (Acetone-d_6, 101 MHz): δ (ppm) 154.1 (C(1)), 138.6 (C(7)), 135.3 (C(6)), 129.38 (C(9)), 129.33 (C(5)), 127.7 (C(9)), 126.8 (C(10)), 99.2 (C(2)), 66.0 (C(1')), 59.0 (C(3')), 38.9 (C(4)), 36.3 (C(3)), 32.9 (C(2')).

IR (neat): 3436 (br), 2938 (w), 1784 (m), 1601 (w), 1494 (w), 1449 (w), 1274 (s), 1203 (s), 1154 (s), 1067 (s), 965 (s), 965 (m), 932 (m), 932 (m), 994 (w), 748 (s), 696 (s), 616 (w) cm^{-1}.

HRMS (ESI): m/z calculated for $C_{15}H_{19}O_2^+$ $[M+H]^+$ = 231.1380; found = 231.1381.

SFC Chiralpak ® IF; 1500 psi, 30 °C; flow 1.5 mL/min; from 1% to 30% MeOH in 8 min; 81% ee (minor enantiomer t_R = 4.98 min; major enantiomer t_R = 7.07 min).

$[\alpha]_D^{25}$ = +4.1 (c = 1.10, CH_2Cl_2).

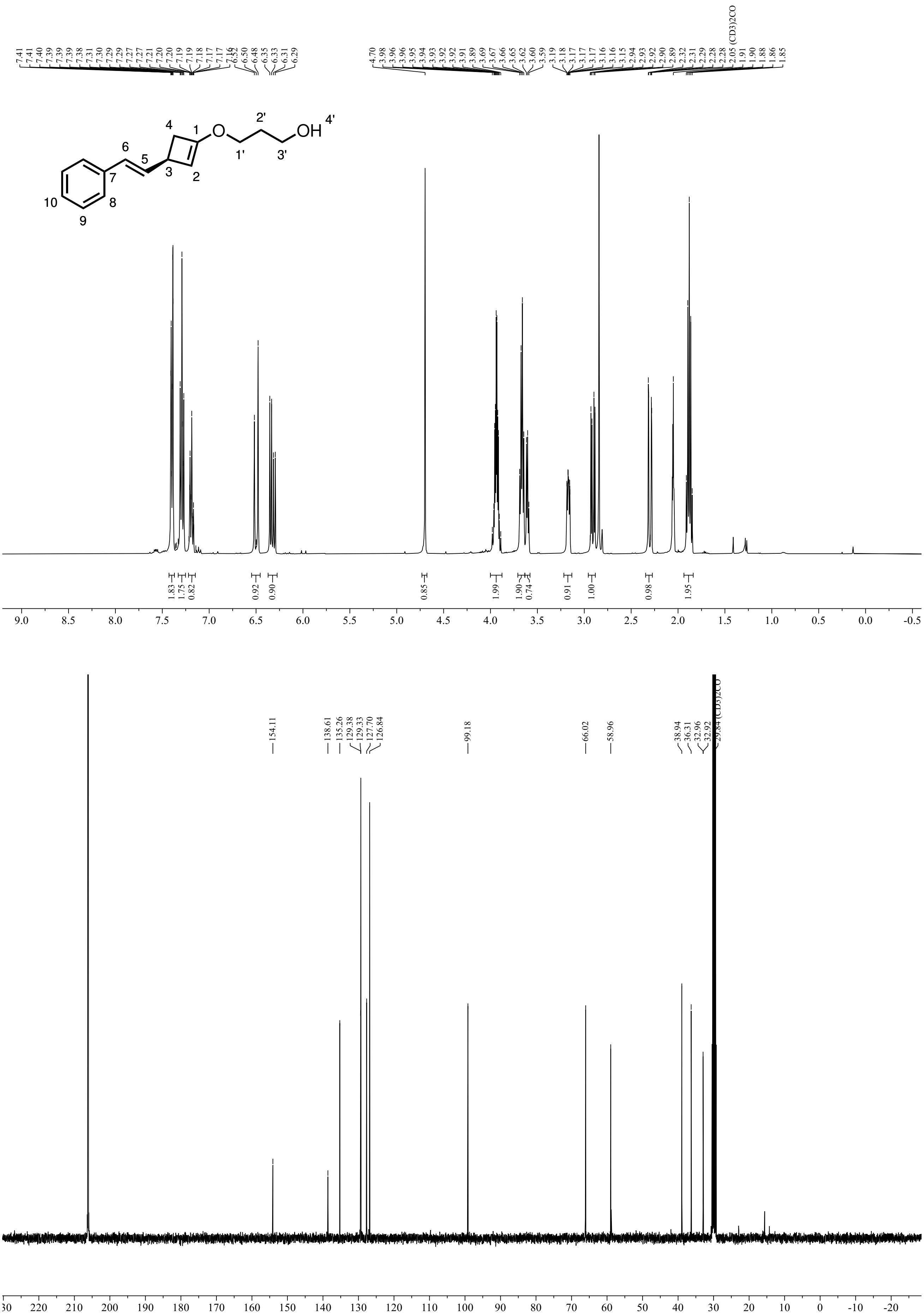

Figure 34: ^{1}H NMR (400 MHz, acetone-d_6, top) and ^{13}C NMR (101 MHz, acetone-d_6, bottom) for **3au**.

Synthesis of 3av

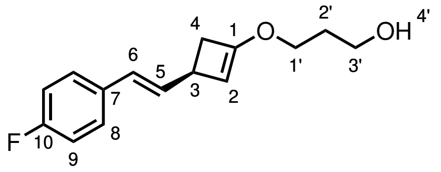

The corresponding compound was prepared following General Procedure A, using trans-2-(4-Fluorophenyl)-vinylboronic acid. Purification by manual flash chromatography (Pentane 80:20 Et_2O) afforded a colorless oil identified as **3ar** (64.6 mg, 65% yield). SFC analysis showed an enantiomeric excess of 81%.

^{1}H NMR (Acetone-d_6, 400 MHz): δ (ppm) 7.48 – 7.38 (m, 2H, C(Ar)-H), 7.12 – 6.99 (m, 2H, C(Ar)-H), 6.49 (dd, J = 15.8, 0.9 Hz, 1H, C(6)-H), 6.27 (dd, J = 15.8, 7.7 Hz, 1H, C(5)-H), 4.69 (d, J = 0.8 Hz, 1H, C(2)-H), 3.93 (tt, J = 6.4, 3.2 Hz, 2H, C(1')-H), 3.71 – 3.59 (m, 3H, C(3')-H, OH), 3.15 (dddt, J = 7.8, 4.5, 1.7, 1.0 Hz, 1H, C(3)-H), 2.94 – 2.86 (m, 1H, C(4)-H), 2.29 (dd, J = 12.9, 1.5 Hz, 1H, C(4)-H), 1.88 (p, J = 6.3 Hz, 2H, C(2')-H).

^{13}C NMR (Acetone-d_6, 101 MHz): δ (ppm) 162.7 (d, J = 243.9 Hz, C(10)), 154.1, C(1), 135.2 (d, J = 2.3 Hz, C(6)), 135.1 (d, J = 3.4 Hz, C(7)), 128.5 (d, J = 7.9 Hz, C(8)), 128.1 (C(5)), 116.0 (d, J = 21.6 Hz, C(9)), 99.1 (C(2)), 66.0 (C(1')), 59.0 (C(3')), 38.9 (C(4)), 36.2 (C(3)), 32.9 (C(2)).

^{19}F NMR (Acetone-d_6, 376 MHz): δ (ppm) -117.3 (tt, J = 8.9, 5.4 Hz)

IR (neat): 3428 (br), 3942 (w), 1782 (w), 1728 (w), 1603 (w), 1510 (s), 1415 (w), 1275 (m), 1224.28 (s), 1156 (s), 1057 (m), 839 (m) cm^{-1}.

HRMS (ESI): m/z calculated for $C_{15}H_{18}O_2^+F$ $[M+H]^+$ = 249.1285; found = 249.1288.

SFC Chiralpak ® IF; 1500 psi, 30 °C; flow 1.5 mL/min; from 1% to 30% MeOH in 5 min; 81% ee (minor enantiomer t_R = 3.60 min; major enantiomer t_R = 4.43 min).

$[\alpha]_D^{25}$ = +5.0 (c = 0.84, CH_2Cl_2).

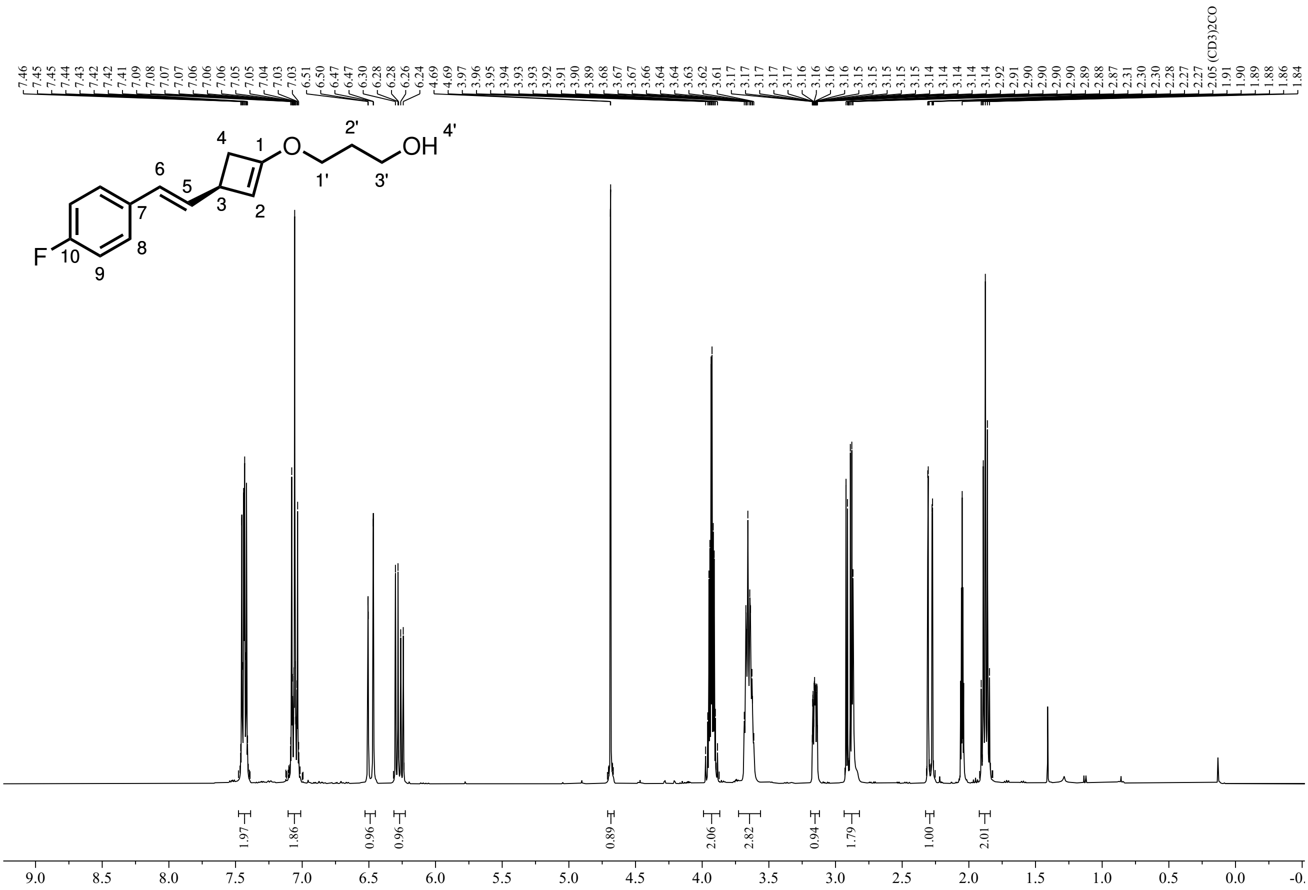

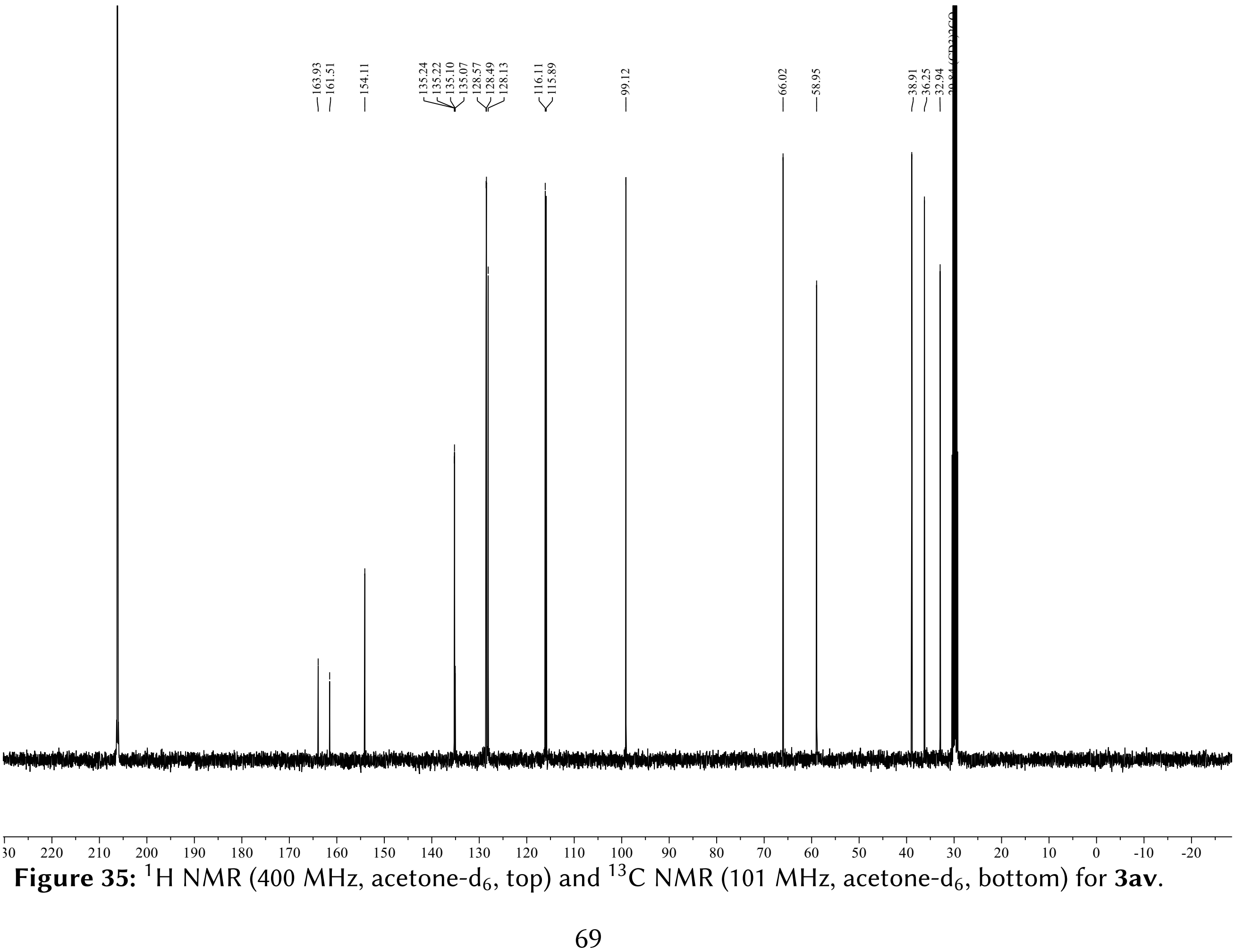

Figure 35: ^{1}H NMR (400 MHz, acetone-d_6, top) and ^{13}C NMR (101 MHz, acetone-d_6, bottom) for **3av**.

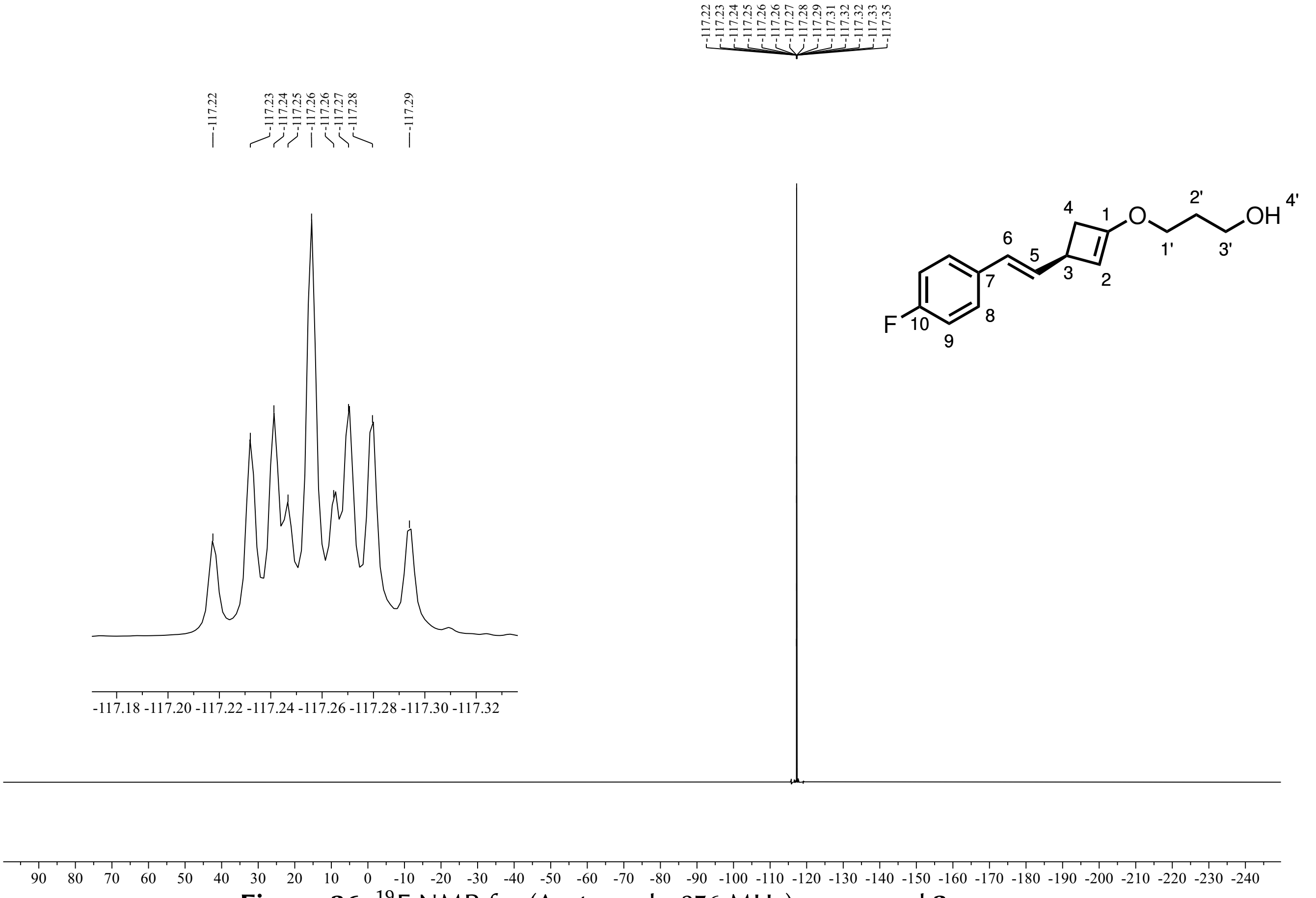

Figure 36: ^{19}F NMR for (Acetone-d_6, 376 MHz) compound **3av**.

Synthesis of 3aw

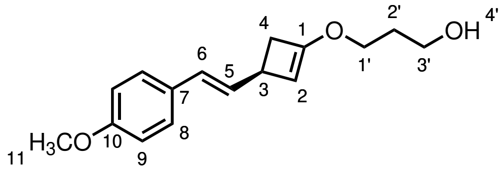

The corresponding compound was prepared following General Procedure A, using trans-2-(4-Methoxy)-vinylboronic acid. Purification by manual flash chromatography (Pentane 80:20 Et_2O) afforded a colorless oil identified as **3aw** (24.0 mg, 23% yield). SFC analysis showed an enantiomeric excess of 79%.

^{1}H NMR (Acetone-d_6, 400 MHz): δ (ppm) 7.38 – 7.28 (m, 2H, C(Ar)-H), 6.92 – 6.82 (m, 2H, C(Ar)-H), 6.43 (d, J = 15.7 Hz, 1H, C(6)-H), 6.16 (dd, J = 15.7, 7.7 Hz, 1H, C(5)-H), 4.68 (d, J = 0.8 Hz, 1H, C(2)-H), 3.93 (tt, J = 6.4, 3.3 Hz, 2H, C(1')-H), 3.78 (s, 3H, C(11)-H), 3.70 – 3.57 (m, 3H), 3.14 (dddd, J = 7.7, 4.4, 1.5, 0.8 Hz, 1H), 2.94 – 2.83 (m, 2H), 2.27 (dd, J = 12.8, 1.5 Hz, 1H), 1.87 (p, J = 6.3 Hz, 2H).

^{13}C NMR (Acetone-d_6, 101 MHz): δ (ppm) 159.8 (C(10)), 154.0 (C(1)), 132.8 (C(6)), 131.3 (C(7)), 128.9 (C(5)), 128.0 (C(Ar)), 114.7 (C(Ar)), 99.3 (C(2)), 66.0 (C(1')), 59.0 (C(3')), 55.5 (C(11)), 39.1 (C(4)), 36.3 (C(3)), 33.0 (C(2')).

IR (neat): 3435 (br), 2937 (w), 1723 (m), 1608 (m), 1513 (s), 1464 (w), 1250 (s), 1175 (s), 1033 (s), 834 (w) cm^{-1}.

HRMS (ESI/APCI): Not found.

Note: We were not able to detect the molecular ion or a characteristic fragment for this molecule.

SFC Chiralpak ® IF; 1500 psi, 30 °C; flow 1.5 mL/min; from 1% to 30% MeOH in 4 min, then 30% to 50% MeOH in 5 minutes; 79% ee (minor enantiomer t_R = 4.46 min; major enantiomer t_R = 3.82 min).

$[\alpha]_D^{25}$ = +7.2 (c = 0.29, CH_2Cl_2).

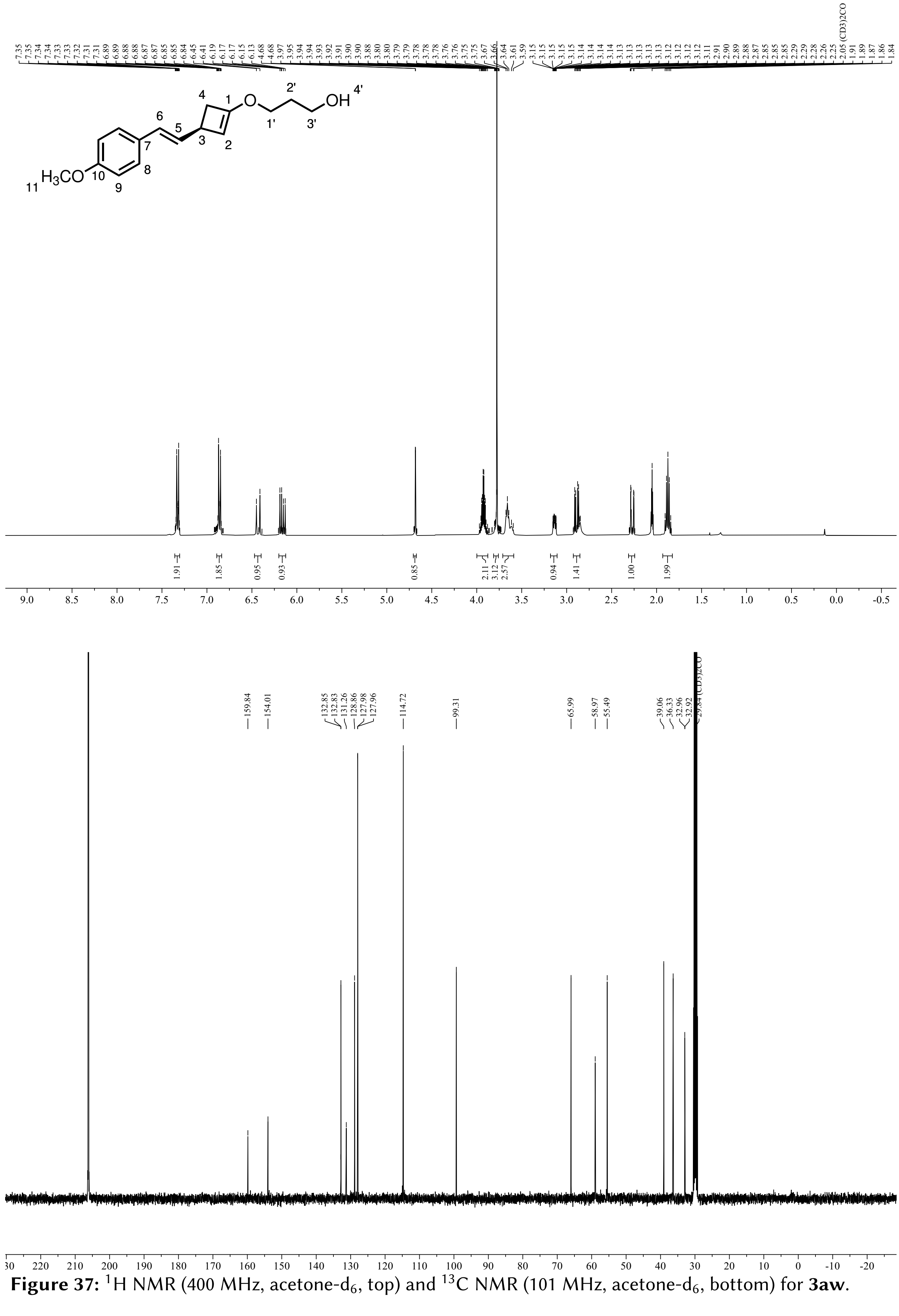

Figure 37: ^{1}H NMR (400 MHz, acetone-d$_{6}$, top) and ^{13}C NMR (101 MHz, acetone-d$_{6}$, bottom) for **3aw**.

Synthesis of 3ba

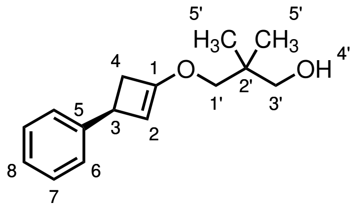

The corresponding compound was prepared following General Procedure C, using phenylboronic acid. Purification by manual flash chromatography (Pentane 80:20 Et_2O) afforded a colorless oil identified as **3ba** (67.8 mg, 73% yield). SFC analysis showed an enantiomeric excess of 96%.

^{1}H NMR (Acetone-d_6, 400 MHz): δ (ppm) 7.33 – 7.22 (m, 4H, C(Ar)-H), 7.22 – 7.12 (m, 1H, C(Ar)-H), 4.81 (d, J = 0.9 Hz, 1H, C(2)-H), 3.76 – 3.64 (m, 3H, C(1')-H, OH), 3.59 (dt, J = 4.6, 1.2 Hz, 1H, C(3)-H), 3.43 – 3.36 (m, 2H, C(3')-H), 3.09 (dd, J = 12.7, 4.6 Hz, 1H, C(4)-H), 2.30 (dd, J = 12.7, 1.5 Hz, 1H, C(4)-H), 0.95 (s, 6H, C(5')-H).

^{13}C NMR (Acetone-d_6, 101 MHz): δ (ppm) 154.7 (C(1)), 145.8 (C(5)), 129.0 (C(7)), 127.3 (C(6)), 126.9 (C(8)), 99.3 (C(2)), 74.5 (C(1')), 68.3 (C(3')), 41.8 (C(4)), 37.8 (C(3)), 37.0 (C(2')), 21.8 (C(5')).

IR (neat): 3378 (br), 2959 (w), 1632 (s), 1510 (w), 1474 (w), 1405 (w), 1303 (m), 1216 (w), 1024 (m), 905 (w), 838 (w), 774 (w), 748 (w) cm^{-1}.

HRMS (ESI): m/z calculated for $C_{15}H_{21}O_2^+$ $[M+H]^+$ = 233.1536; found = 233.1536.

SFC Chiralpak ® IF; 1500 psi, 30 °C; flow 1.5 mL/min; from 1% to 30% MeOH in 5 min; 96% ee (minor enantiomer t_R = 3.06 min; major enantiomer t_R = 3.33 min).

$[\alpha]_D^{25}$ = +36.6 (c = 1.07, CH_2Cl_2).

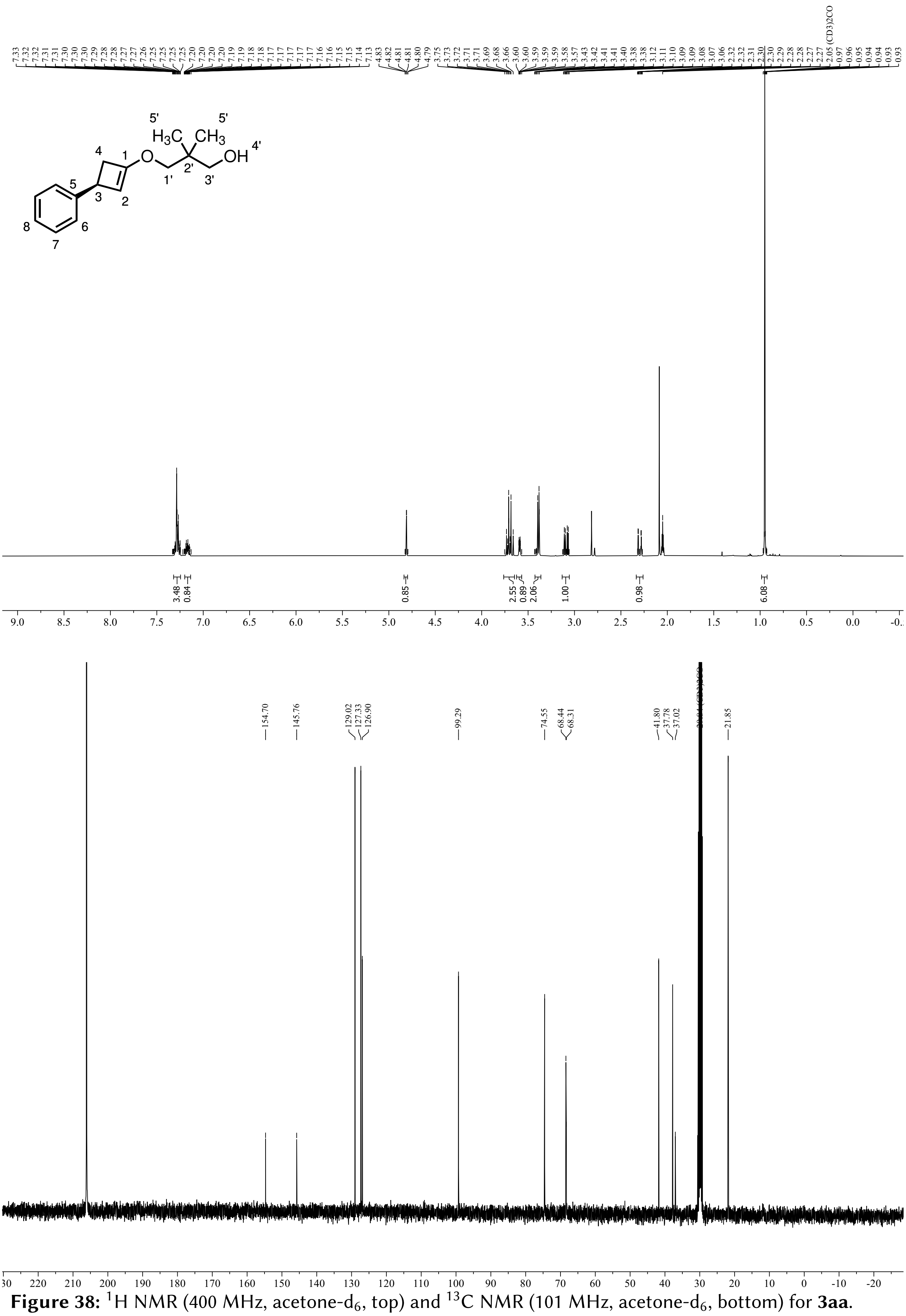

Figure 38: ^{1}H NMR (400 MHz, acetone-d_6, top) and ^{13}C NMR (101 MHz, acetone-d_6, bottom) for **3aa**.

Synthesis of 3bb

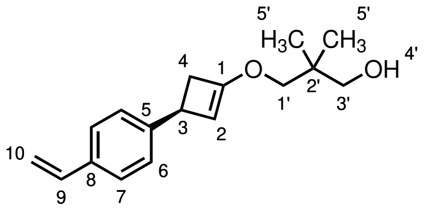

The corresponding compound was prepared following General Procedure C, using 4-vinyl-phenylboronic acid. Purification by manual flash chromatography (Pentane 80:20 Et_2O) afforded a colorless oil identified as **3bb** (66.1 mg, 73% yield). SFC analysis showed an enantiomeric excess of 97%.

^{1}H NMR (Acetone-d_6, 400 MHz): δ (ppm) 7.42 – 7.34 (m, 2H, C(Ar)-H), 7.32 – 7.21 (m, 2H, C(Ar)-H), 6.72 (dd, J = 17.6, 10.9 Hz, 1H, C(10)-H), 5.75 (dd, J = 17.7, 1.1 Hz, 1H, C(9)-H), 5.17 (dd, J = 10.9, 1.1 Hz, 1H, C(9)-H), 4.80 (d, J = 0.9 Hz, 1H, C(2)-H), 3.78 – 3.63 (m, 3H, C(1')-H, OH), 3.59 (dt, J = 4.3, 1.2 Hz, 1H, C(3)-H), 3.39 (d, J = 5.5 Hz, 2, C(3')-H), 3.09 (dd, J = 12.8, 4.6 Hz, 1H, C(4)-H), 2.30 (dd, J = 12.8, 1.6 Hz, 1H, C(4)-H), 0.95 (s, 6H, C(5')-H).

^{13}C NMR (Acetone-d_6, 101 MHz): δ (ppm) 154.7 (C(1)), 145.7 (C(5)), 137.7 (C(9)), 136.5 (C(8)), 127.6 (C(7)), 126.9 (C(6)), 113.1 (C(10)), 99.2 (C(2)), 74.5 (C(1')), 68.4 (C(3')), 41.8 (C(4)), 37.5 (C(3)), 37.0 (C(2')), 21.8 (C(5')).

IR (neat): 3378 (br), 2959 (w), 1632 (s), 1510 (w), 1474 (w), 1405 (w), 1303 (m), 1216 (w), 1024 (m), 905 (w), 838 (w), 774 (w), 748 (w) cm^{-1}.

HRMS (ESI): m/z calculated for $C_{17}H_{23}O_2^+$ $[M+H]^+$ = 259.1693; found = 259.1696.

SFC Chiralpak ® IF; 1500 psi, 30 °C; flow 1.5 mL/min; from 1% to 30% MeOH in 5 min; 97% ee (minor enantiomer t_R = 3.77 min; major enantiomer t_R = 4.27 min).

$[\alpha]_D^{25}$ = +34.7 (c = 1.11, CH_2Cl_2).

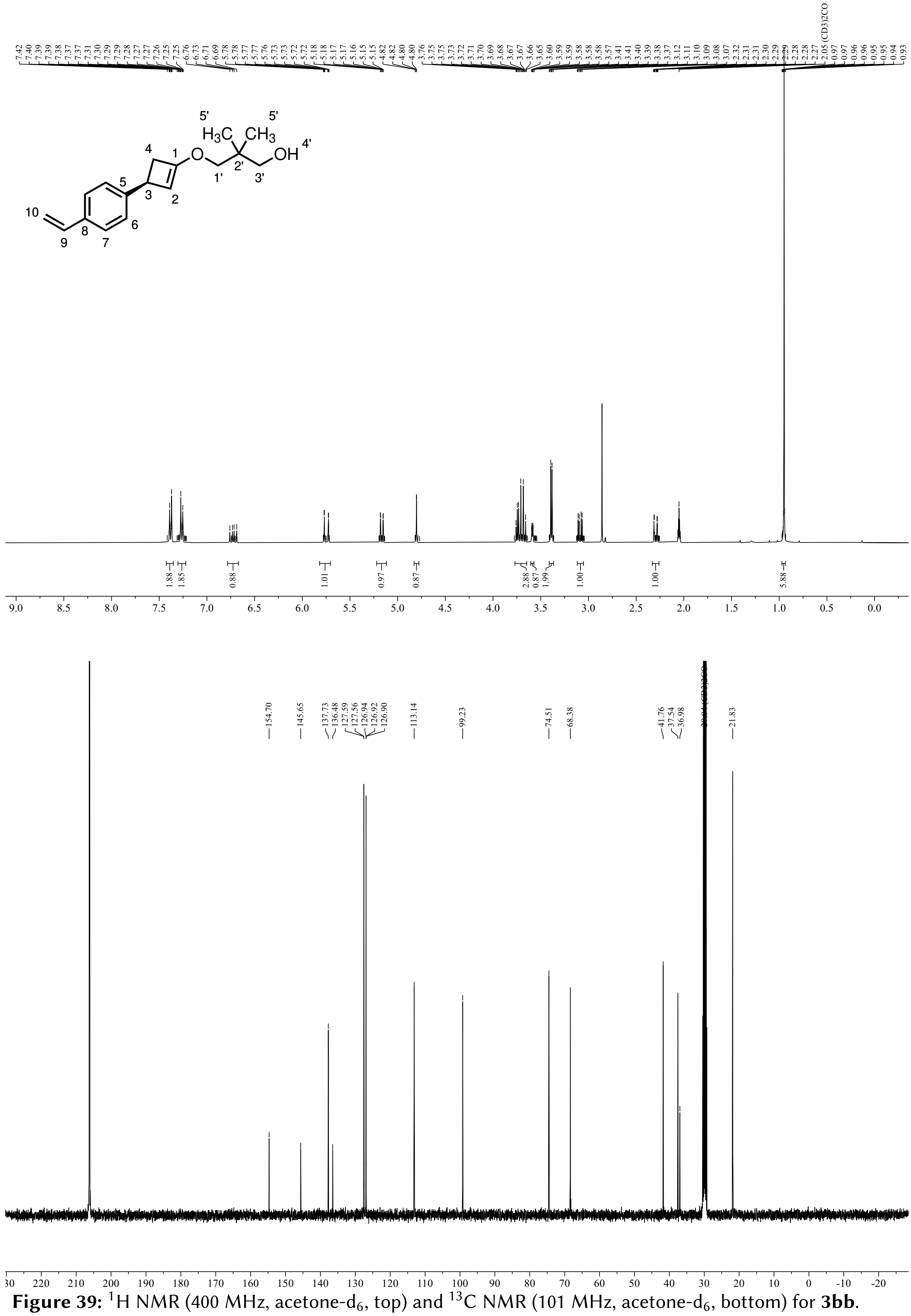

Figure 39: ^{1}H NMR (400 MHz, acetone-d_6, top) and ^{13}C NMR (101 MHz, acetone-d_6, bottom) for **3bb**.

Synthesis of 3bc

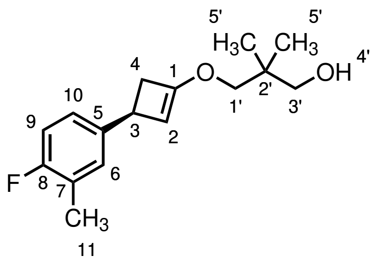

The corresponding compound was prepared following General Procedure C, using 4-Fluoro-3-methylphenylboronic acid. Purification by manual flash chromatography (Pentane 80:20 Et_2O) afforded a colorless oil identified as **3bc** (69.8 mg, 66% yield). SFC analysis showed an enantiomeric excess of 97%.

^{1}H NMR (Acetone-d_6, 400 MHz): δ (ppm) 7.16 (dd, J = 7.6, 2.3 Hz, 1H, C(Ar)-H), 7.11 (dddd, J = 8.5, 5.1, 2.5, 0.6 Hz, 1H, C(Ar)-H), 6.95 (dd, J = 9.8, 8.3 Hz, 1H, C(Ar)-H), 4.78 (d, J = 0.9 Hz, 1H, C(2)-H), 3.78 – 3.62 (m, 3H, C(1')-H, OH), 3.55 (d, J = 4.5 Hz, 1H, C(3)-H), 3.38 (d, J = 5.5 Hz, 2H, C(3')-H), 3.07 (dd, J = 12.7, 4.6 Hz, 1H, C(4)-H), 2.26 (dd, J = 12.8, 1.6 Hz, 2H, C(4)-H), 2.22 (d, J = 2.0 Hz, 3H, C(11)-H), 0.94 (s, 6H, C(5')-H).

^{13}C NMR (Acetone-d_6, 101 MHz): δ (ppm) 160.7 (d, J = 240.6 Hz, C(8)), 154.7 (C(1)), 141.4 (d, J = 3.4 Hz, C(5)), 130.4 (d, J = 4.8 Hz, C(6)), 126.2 (d, J = 7.9 Hz, C(10)), 124.8 (d, J = 17.1 Hz, C(8)), 115.2 (d, J = 22.2 Hz, C(9)), 99.3 (C(2)), 74.5 (C(1')), 68.4 (C(3')), 41.9 (C(4)), 37.05 (C(3)), 36.97 (C(2')) 21.8 (C(5')), 14.5 (d, J = 3.7 Hz, C(11)).

^{19}F NMR (Acetone-d_6, 376 MHz): δ (ppm) -123.32 (dddt, J = 9.6, 7.2, 4.5, 2.1 Hz).

IR (neat): 3356 (br), 2959 (w), 1633 (s), 1502 (m), 1475 (w), 1301 (m), 1243 (m), 1214 (w), 1143 (m), 1117 (w), 1025 (m) 884 (w), 819 (w) cm^{-1}.

HRMS (ESI): m/z calculated for $C_{16}H_{22}FO_2^+$ $[M+H]^+$ = 265.1598; found = 265.1607.

SFC Chiralpak ® IF; 1500 psi, 30 °C; flow 1.5 mL/min; from 1% to 30% MeOH in 5 min; 97% ee (minor enantiomer t_R = 2.55 min; major enantiomer t_R = 2.68 min).

$[\alpha]_D^{25}$ = +40.2 (c = 0.96, CH_2Cl_2).

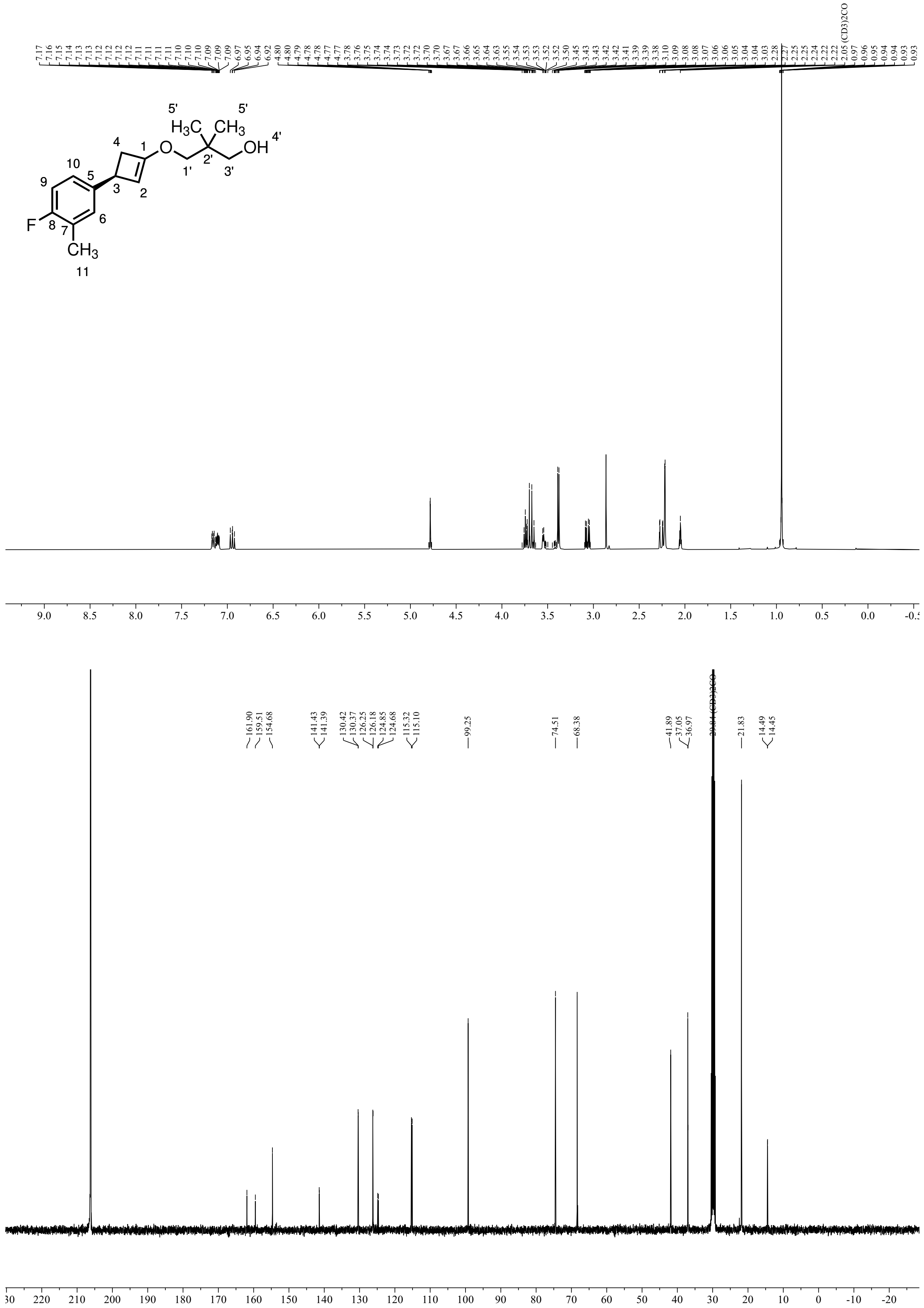

Figure 40: ^{1}H NMR (400 MHz, acetone-d$_6$, top) and ^{13}C NMR (101 MHz, acetone-d$_6$, bottom) for **3bc**.

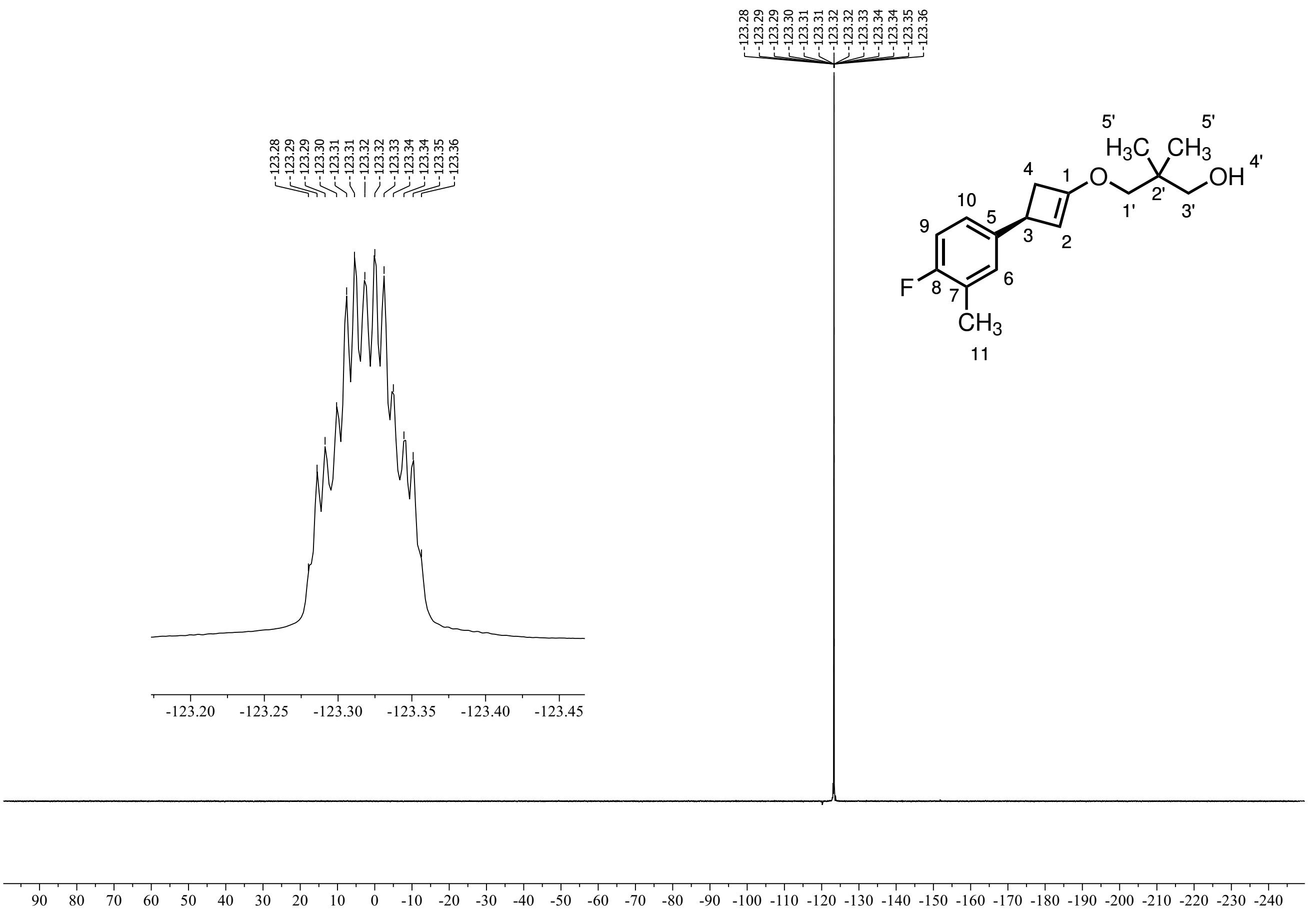

Figure 41: ^{19}F NMR (Acetone-d_6, 376 MHz) for **3bc**.

Synthesis of 3ca

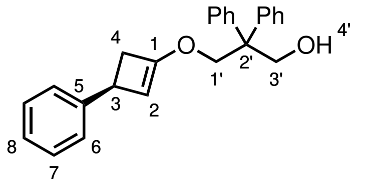

The corresponding compound was prepared following General Procedure D, using phenylboronic acid. Purification by manual flash chromatography (Pentane 80:20 Et_2O) afforded a colorless oil identified as **3ca** (98,4 mg, 69% yield). SFC analysis showed an enantiomeric excess of 97%.

^{1}H NMR (Acetone-d_6, 400 MHz): δ (ppm) 7.34 – 7.14 (m, 15H, C(Ar)-H), 4.95 (d, J = 0.9 Hz, 1H, C(2)-H), 4.63 (d, J = 9.8 Hz, 1H, C(1')-H), 4.58 (d, J = 9.8 Hz, 1H, C(1')-H), 4.37 – 4.27 (m, 2H, C(3')-H), 3.98 (t, J = 5.5 Hz, 1H, OH), 3.58 (dt, J = 4.8, 1.2 Hz, 1H, C(3)-H), 3.04 (dd, J = 12.7, 4.5 Hz, 1H, C(4)-H), 2.25 (dd, J = 12.8, 1.6 Hz, 1H, C(4)-H).

^{13}C NMR (Acetone-d_6, 101 MHz): δ (ppm) 154.1 (C(1)), 145.5 (C(Ar)), 145.4 (C(Ar)), 129.1 (C(Ar)), 128.9 (C(Ar)), 128.8 (C(Ar)), 127.3 (C(Ar)), 127.1 (C(Ar)), 126.9 (C(Ar)), 100.2 (C(2)), 72.0 (C(1')), 66.5 (C(3')), 52.9 (C(2')), 41.8 (C(4)), 37.8 (C(3)).

IR (neat): 3573 (br), 2928 (w), 1630 (m), 1494 (w), 1477 (w), 1300 (m), 1217 (w), 1022 (m), 791 (m), 755 (m), 699 (s) cm^{-1}.

HRMS (ESI): m/z calculated for $C_{25}H_{24}O_2^+$ $[M+H]^+$ = 357.1849; found = 357.1851.

SFC Chiralpak ® IF; 1500 psi, 30 °C; flow 1.5 mL/min; from 1% to 30% MeOH in 5 min; 97% ee (minor enantiomer t_R = 5.52 min; major enantiomer t_R = 5.83 min).

$[\alpha]_D^{25}$ = +27.3 (c = 1.11, CH_2Cl_2).

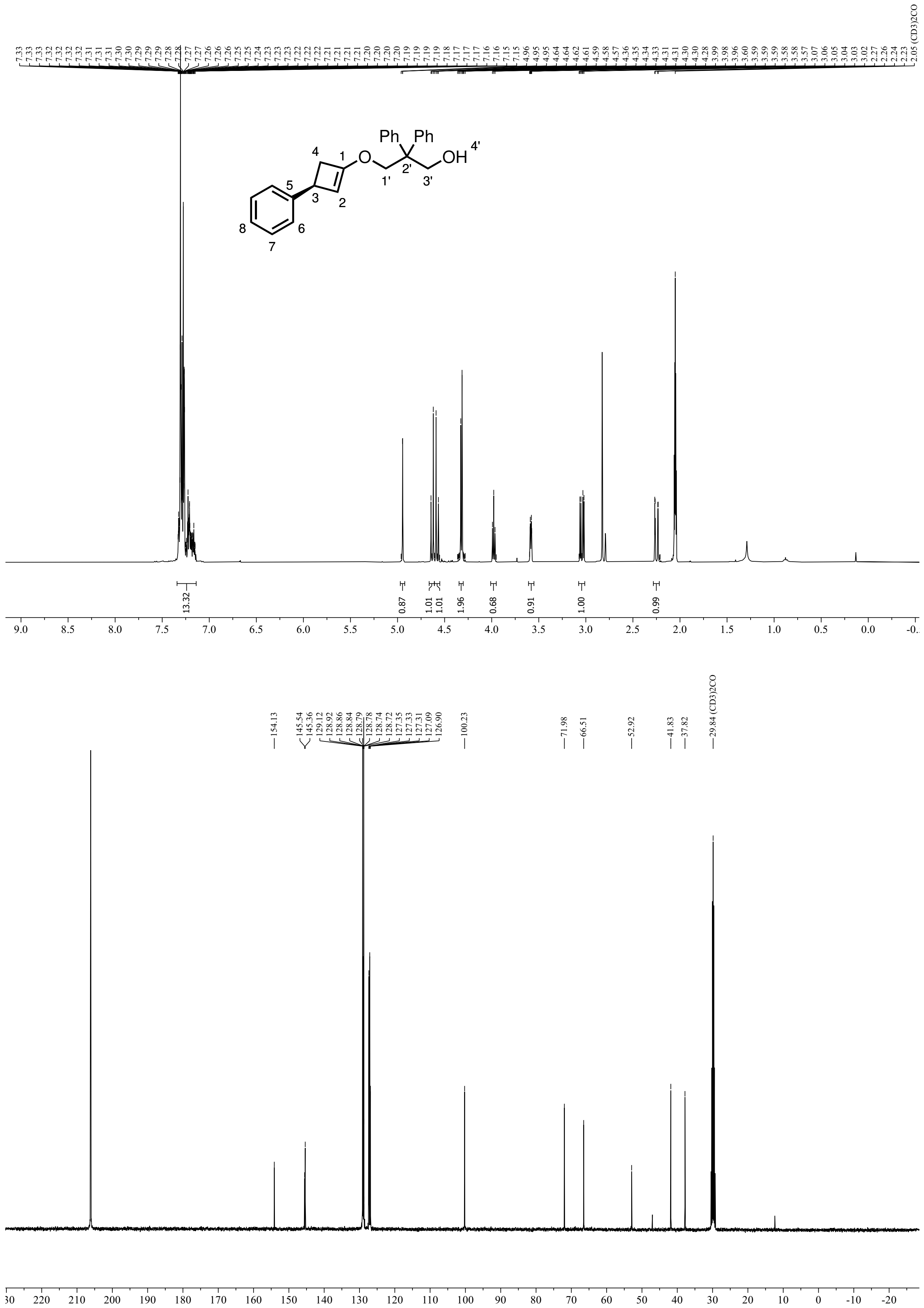

Figure 42: ^{1}H NMR (400 MHz, acetone-d_6, top) and ^{13}C NMR (101 MHz, acetone-d_6, bottom) for **3ca**.

Synthesis of 3cb

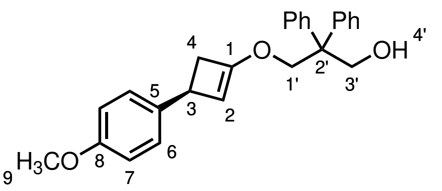

The corresponding compound was prepared following General Procedure D, using 4-methoxyphenylboronic acid. Purification by manual flash chromatography (Pentane 80:20 Et_2O) afforded a colorless oil identified as **3cb** (100.5 mg, 65% yield). SFC analysis showed an enantiomeric excess of 96%.

^{1}H NMR (Acetone-d_6, 400 MHz): δ (ppm) 7.34 – 7.16 (m, 12H, C(Ar)-H), 6.88 – 6.79 (m, 2H, C(Ar)-H), 4.91 (d, J = 0.9 Hz, 1H, C(2)-H), 4.62 (d, J = 9.8 Hz, 1H, C(1')-H), 4.57 (d, J = 9.8 Hz, 1H, C(1')-H), 4.32 (d, J = 5.6 Hz, 2H, C(3')-H), 3.94 (t, J = 5.5 Hz, 1H, OH), 3.56 – 3.50 (m, 1H, C(3)-H), 3.01 (dd, J = 12.7, 4.5 Hz, 1H, C(4)-H), 2.21 (dd, J = 12.7, 1.5 Hz, 1H, C(4)-H).

^{13}C NMR (Acetone-d_6, 101 MHz): δ (ppm) 159.2 (C(Ar)), 154.0 (C(1)), 145.4 (C(Ar)), 137.4 (C(Ar)), 129.1 (C(Ar)), 128.8 (C(Ar)), 128.3 (C(Ar)), 127.1 (C(Ar)), 114.4 (C(Ar)), 100.4 (C(2)), 72.0 (C(1')), 66.5 (C(3')), 55.5 (C(9)), 52.9 (C(2')), 42.0 (C(4)), 37.2 (C(3)).

IR (neat): 3451 (br), 2929 (w), 1630 (s), 1610 (m), 1581 (w), 1510 (s), 1465 (w), 1446 (w), 1299 (s), 1245 (s), 1175 (m), 1035 (s), 829 (m), 805 (m), 756 (m), 700 (s), 633 (w) cm^{-1}.

HRMS (ESI): m/z calculated for $C_{26}H_{27}O_3^+$ $[M+H]^+$ = 387.1955; found = 387.1963.

SFC Chiralpak ® IF; 1500 psi, 30 °C; flow 1.5 mL/min; from 1% to 30% MeOH in 5 min; 96% ee (minor enantiomer t_R = 6.33 min; major enantiomer t_R = 6.56 min).

$[\alpha]_D^{25}$ = +25.6 (c = 1.00, CH_2Cl_2).

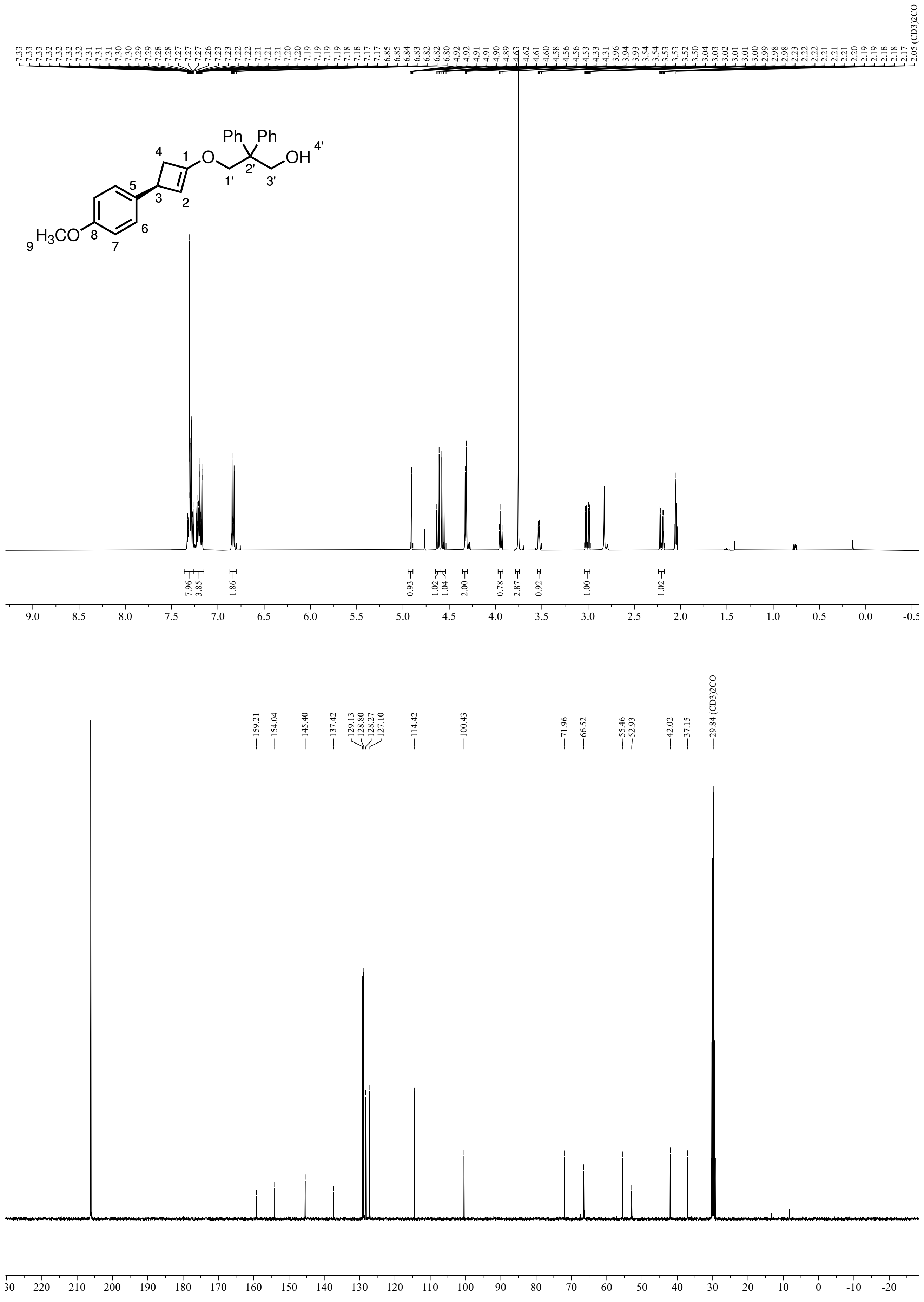

Figure 43: ^{1}H NMR (400 MHz, acetone-d_6, top) and ^{13}C NMR (101 MHz, acetone-d_6, bottom) for **3cb**.

Synthesis of 3cc

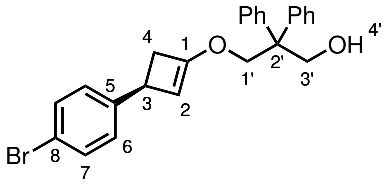

The corresponding compound was prepared following General Procedure D, using 4-bromo-phenylboronic acid. Purification by manual flash chromatography (Pentane 80:20 Et_2O) afforded a colorless oil identified as **3cc** (45.3 mg, 26% yield). SFC analysis showed an enantiomeric excess of 97%.

^{1}H NMR (Acetone-d_6, 400 MHz): δ (ppm) 7.49 – 7.40 (m, 2H, C(Ar)-H), 7.34 – 7.18 (m, 12H, C(Ar)-H), 4.92 (d, J = 0.9 Hz, 1H, C(2)-H), 4.63 (d, J = 9.9 Hz, 1H, C(1')-H), 4.59 (d, J = 9.9 Hz, 1H, C(1')-H), 4.32 (d, J = 5.5 Hz, 2H, C(3')-H), 3.95 (t, J = 5.5 Hz, 1H, OH), 3.57 (dt, J = 4.4, 1.3 Hz, 1H, C(3)-H), 3.05 (dd, J = 12.8, 4.6 Hz, 1H, C(4)-H), 2.23 (dd, J = 12.8, 1.6 Hz, 1H, C(4)-H).

^{13}C NMR (Acetone-d_6, 101 MHz): δ (ppm) 154.4 (C(1)), 145.4 (C(Ar)), 145.1 (C(Ar)), 132.0 (C(Ar)), 129.5 (C(Ar)), 129.1 (C(Ar)), 128.8 (C(Ar)), 127.1 (C(Ar)), 120.1 (C(Ar)), 100.0 (C(2)), 72.0 (C(1')), 66.5 (C(3')), 52.9 (C(2')), 41.8 (C(4)), 37.3 (C(3)).

IR (neat): 3481 (br), 2930 (w), 1630 (m), 1581 (W), 1510 (m), 1465 (w), 1446 (w), 1298 (m), 1245 (m), 1216 (m), 1175 (m), 1131 (m), 1035 (m), 830 (w), 805 (w), 755 (m), 700 (s), 633 (w) cm^{-1}.

HRMS (ESI): m/z calculated for $C_{25}H_{23}BrO_2Na^+$ $[M+Na]^+$ = 457.0774; found = 457.0774.

SFC Chiralpak ® IF; 1500 psi, 30 °C; flow 1.5 mL/min; from 1% to 30% MeOH in 5 min; 97% ee (minor enantiomer t_R = 6.34 min; major enantiomer t_R = 6.59 min).

$[\alpha]_D^{25}$ = +23.9 (c = 0.85, CH_2Cl_2).

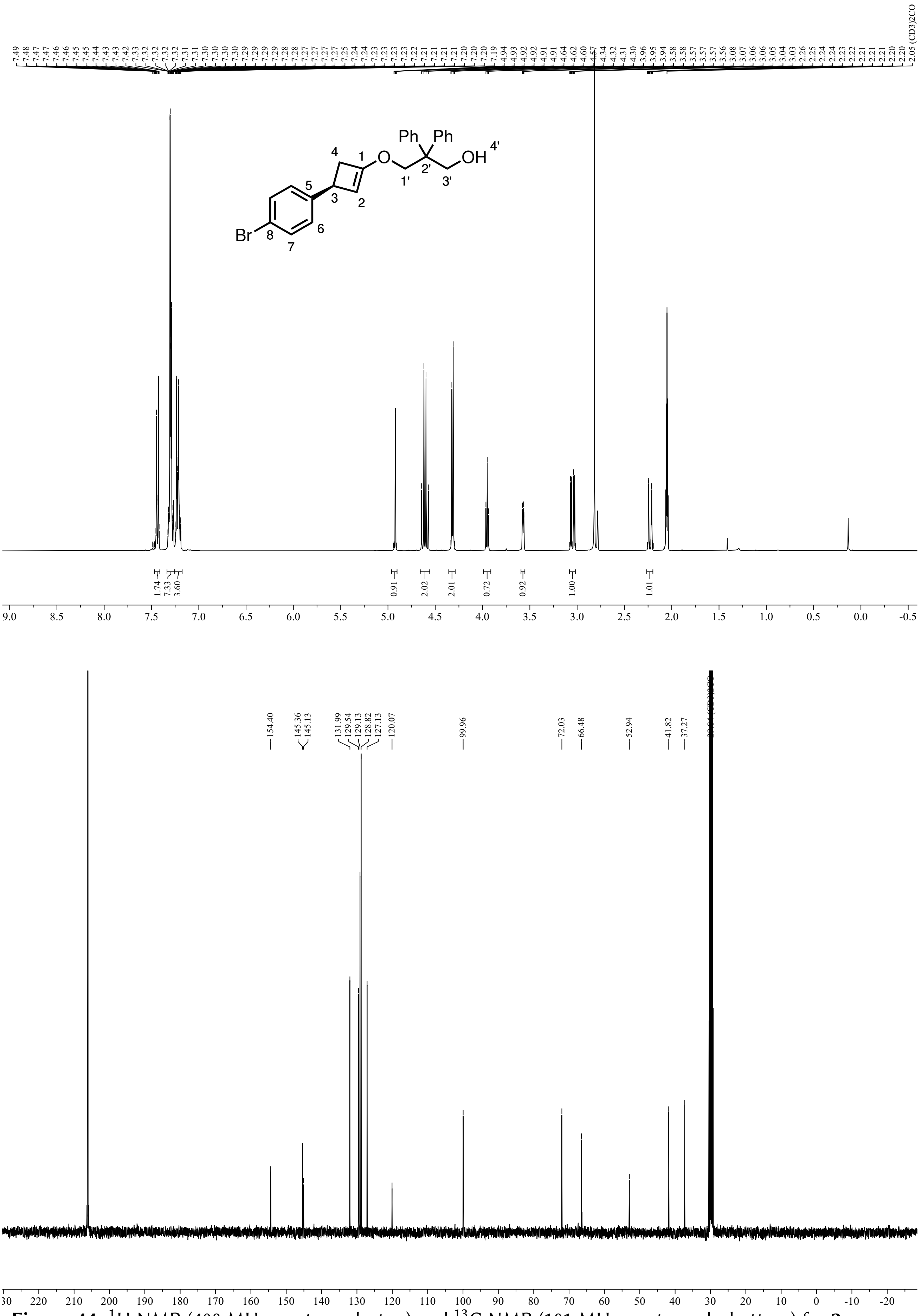

Figure 44: ^{1}H NMR (400 MHz, acetone-d$_6$, top) and ^{13}C NMR (101 MHz, acetone-d$_6$, bottom) for **3cc**.

Synthesis of 3da

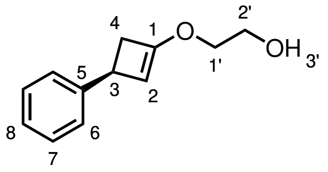

The corresponding compound was prepared following General Procedure E, using phenylboronic acid. Purification by manual flash chromatography (Pentane 80:20 Et_2O) afforded a colorless oil identified as **3da** (63.9 mg, 84% yield). SFC analysis showed an enantiomeric excess of 90%.

^{1}H NMR (Acetone-d_6, 400 MHz): δ (ppm) 7.35 – 7.23 (m, 4H, C(Ar)-H), 7.21 – 7.12 (m, 1H, C(Ar)-H), 4.83 (d, J = 0.9 Hz, 1H, C(Ar)-H), 4.00 – 3.88 (m, 3H, C(1')-H, OH), 3.80 (ddd, J = 6.3, 5.6, 4.6 Hz, 2H, C(2')-H), 3.60 (dt, J = 4.6, 1.2 Hz, 1H, C(3)-H), 3.09 (dd, J = 12.8, 4.6 Hz, 1H, C(4)-H), 2.30 (dd, J = 12.8, 1.6 Hz, 1H, C(4)-H).

^{13}C NMR (Acetone-d_6, 101 MHz): δ (ppm) 154.4 (C(1)), 145.6 (C(5)), 129.0 (C(7)), 127.3 (C(6)), 126.9 (C(8)), 99.6 (C(2)), 70.9 (C(1')), 61.0 (C(2')), 41.8 (C(4)), 37.7 (C(3)).

IR (neat): 3372 (br), 2927 (w), 1633 (s), 1493 (w), 1453 (w), 1303 (s), 1217 (m), 1083 (m), 1044 (m), 956 (m), 943 (m), 893 (w), 791 (m), 761 (m), 700 (m) cm^{-1}.

HRMS (ESI): m/z calculated for $C_{12}H_{15}O_2^+$ $[M+H]^+$ = 191.1067; found = 191.1062.

SFC Chiralpak ® IB; 1500 psi, 30 °C; flow 1.5 mL/min; from 1% to 30% MeOH in 5 min; 90% ee (major enantiomer t_R = 2.60 min; minor enantiomer t_R = 2.71 min).

$[\alpha]_D^{25}$ = +37.2 (c = 1.06, CH_2Cl_2).

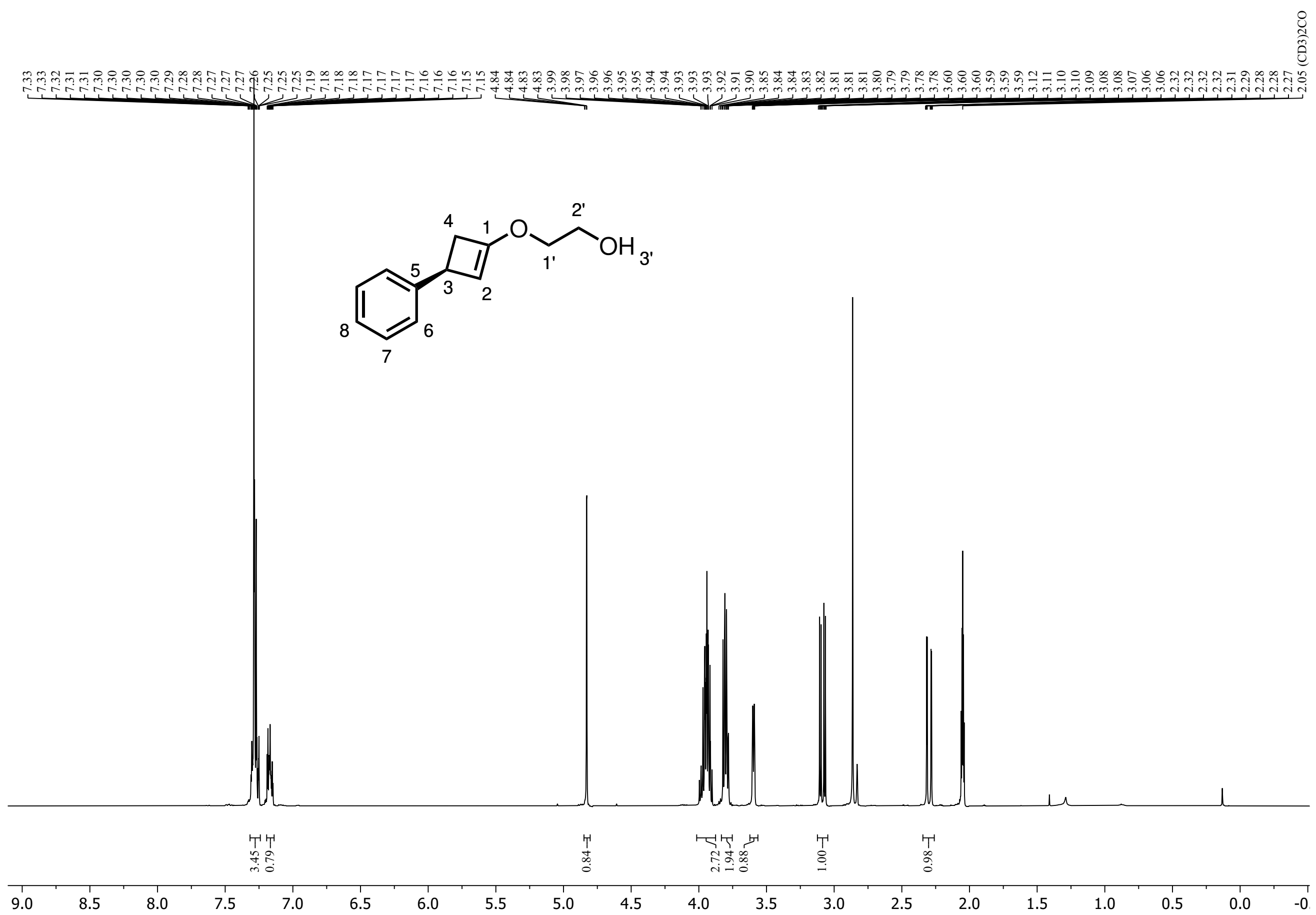

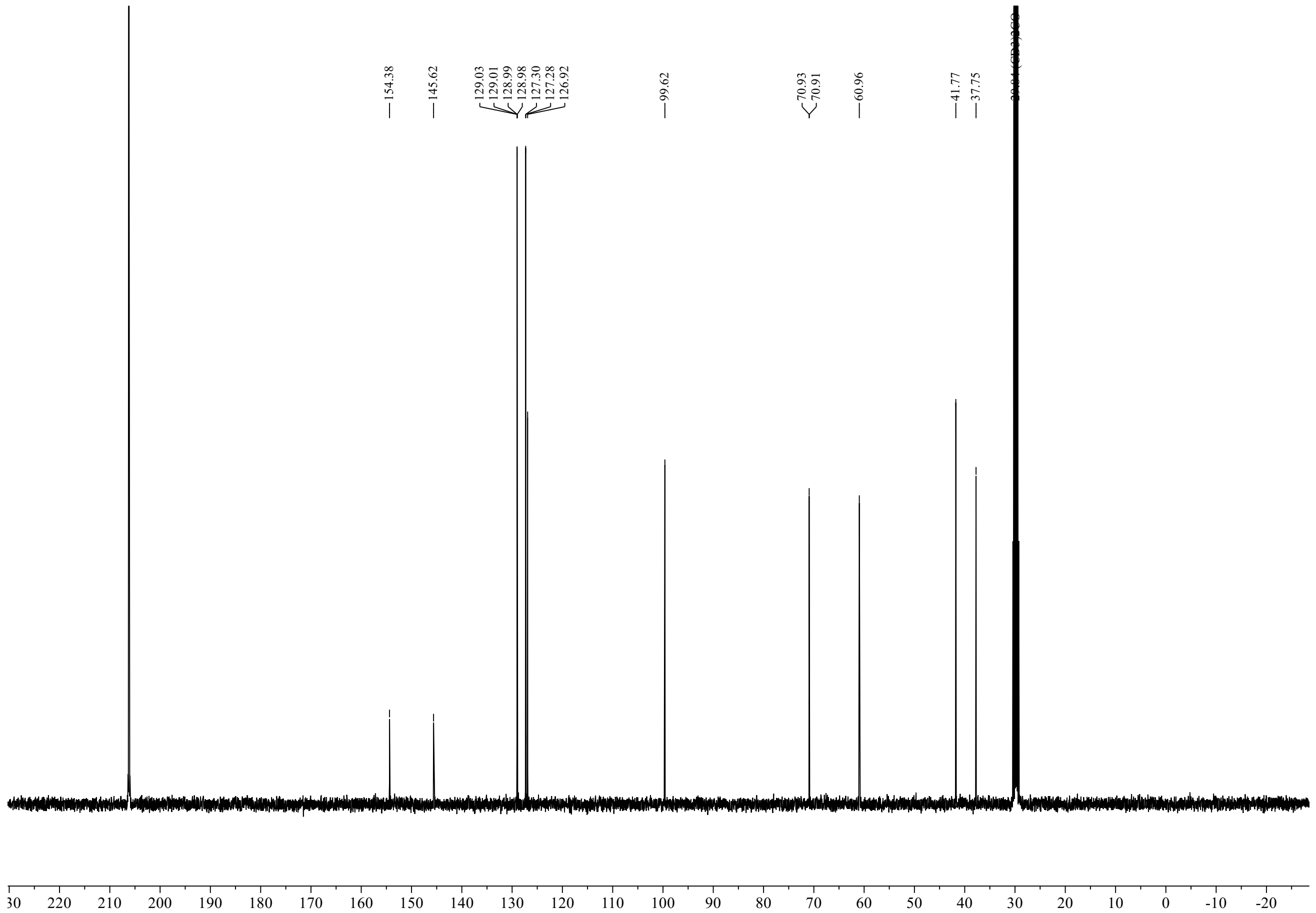

Figure 45: ^{1}H NMR (400 MHz, acetone-d$_6$, top) and ^{13}C NMR (101 MHz, acetone-d$_6$, bottom) for **3da**.

Synthesis of 3db

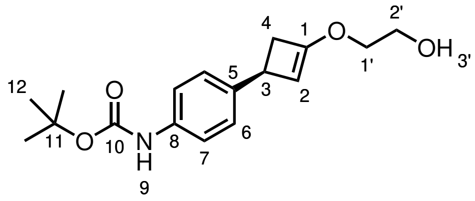

The corresponding compound was prepared following General Procedure E, using 4-(N-Boc-amino)phenylboronic acid. Purification by manual flash chromatography (Pentane 80:20 Et_2O) afforded a colorless oil identified as **3db** (91.6 mg, 75% yield). SFC analysis showed an enantiomeric excess of 87%.

^{1}H NMR (Acetone-d_6, 400 MHz): δ (ppm) 8.30 (s, 1H, NH), 7.49 – 7.43 (m, 2H, C(Ar)-H), 7.24 – 7.15 (m, 2H, C(Ar)-H), 4.80 (d, J = 0.9 Hz, 1H, C(2)-H), 4.01 – 3.87 (m, 3H, C(1')-H, OH), 3.86 – 3.75 (m, 2H, C(2')-H), 3.55 (dt, J = 4.3, 1.2 Hz, 1H, C(3)-H), 3.06 (dd, J = 12.7, 4.6 Hz, 1H, C(4)-H), 2.26 (dd, J = 12.7, 1.6 Hz, 1H, C(4)-H), 1.47 (s, 9H, C(12)-H).

^{13}C NMR (Acetone-d_6, 101 MHz): δ (ppm) 153.4 (C(1)), 152.9 (C(10)), 138.5 (C(5)), 137.9 (C(8)), 126.6 (C(7)), 118.2 (C(6)), 98.9 (C(2)), 78.9 (C(11)), 70.0 (C(1')), 60.1 (C(2)), 41.0(C(4)), 36.4 (C(3)), 27.7 (C(12)).

IR (neat): 3332 (br), 2980 (m), 1703 (m), 1633 (m), 1596 (w), 1527 (m), 1453 (w), 1412 (w), 1368 (m), 1306 (m), 1244 (m), 1162 (s), 1055 (m), 947 (w), 834 (w), 670 (w) cm^{-1}.

HRMS (ESI): m/z calculated for $C_{17}H_{24}NO_4^+$ $[M+H]^+$ = 306.1700; found = 306.1702.

SFC Chiralpak ® IB; 1500 psi, 30 °C; flow 1.5 mL/min; from 1% to 30% MeOH in 8 min; 87% ee (major enantiomer t_R = 5.22 min; minor enantiomer t_R = 5.60 min).

$[\alpha]_D^{25}$ = +18.8 (c = 1.03, CH_2Cl_2).

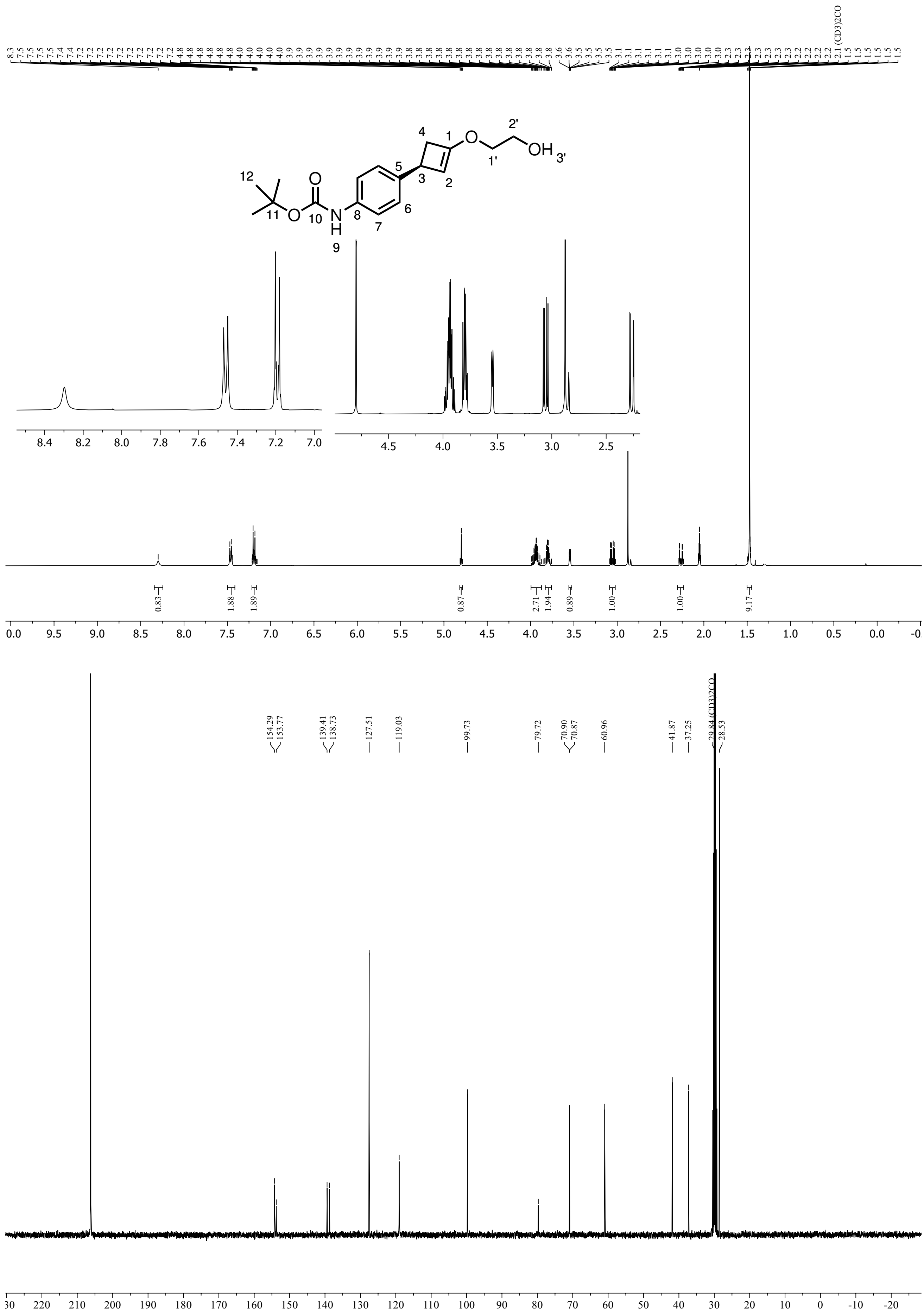

Figure 46: ^{1}H NMR (400 MHz, acetone-d_6, top) and ^{13}C NMR (101 MHz, acetone-d_6, bottom) for **3db**.

Synthesis of 3dc

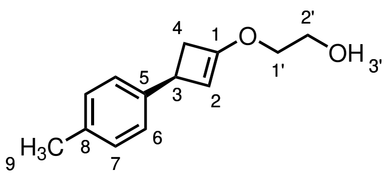

The corresponding compound was prepared following General Procedure E, using *p*-tolylboronic acid. Purification by manual flash chromatography (Pentane 80:20 Et_2O) afforded a colorless oil identified as **3dc** (71.1 mg, 87% yield). SFC analysis showed an enantiomeric excess of 86%.

^{1}H NMR (Acetone-d_6, 400 MHz): δ (ppm) 7.19 – 7.14 (m, 2H, C(Ar)-H), 7.10 – 7.06 (m, 2H, C(Ar)-H), 4.80 (d, J = 0.9 Hz, 1H, C(2)-H), 4.01 – 3.87 (m, 3H, C(1')-H, OH), 3.86 – 3.75 (m, 2H, C(2')-H), 3.58 – 3.52 (m, 1H, C(3)-H), 3.06 (dd, J = 12.7, 4.6 Hz, 1H, C(4)-H), 2.33 – 2.23 (m, 4H, C(4)-H, C(9)-H).

^{13}C NMR (Acetone-d_6, 101 MHz): δ (ppm) 153.4 (C(1)), 141.7 (C(5)), 135.3 (C(8)), 128.7 (C(7)), 126.3 (C(6)), 98.8 (C(2)), 70.0 (C(1')), 60.1 (C(2')), 40.9 (C(4)), 36.6 (C(3)), 20.1 (C(9)).

IR (neat): 3379 (br), 2926 (w), 1634 (s), 1514 (w), 1453 (w), 1307 (s), 1296 (s), 1217 (m), 1084 (m), 1045 (m), 989 (m), 893 (w), 816 (w), 778 (w) cm^{-1}.

HRMS (ESI): m/z calculated for $C_{13}H_{17}O_2^+$ $[M+H]^+$ = 205.1223; found = 205.1224.

SFC Chiralpak ® IB; 1500 psi, 30 °C; flow 1.5 mL/min; from 1% to 30% MeOH in 5 min; 86% ee (minor enantiomer t_R = 2.61 min; major enantiomer t_R = 2.72 min).

$[\alpha]_D^{25}$ = +25.7 (c = 1.03, CH_2Cl_2).

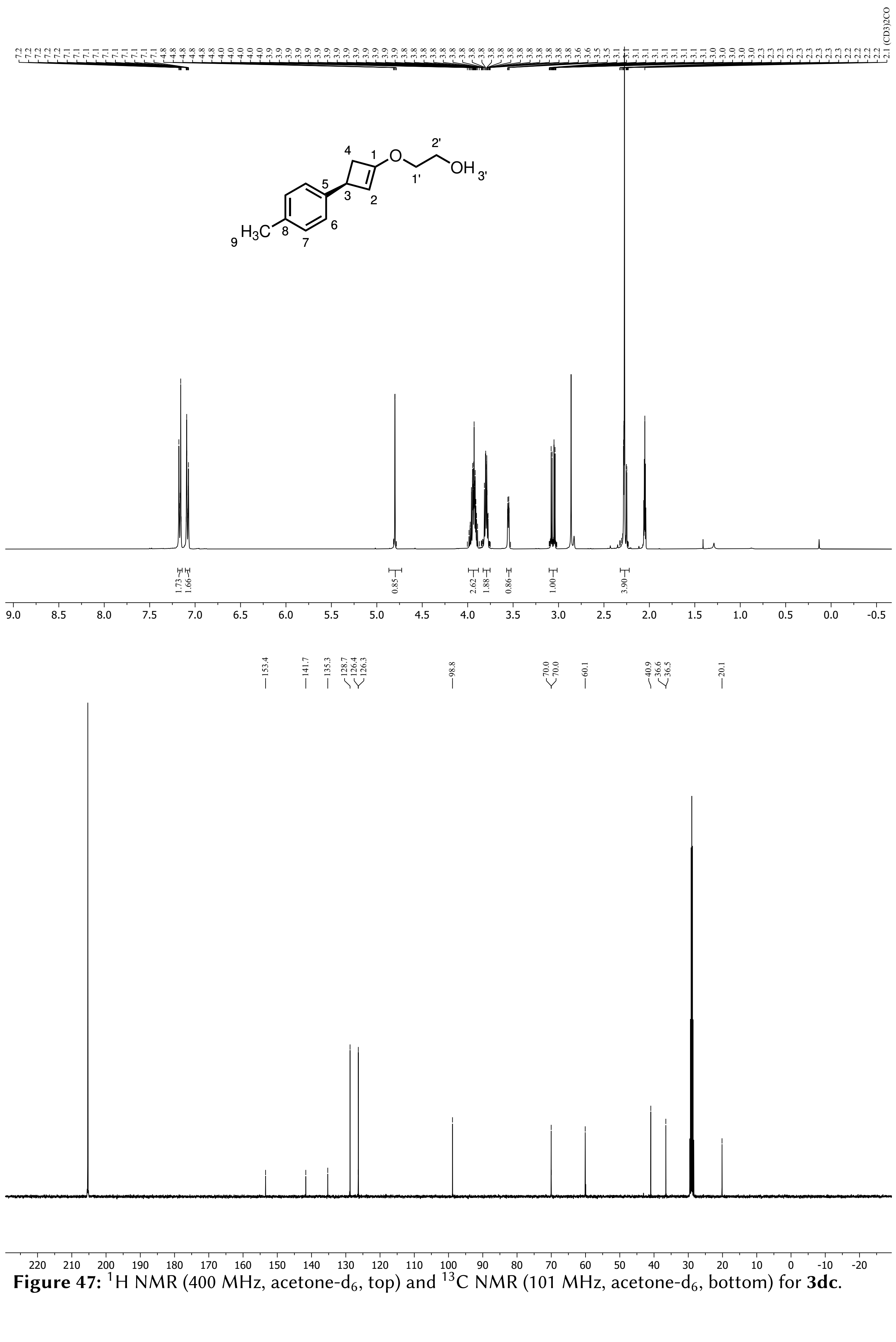

Figure 47: ^{1}H NMR (400 MHz, acetone-d_6, top) and ^{13}C NMR (101 MHz, acetone-d_6, bottom) for **3dc**.

4 Further transformations

Synthesis of 4ar

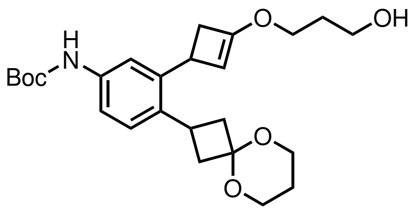

Isolated as a mixture with **3ar** (1.5 mg).

HRMS (ESI): m/z calculated for $C_{25}H_{36}NO_6^+$ $[M+H]^+$ = 446.2537; found = 446.2553. $C_{25}H_{35}NO_6Na^+$ $[M+Na]^+$ = 468.2357; found = 468.2376.

^{1}H NMR (Acetone-d_6, 400 MHz): As a mixture of approximately **4ar** 1.4:1 **3ar**.

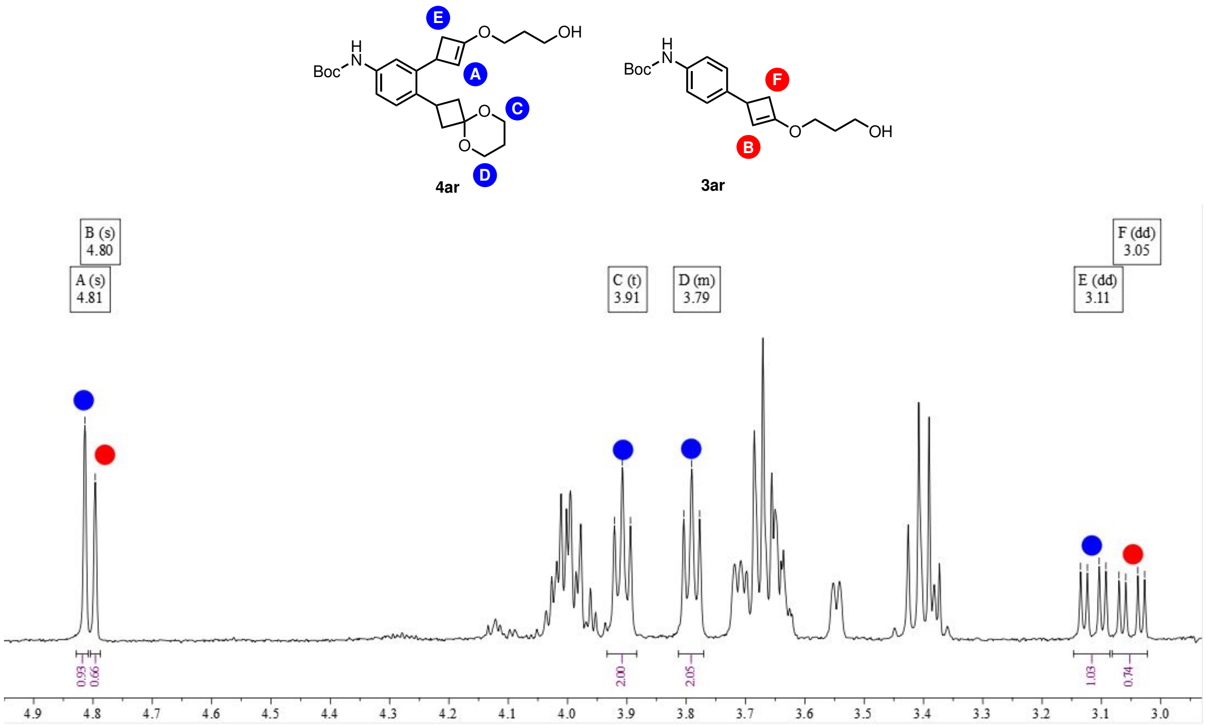

Figure 48: ^{1}H NMR (400 MHz, acetone-d_6, zoomed) for **4ar 1:1 3ar**.

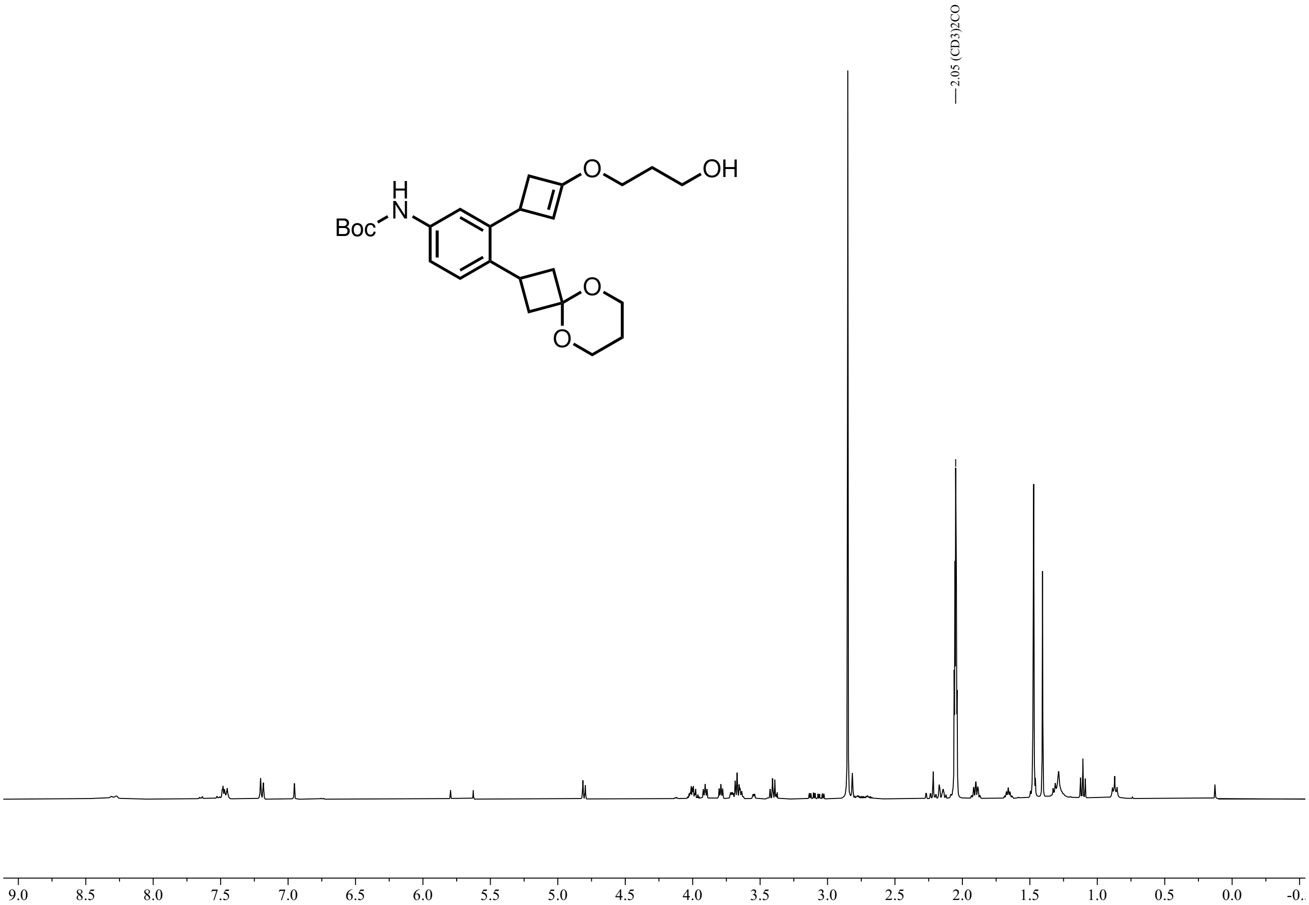

Figure 49: ^{1}H NMR (400 MHz, acetone-d$_6$, full) **4ar**.

Synthesis of 5

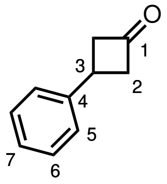

Compound **(±)-3aa** (81.7 mg, 0.4 mmol) was dissolved in a mixture of acetic acid and water (1.0 mL, 1:1 mixture) and stirred at room temperature overnight. The next day, Et_2O (1.0 mL) and a saturated solution of $NaHCO_3$ (2.0 mL) were added. The organic layer was washed with brine (1.0 mL) and water (1.0 mL), dried over Na_2SO_4, filtered and concentrated under reduced pressure. Purification by manual flash chromatography (pentane) afforded a colorless oil identified as **5** (49.7 mg, 85% yield).

^{1}H NMR ($CDCl_3$, 400 MHz): δ (ppm) 7.34 – 7.16 (m, 5H, C(Ar)-H), 3.61 (tt, J = 9.3, 7.5 Hz, 1H, C(3)-H), 3.49 – 3.37 (m, 2H, C(2)-H), 3.25 – 3.11 (m, 2H, C(2)-H).

^{13}C NMR ($CDCl_3$, 101 MHz): 207.0 (C(1)), 143.7 (C(4)), 128.9 (C(6)), 126.8 (C(7)), 126.6 (C(5)), 54.9 (C(2)), 28.6 (C(3)).

In agreement with previous literature.[3]

[3] *J. Org. Chem.* **2021**, 86, 95639586.

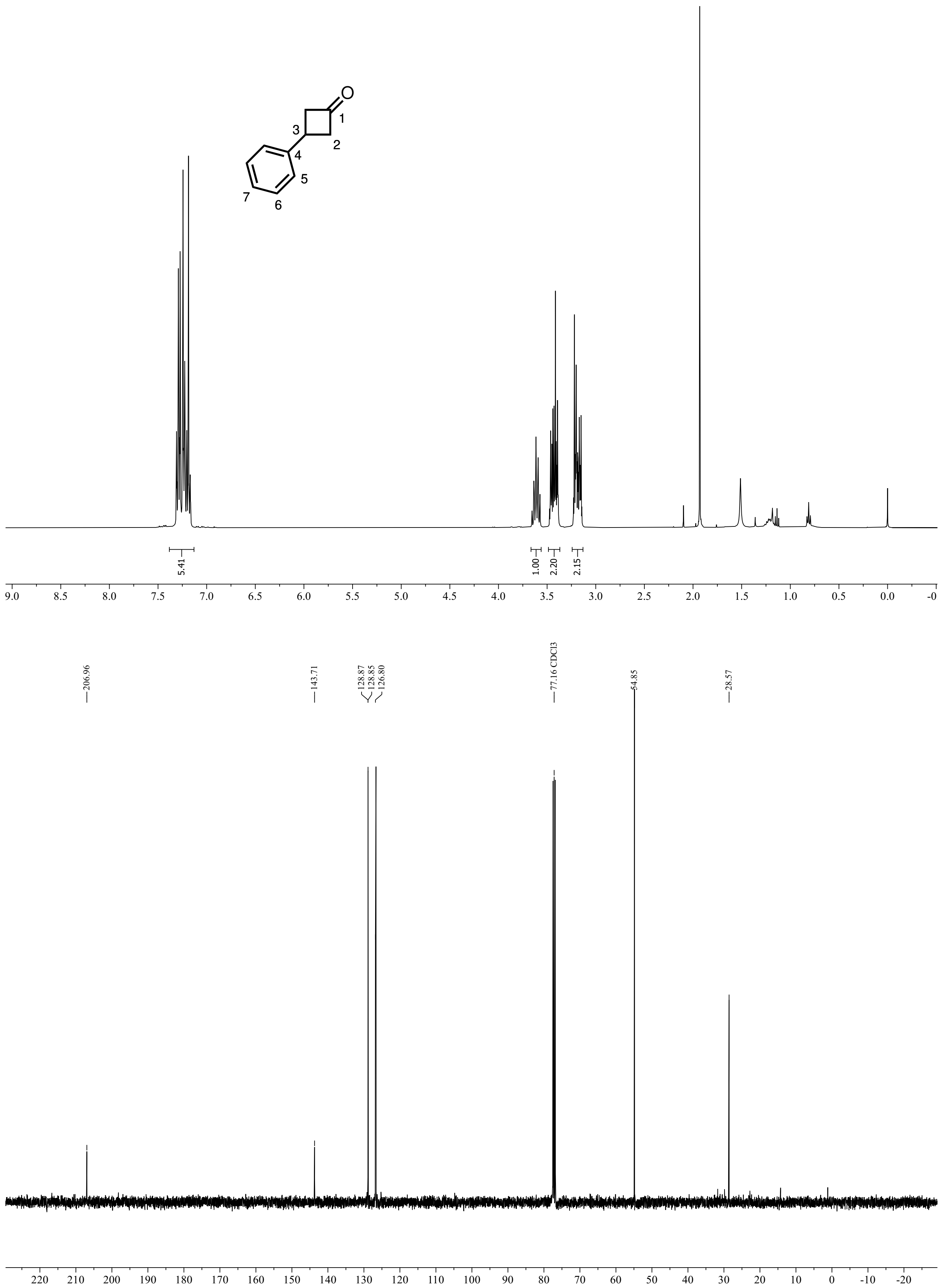

Figure 50: ^{1}H NMR (400 MHz, $CDCl_3$, top) and ^{13}C NMR (101 MHz, $CDCl_3$, bottom) for **5**.

Synthesis of 6

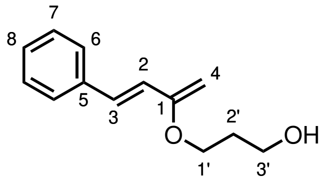

Compound **(±)-3aa** (81.7 mg, 0.4 mmol) was dissolved in dioxane (1.0 mL) under an argon atmosphere and was stirred at 100 °C for 16 h. Then, the solvent was removed under reduced pressure. Purification by manual flash chromatography (Pentane 80:20 Et_2O) afforded a colorless oil identified as **6** (62.9 mg, 77% yield).

^{1}H NMR (Acetone-d_6, 400 MHz): δ (ppm) 7.54 – 7.47 (m, 2H, C(Ar)-H), 7.39 – 7.31 (m, 2H, C(Ar)-H), 7.29 – 7.21 (m, 1H, C(Ar)-H), 6.94 (d, *J* = 15.9 Hz, 1H, C(2)-H), 6.70 (d, *J* = 16.0 Hz, 1H, C(3)-H), 4.39 – 4.27 (m, 2H, C(4)-H), 3.95 (t, *J* = 6.3 Hz, 2H, C(1')-H), 3.75 (td, *J* = 6.2, 5.2 Hz, 2H, C(3')-H), 1.97 (p, *J* = 6.3 Hz, 2H, C(2')-H).

^{13}C NMR (Acetone-d_6, 400 MHz): 159.4 (C(1)), 137.8 (C(5)), 129.5 (C(3)), 129.0 (C(6)), 128.5 (C(7)), 127.5 (C(8)), 126.1 (C(2)), 88.4 (C(4)), 65.1 (C(1')), 59.3 (C(3')), 33.2 (C(2')).

IR (neat): 3465 (br), 1176 (s), 1110 (s), 971 (m), 771.4 (s), 693 (s) cm $^{-1}$.

HRMS (ESI): m/z calculated for $C_{13}H_{17}O_2^+$ $[M+H]^+$ = 205.1223; found = 205.1230.

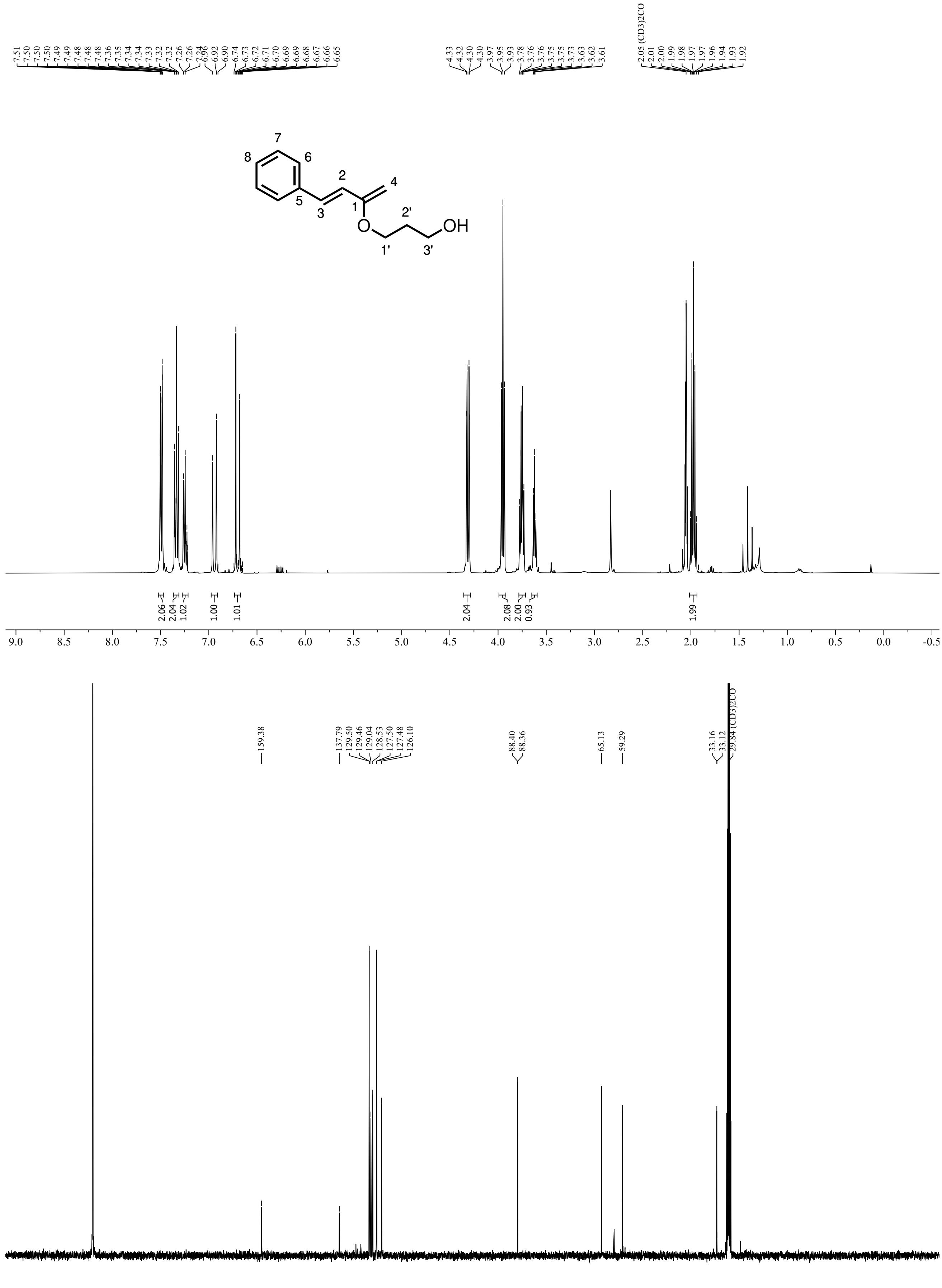

Figure 51: ^{1}H NMR (400 MHz, acetone-d$_6$, top) and ^{13}C NMR (101 MHz, acetone-d$_6$, bottom) for **6**.

Synthesis of 7

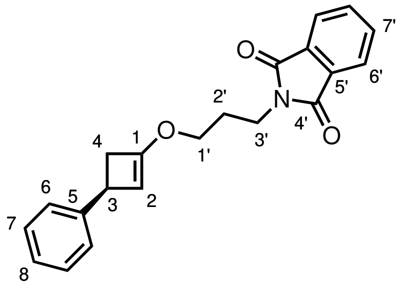

To a round bottom flask containing phthalimide (48.0 mg, 0.35 mmol, 1.3 equiv.) and triphenylphosphine (85.0 mg, 0.35 mmol, 1.3 equiv.), a solution of **(+)-3aa** (55.0 mg, 0.27 mmol, 1 equiv.) in THF (1.0 mL) was added at 0°C under an argon atmosphere. Then, diisopropyl azodicarboxylate (DIAD) (64 μL, 0.35 mmol, 1.3 equiv.) was added and the reaction was allowed to warm-up to room temperature.

After 1 h, Celite® (c.a. 1 g) was added and the reaction mixture was concentrated under reduced pressure. Purification by manual flash chromatography (Pentane 95:5 Et_2O) afforded a colorless oil identified as **7** (62.2 mg, 0.19 mmol, 75% yield).

^{1}H NMR (Acetone-d_6, 400 MHz): δ (ppm) 7.90 – 7.78 (m, 4H, C(6')-H, C(7')-H), 7.32 – 7.22 (m, 4H, C(6)-H, C(7)-H), 7.22 – 7.11 (m, 1H, C8)-H), 4.78 (d, J = 0.9 Hz, 1H, C(2)-H), 4.06 – 3.91 (m, 2H, C(3')-H), 3.83 (t, J = 6.8 Hz, 2H, C(1')-H), 3.55 (ddd, J = 4.5, 1.5, 0.8 Hz, 1H, C(3)-H), 2.97 (dd, J = 12.7, 4.6 Hz, 1H, C(4)-H), 2.17 (dd, J = 12.7, 1.5 Hz, 1H, C(4)-H), 2.14 – 2.08 (m, 2H, C(2')-H).

^{13}C NMR (Acetone-d_6, 101 MHz): δ (ppm) 168.8 (C(4')), 154.1 (C(1)), 145.6 (C(5')), 134.9 (C(7')), 133.2 (C(5)), 129.0 (C(7)), 127.3 (C(6)), 126.9 (C(8)), 123.7 (C(6')), 99.9 (C(2)), 67.0 (C(1')), 41.7 (C(3')), 37.8 (C(4)), 36.0 (C(3)), 28.4 (C(2')).

IR (neat): 2927 (m), 1772 (w), 1712 (s), 1632 (m), 1397 (m), 1302 (m), 721 (m), 701 (w) cm^{-1}.

HRMS (ESI): m/z calculated for $C_{21}H_{19}O_3Na^+$ $[M+Na]^+$ = 356.1257; found = 356.1259.

SFC Chiralpak ® IE; 1500 psi, 30 °C; flow 1.5 mL/min; from 1% to 50% MeOH in 10 min; 95% ee (minor enantiomer t_R = 7.52 min; major enantiomer t_R = 8.00 min).

$[\alpha]_D^{25}$ = +20.6 (c = 1.04, CH_2Cl_2).

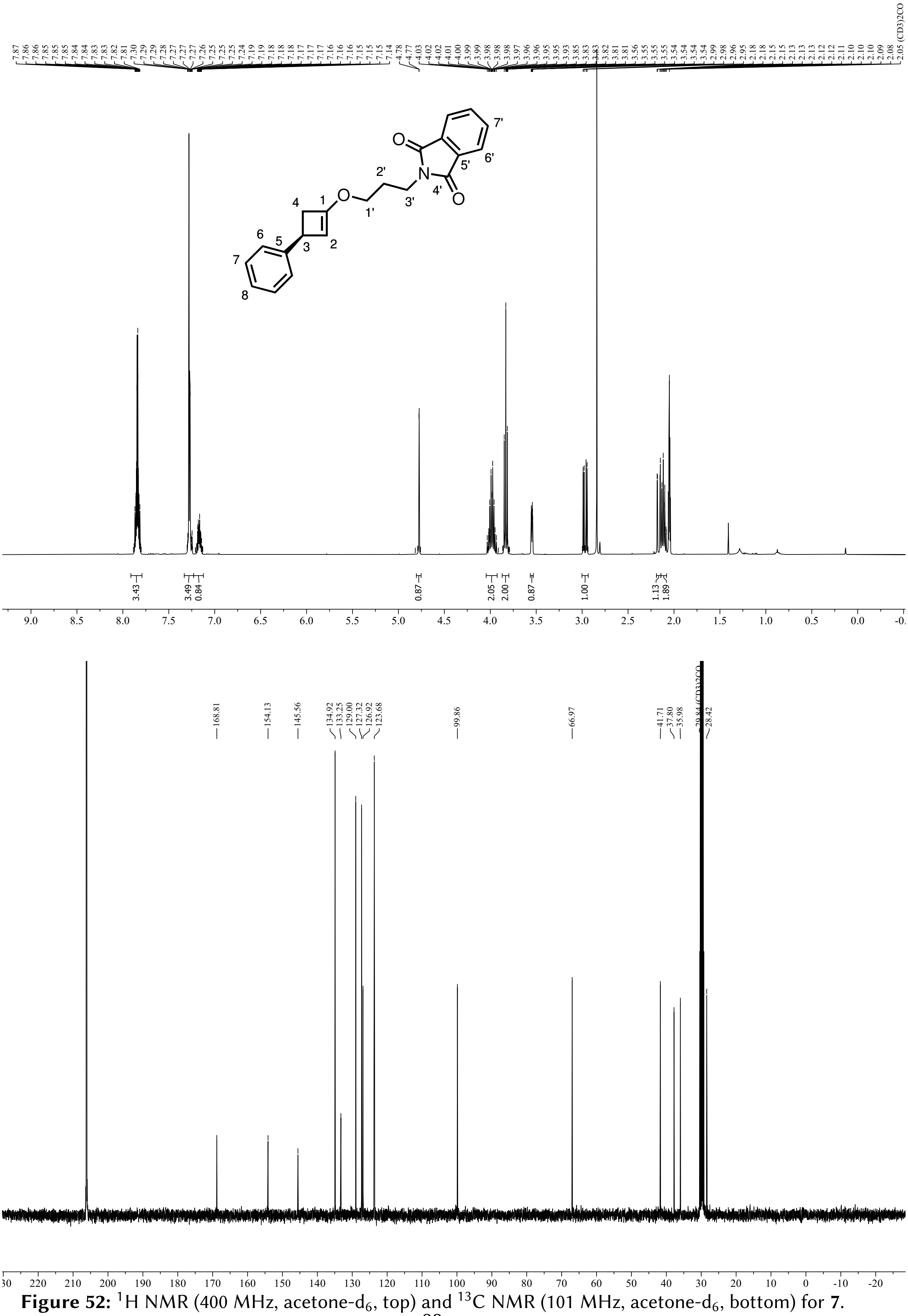

Figure 52: ^{1}H NMR (400 MHz, acetone-d_6, top) and ^{13}C NMR (101 MHz, acetone-d_6, bottom) for **7**.

Synthesis of 8

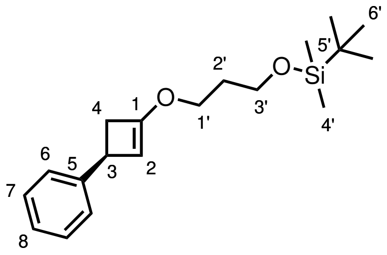

A solution of **(+)-3aa** (500.0 mg, 2.45 mmol, 1 equiv.) in DCM (10 mL) was added to a round bottom flask containing TBDMSCl (480.7 mg, 3.19 mmol, 1.3 equiv.) and imidazole (333.7 mg, 4.9 mmol, 2.0 equiv.). After stirring at room temperature for 1 h, Celite® (c.a. 3.0 g) was added and the solvent was removed under reduced pressure. Purification by manual flash column chromatography (Pentane 99.5:0.5 Et_2O) afforded a colorless oil identified as **8** (663.3 mg, 2.08 mmol, 85% yield).

^{1}H NMR (Acetone-d_6, 400 MHz): δ (ppm) 7.31 – 7.13 (m, 5H, C(Ar)-H), 4.82 (d, J = 0.9 Hz, 1H, C(2)-H), 3.98 (qt, J = 9.9, 6.2 Hz, 2H, C(1')-H), 3.77 (t, J = 6.0 Hz, 2H, C(3')-H), 3.59 (dt, J = 4.6, 1.2 Hz, 1H, C(3)-H), 3.08 (dd, J = 12.7, 4.6 Hz, 1H, C(4)-H), 2.39 (dd, J = 12.8, 1.6 Hz, 1H, C(4)-H), 1.90 (p, J = 6.2 Hz, 2H, C(2')-H), 0.91 (s, 9H, C(6')-H), 0.08 (d, J = 0.8 Hz, 6H, C(4')-H).

^{13}C NMR (Acetone-d_6, 101 MHz): δ (ppm) 154.4 (C(1)), 145.7 (C(5)) , 129.0 (C(7)), 127.3 (C(6)), 126.9 (C(8)), 99.5 (C(2)), 65.6 (C(1' or 3')), 60.0 (C(1' or 3')), 41.8 (C(4)), 37.8 (C(3)), 32.8 (C(2')), 26.3 (C(6')), 18.8 (C(5')), -5.2 (C(4')).

IR (neat): 2955 (w), 1790 (m), 1256 (m), 1096 (m), 836 (s), 776 (m), 699 (w) cm^{-1}.

HRMS (ESI): m/z calculated for $C_{19}H_{31}O_2Si^+$ $[M+H]^+$ = 319.2088; found = 319.2087.

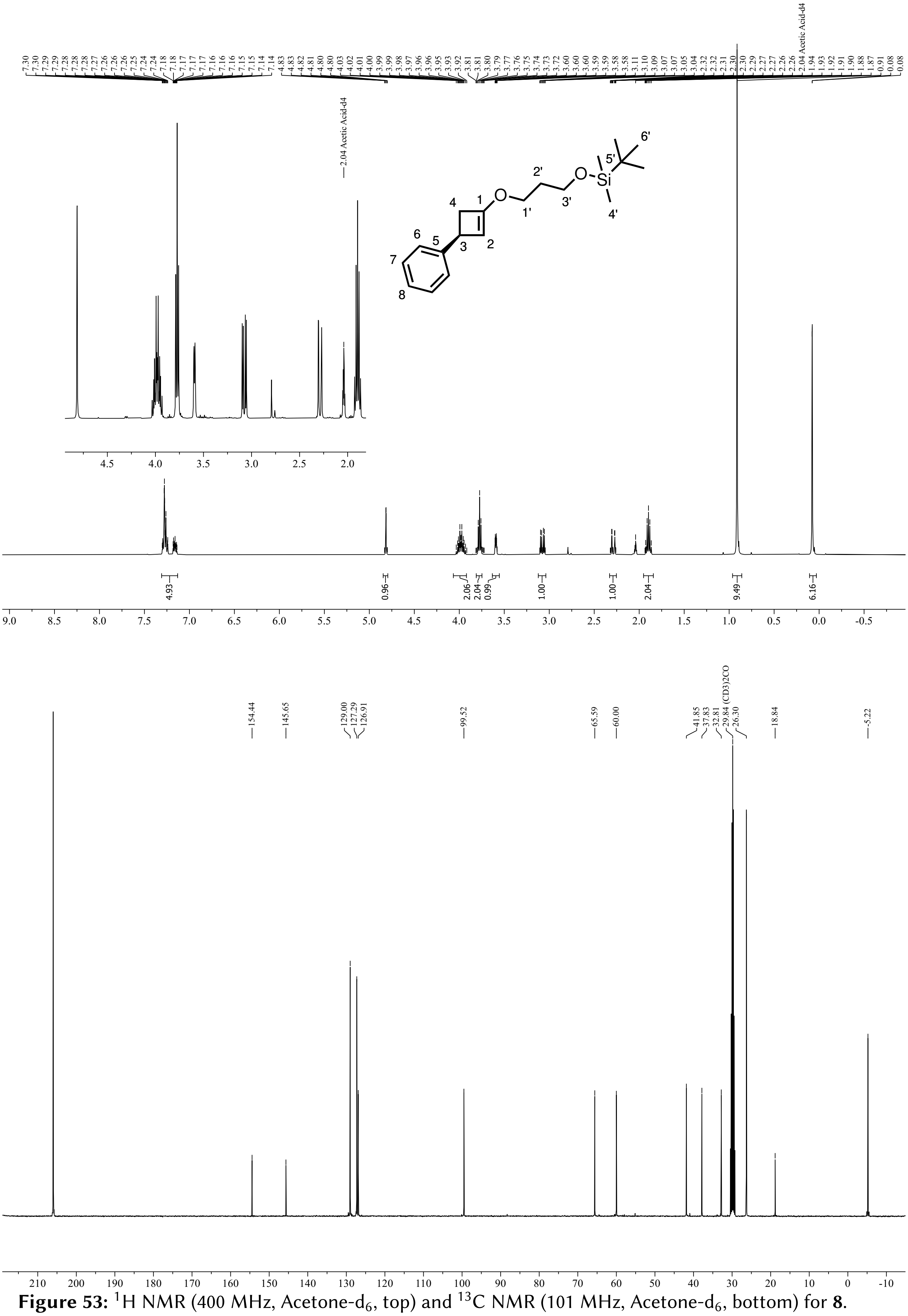

Figure 53: ^{1}H NMR (400 MHz, Acetone-d_6, top) and ^{13}C NMR (101 MHz, Acetone-d_6, bottom) for **8**.

Synthesis of 9

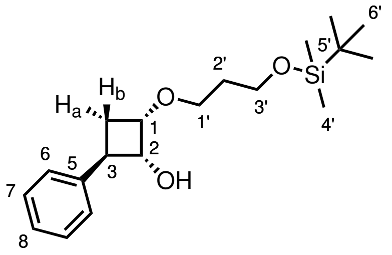

9-BBN (0.5 M in THF, 3 mL, 1.5 mmol, 3 equiv.) was added to a round-bottom flask containing **8** (161.0 mg, 0.5 mmol, 1 equiv.) under an argon atmosphere. After stirring for 1 h at room temperature, the reaction mixture was cooled down to 0 °C and NaOH (1 M aqueous solution, 2.75 mL) and H_2O_2 (30% (w/w) in H_2O, 1.5 mL) were added under air. The mixture was allowed to reach room temperature.

After stirring for 1 h, the excess peroxide is neutralized carefully with a saturated solution of $Na_2S_2O_3$ (c.a. 2 mL) at 0 °C under air. Then, the aqueous layer was extracted 3 times with ethyl acetate (c.a. 3x2 mL). The combined organic layers were dried over anhydrous Na_2SO_4, filtered, concentrated under reduced pressure and purified by manual flash chromatography (Hexane 90:10 Ethyl Acetate) to afford a colorless oil identified as **9** (153.8 mg, 0.46 mmol, 91% yield).

NOTE: Analysis of relative configuration of compound **9** can be found in section 5.1 of this document.

^{1}H NMR ($CDCl_3$, 400 MHz): δ (ppm) 7.36 – 7.18 (m, 5H C(Ar)-H), 4.03 (ddd, J = 8.2, 6.1, 1.0 Hz, 1H C(2)-H), 3.81 – 3.69 (m, 3H, C(1)-H, C(3')-H), 3.62 (t, J = 6.4 Hz, 2H, C(1')-H), 2.78 (dt, J = 10.7, 8.4 Hz, 1H, C(3)-H), 2.48 (dddd, J = 10.8, 8.7, 7.6, 1.0 Hz, 1H, C(4a)-H), 1.80 (p, J = 6.2 Hz, 2H, C(2')-H), 1.56 (td, J = 10.7, 8.4 Hz, 1H, C(4b)-H), 0.90 (s, 9H, C(6')-H), 0.06 (s, 6H, C(4')-H).

^{13}C NMR ($CDCl_3$, 101 MHz): δ (ppm) 141.9 (C(5)), 128.6 (C(7)), 127.1 (C(6)), 126.6 (C(8)), 80.3 (C(1)), 78.5 (C(2)), 65.8 (C(1')), 60.0 (C(3')), 41.0 (C(3)), 33.2 (C(2')), 27.5 (C(4)), 26.1 (C(5')), 18.5 (C(6')), -5.2 (C(4')).

IR (neat): 3395 (br), 2954 (w), 2929 (w), 2858 (w), 1256 (m), 1098 (s), 836 (s), 776 (m), 751 (m), 662 (m) cm^{-1}.

HRMS (ESI): m/z calculated for $C_{19}H_{33}O_3Si^+$ $[M+H]^+$ = 337.2194; found = 337.2195.

SFC Chiralpak ® ID; 1500 psi, 30 °C; flow 1.5 mL/min; from 1% to 30% MeOH in 5 min; 95% ee (minor enantiomer t_R = 2.22 min; major enantiomer t_R = 2.29 min).

$[\alpha]_D^{25}$ = +10.5 (c = 2.17, CH_2Cl_2).

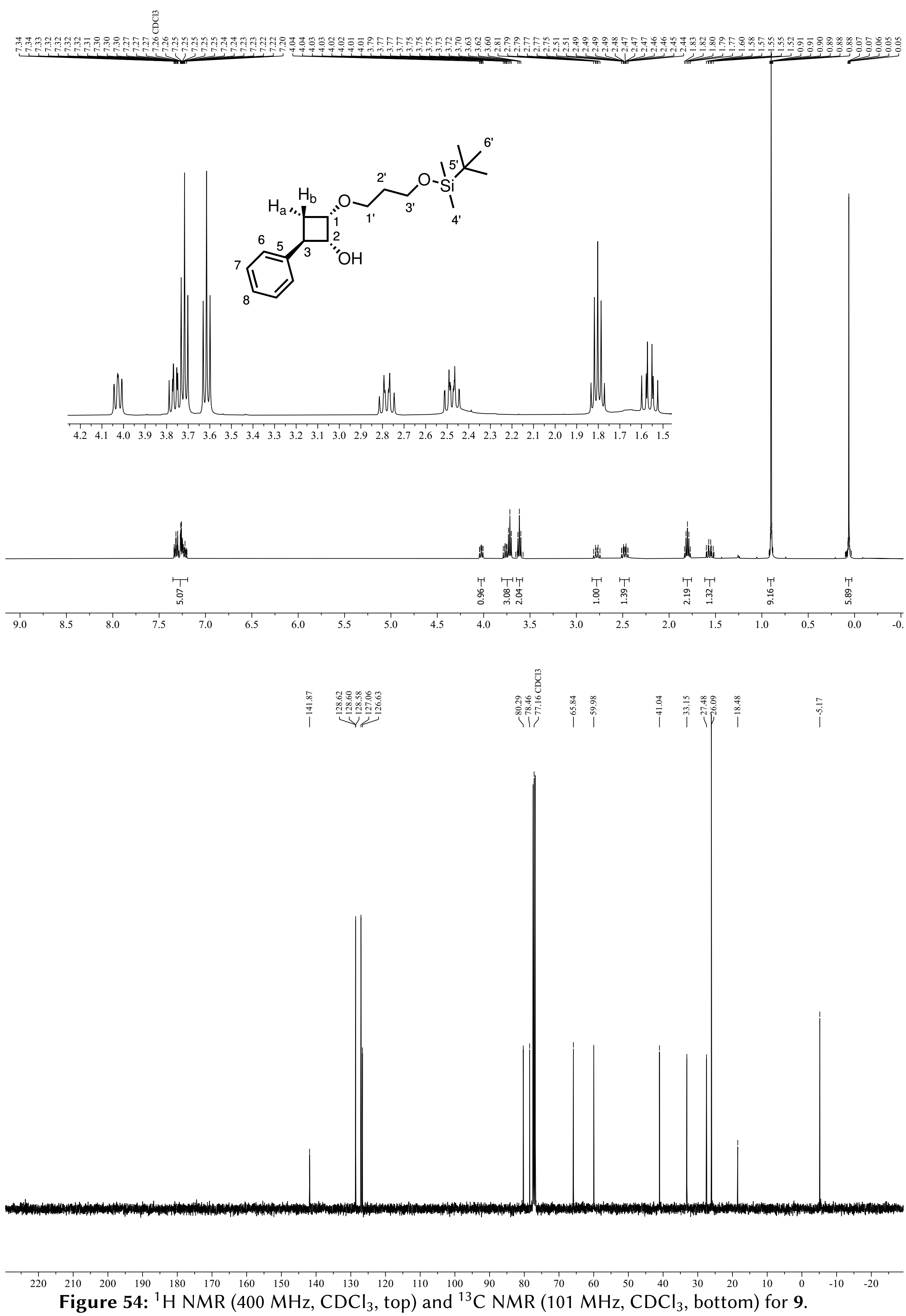

Figure 54: ^{1}H NMR (400 MHz, $CDCl_3$, top) and ^{13}C NMR (101 MHz, $CDCl_3$, bottom) for **9**.

Synthesis of 10

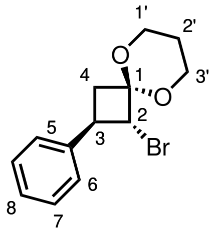

A solution of **(+)-3aa** (204.0 mg, 1 mmol, 1 equiv.) in DCM (5 mL) was added to a dispersion of *N*-Bromosuccinimide (204.7 mg, 1.15 mmol, 1.15 equiv.) in DCM (5 mL) at 0 °C under an argon atmosphere. After stirring for 30 min, the reaction mixture was washed with a saturated solution of $NaHCO_3$ (c.a. 10 mL) and brine (c.a. 10 mL). The solvent was removed under reduced pressure and the mixture was purified by manual flash chromatography (Hexane/Ethyl Acetate/Acetone 97.5:2:0.5) to afford a colorless oil identified as **10** (165.0 mg, 0.58 mmol, 58% yield).

^{1}H NMR (Acetone-d_6, 400 MHz): δ (ppm) 7.37 – 7.21 (m, 5H, C(Ar)-H), 4.34 (dd, J = 9.4, 0.7 Hz, 1H, C(2)-H), 4.18 – 4.02 (m, 2H, C(1' or 3')-H), 3.93 – 3.85 (m, 2H, C(1' or 3')-H), 3.44 – 3.33 (m, 1H, C(3)-H), 3.11 (dd, J = 11.4, 9.8 Hz, 1H, C(4)-H), 2.14 (ddd, J = 11.5, 9.8, 0.7 Hz, 1H, C(4)-H), 2.03 – 1.91 (m, 1H, C(2')-H), 1.52 (dp, J = 13.3, 3.0 Hz, 1H, C(2')-H).

^{13}C NMR (Acetone-d_6, 101 MHz): δ (ppm) 141.5 (C(5)), 129.4 (C(7)), 127.8 (C(6)), 127.5 (C(8)), 98.3 (C(1)), 62.4 (C(3' or 1')), 62.3 (C(3' or 1')), 55.2 (C(2)), 43.9 (C(4)), 37.3 (C(3)), 26.2 (C(2')).

IR (neat): 2970 (w), 2866 (w), 1276 (s), 1155 (m), 1043 (m), 764 (m), 711 (m), 699 (m) cm^{-1}.

HRMS (ESI): m/z calculated for $C_{13}H_{16}O_2[^{79}Br]^+$ $[M+H]^+$ = 283.0328; found = 283.0328; m/z calculated for $C_{13}H_{16}O_2[^{81}Br]^+$ $[M+H]^+$ = 285.0308; found = 285.0307.

SFC Chiralpak ® ID; 1500 psi, 30 °C; flow 1.5 mL/min; from 1% to 30% MeOH in 5 min; 95% ee (minor enantiomer t_R = 4.03 min; major enantiomer t_R = 4.17 min).

$[\alpha]_D^{25}$ = +72.9 (c = 1.02, CH_2Cl_2).

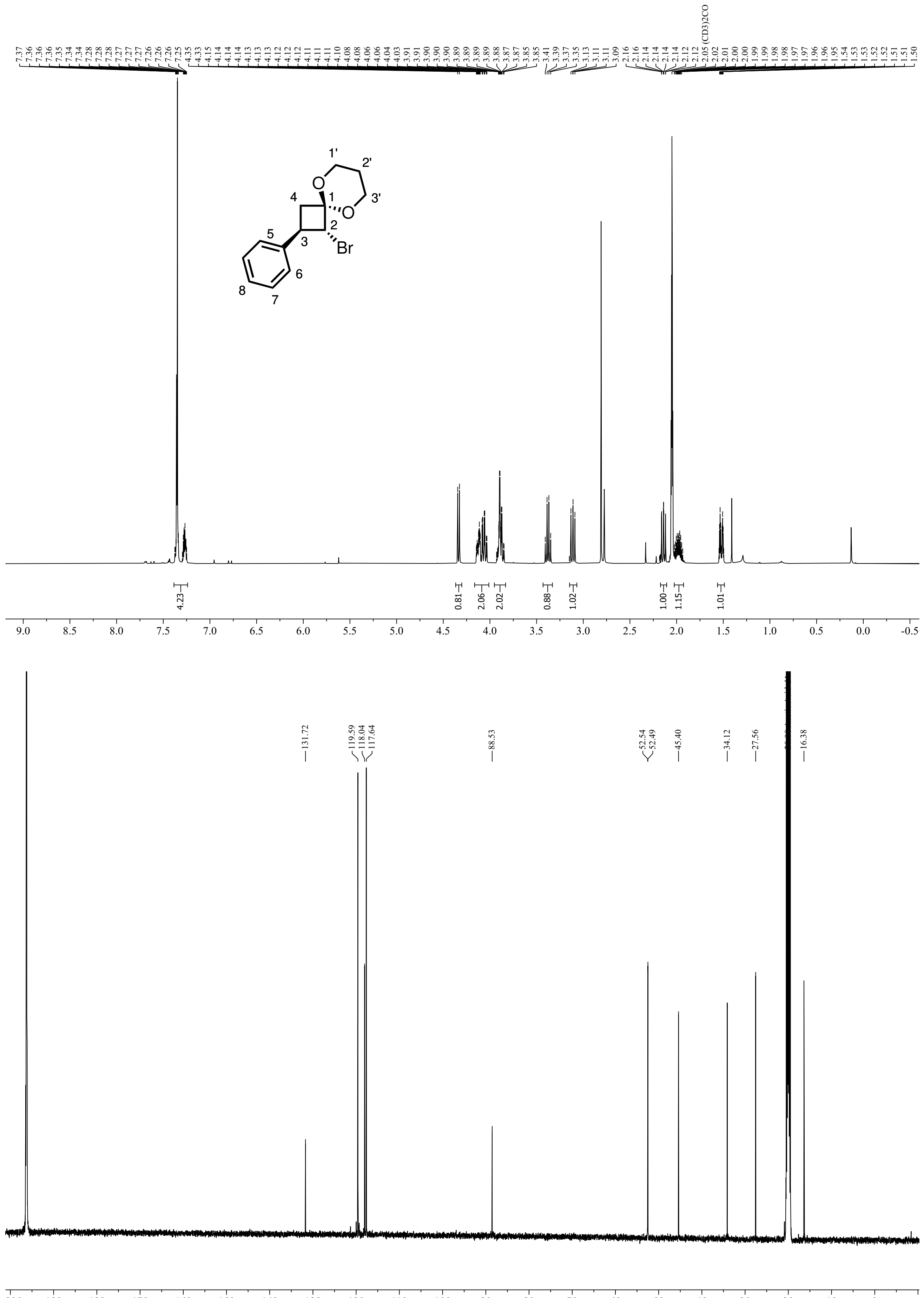

Figure 55: ^{1}H NMR (500 MHz, Acetone-d_6, top) and ^{13}C NMR (126 MHz, Acetone-d_6, bottom) for **8**.

Synthesis of 9-(*S*)-Mosher ester

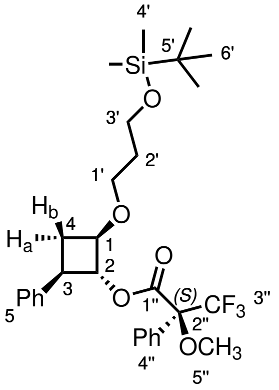

≡

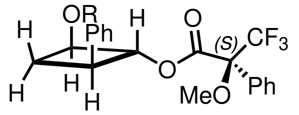

9-(*S*)-Mosher ester

DCC (42.0 mg, 0.18 mmol, 3.1 equiv) and DMAP (25.0 mg, 0.18 mmol, 3.1 equiv) were added to a solution containing **9** (20.0 mg, 0.06 mmol, 1 equiv) and (*S*)-(–)-MTPA (42.0 mg, 0.18 mmol, 3.1 equiv) in DCM (1.0 mL, [**9**]=0.06 M) under an argon atmosphere. After stirring 5 h at room temperature, Celite® (c.a. 1 g) was added and the solvent was removed under reduced pressure. Purification by manual flash chromatography (hexanes 98:2 ethyl acetate) afforded a colorless oil identified as **9-(*S*)-Mosher ester** (18.5 mg, 0.046 mmol, 79% yield).

^{1}H NMR ($CDCl_3$, 500 MHz): δ (ppm) 7.44 – 7.40 (m, 2H, (C($4''$Ar)-H), 7.37 – 7.25 (m, 5H, 3xC($4''$Ar)-H + 2xC(^{5})-H), 7.20 – 7.16 (m, 3H, 3xC(^{5}Ar)-H), 5.31 (ddd, J = 8.4, 6.0, 1.1 Hz, 1H, C(2)-H), 3.86 (td, J = 8.3, 6.0 Hz, 1H, C(1)-H), 3.67 – 3.59 (m, 2H, C(3')-H), 3.48 (dt, J = 9.2, 6.2 Hz, 1H, 1xC(1')-H), 3.45 (q, J = 1.2 Hz, 3H, C(5")-H), 3.36 (dt, J = 9.3, 6.4 Hz, 1H, 1xC(1')-H), 3.03 (dt, J = 10.8, 8.7 Hz, 1H, C(3)-H), 2.55 (dddd, J = 11.0, 9.2, 8.1, 1.0 Hz, 1H, C(4)-H_a), 1.76 – 1.67 (m, 3H, C(4)-H_b + C(2')-H), 0.84 (s, 9H, C(6')-H), 0.00 (s, 6H, C(4')-H).

^{13}C NMR ($CDCl_3$, 101 MHz): δ (ppm) 165.4 (C(1")), 140.1 (C(^{5}Ar)), 132.3 (C(Ar)), 129.8 (C(Ar)), 128.8 (C(Ar)), 128.6 (C(Ar)), 127.4 (C(Ar)), 127.1 (C(Ar)), 127.0 (C(Ar)), 80.5 (C(2)), 75.4 (C(1)), 65.9 (C(1')), 59.8 (C(3')), 55.6 (C(5")), 38.6 (C(3)), 33.0 (C(2')), 28.5 (C(4)), 26.1 (C(6')), 18.5 (C(5')), -5.2 (C(4')), -5.2 (C(4')).

^{19}F NMR ($CDCl_3$, 472 MHz): δ (ppm) -71.43 (CF_3).

IR (neat): 1743 (m), 1274 (m), 1190 (s), 710 (w) cm^{-1}

HRMS (ESI): m/z calculated for $C_{29}H_{40}F_3O_5Si^+$ $[M+H]^+$ = 553.2592; found = 553.2608.

$[\alpha]_D^{25}$ = -42.9 (c = 0.45, CH_2Cl_2).

HSQC NMR ($CDCl_3$, 500 MHz):

Signal	Assignment	f1 (ppm) (13C)	f2 (ppm) (1H)
**A**	C(2)-H	80.5	5.31
**B**	C(1)-H	75.5	3.85
**C**	C(3')-H_2	59.9	3.63
**D**	C(1')-H	66.0	3.50
**E**	C(5")-H_3	55.5	3.45
**F**	C(1')-H	65.8	3.36
**G**	C(3)-H	38.6	3.03
**H**	C(4)-H_a	28.5	2.55
**I**	C(4)-H_b	28.5	1.71
**J**	C(2')-H_2	33.1	1.71
**K**	C(6')-H_3	26.2	0.85

HMBC NMR ($CDCl_3$, 500 MHz):

Signal	Interaction Assignment	f1 (ppm) (13C)	f2 (ppm) (1H)	Signal	Interaction Assignment	f1 (ppm) (13C)	f2 (ppm) (1H)
**A**	C(CF_3)-H(Ar)	84.6	7.43	**L**	C(2')-1xH(1')	32.9	3.48
**B**	C(3)-H(Ar)	38.4	7.18	**M**	C(3')-1xH(1')	59.7	3.49
**C**	C(3)-H(2)	38.5	5.31	**N**	C(1)-1xH(1')	75.2	3.49
**D**	C(1)-H(2)	75.4	5.30	**O**	C(CF_3)-H(5")	84.6	3.45
**E**	C(Ar)-H(2)	140.0	5.31	**P**	C(2')-1xH(1')	33.0	3.36
**F**	C(CO)-H(2)	165.3	5.31	**Q**	C(3')-1xH(1')	59.7	3.36
**G**	C(4)-H(1)	28.3	3.86	**R**	C(1)-1xH(1')	75.2	3.36
**H**	C(1')-H(1)	65.8	3.86	**S**	C(4)-H(3)	28.3	3.04
**I**	C(2)-H(1)	80.3	3.86	**T**	C(2)-H(3)	80.3	3.04
**J**	C(2')-H(3')	32.8	3.63	**U**	C(Ar)-H(3)	126.8	3.03
**K**	C(1')-H(3')	65.8	3.63	**V**	C(Ar)-H(3)	139.9	3.03
				**W**	C(2)-H_a(4)	80.3	2.55

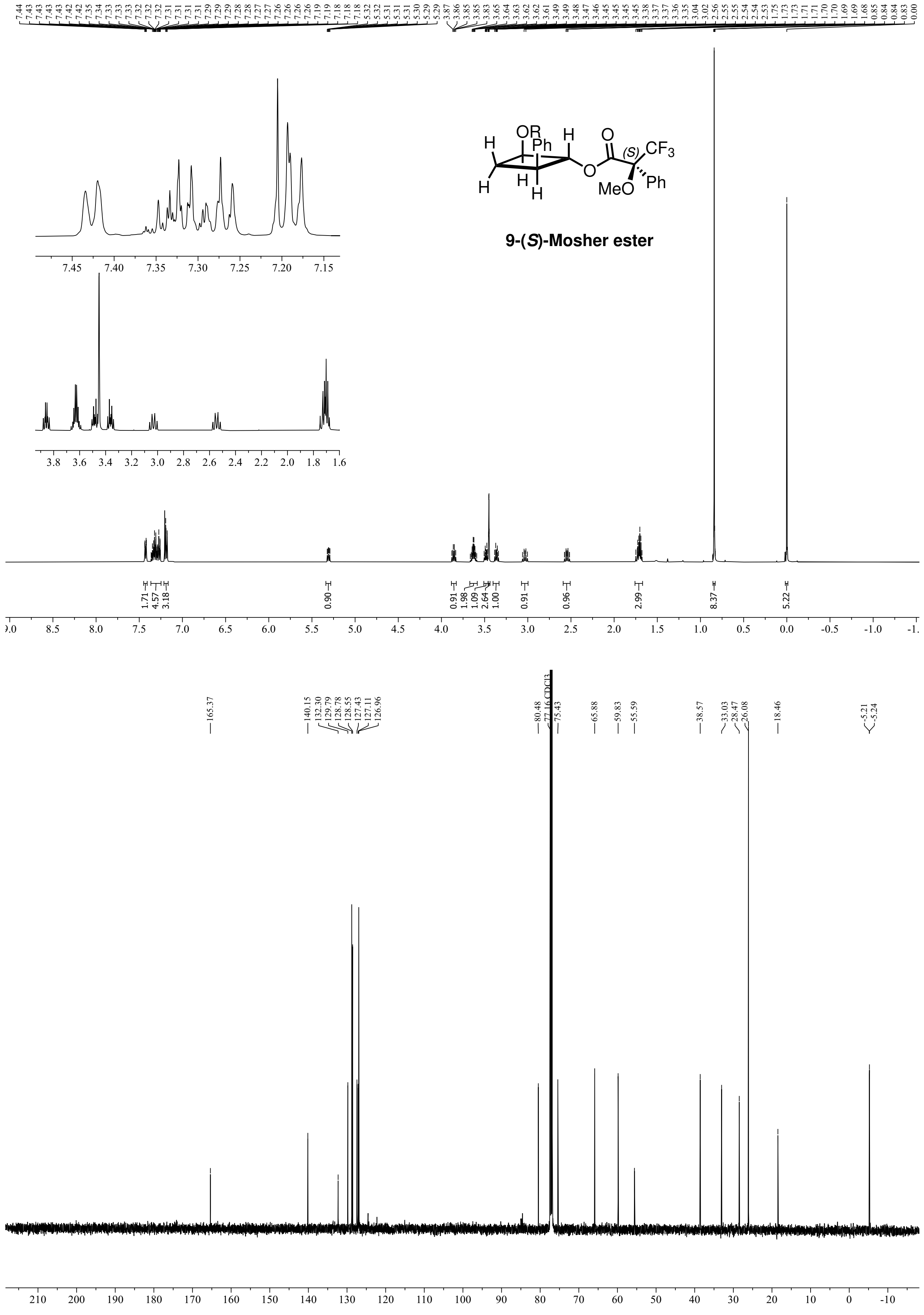

Figure 56: ^{1}H NMR (500 MHz, $CDCl_3$, top) and ^{13}C NMR (126 MHz, $CDCl_3$, bottom) for **9-(*S*)-Mosher ester**.

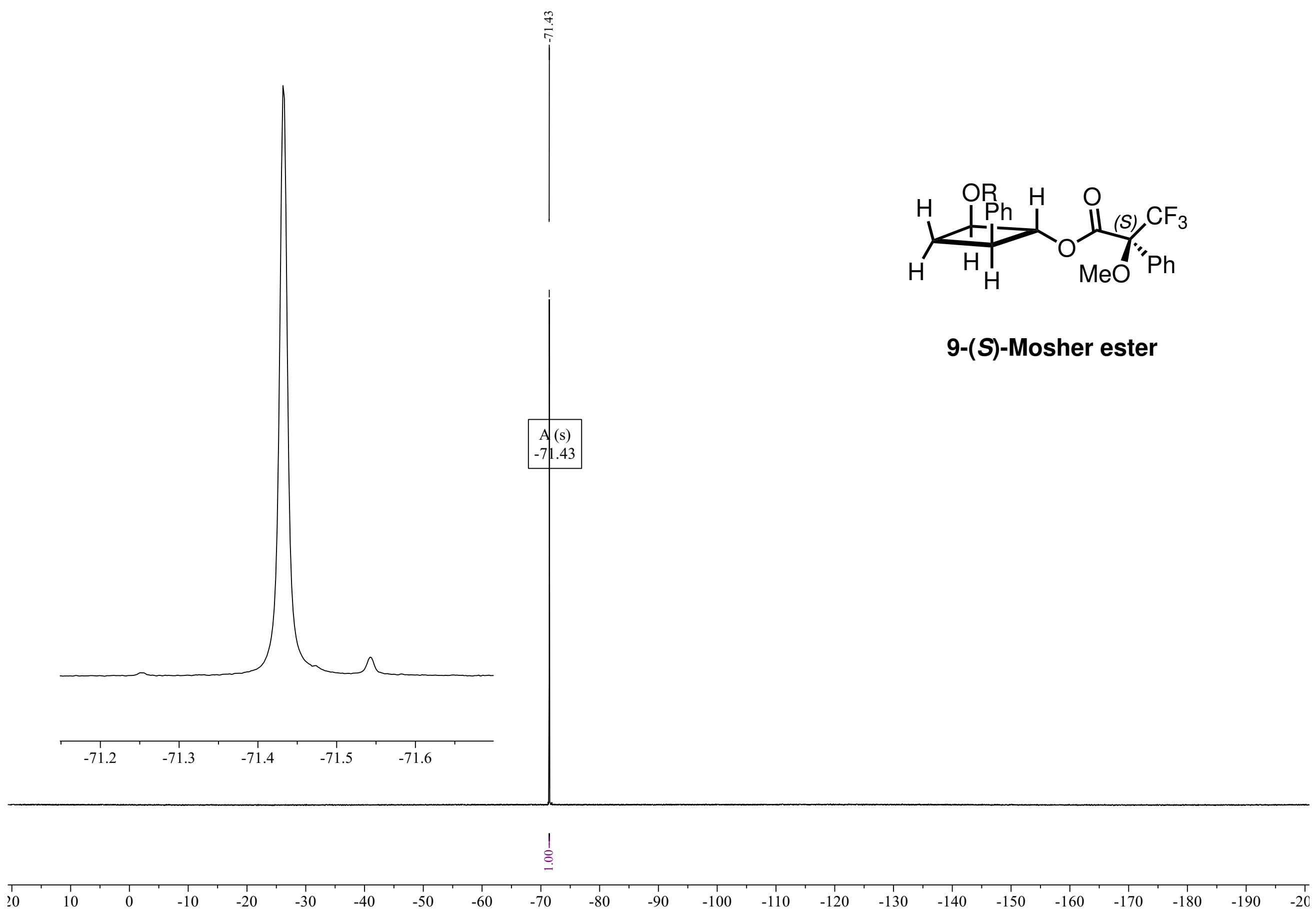

Figure 57: ^{19}F NMR for compound **9-(*S*)-Mosher ester**.

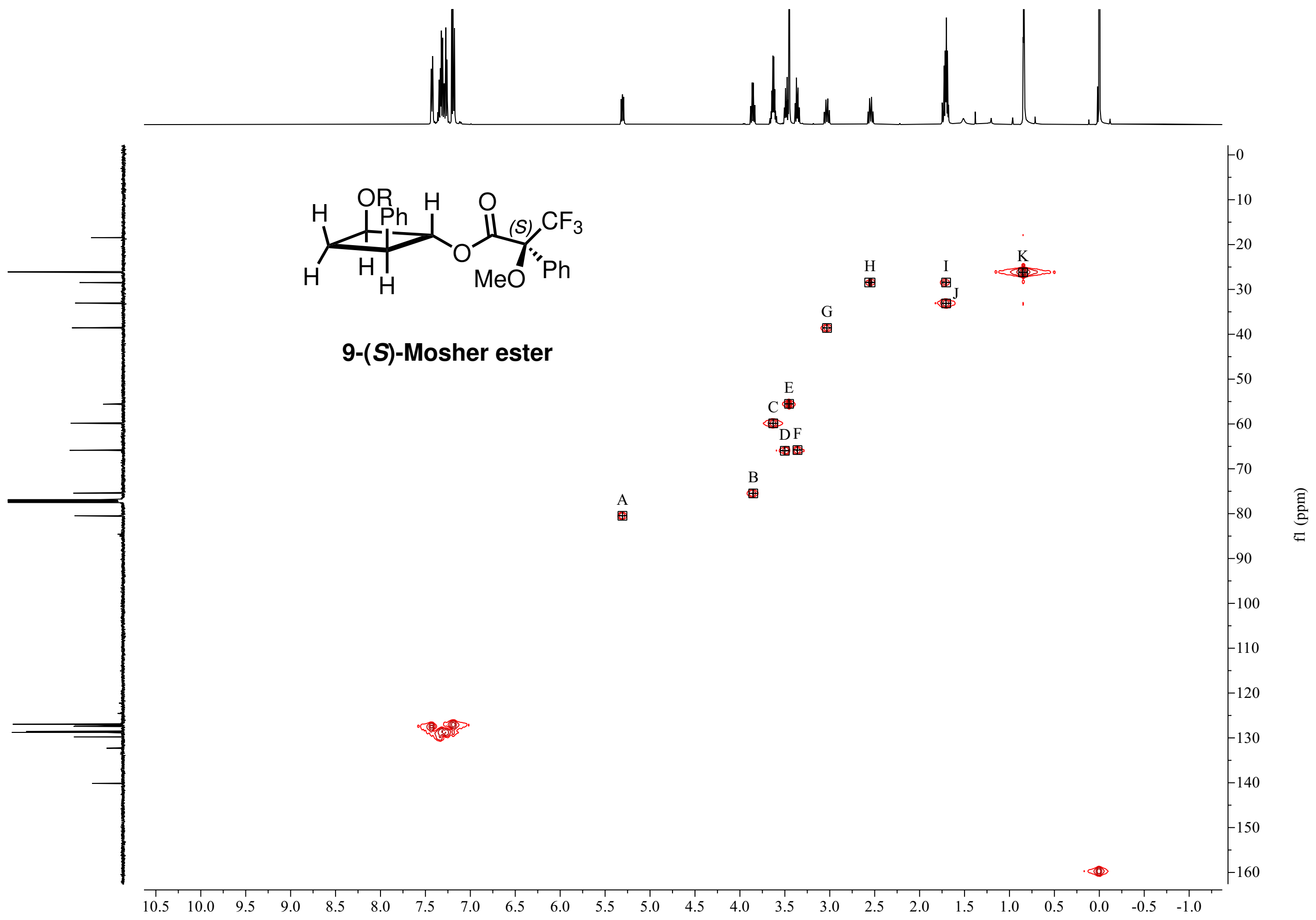

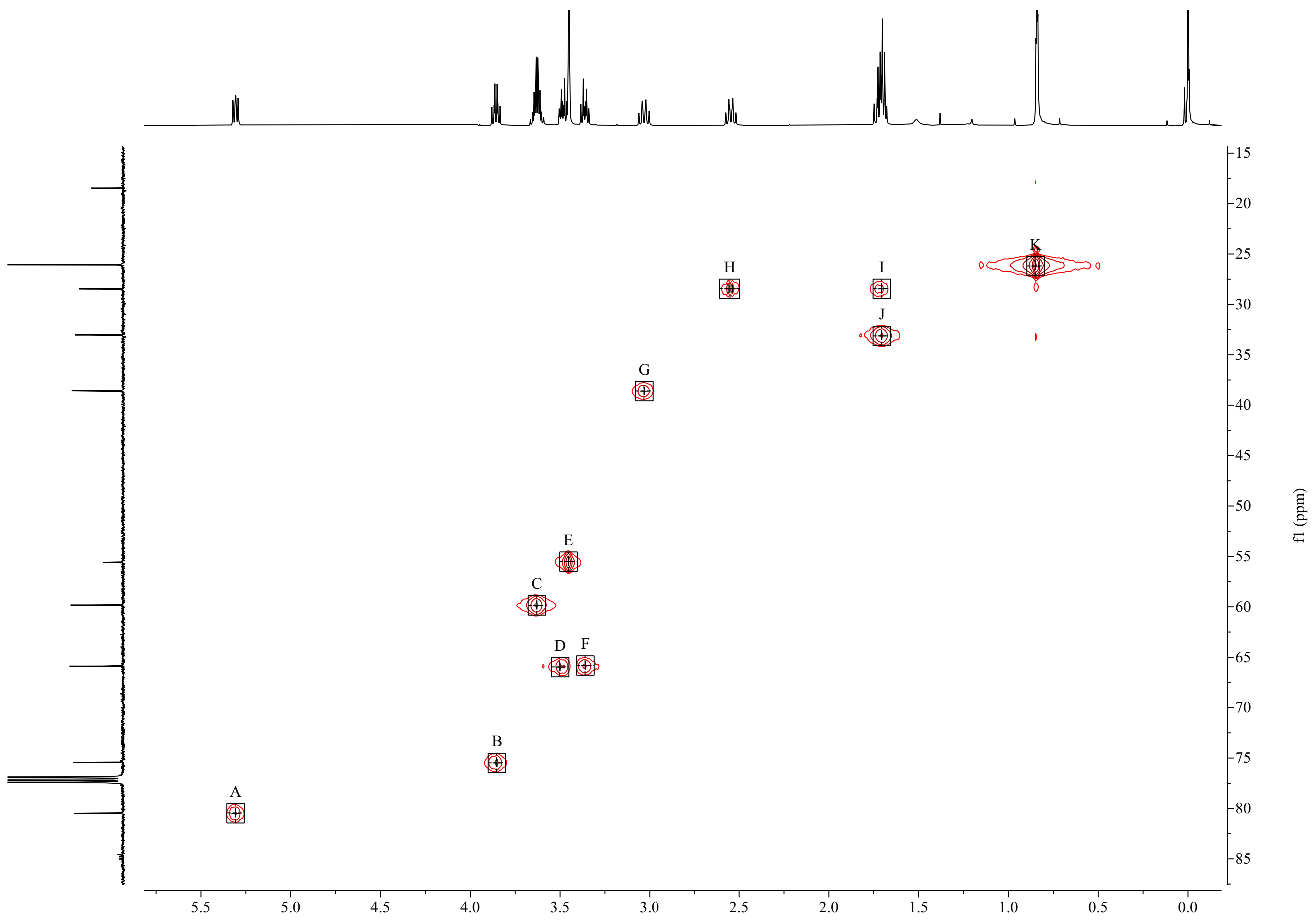

Figure 58: HSQC NMR (500 MHz, $CDCl_3$, full, top) and HSQC NMR (500 MHz, $CDCl_3$, zoomed, bottom) for **9-(*S*)-Mosher ester**.

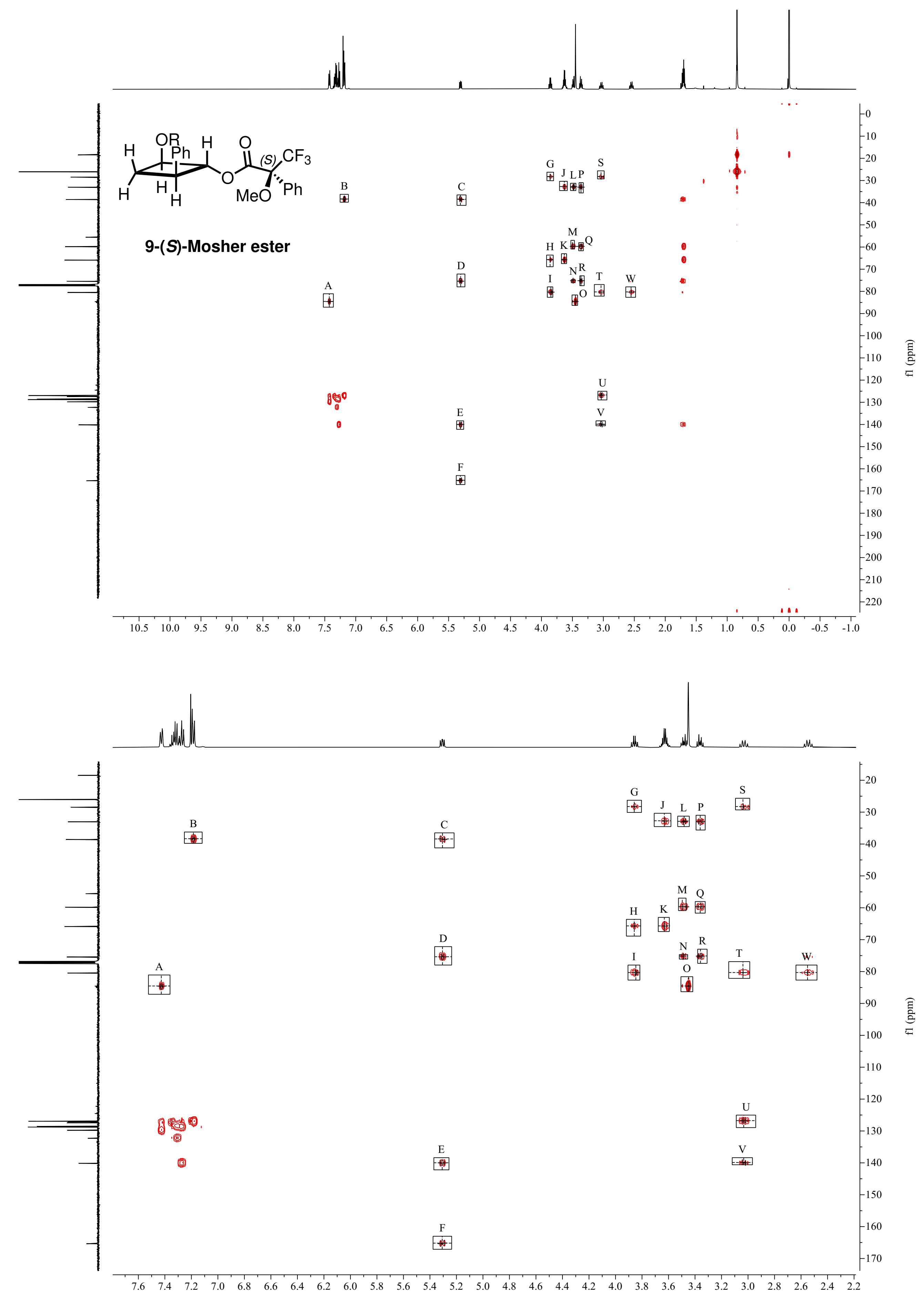

Figure 59: HMBC NMR (500 MHz, $CDCl_3$, full, top) and HMBC NMR (500 MHz, $CDCl_3$, zoomed, bottom) for **9-(*S*)-Mosher ester**.

Synthesis of 9-(*R*)-Mosher ester

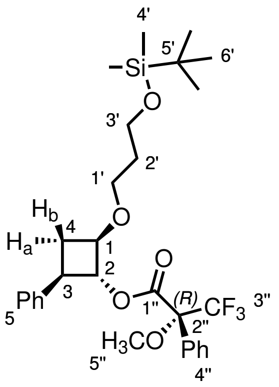

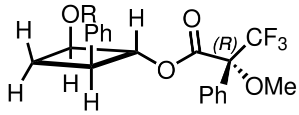

9-(*R*)-Mosher ester

DCC (42.0 mg, 0.18 mmol, 3.1 equiv) and DMAP (25.0 mg, 0.18 mmol, 3.1 equiv) were added to a solution containing **9** (20.0 mg, 0.06 mmol, 1 equiv) and (*R*)-(+)-MTPA (42.0 mg, 0.18 mmol, 3.1 equiv) in DCM (1.0 mL, [**9**]=0.06 M) under an argon atmosphere. After stirring 5 h at room temperature, Celite® (c.a. 1 g) was added and the solvent was removed under reduced pressure. Purification by manual flash chromatography (hexanes 98:2 ethyl acetate) afforded a colorless oil identified as **9-(*R*)-Mosher ester** (19.2 mg, 0.048 mmol, 82% yield).

^{1}H NMR ($CDCl_3$, 500 MHz): δ (ppm) 7.43 – 7.23 (m, 8H, 5xC($^{4''}$Ar)-H + 3xC(^{5}Ar)-H), 7.21 – 7.09 (m, 2H, 3xC(^{5}Ar)-H), 5.30 (ddd, J = 8.3, 6.1, 1.0 Hz, 1H, C(2)-H), 3.96 (td, J = 8.2, 6.1 Hz, 1H, C(1)-H), 3.69 – 3.61 (m, 2H, C(3')-H), 3.57 – 3.51 (m, 1H, 1xC(1')-H), 3.50 (q, J = 1.2 Hz, 3H, C(5")-H), 3.46 (dt, J = 9.2, 6.4 Hz, 1H, 1xC(1')-H), 2.92 (dt, J = 10.7, 8.7 Hz, 1H, C(3)-H), 2.54 (dddd, J = 11.1, 9.1, 8.0, 1.0 Hz, 1H, 1xC(1')-H, C(4)-H_a), 1.77 – 1.68 (m, 3H, C(2')-H + C(4)-H_b), 0.84 (s, 9H, C(6')-H), 0.00 (s, 6H, C(4')-H).

^{13}C NMR ($CDCl_3$, 101 MHz): δ (ppm) 165.4, 132.3, 129.8, 128.7, 128.5, 127.4, 127.0, 126.9, 80.4, 75.3, 65.9, 59.8, 55.7, 38.6, 33.1, 28.3, 26.1, 18.5, -5.2, -5.2.

^{19}F NMR ($CDCl_3$, 472 MHz): δ (ppm) -71.54 (CF_3).

IR (neat): 1740 (m), 1268 (m), 1176 (s), 712 (s). cm^{-1}.

HRMS (ESI): $C_{29}H_{40}F_3O_5Si^+$ $[M+H]^+$ = 553.2592; found = 553.2604.

$[\alpha]_D^{25}$ = +35.2 (c = 0.69, CH_2Cl_2).

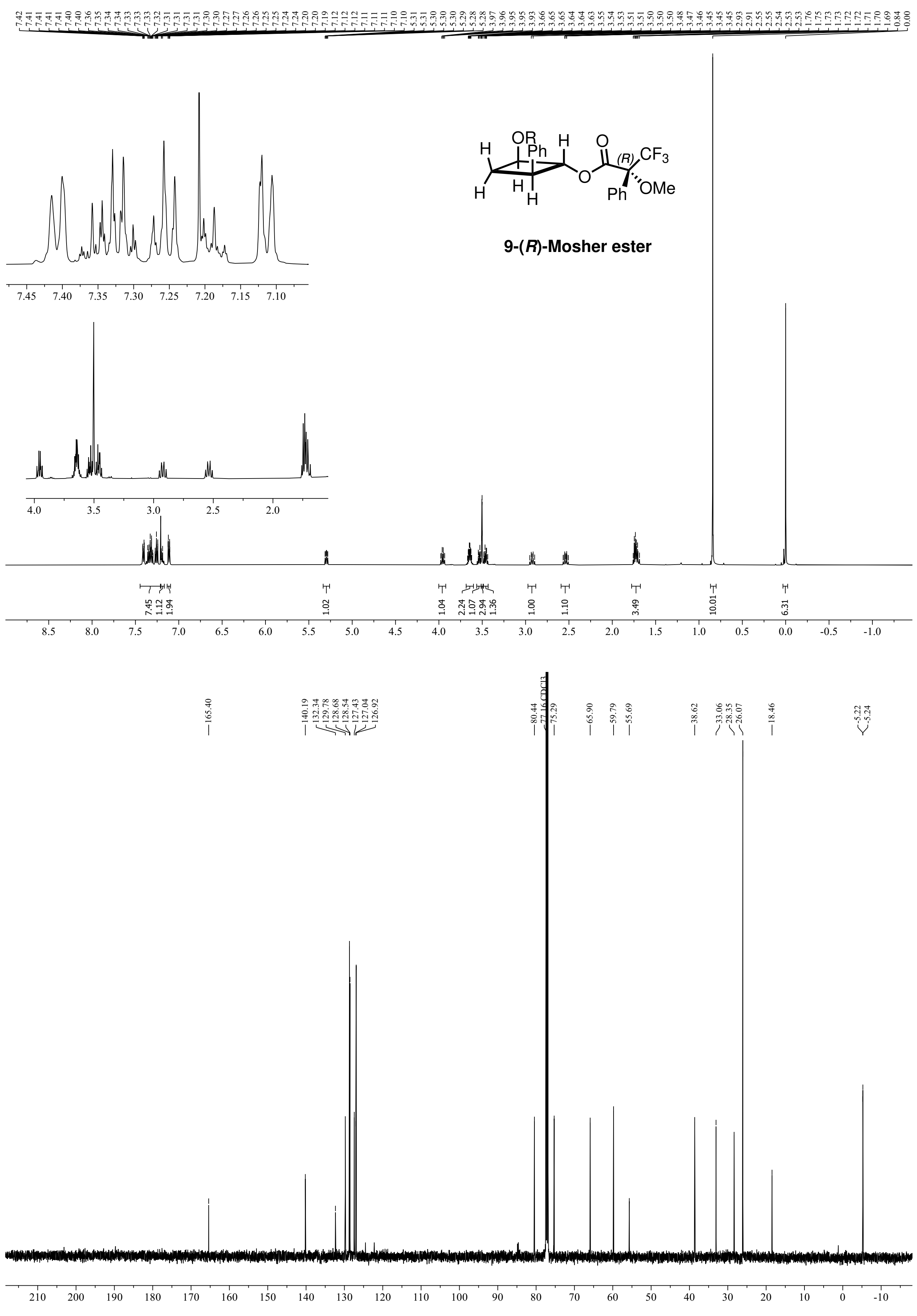

Figure 60: ^{1}H NMR (500 MHz, $CDCl_3$, top) and ^{13}C NMR (126 MHz, $CDCl_3$, bottom) for **9-(*R*)-Mosher ester**.

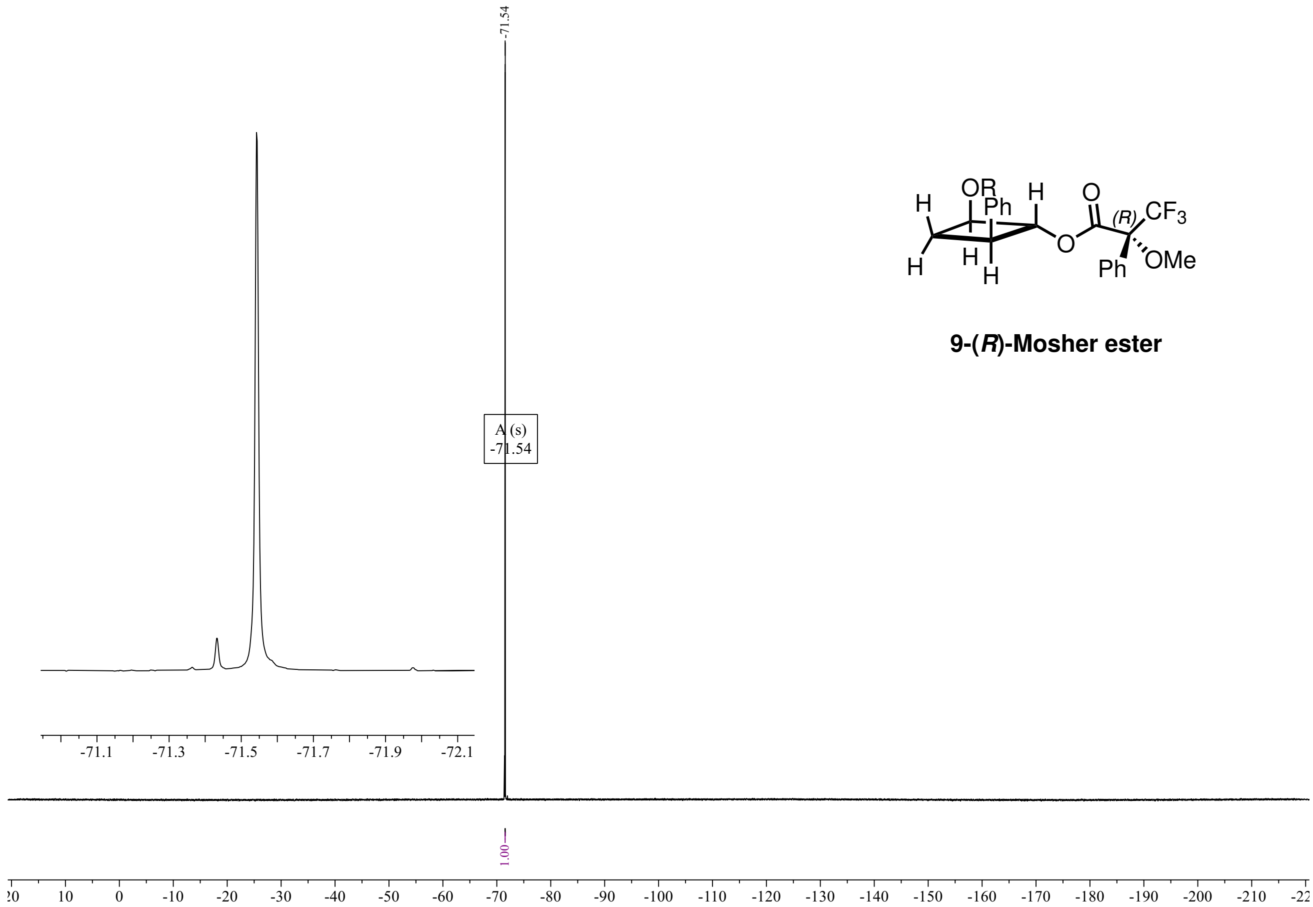

Figure 61: ^{19}F NMR for compound **9-(*R*)-Mosher ester**.

5 Mosher ester analysis for the determination of absolute configuration

5.1 Determination of relative configuration in compound 9

In order to determine the absolute configuration of compound **9** (and thus the absolute stereochemistry set by the Rh in the key step) by Mosher ester analysis, first we need to determine the relative configuration of compound **9**.

1. **HSQC of compound 9** (Figure 62):
   - Signal B (δ 4.01, 80.2 ppm): show H-2.
   - Signal C (δ 3.74, 78.4 ppm): show H-1.
   - Signals D (δ 3.70, 78.4 ppm), E (δ 3.61, 65.6 ppm) and H (δ 1.80, 33.1 ppm): show 3xCH_2 assigned to 3', 1' and 2' respectively.
   - Signal F (δ 2.78, 41.0 ppm): shows benzylic CH (H-3).
   - Signals G (δ 2.48, 27.5 ppm) and I (1.56, 27.5 ppm): show diasterotopic CH_2. These are protons H-4a and H-4b respectively.
2. **HMBC of compound 9** (Figure 63):
   - Signal D (δ 3.61, 78.3 ppm): Shows interaction between 1' and 1. This signal helps us distinguish 1' from 3' and 1 from 2.
3. **NOESY of compound 9** (Figure 64):
   - Signal A (δ 2.80, 3.78 ppm): shows interaction between H-3 and H-1 $\longrightarrow$ H-3 and H-1 are in *cis*.
   - Signal B (δ 2.48, 2.81 ppm): shows interaction between H-4a and H-3. This signal helps us distinguish 4a from 4b.

From all the above, one can draw the conclusion that protons H-1, H-4a and H-3 are all in *cis*, leaving the OH group in *trans* with the phenyl ring and the ether chain. This is in concordance with the idea that the hydroborylation step to generate **10** from **3aa** takes place at the face of the cyclobutene opposite from the phenyl ring.

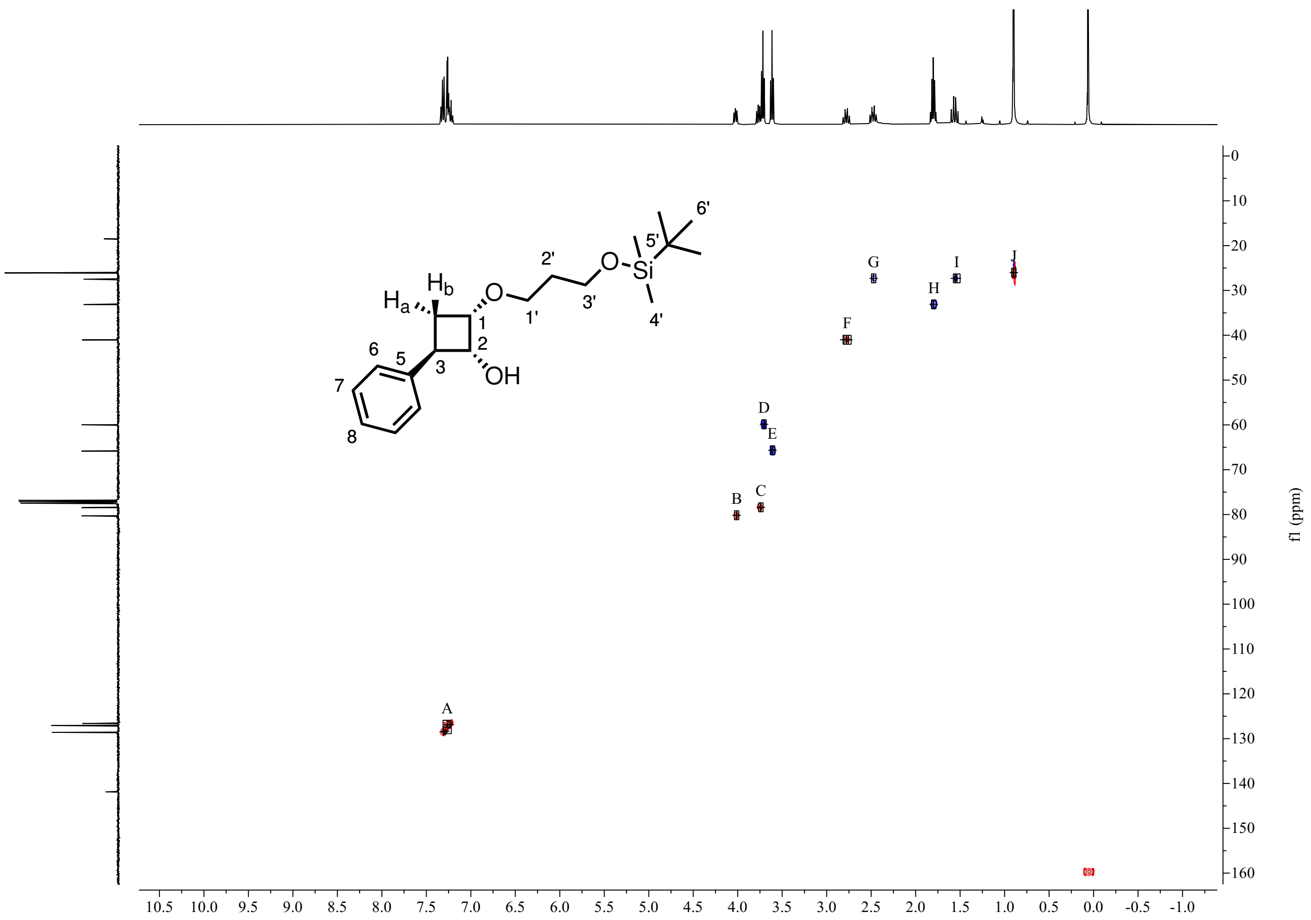

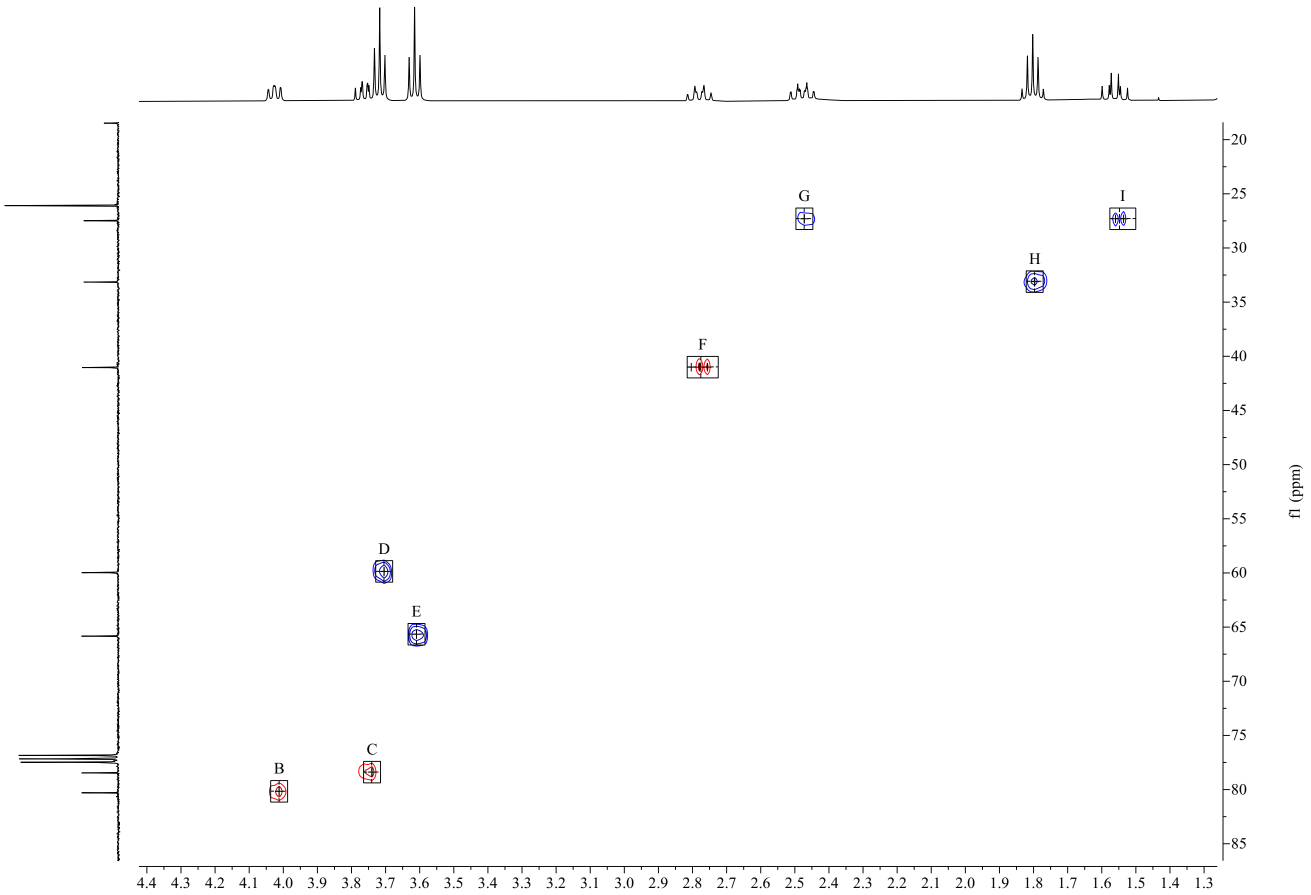

Figure 62: ^{1}H NMR (400 MHz, $CDCl_3$, top) and ^{13}C NMR (101 MHz, $CDCl_3$, bottom) for **9**.

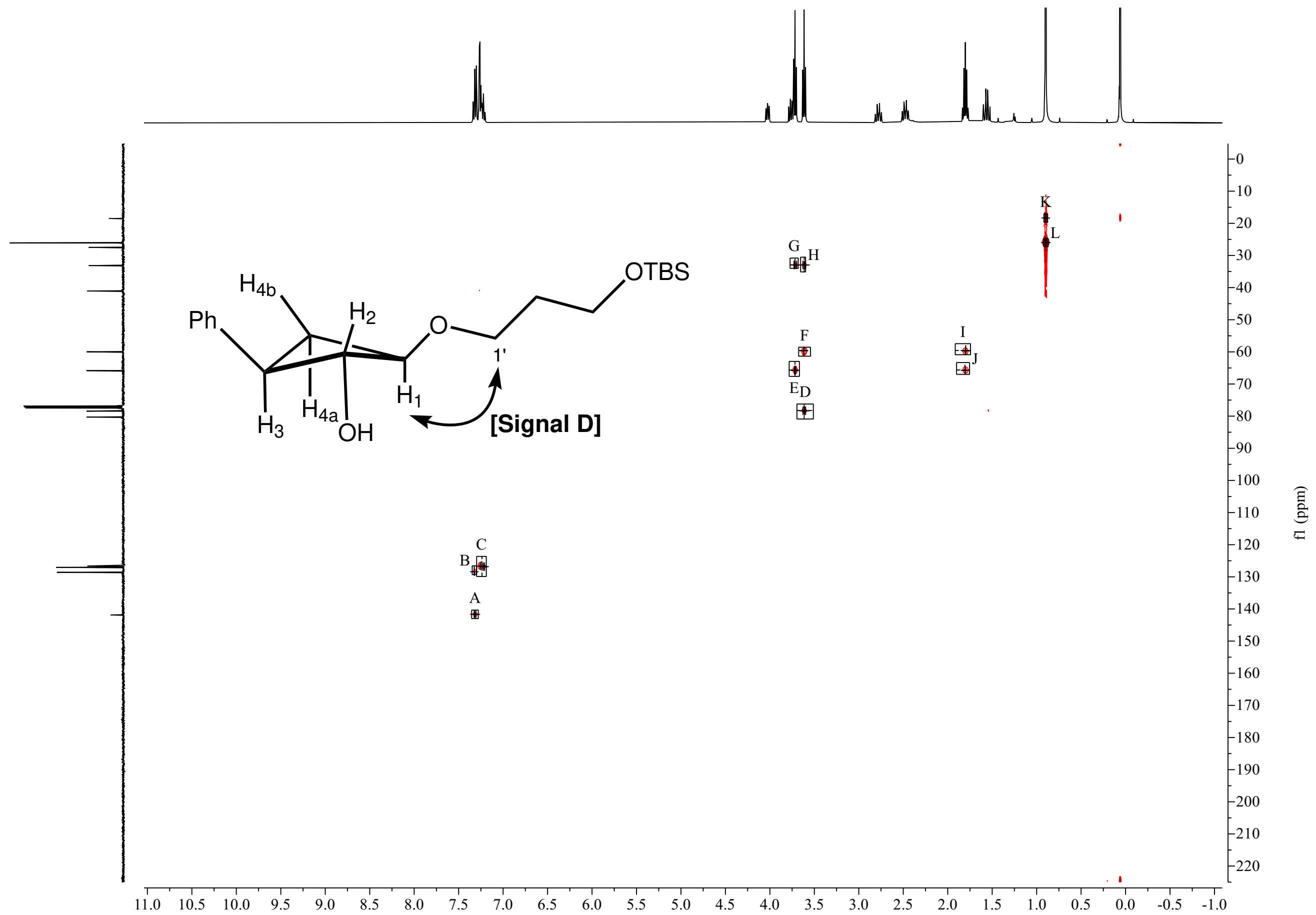

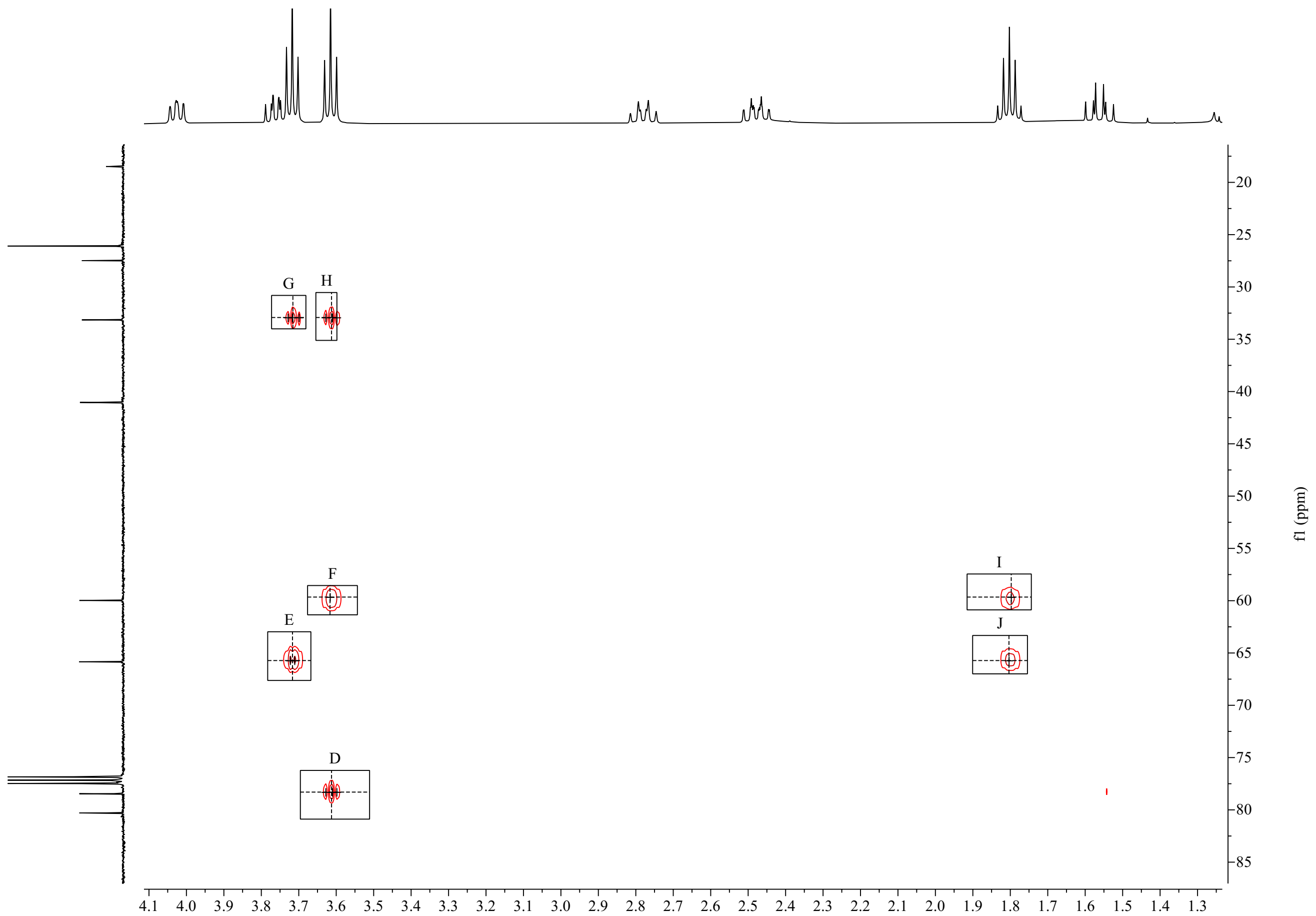

Figure 63: ^{1}H NMR (400 MHz, $CDCl_3$, top) and ^{13}C NMR (101 MHz, $CDCl_3$, bottom) for **9**.

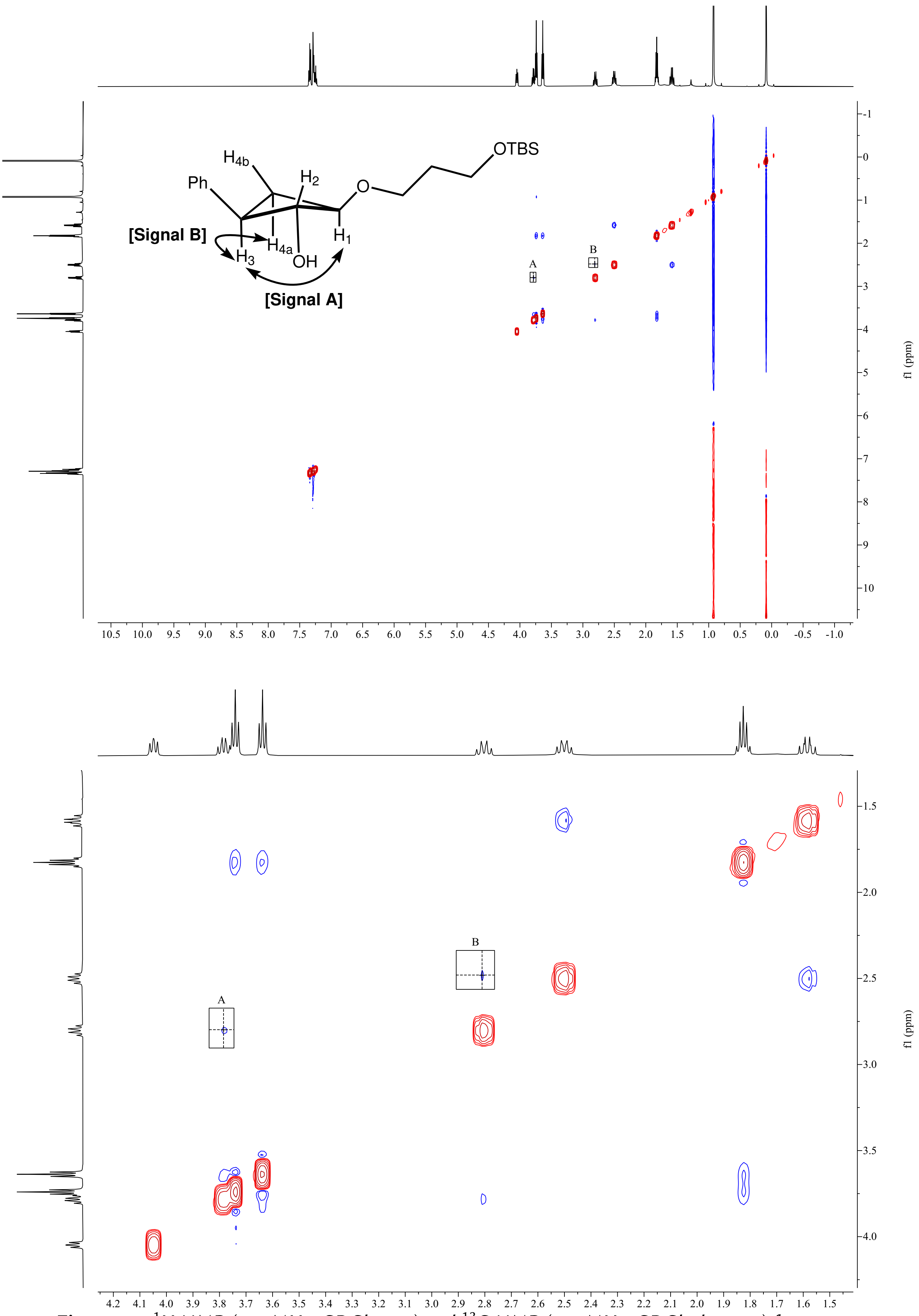

Figure 64: ^{1}H NMR (400 MHz, $CDCl_3$, top) and ^{13}C NMR (101 MHz, $CDCl_3$, bottom) for **9**.

5.2 Determination of absolute configuration in compound 9

The absolute stereochemistry of compound **10**, and also the absolute stereochemistry set by the Rh in the key step, is elucidated by performing a Mosher ester analysis.[4] Both diasteroisomers of the Mosher ester were prepared from **9** (95% ee) using both both enantiomers of the corresponding acid (Figure 65).

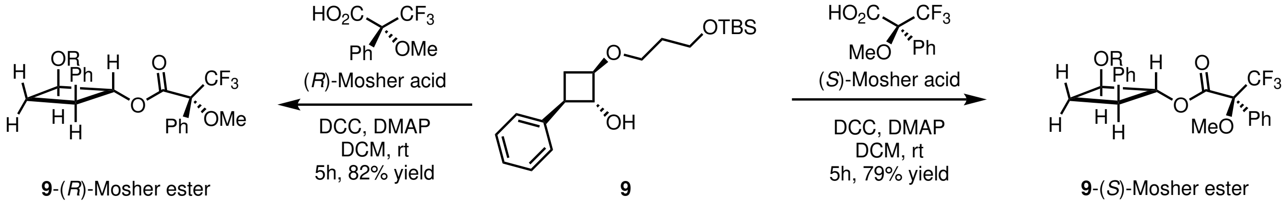

Figure 65: Preparation of both diasteroisomers of Mosher ester.

After isolation by manual flash chromatography, ^{1}HNMR (500 MHz, $CDCl_3$) was recorded for both compounds. Table 1 shows the chemical shifts of the different H atoms in both molecules, as well as the difference in ppm and Hz with respect to the (*S*) ester.

Table 1: Chemical shifts of (*S*) and (*R*) esters and the difference between the two, ordered from largest to smallest. ^{a}Due to the complexity of the aromatic region, an arbitrary sharp peak was chosen to be representative of the whole block. NOTE: H-4b couldn't be assigned as it overlapped with H-2' in both spectra.

H Assignment	δ S-ester (9-(S)-ester) (ppm)	δ R-ester (9-(R)-ester) (ppm)	$\Delta\delta^{SR}$ (=$\delta_S - \delta_R$) ppm	Hz (500 MHz)
**3**	3.03	2.92	0.11	+55
**Ar**^{a}	7.18	7.10	0.08	+40
**4a**	5.31	5.30	0.01	+5
**4'**	2.55	2.54	0.01	+5
**6'**	0.84	0.84	0	0
**2'**	1.71	1.72	-0.01	-5
**3'**	3.63	3.64	-0.01	-5
**1' (x1)**	3.48	3.54	-0.05	-25
**1**	3.86	3.96	-0.11	-50
**1' (x1)**	3.36	3.46	-0.1	-50

In summary, what Table 1 shows is that when using the (*S*) enantiomer of the Mosher acid, the side of the molecule containing the alkyl ether (e.g. H-1 and H-1') is remarkably shielded, while the side of the molecule containing the aryl (e.g. H-3 and H-Ar) is shielded when using the (*R*) enantiomer of the Mosher acid. Knowing that the spectroscopically relevant conformation of the Mosher ester has the C(H)-O-C(=O)-C-CF_3 substructure in the same plane (0° dihedral angle),[5] we

[4] *Nat Protoc* **2007**, 2, 2451–2458.

[5] *J. Org. Chem.* **1973**, 38, 12, 2143–2147.

can confidently assign the absolute sterecohemistry of alcohol **9** and product **3aa** (Figure 66).

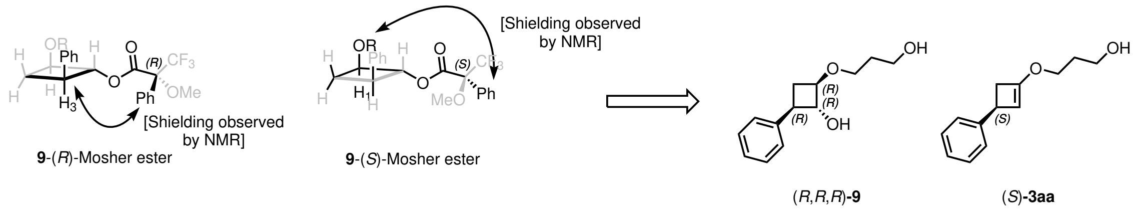

Figure 66: Depicted interactions in Table 1 and absolute configuration in products **9** and **3aa**.

6 SFC traces

SFC traces for compound 3aa

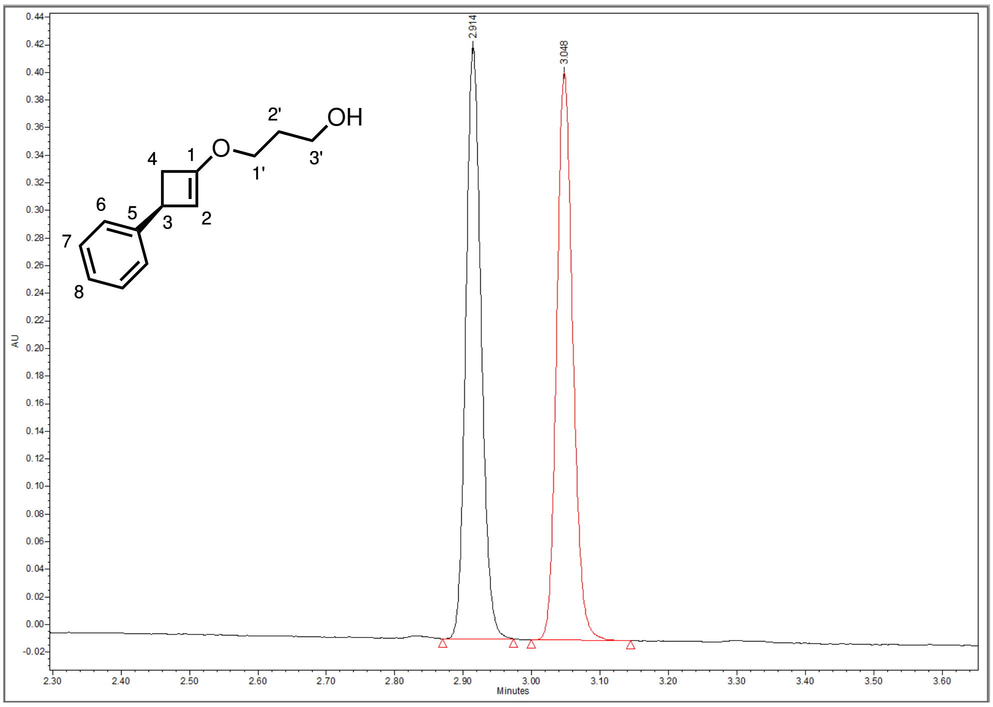

	Name	Retention Time (min)	Purity1 Angle	Purity1 Threshold	PDA/FLR Match1 Spect. Name	PDA/FLR Match1 Angle	PDA/FLR Match1 Threshold	PDA/FLR Match1 Lib. Name	Area (µV*sec)	% Area
1		2.914							667444	49.71
2		3.048							675144	50.29

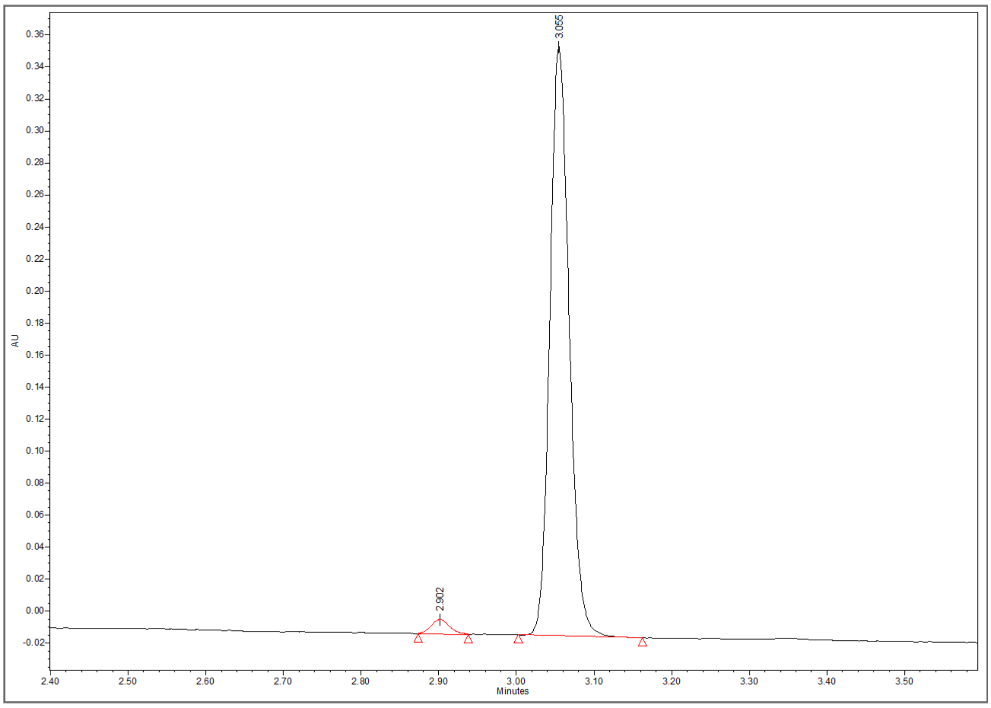

	Name	Retention Time (min)	Purity1 Angle	Purity1 Threshold	PDA/FLR Match1 Spect. Name	PDA/FLR Match1 Angle	PDA/FLR Match1 Threshold	PDA/FLR Match1 Lib. Name	Area (µV*sec)	% Area
1		2.902							14658	2.25
2		3.055							638125	97.75

Figure 67: SFC traces for compound **(±)-3aa** (top) and enantioenriched **(+)-3aa** (bottom).

SFC traces for compound 3ab

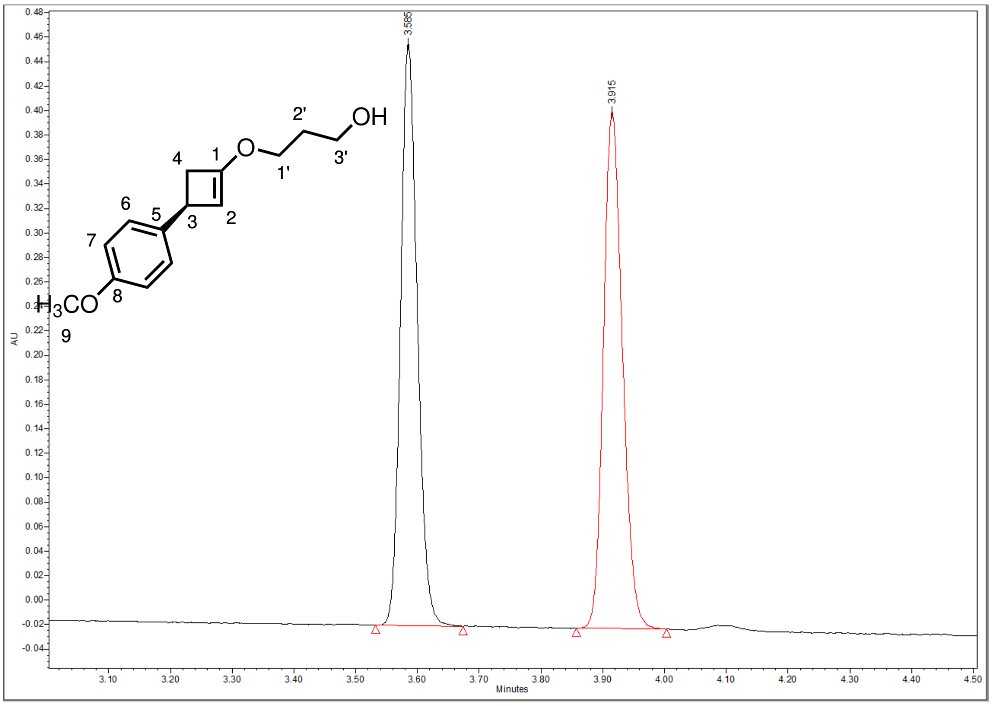

	Name	Retention Time (min)	Purity1 Angle	Purity1 Threshold	PDA/FLR Match1 Spect. Name	PDA/FLR Match1 Angle	PDA/FLR Match1 Threshold	PDA/FLR Match1 Lib. Name	Area (µV*sec)	% Area
1		3.585							898357	49.88
2		3.915							902524	50.12

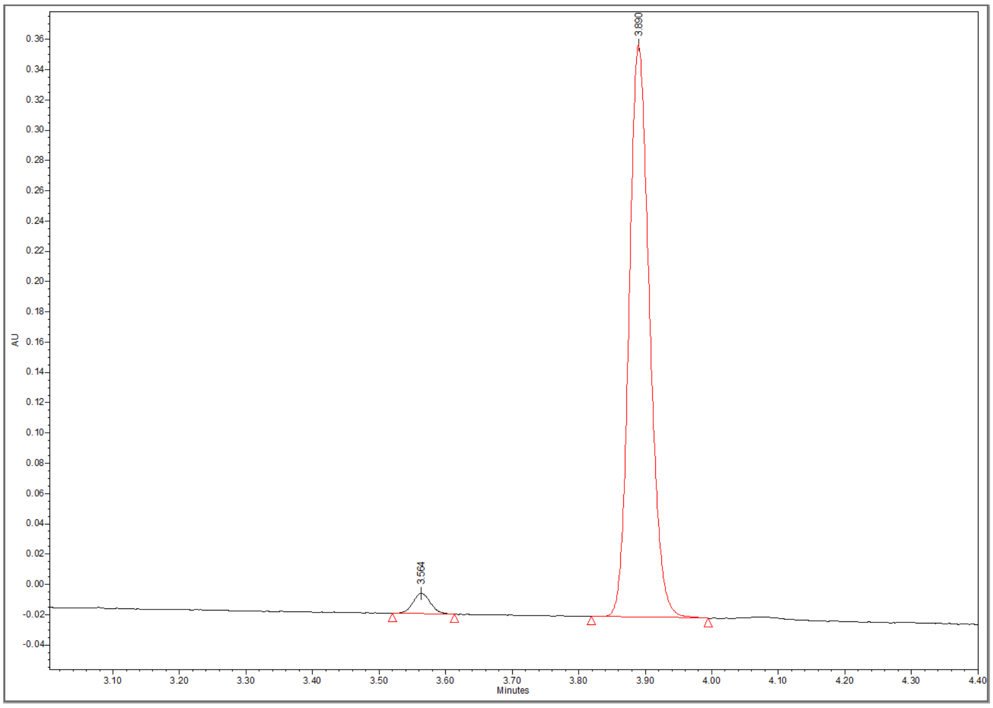

	Name	Retention Time (min)	Purity1 Angle	Purity1 Threshold	PDA/FLR Match1 Spect. Name	PDA/FLR Match1 Angle	PDA/FLR Match1 Threshold	PDA/FLR Match1 Lib. Name	Area (µV*sec)	% Area
1		3.564							24176	2.92
2		3.890							804763	97.08

Figure 68: SFC traces for compound **(±)-3ab** (top) and enantioenriched **(+)-3ab** (bottom).

SFC traces for compound 3ac

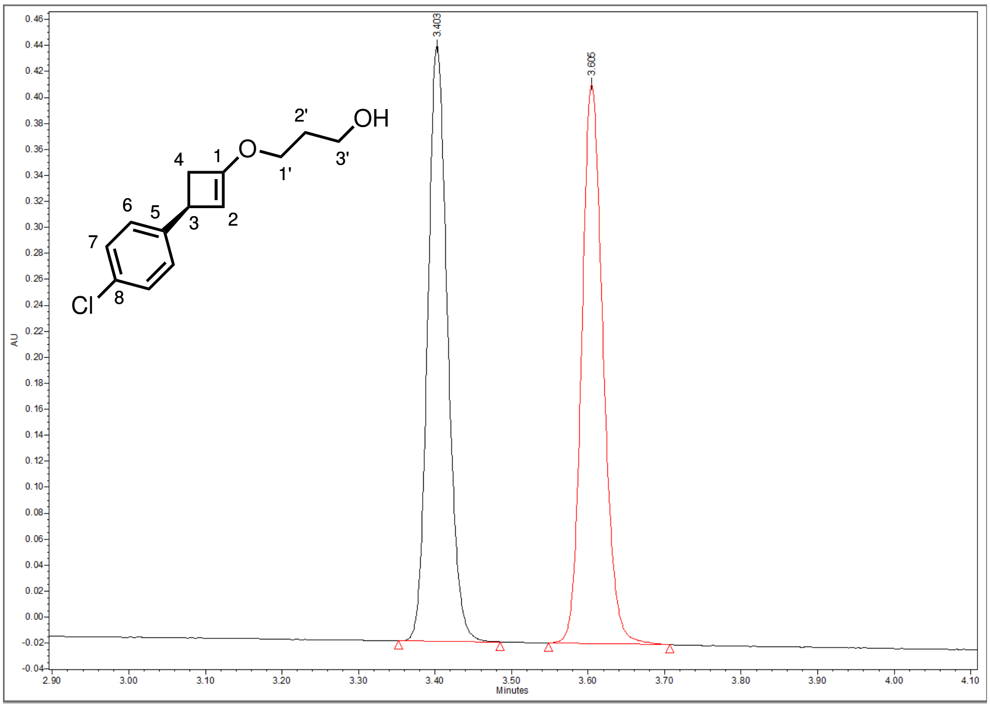

Name	Retention Time (min)	Purity1 Angle	Purity1 Threshold	PDA/FLR Match1 Spect. Name	PDA/FLR Match1 Angle	PDA/FLR Match1 Threshold	PDA/FLR Match1 Lib. Name	Area (µV*sec)	% Area
1		3.403							824685	49.69
2		3.605							834992	50.31

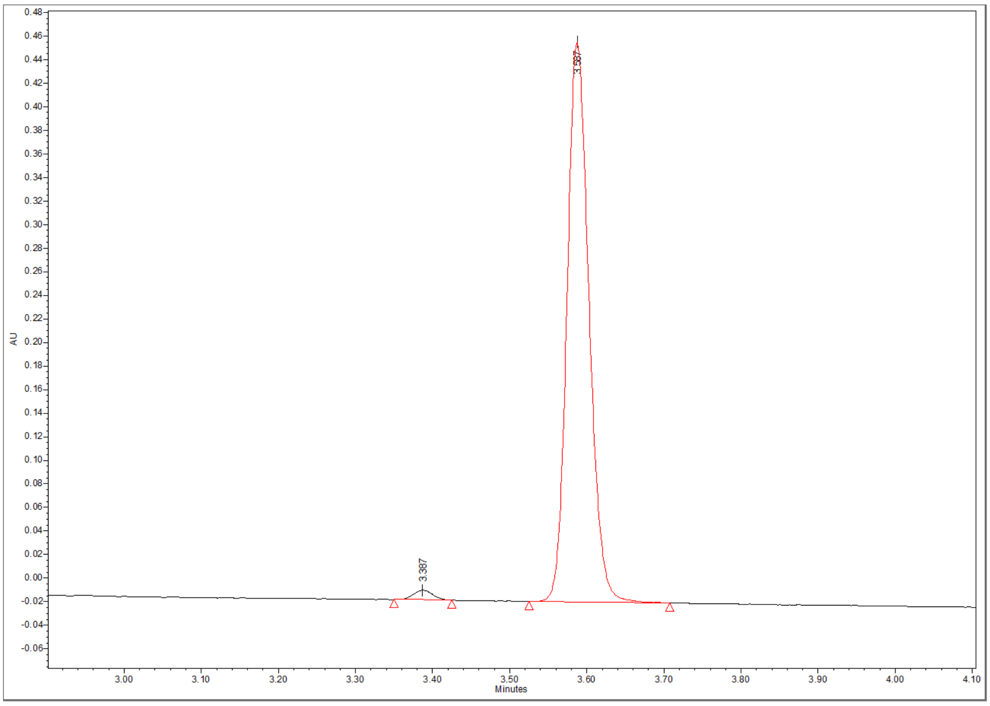

Name	Retention Time (min)	Purity1 Angle	Purity1 Threshold	PDA/FLR Match1 Spect. Name	PDA/FLR Match1 Angle	PDA/FLR Match1 Threshold	PDA/FLR Match1 Lib. Name	Area (µV*sec)	% Area
1		3.387							14073	1.48
2		3.587							938379	98.52

Figure 69: SFC traces for compound **(±)-3ac** (top) and enantioenriched **(+)-3ac** (bottom).

SFC traces for compound 3ad

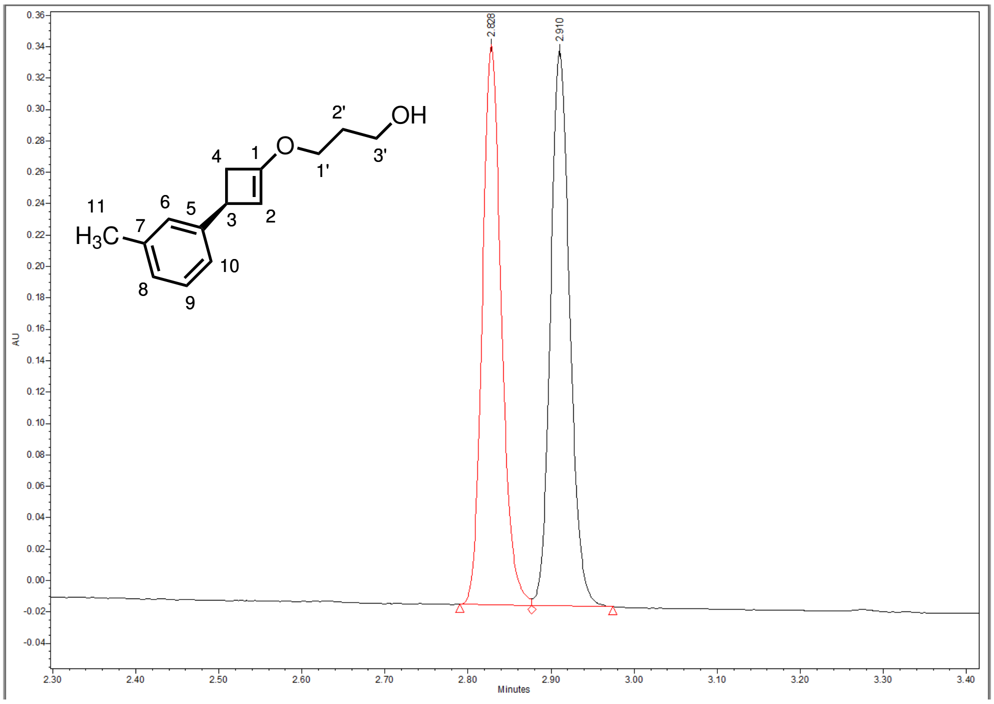

	Name	Retention Time (min)	Purity1 Angle	Purity1 Threshold	PDA/FLR Match1 Spect. Name	PDA/FLR Match1 Angle	PDA/FLR Match1 Threshold	PDA/FLR Match1 Lib. Name	Area (µV*sec)	% Area
1		2.828							569147	49.99
2		2.910							569488	50.01

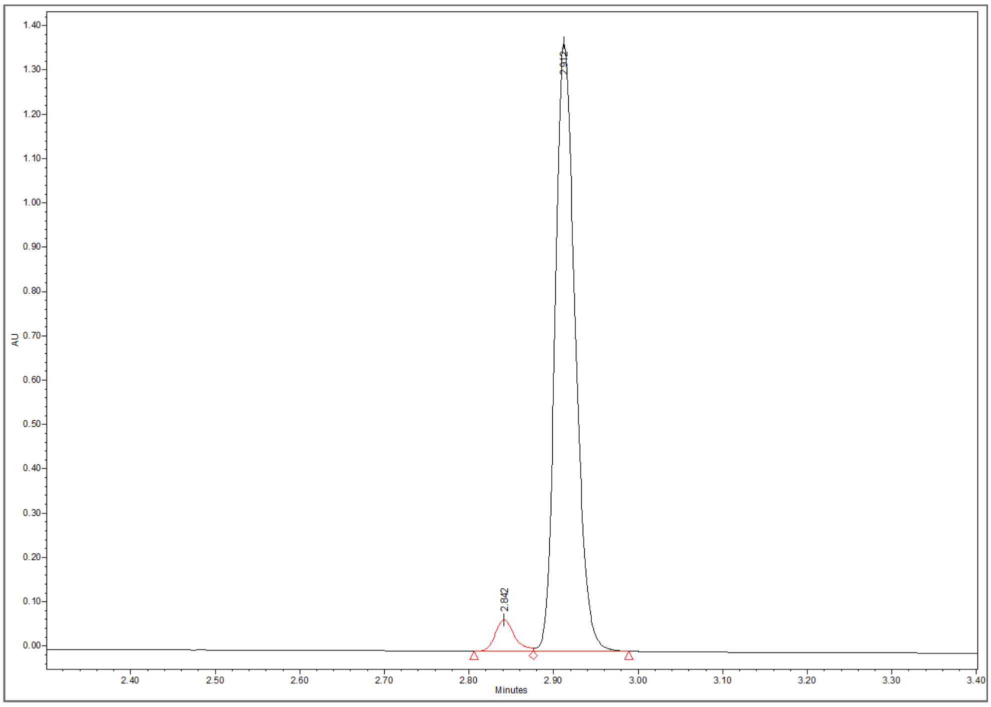

	Name	Retention Time (min)	Purity1 Angle	Purity1 Threshold	PDA/FLR Match1 Spect. Name	PDA/FLR Match1 Angle	PDA/FLR Match1 Threshold	PDA/FLR Match1 Lib. Name	Area (µV*sec)	% Area
1		2.842							112086	4.71
2		2.912							2269549	95.29

Figure 70: SFC traces for compound **(±)-3ad** (top) and enantioenriched **(+)-3ad** (bottom).

SFC traces for compound 3ae

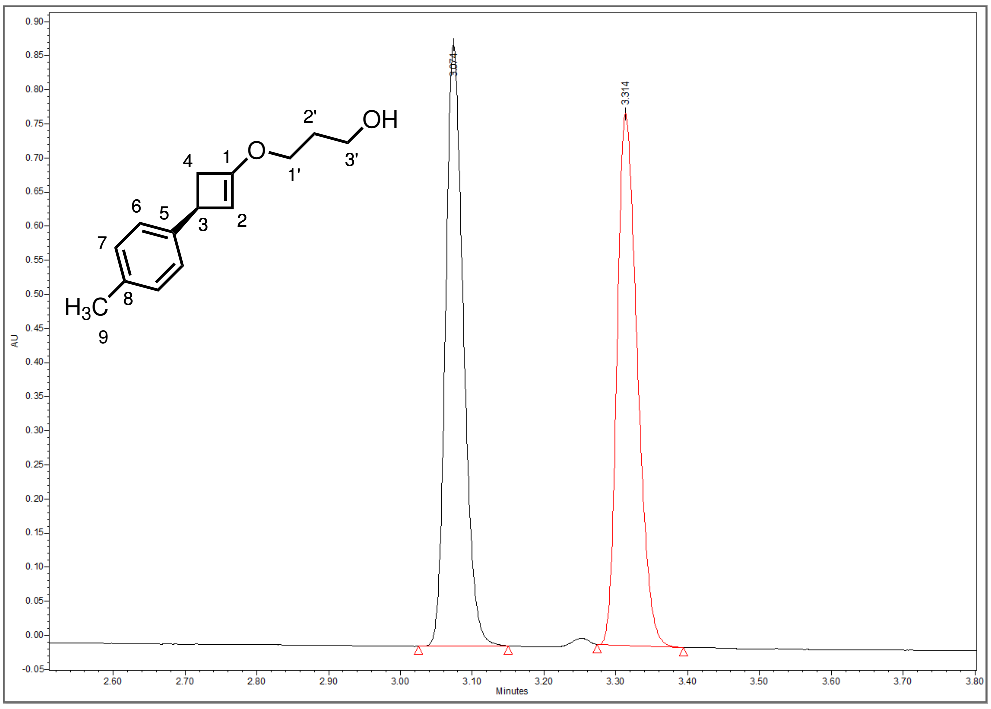

E	Name	Retention Time (min)	Purity1 Angle	Purity1 Threshold	PDA/FLR Match1 Spect. Name	PDA/FLR Match1 Angle	PDA/FLR Match1 Threshold	PDA/FLR Match1 Lib. Name	Area (µV*sec)	% Area
1		3.074							1531404	50.02
2		3.314							1530058	49.98

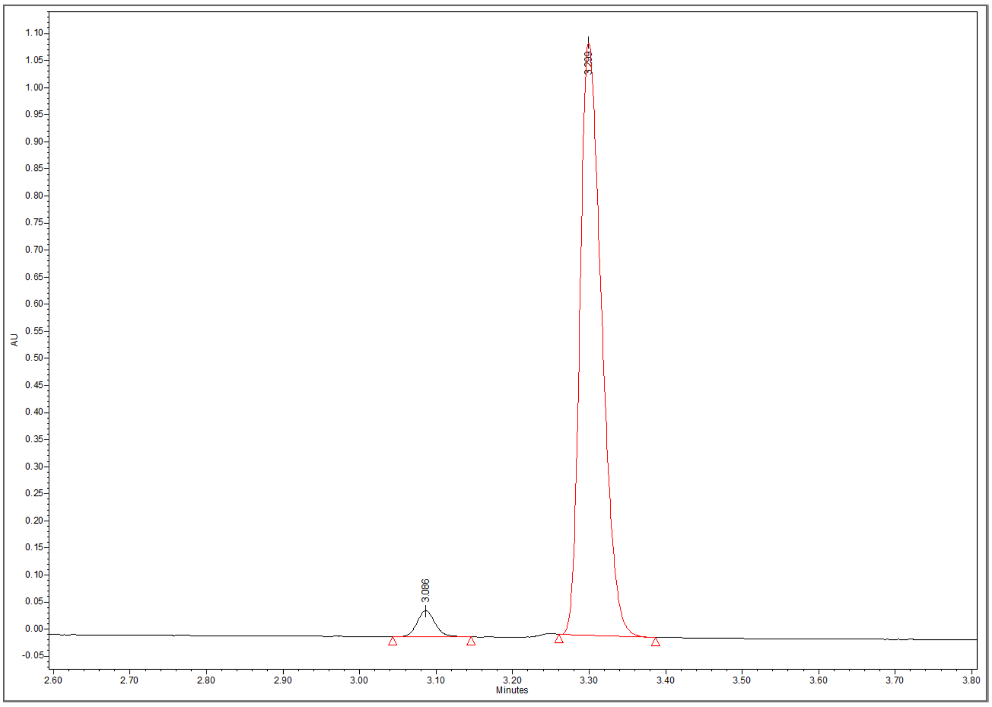

E	Name	Retention Time (min)	Purity1 Angle	Purity1 Threshold	PDA/FLR Match1 Spect. Name	PDA/FLR Match1 Angle	PDA/FLR Match1 Threshold	PDA/FLR Match1 Lib. Name	Area (µV*sec)	% Area
1		3.086							77533	3.49
2		3.299							2146482	96.51

Figure 71: SFC traces for compound **(±)-3ae** (top) and enantioenriched **(+)-3ae** (bottom).

SFC traces for compound 3af

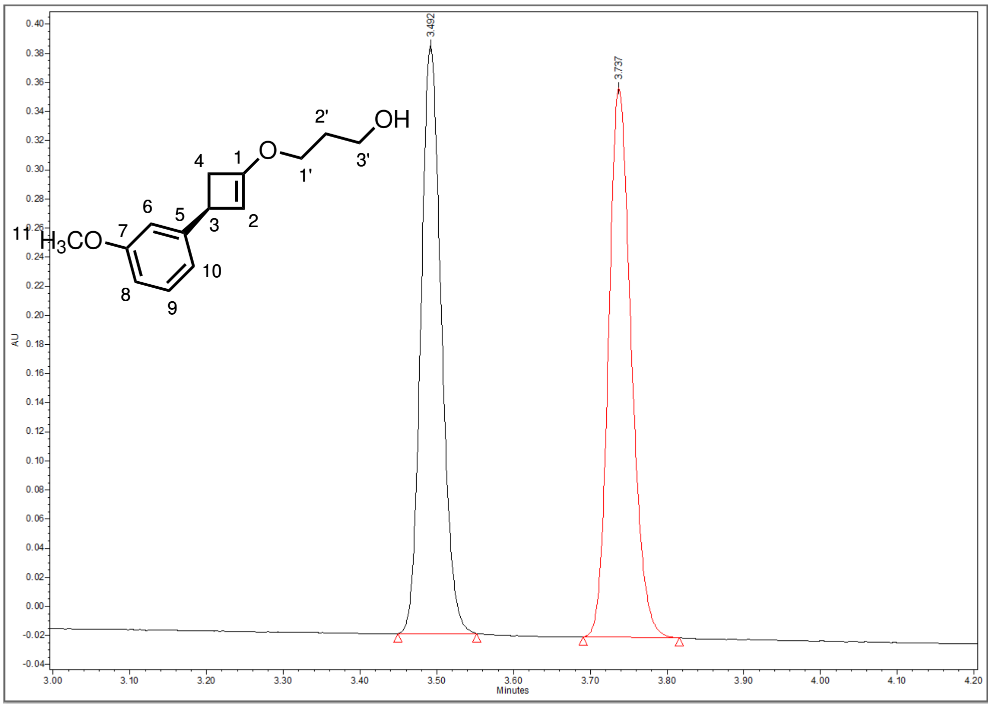

	Name	Retention Time (min)	Purity1 Angle	Purity1 Threshold	PDA/FLR Match1 Spect. Name	PDA/FLR Match1 Angle	PDA/FLR Match1 Threshold	PDA/FLR Match1 Lib. Name	Area (µV*sec)	% Area
1		3.492							744937	48.99
2		3.737							775621	51.01

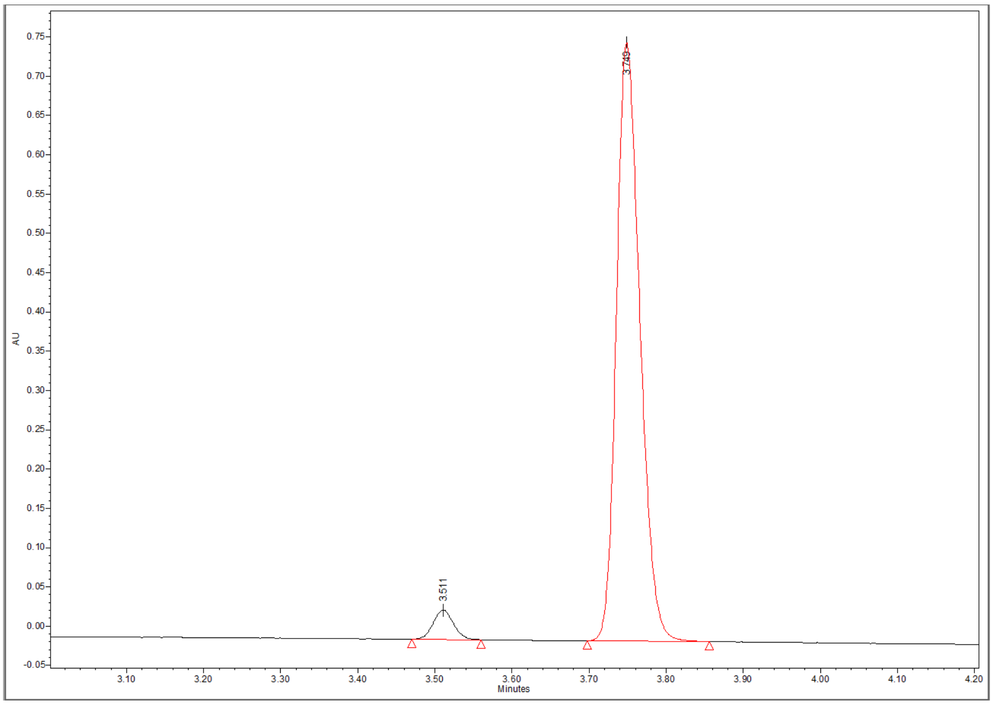

	Name	Retention Time (min)	Purity1 Angle	Purity1 Threshold	PDA/FLR Match1 Spect. Name	PDA/FLR Match1 Angle	PDA/FLR Match1 Threshold	PDA/FLR Match1 Lib. Name	Area (µV*sec)	% Area
1		3.511							68056	4.06
2		3.749							1608274	95.94

Figure 72: SFC traces for compound **(±)-3af** (top) and enantioenriched **(+)-3af** (bottom).

SFC traces for compound 3ag

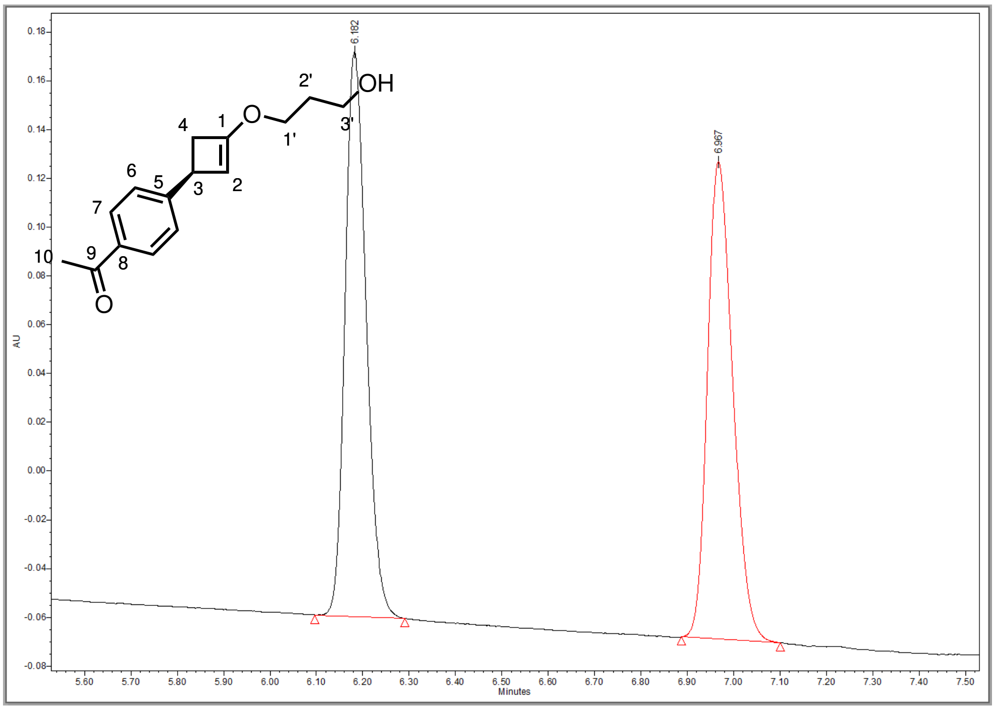

Name	Retention Time (min)	Purity1 Angle	Purity1 Threshold	PDA/FLR Match1 Spect. Name	PDA/FLR Match1 Angle	PDA/FLR Match1 Threshold	PDA/FLR Match1 Lib. Name	Area (µV*sec)	% Area
1		6.182							731603	49.64
2		6.967							742147	50.36

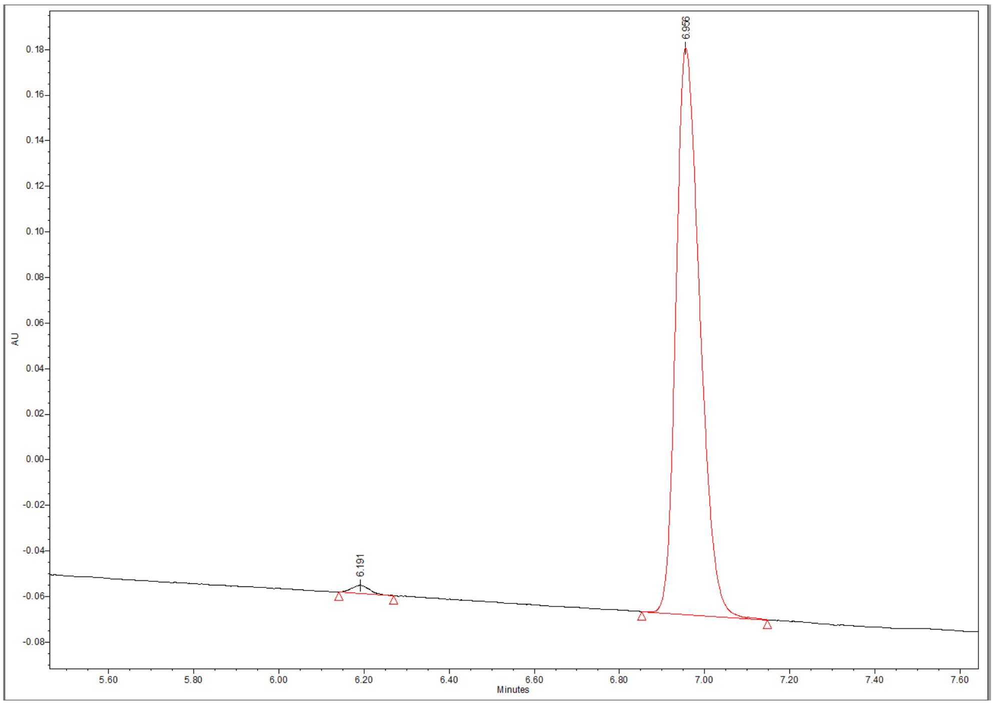

	Name	Retention Time (min)	Purity1 Angle	Purity1 Threshold	PDA/FLR Match1 Spect. Name	PDA/FLR Match1 Angle	PDA/FLR Match1 Threshold	PDA/FLR Match1 Lib. Name	Area (µV*sec)	% Area
1		6.191							9866	1.01
2		6.956							967272	98.99

Figure 73: SFC traces for compound **(±)-3ag** (top) and enantioenriched **(+)-3ag** (bottom).

SFC traces for compound 3ah

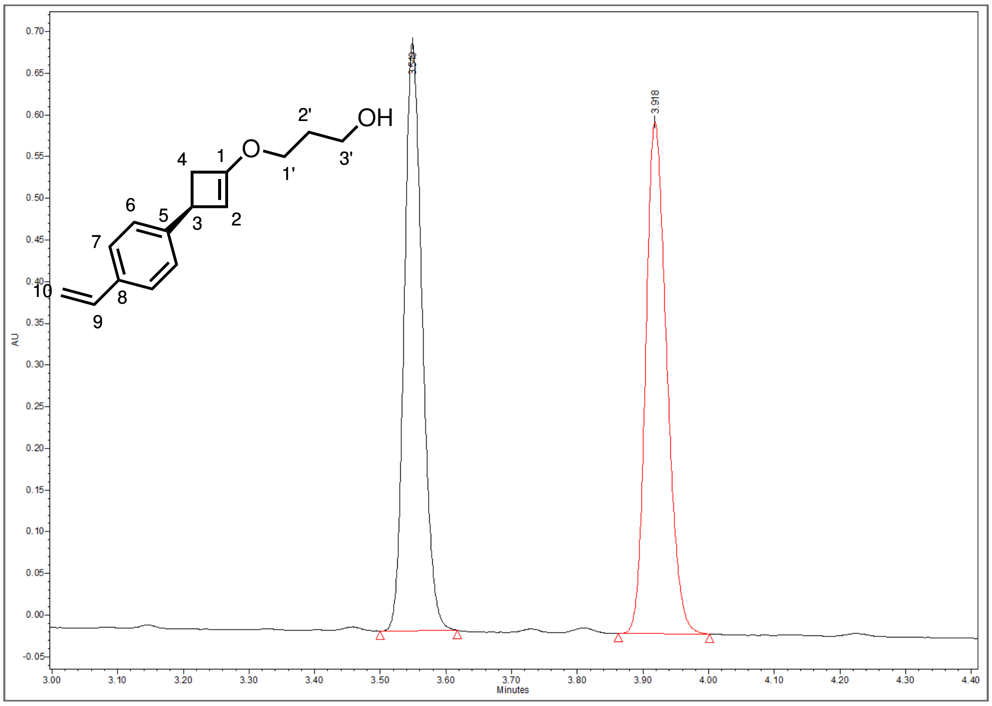

	Name	Retention Time (min)	Purity1 Angle	Purity1 Threshold	PDA/FLR Match1 Spect. Name	PDA/FLR Match1 Angle	PDA/FLR Match1 Threshold	PDA/FLR Match1 Lib. Name	Area (μV*sec)	% Area
1		3.549							1346379	49.67
2		3.918							1364474	50.33

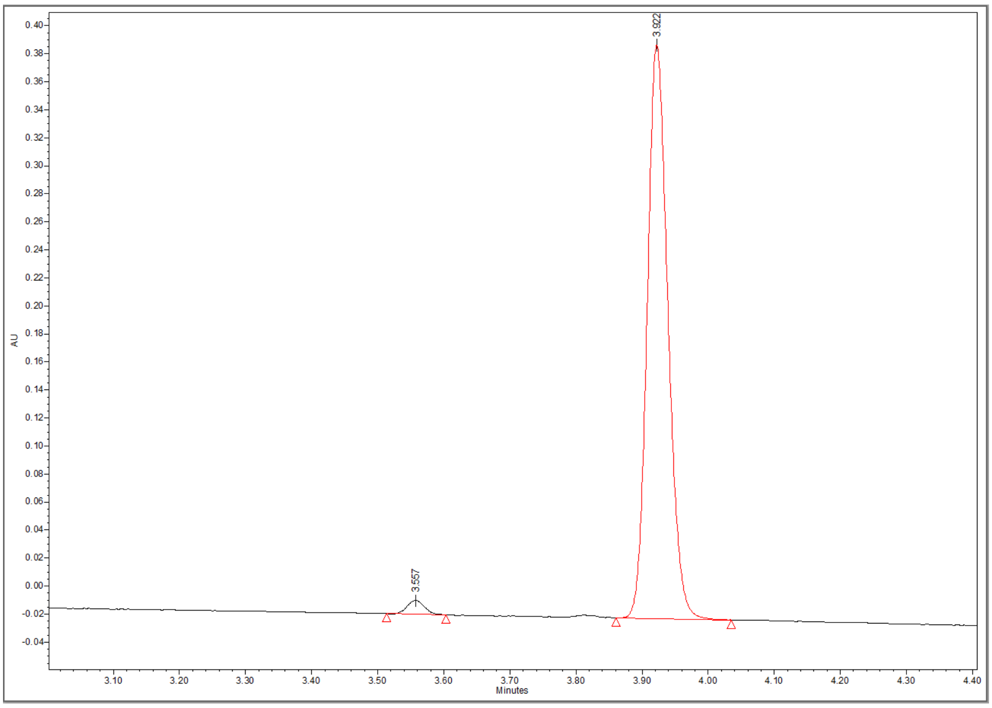

	Name	Retention Time (min)	Purity1 Angle	Purity1 Threshold	PDA/FLR Match1 Spect. Name	PDA/FLR Match1 Angle	PDA/FLR Match1 Threshold	PDA/FLR Match1 Lib. Name	Area (μV*sec)	% Area
1		3.557							17933	1.97
2		3.922							890258	98.03

Figure 74: SFC traces for compound **(±)-3ah** (top) and enantioenriched **(+)-3ah** (bottom).

SFC traces for compound 3ai

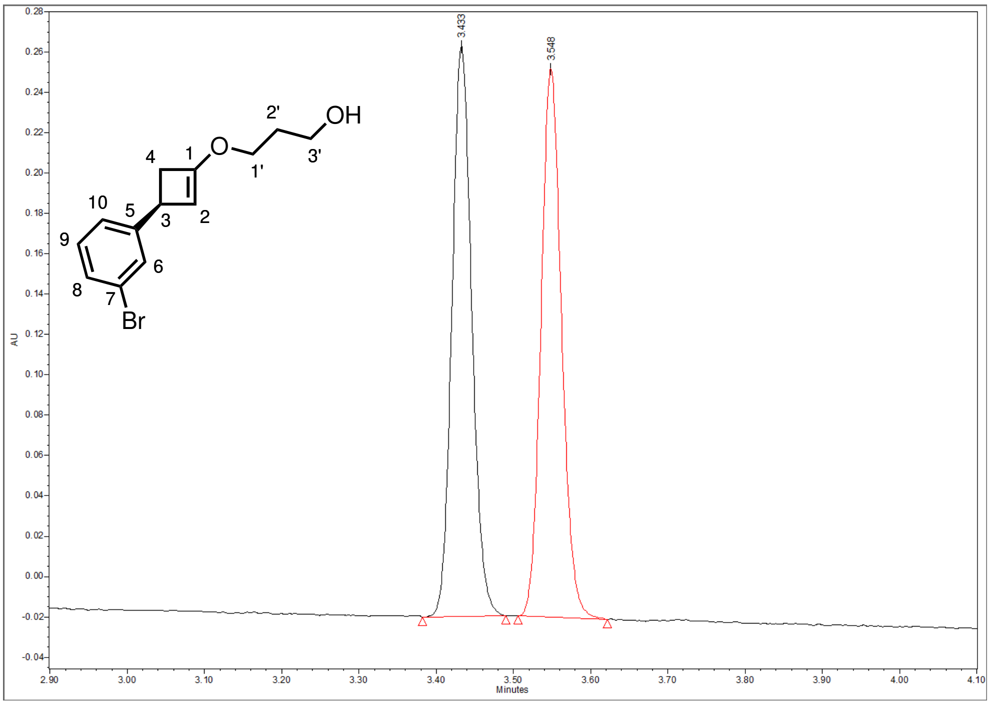

	Name	Retention Time (min)	Purity1 Angle	Purity1 Threshold	PDA/FLR Match1 Spect. Name	PDA/FLR Match1 Angle	PDA/FLR Match1 Threshold	PDA/FLR Match1 Lib. Name	Area (µV*sec)	% Area
1		3.433							503789	49.95
2		3.548							504827	50.05

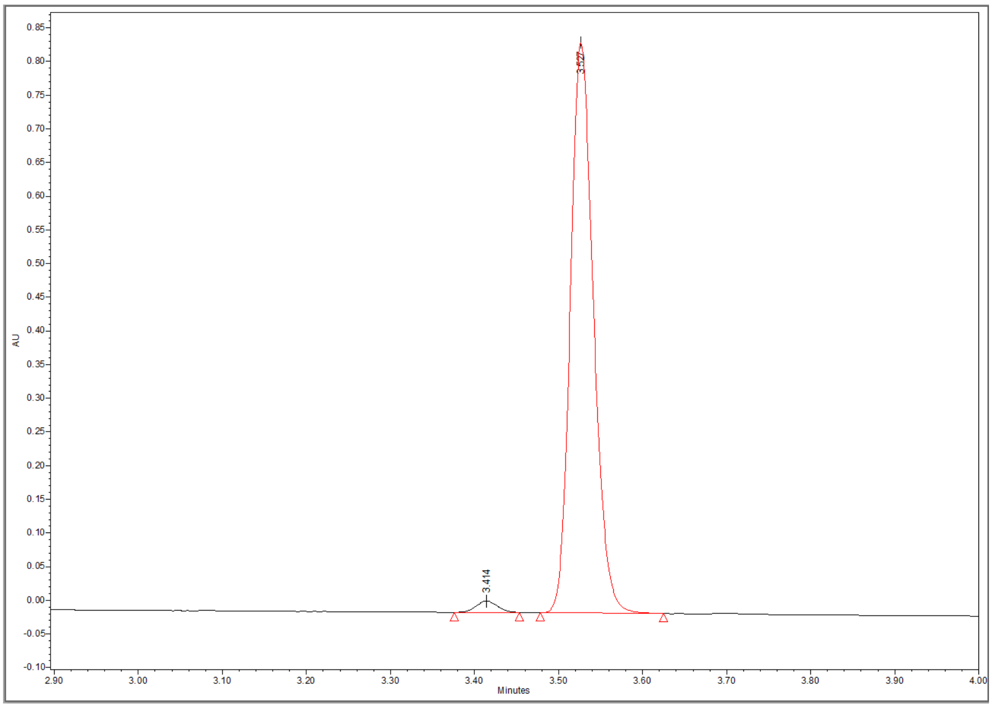

	Name	Retention Time (min)	Purity1 Angle	Purity1 Threshold	PDA/FLR Match1 Spect. Name	PDA/FLR Match1 Angle	PDA/FLR Match1 Threshold	PDA/FLR Match1 Lib. Name	Area (µV*sec)	% Area
1		3.414							30133	1.83
2		3.527							1613526	98.17

Figure 75: SFC traces for compound **(±)-3ai** (top) and enantioenriched **(+)-3ai** (bottom).

SFC traces for compound 3aj

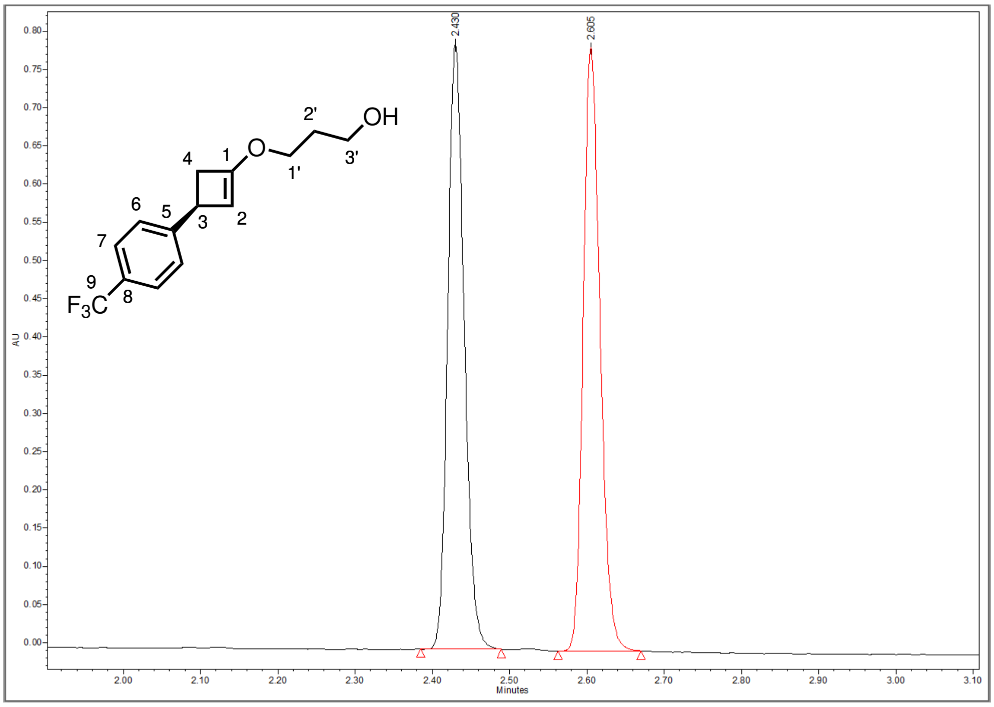

	Name	Retention Time (min)	Purity1 Angle	Purity1 Threshold	PDA/FLR Match1 Spect. Name	PDA/FLR Match1 Angle	PDA/FLR Match1 Threshold	PDA/FLR Match1 Lib. Name	Area (µV*sec)	% Area
1		2.430							1181573	49.69
2		2.605							1196093	50.31

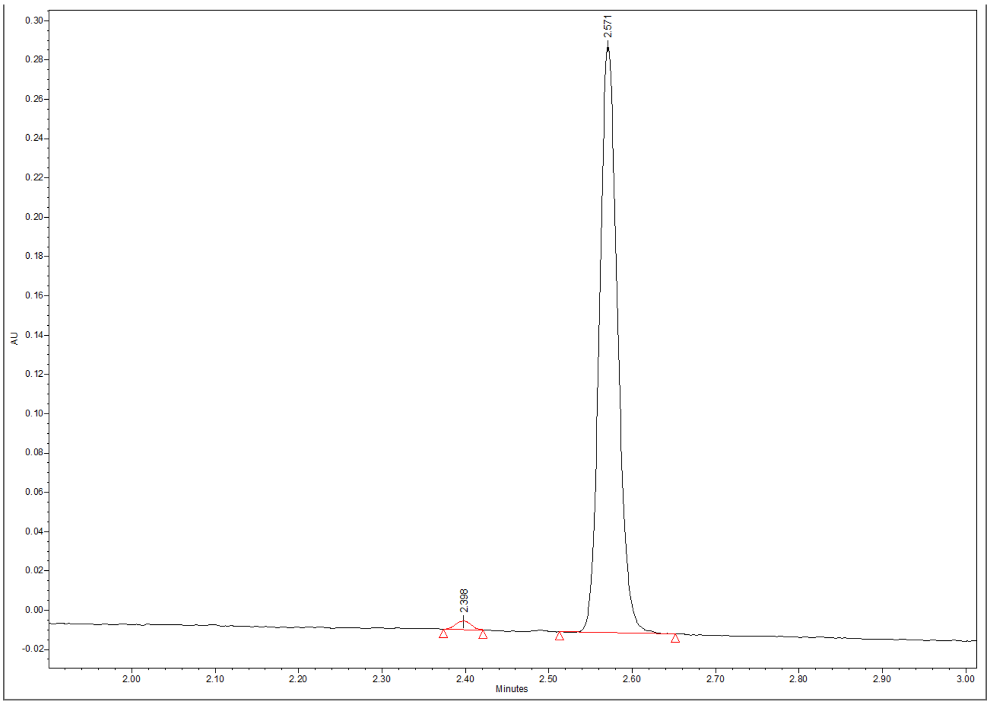

	Name	Retention Time (min)	Purity1 Angle	Purity1 Threshold	PDA/FLR Match1 Spect. Name	PDA/FLR Match1 Angle	PDA/FLR Match1 Threshold	PDA/FLR Match1 Lib. Name	Area (µV*sec)	% Area
1		2.398							5492	1.21
2		2.571							449767	98.79

Figure 76: SFC traces for compound **(±)-3aj** (top) and enantioenriched **(+)-3aj** (bottom).

SFC traces for compound 3ak

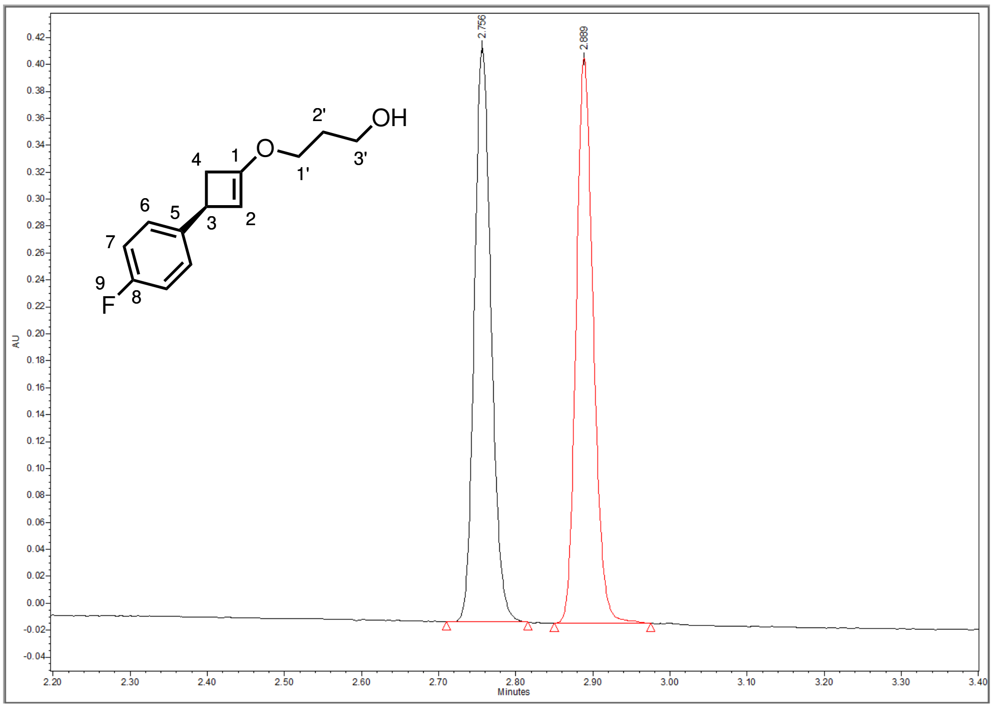

Name	Retention Time (min)	Purity1 Angle	Purity1 Threshold	PDA/FLR Match1 Spect. Name	PDA/FLR Match1 Angle	PDA/FLR Match1 Threshold	PDA/FLR Match1 Lib. Name	Area (µV*sec)	% Area
1	2.756							645408	49.83
2	2.889							649862	50.17

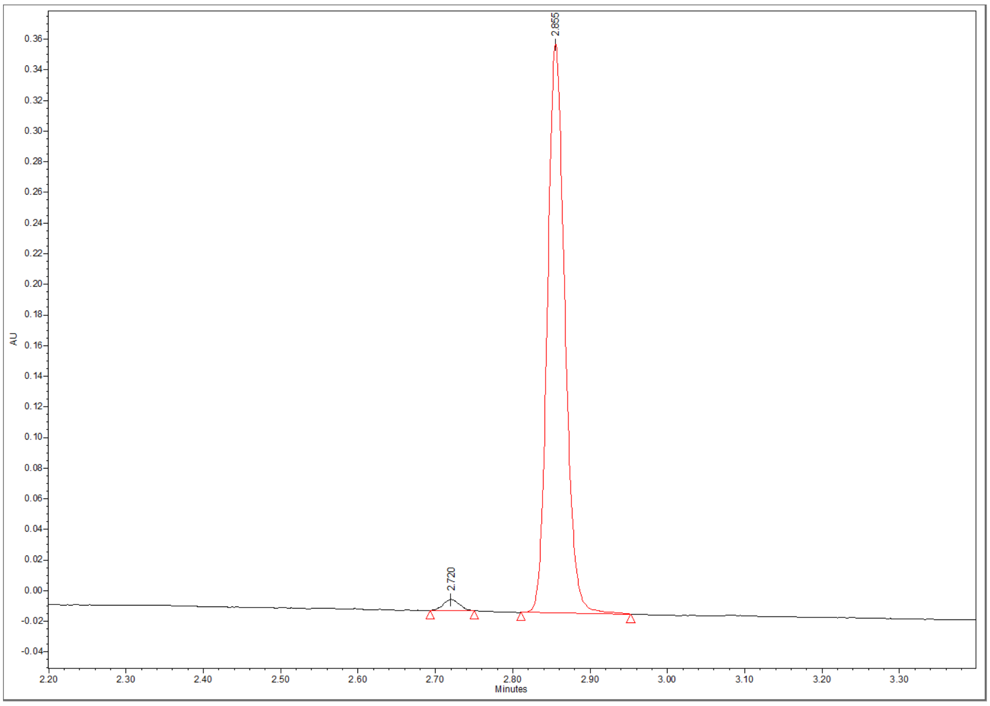

Name	Retention Time (min)	Purity1 Angle	Purity1 Threshold	PDA/FLR Match1 Spect. Name	PDA/FLR Match1 Angle	PDA/FLR Match1 Threshold	PDA/FLR Match1 Lib. Name	Area (µV*sec)	% Area
1	2.720							10506	1.76
2	2.855							585898	98.24

Figure 77: SFC traces for compound **(±)-3ak** (top) and enantioenriched **(+)-3ak** (bottom).

SFC traces for compound 3al

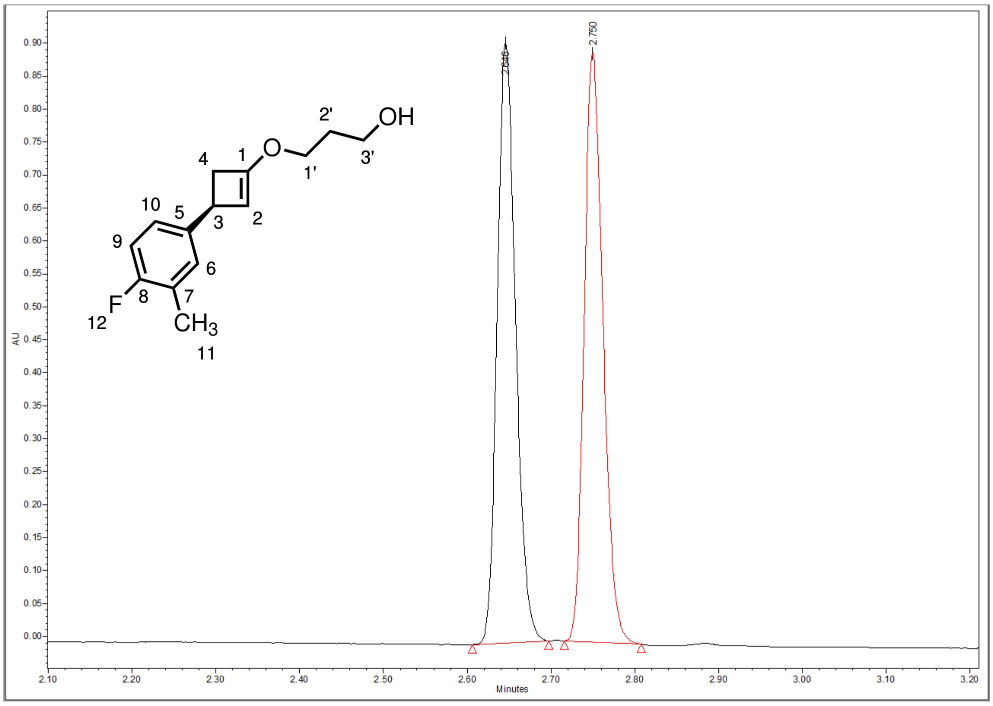

	Name	Retention Time (min)	Purity1 Angle	Purity1 Threshold	PDA/FLR Match1 Spect. Name	PDA/FLR Match1 Angle	PDA/FLR Match1 Threshold	PDA/FLR Match1 Lib. Name	Area (µV*sec)	% Area
1		2.646							1394232	49.93
2		2.750							1398259	50.07

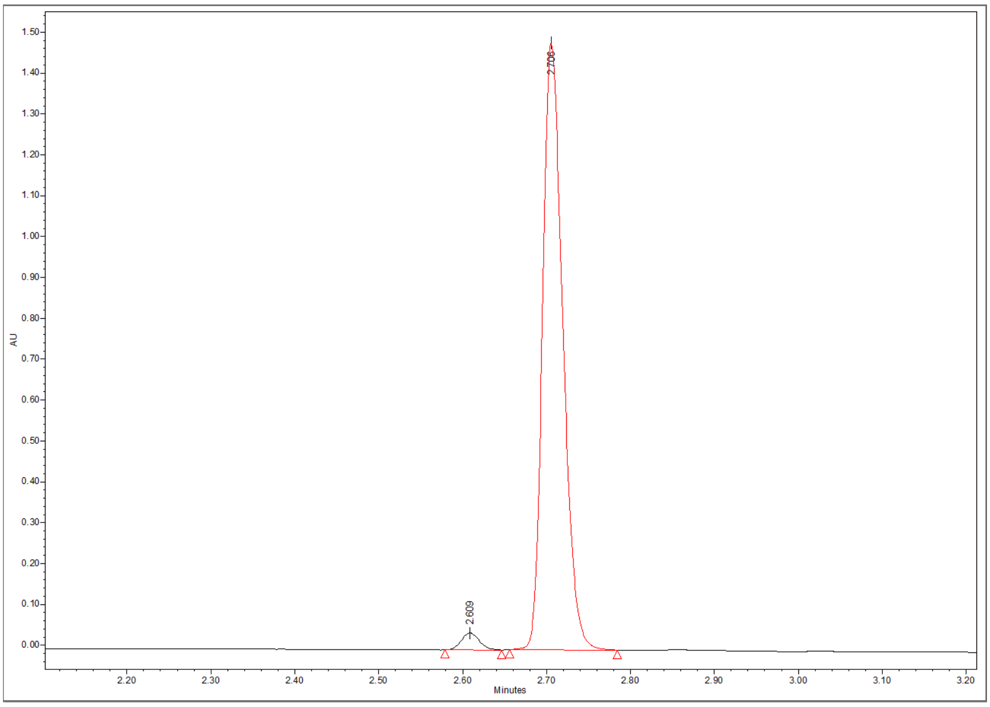

	Name	Retention Time (min)	Purity1 Angle	Purity1 Threshold	PDA/FLR Match1 Spect. Name	PDA/FLR Match1 Angle	PDA/FLR Match1 Threshold	PDA/FLR Match1 Lib. Name	Area (µV*sec)	% Area
1		2.609							61443	2.41
2		2.706							2489483	97.59

Figure 78: SFC traces for compound **(±)-3al** (top) and enantioenriched **(+)-3al** (bottom).

SFC traces for compound 3am

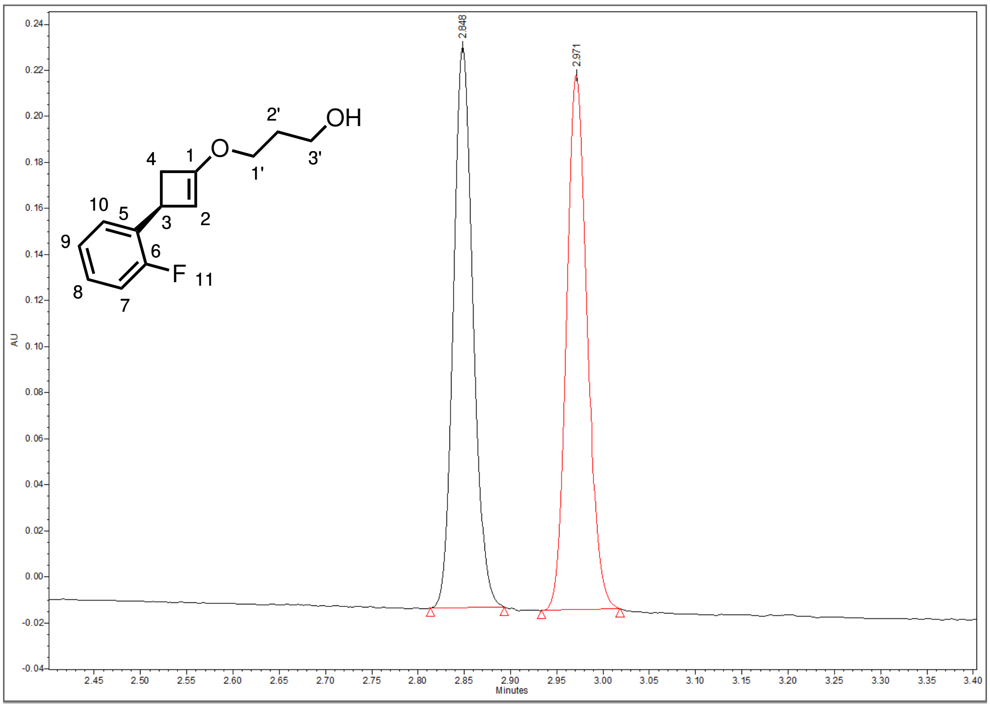

	Name	Retention Time (min)	Purity1 Angle	Purity1 Threshold	PDA/FLR Match1 Spect. Name	PDA/FLR Match1 Angle	PDA/FLR Match1 Threshold	PDA/FLR Match1 Lib. Name	Area (µV*sec)	% Area
1		2.848							361912	49.92
2		2.971							363127	50.08

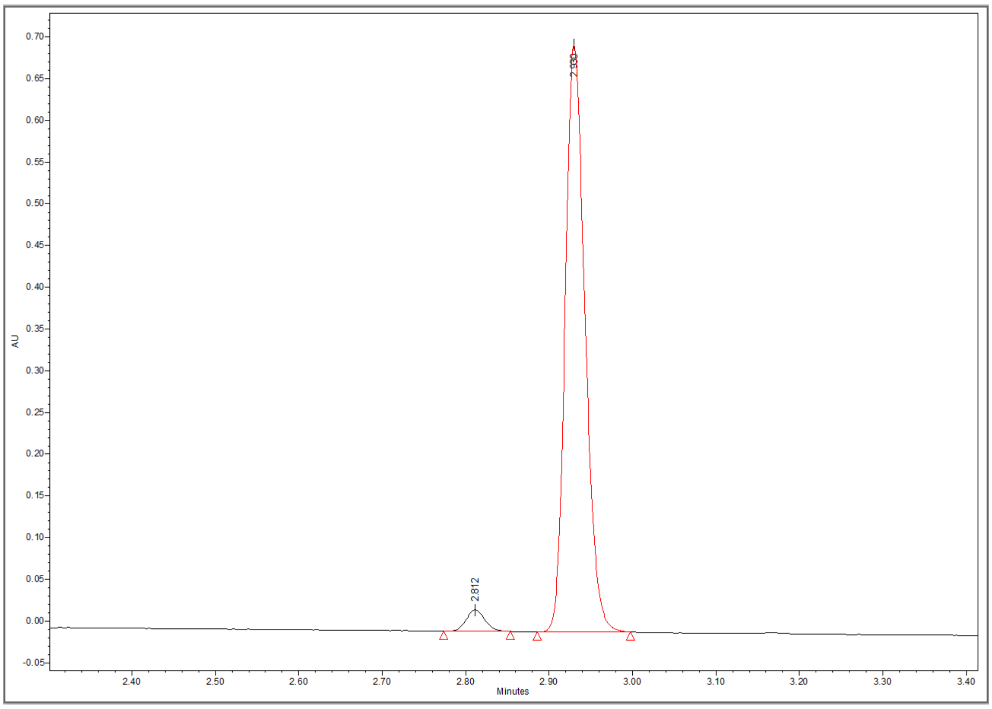

	Name	Retention Time (min)	Purity1 Angle	Purity1 Threshold	PDA/FLR Match1 Spect. Name	PDA/FLR Match1 Angle	PDA/FLR Match1 Threshold	PDA/FLR Match1 Lib. Name	Area (µV*sec)	% Area
1		2.812							39529	3.30
2		2.930							1158672	96.70

Figure 79: SFC traces for compound **(±)-3am** (top) and enantioenriched **(+)-3am** (bottom).

SFC traces for compound 3an

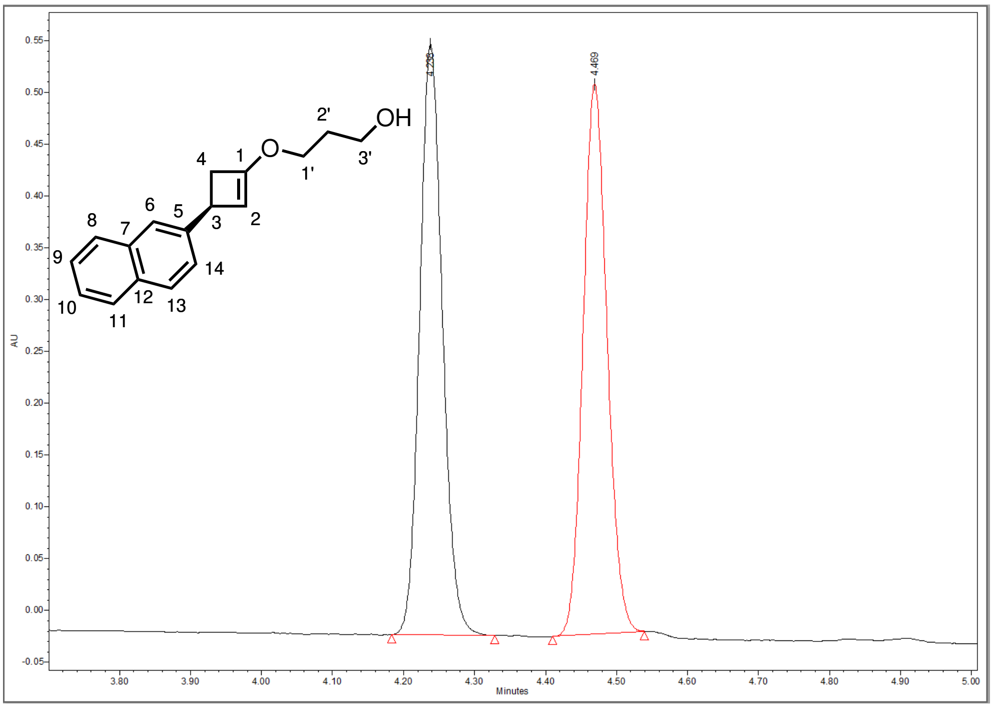

	Name	Retention Time (min)	Purity1 Angle	Purity1 Threshold	PDA/FLR Match1 Spect. Name	PDA/FLR Match1 Angle	PDA/FLR Match1 Threshold	PDA/FLR Match1 Lib. Name	Area (µV*sec)	% Area
1		4.238							1267562	50.45
2		4.469							1244969	49.55

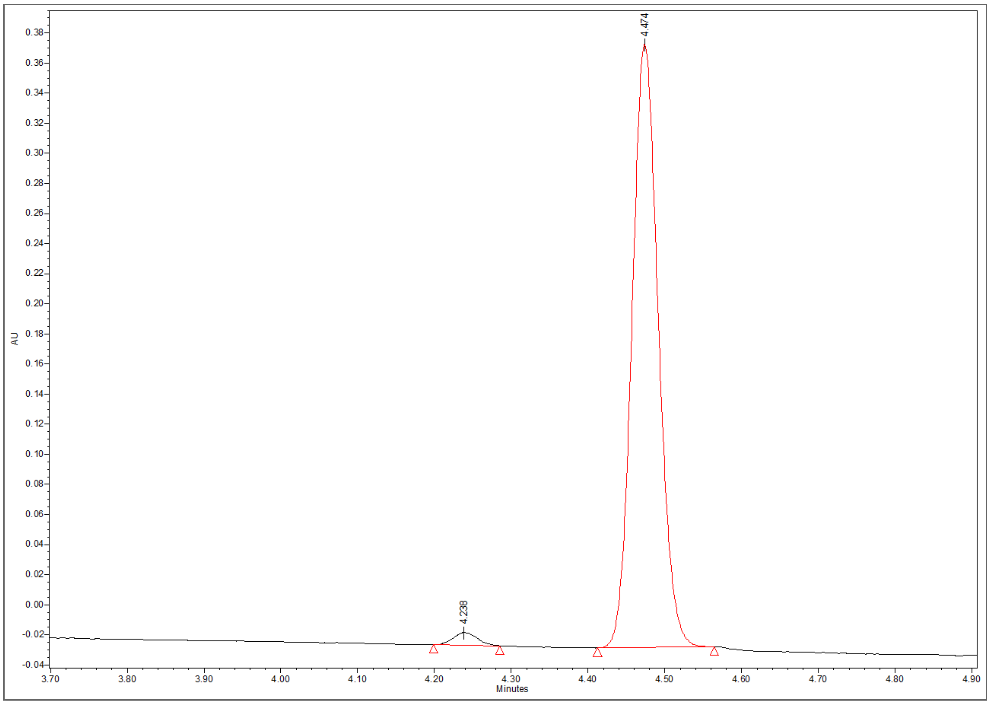

	Name	Retention Time (min)	Purity1 Angle	Purity1 Threshold	PDA/FLR Match1 Spect. Name	PDA/FLR Match1 Angle	PDA/FLR Match1 Threshold	PDA/FLR Match1 Lib. Name	Area (µV*sec)	% Area
1		4.238							18262	1.88
2		4.474							953453	98.12

Figure 80: SFC traces for compound **(±)-3an** (top) and enantioenriched **(+)-3an** (bottom).

SFC traces for compound 3ao

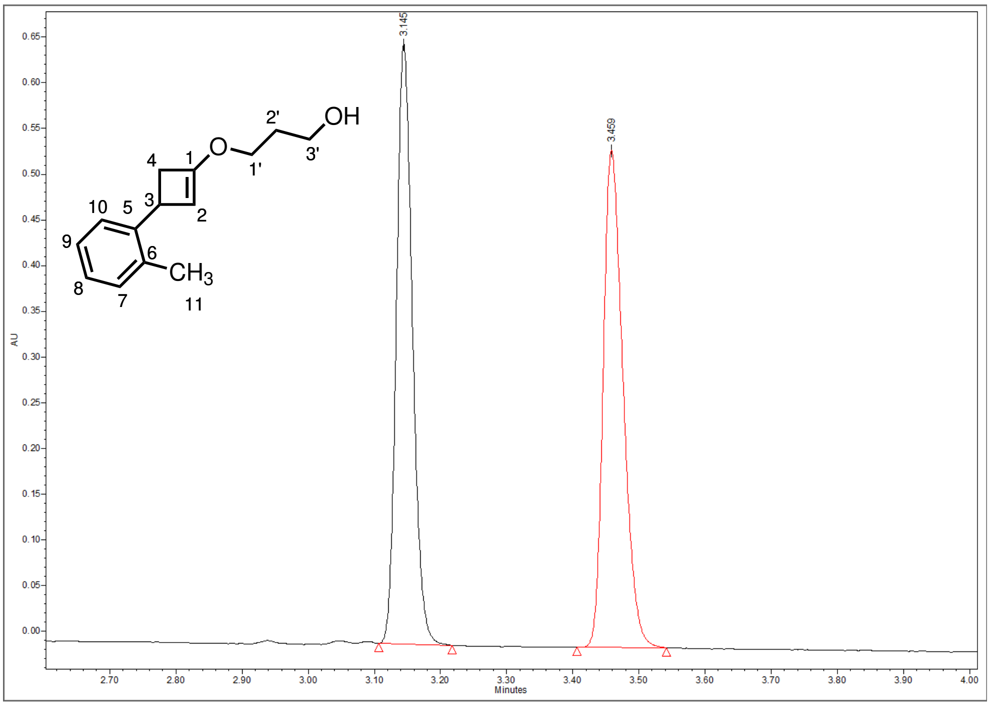

	Name	Retention Time (min)	Purity1 Angle	Purity1 Threshold	PDA/FLR Match1 Spect. Name	PDA/FLR Match1 Angle	PDA/FLR Match1 Threshold	PDA/FLR Match1 Lib. Name	Area (µV*sec)	% Area
1		3.145							1092474	49.19
2		3.459							1128633	50.81

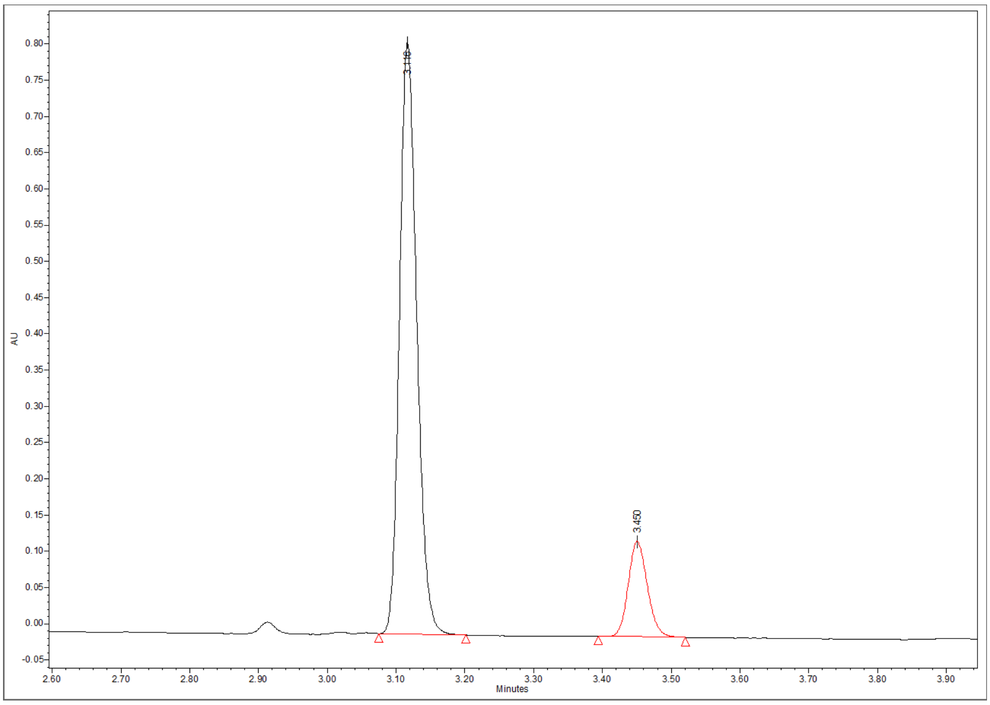

	Name	Retention Time (min)	Purity1 Angle	Purity1 Threshold	PDA/FLR Match1 Spect. Name	PDA/FLR Match1 Angle	PDA/FLR Match1 Threshold	PDA/FLR Match1 Lib. Name	Area (µV*sec)	% Area
1		3.116							1427499	84.97
2		3.450							252469	15.03

Figure 81: SFC traces for compound **(±)-3ao** (top) and enantioenriched **(−)-3ao** (bottom).

SFC traces for compound 3ap

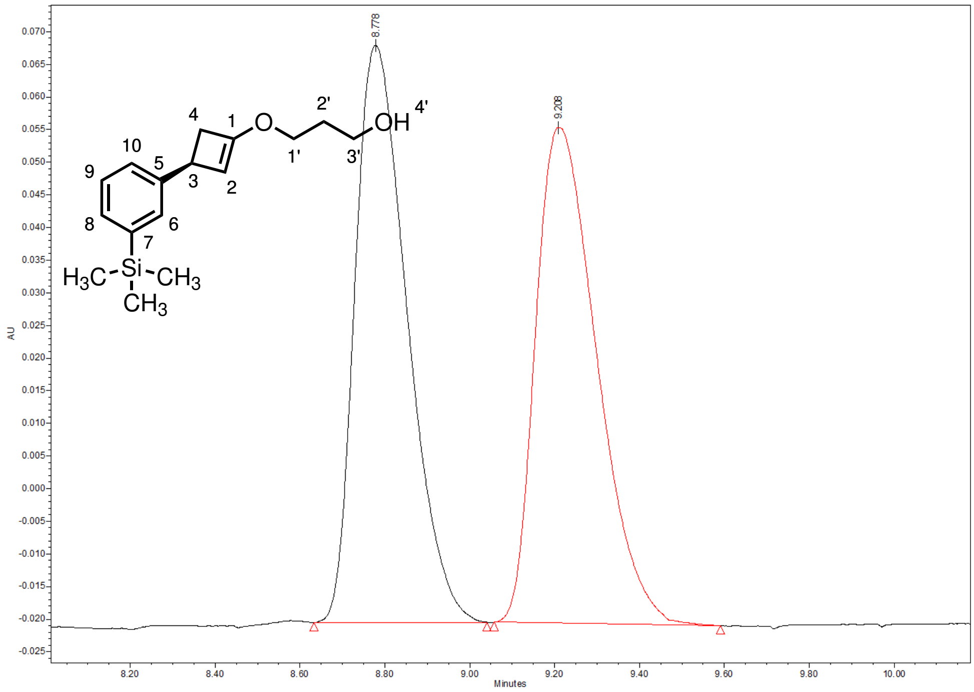

E	Name	Retention Time (min)	Purity1 Angle	Purity1 Threshold	PDA/FLR Match1 Spect. Name	PDA/FLR Match1 Angle	PDA/FLR Match1 Threshold	PDA/FLR Match1 Lib. Name	Area (μV*sec)	% Area
1		8.778							753717	49.83
2		9.208							758748	50.17

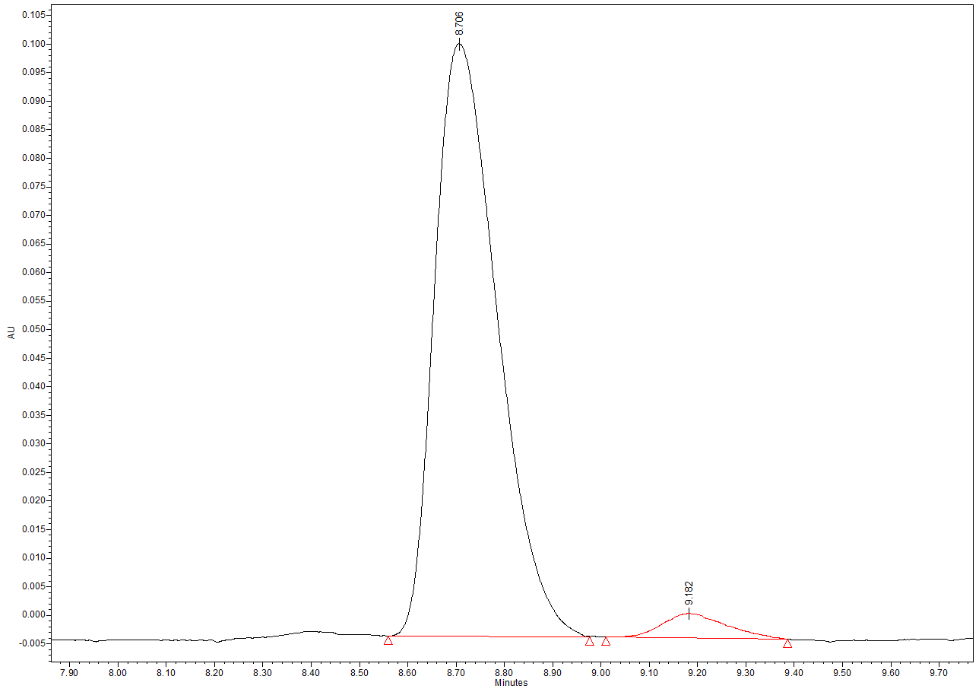

E	Name	Retention Time (min)	Purity1 Angle	Purity1 Threshold	PDA/FLR Match1 Spect. Name	PDA/FLR Match1 Angle	PDA/FLR Match1 Threshold	PDA/FLR Match1 Lib. Name	Area (μV*sec)	% Area
1		8.706							936316	96.02
2		9.182							38842	3.98

Figure 82: SFC traces for compound **(±)-3ap** (top) and enantioenriched **(+)-3ap** (bottom).

SFC traces for compound 3aq

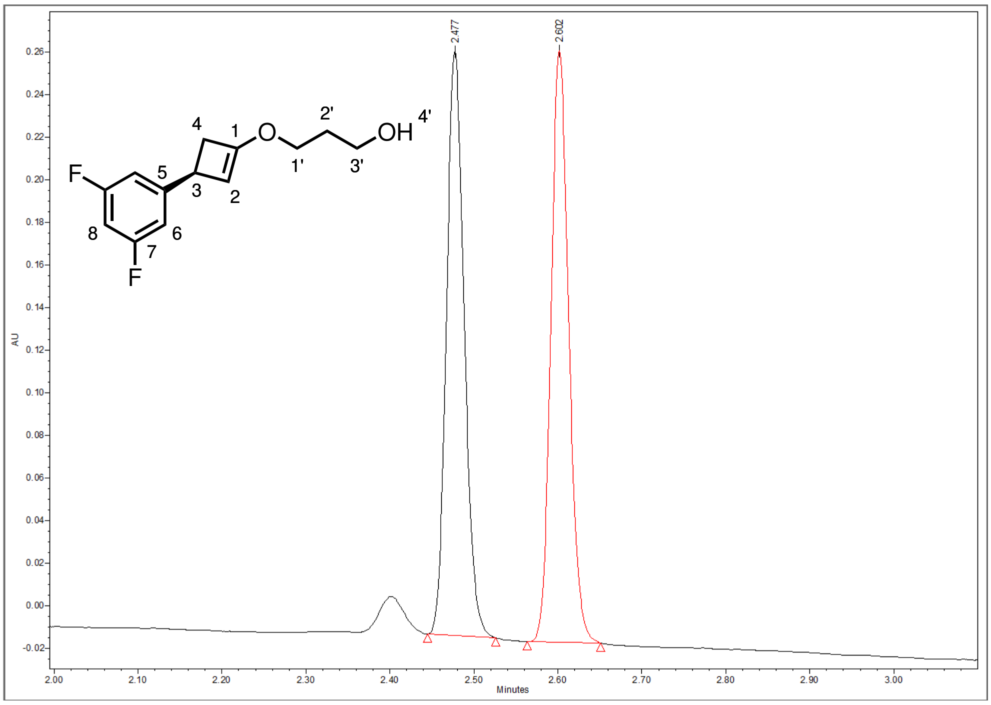

	Name	Retention Time (min)	Purity1 Angle	Purity1 Threshold	PDA/FLR Match1 Spect. Name	PDA/FLR Match1 Angle	PDA/FLR Match1 Threshold	PDA/FLR Match1 Lib. Name	Area (μV*sec)	% Area
1		2.477							403794	49.60
2		2.602							410306	50.40

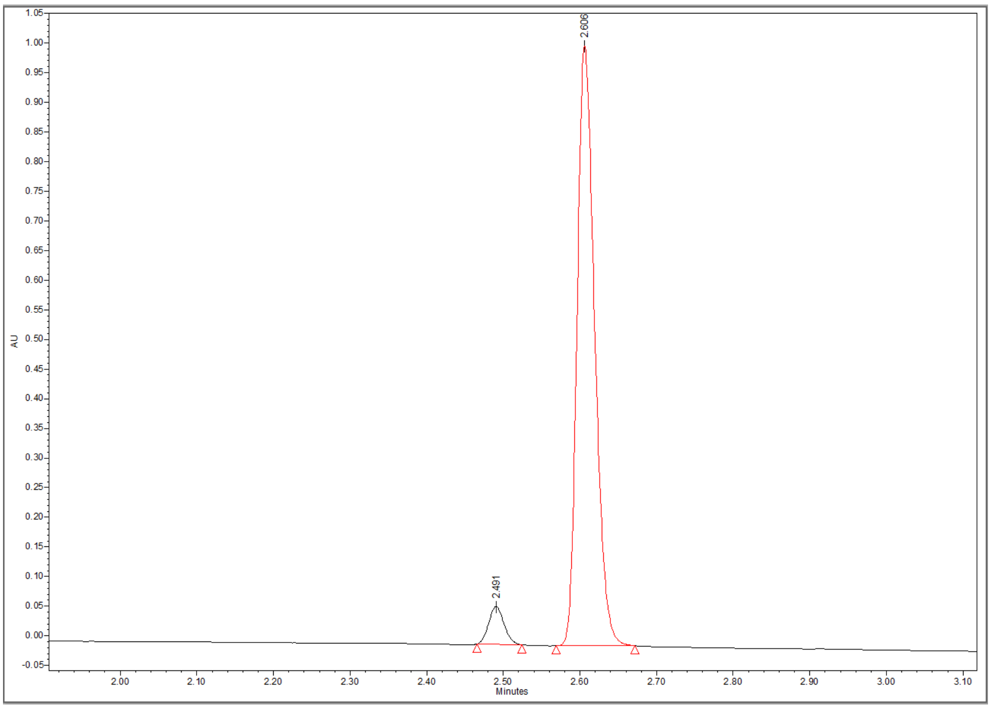

	Name	Retention Time (min)	Purity1 Angle	Purity1 Threshold	PDA/FLR Match1 Spect. Name	PDA/FLR Match1 Angle	PDA/FLR Match1 Threshold	PDA/FLR Match1 Lib. Name	Area (μV*sec)	% Area
1		2.491							88715	5.23
2		2.606							1607675	94.77

Figure 83: SFC traces for compound **(±)-3aq** (top) and enantioenriched **(+)-3aq** (bottom).

SFC traces for compound 3ar

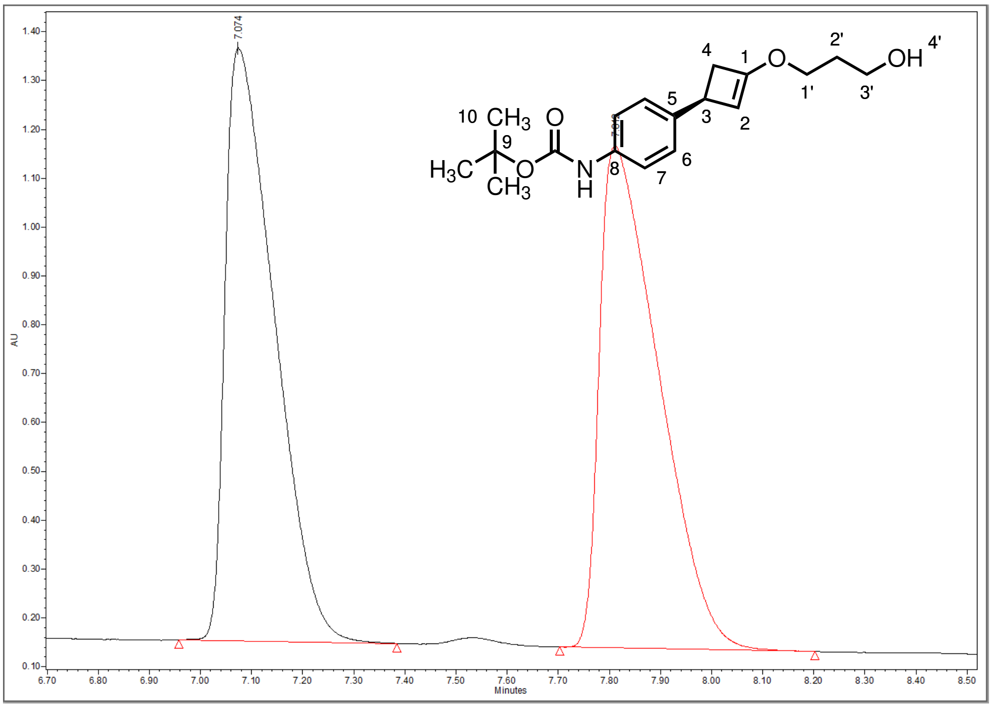

	Name	Retention Time (min)	Purity1 Angle	Purity1 Threshold	PDA/FLR Match1 Spect. Name	PDA/FLR Match1 Angle	PDA/FLR Match1 Threshold	PDA/FLR Match1 Lib. Name	Area (µV*sec)	% Area
1		7.074							8100508	49.50
2		7.812							8262920	50.50

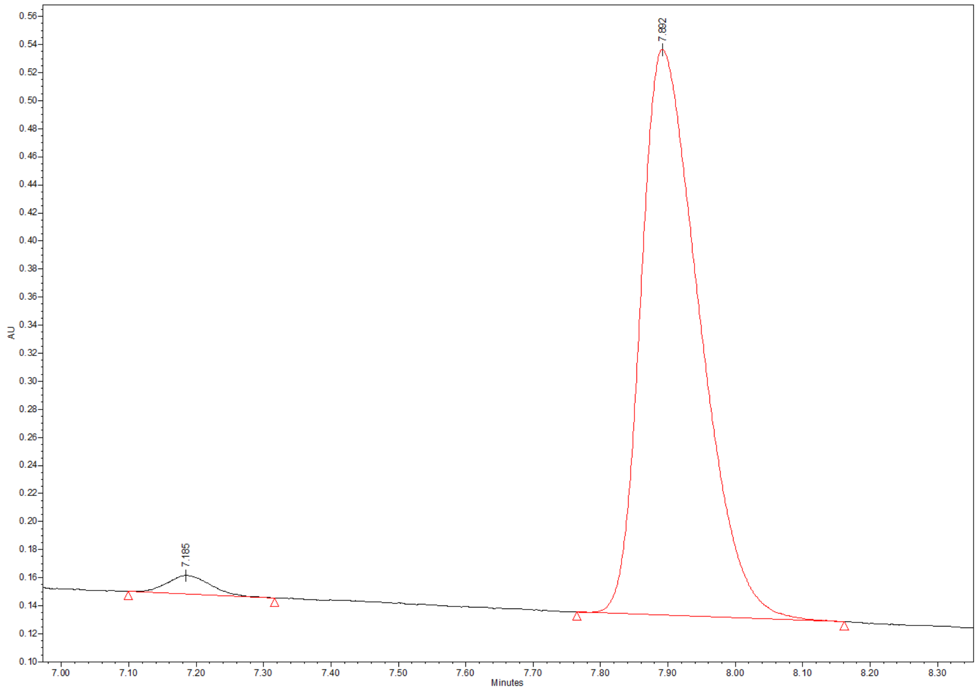

	Name	Retention Time (min)	Purity1 Angle	Purity1 Threshold	PDA/FLR Match1 Spect. Name	PDA/FLR Match1 Angle	PDA/FLR Match1 Threshold	PDA/FLR Match1 Lib. Name	Area (µV*sec)	% Area
1		7.185							59509	2.39
2		7.892							2427407	97.61

Figure 84: SFC traces for compound **(±)-3ar** (top) and enantioenriched **(+)-3ar** (bottom).

SFC traces for compound 3as

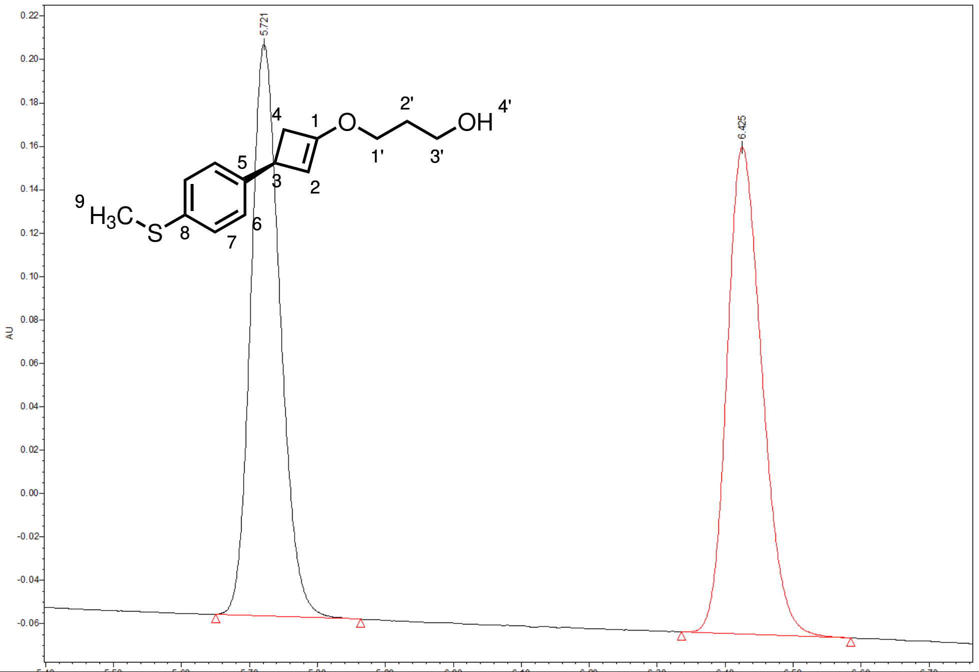

	Name	Retention Time (min)	Purity1 Angle	Purity1 Threshold	PDA/FLR Match1 Spect. Name	PDA/FLR Match1 Angle	PDA/FLR Match1 Threshold	PDA/FLR Match1 Lib. Name	Area (µV*sec)	% Area
1		5.721							789671	49.63
2		6.425							801472	50.37

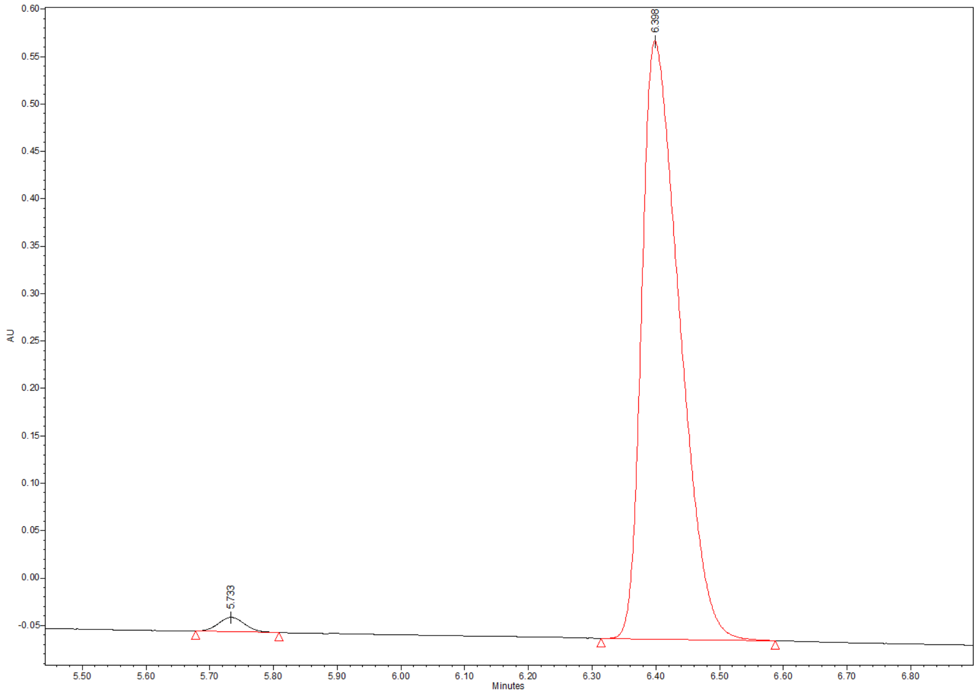

	Name	Retention Time (min)	Purity1 Angle	Purity1 Threshold	PDA/FLR Match1 Spect. Name	PDA/FLR Match1 Angle	PDA/FLR Match1 Threshold	PDA/FLR Match1 Lib. Name	Area (µV*sec)	% Area
1		5.733							43123	1.64
2		6.398							2588451	98.36

Figure 85: SFC traces for compound **(±)-3as** (top) and enantioenriched **(+)-3as** (bottom).

SFC traces for compound 3at

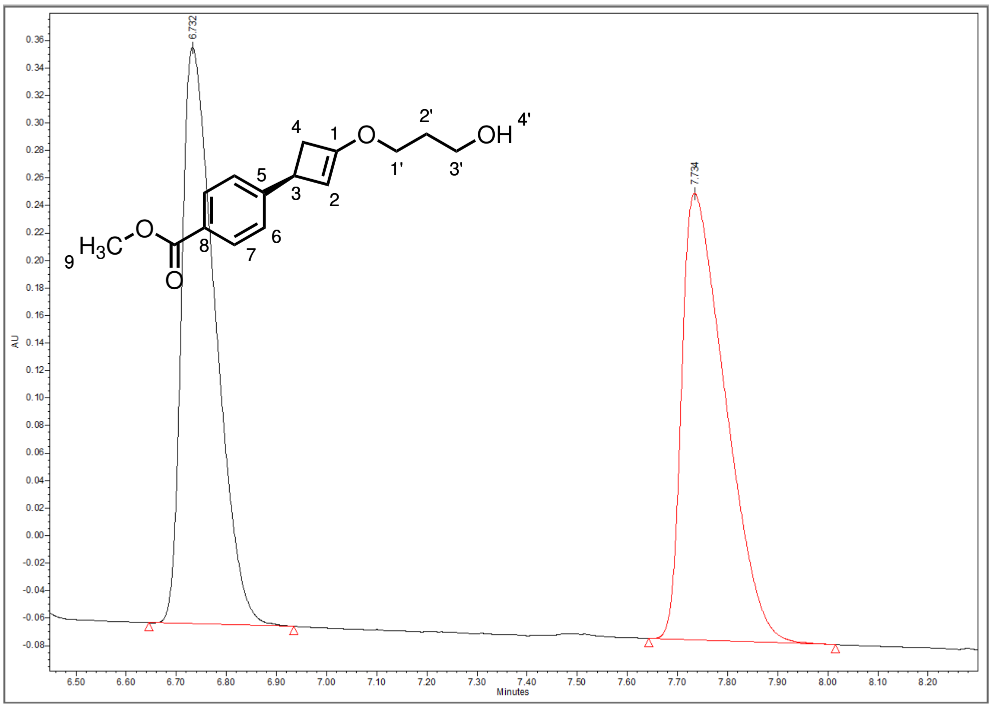

	Name	Retention Time (min)	Purity1 Angle	Purity1 Threshold	PDA/FLR Match1 Spect. Name	PDA/FLR Match1 Angle	PDA/FLR Match1 Threshold	PDA/FLR Match1 Lib. Name	Area (µV*sec)	% Area
1		6.732							1942506	49.80
2		7.734							1958167	50.20

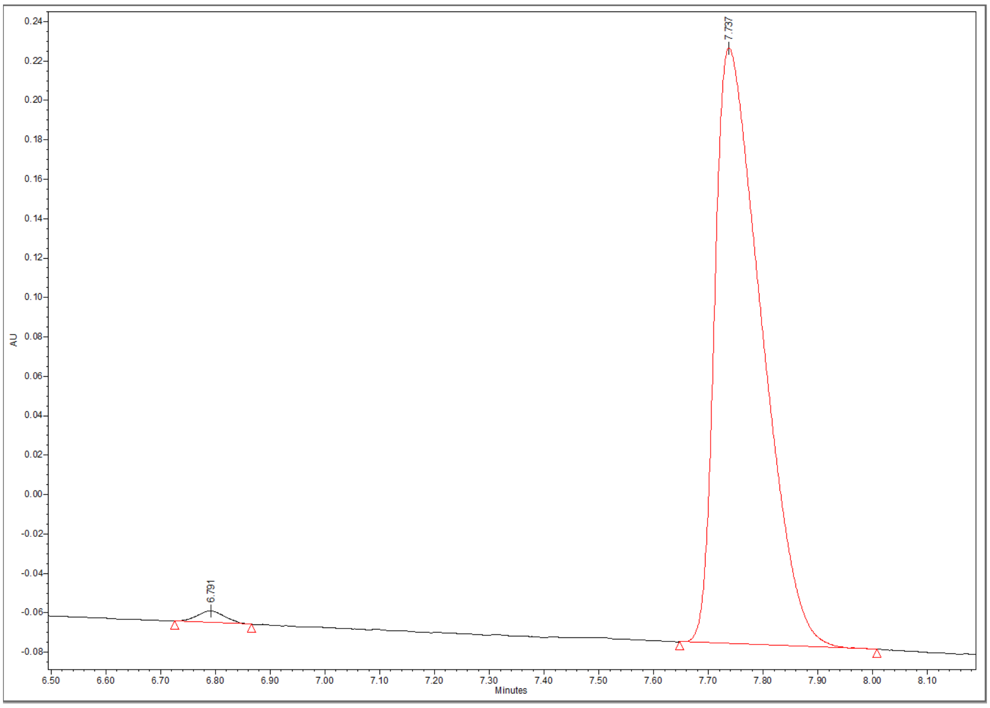

	Name	Retention Time (min)	Purity1 Angle	Purity1 Threshold	PDA/FLR Match1 Spect. Name	PDA/FLR Match1 Angle	PDA/FLR Match1 Threshold	PDA/FLR Match1 Lib. Name	Area (µV*sec)	% Area
1		6.791							20381	1.13
2		7.737							1778655	98.87

Figure 86: SFC traces for compound **(±)-3at** (top) and enantioenriched **(+)-3tq** (bottom).

SFC traces for compound 3au

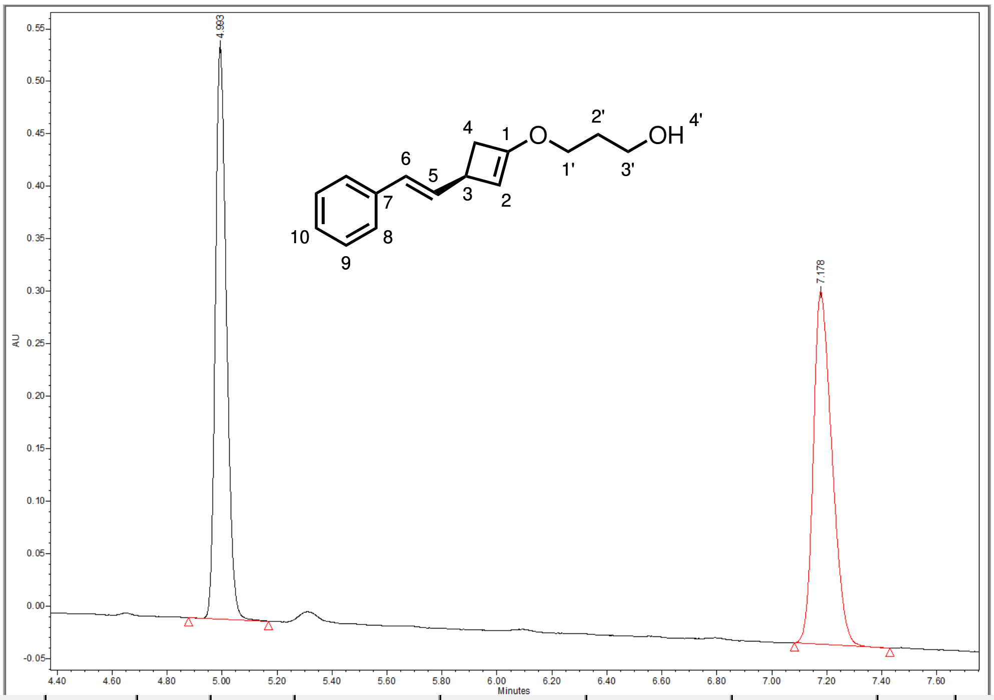

	Name	Retention Time (min)	Purity1 Angle	Purity1 Threshold	PDA/FLR Match1 Spect. Name	PDA/FLR Match1 Angle	PDA/FLR Match1 Threshold	PDA/FLR Match1 Lib. Name	Area (µV*sec)	% Area
1		4.993							1526749	49.10
2		7.178							1582714	50.90

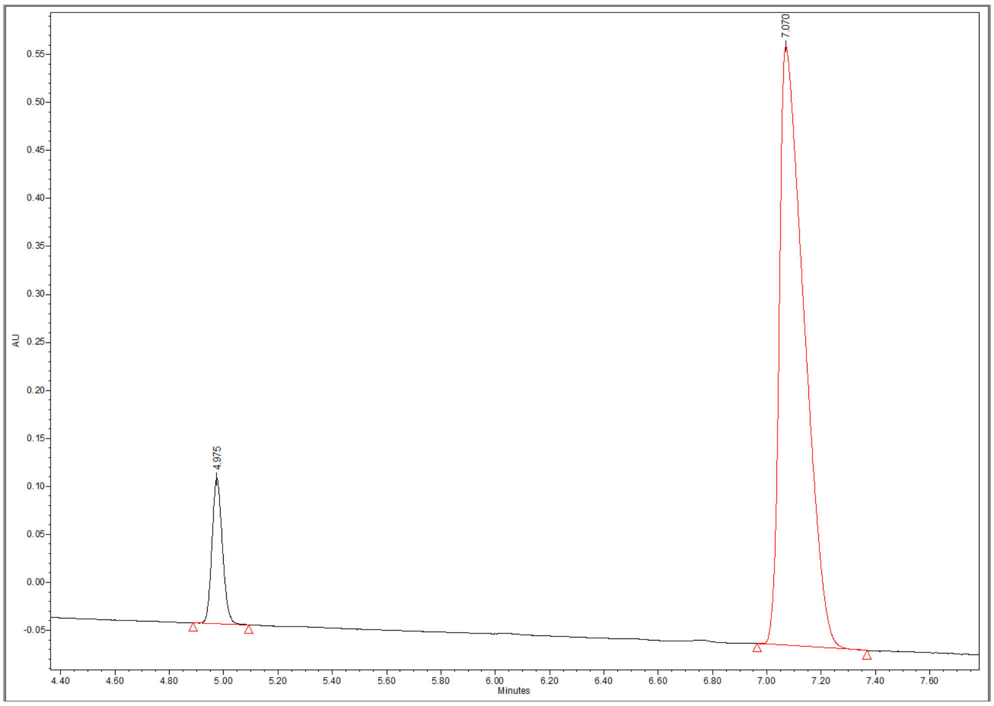

	Name	Retention Time (min)	Purity1 Angle	Purity1 Threshold	PDA/FLR Match1 Spect. Name	PDA/FLR Match1 Angle	PDA/FLR Match1 Threshold	PDA/FLR Match1 Lib. Name	Area (µV*sec)	% Area
1		4.975							403764	9.29
2		7.070							3941869	90.71

Figure 87: SFC traces for compound **(±)-3au** (top) and enantioenriched **(+)-3au** (bottom).

SFC traces for compound 3av

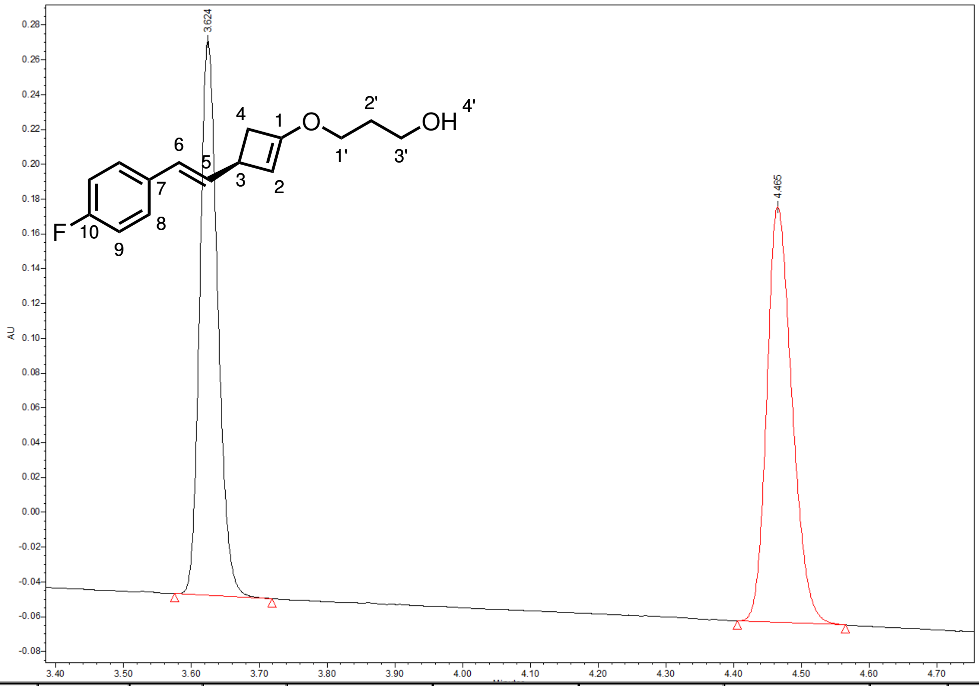

	Name	Retention Time (min)	Purity1 Angle	Purity1 Threshold	PDA/FLR Match1 Spect. Name	PDA/FLR Match1 Angle	PDA/FLR Match1 Threshold	PDA/FLR Match1 Lib. Name	Area (μV*sec)	% Area
1		3.624							591365	48.89
2		4.465							618336	51.11

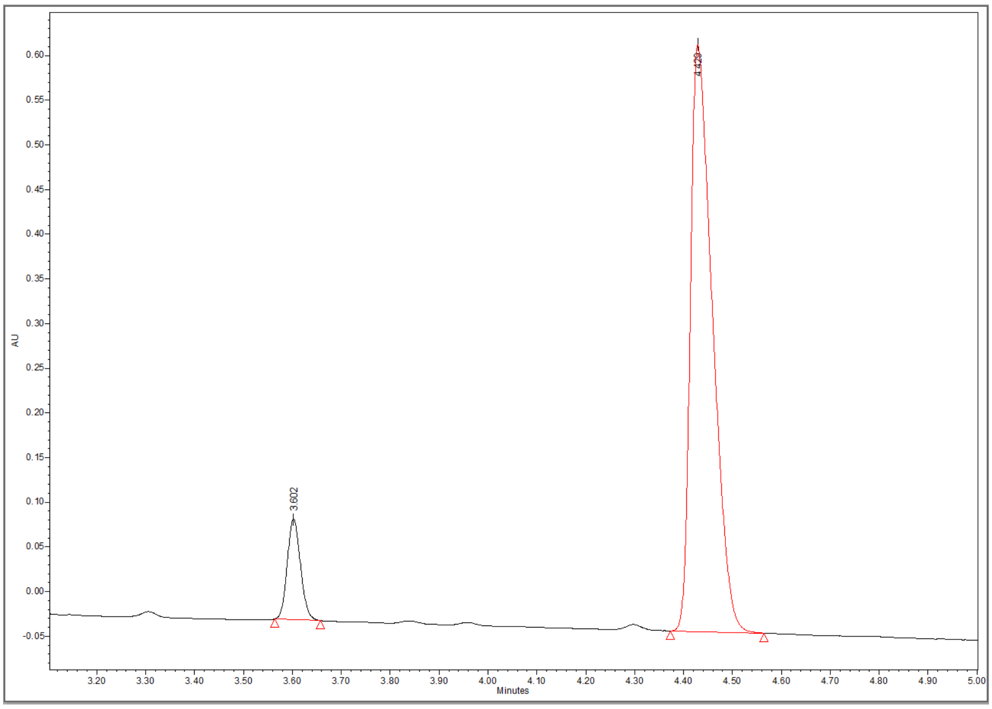

	Name	Retention Time (min)	Purity1 Angle	Purity1 Threshold	PDA/FLR Match1 Spect. Name	PDA/FLR Match1 Angle	PDA/FLR Match1 Threshold	PDA/FLR Match1 Lib. Name	Area (μV*sec)	% Area
1		3.602							211899	9.37
2		4.429							2050199	90.63

Figure 88: SFC traces for compound **(±)-3av** (top) and enantioenriched **(+)-3av** (bottom).

SFC traces for compound 3aw

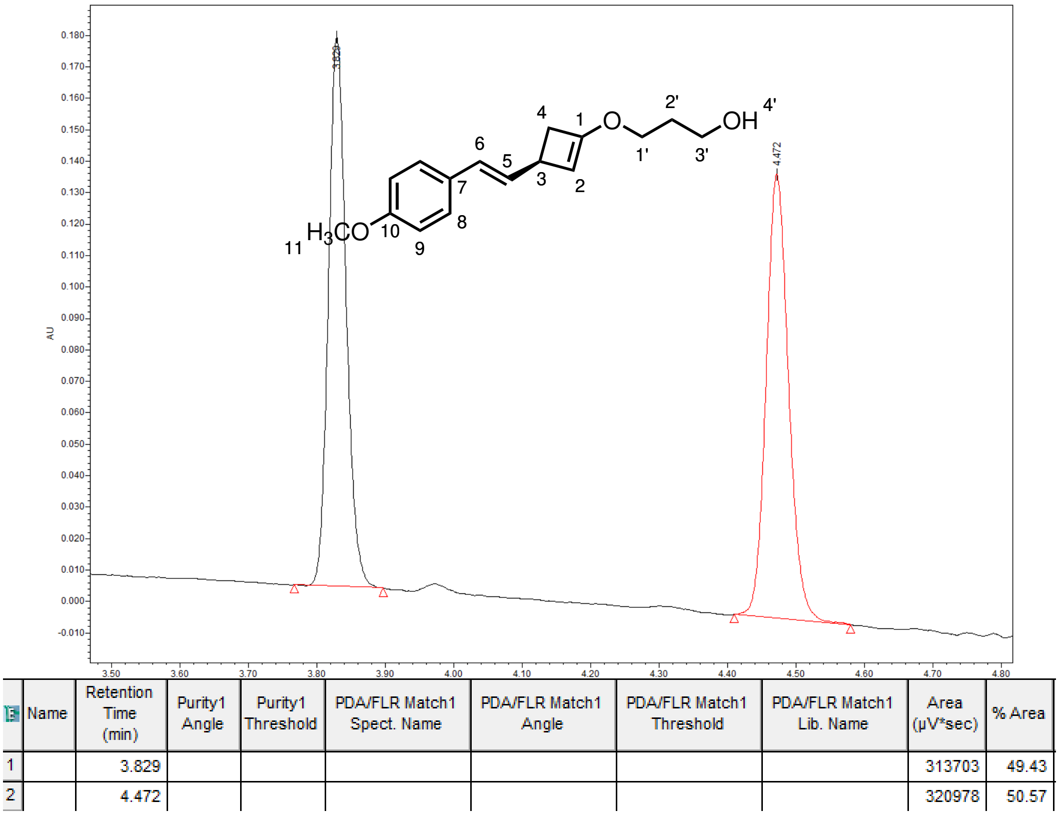

	Name	Retention Time (min)	Purity1 Angle	Purity1 Threshold	PDA/FLR Match1 Spect. Name	PDA/FLR Match1 Angle	PDA/FLR Match1 Threshold	PDA/FLR Match1 Lib. Name	Area (µV*sec)	% Area
1		3.829							313703	49.43
2		4.472							320978	50.57

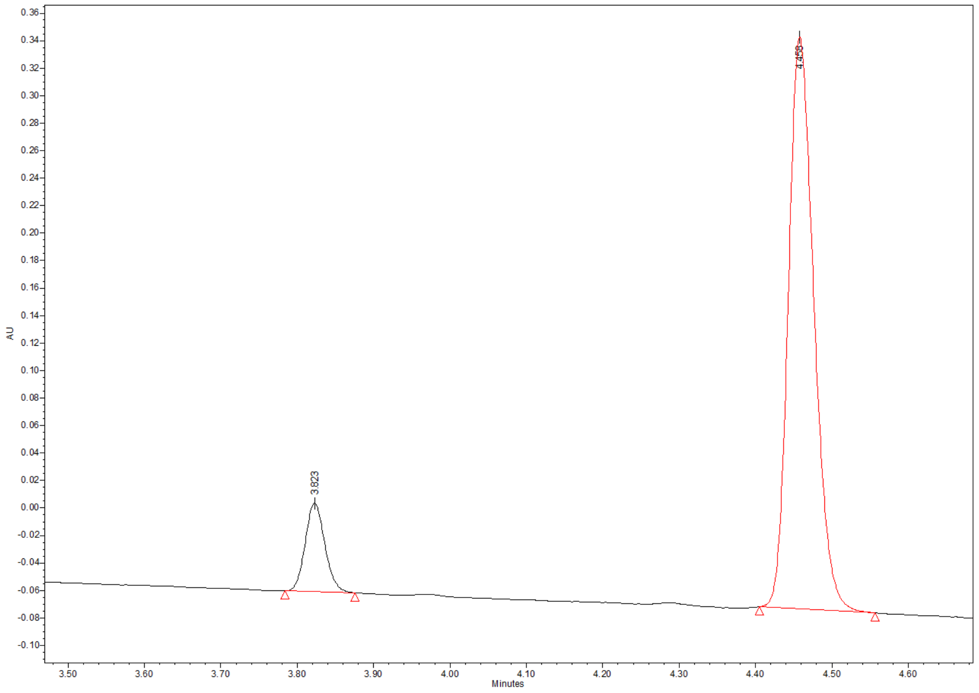

	Name	Retention Time (min)	Purity1 Angle	Purity1 Threshold	PDA/FLR Match1 Spect. Name	PDA/FLR Match1 Angle	PDA/FLR Match1 Threshold	PDA/FLR Match1 Lib. Name	Area (µV*sec)	% Area
1		3.823							115575	10.63
2		4.458							971181	89.37

Figure 89: SFC traces for compound **(±)-3aw** (top) and enantioenriched **(+)-3aw** (bottom).

SFC traces for compound 3ba

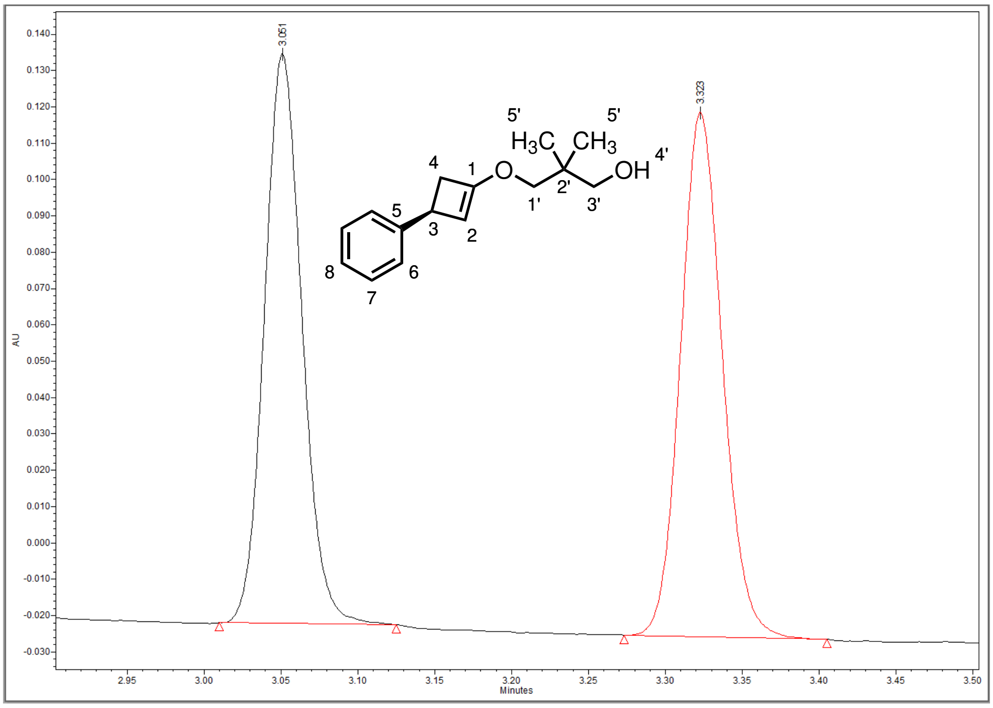

	Name	Retention Time (min)	Purity1 Angle	Purity1 Threshold	PDA/FLR Match1 Spect. Name	PDA/FLR Match1 Angle	PDA/FLR Match1 Threshold	PDA/FLR Match1 Lib. Name	Area (µV*sec)	% Area
1		3.051							266157	49.35
2		3.323							273155	50.65

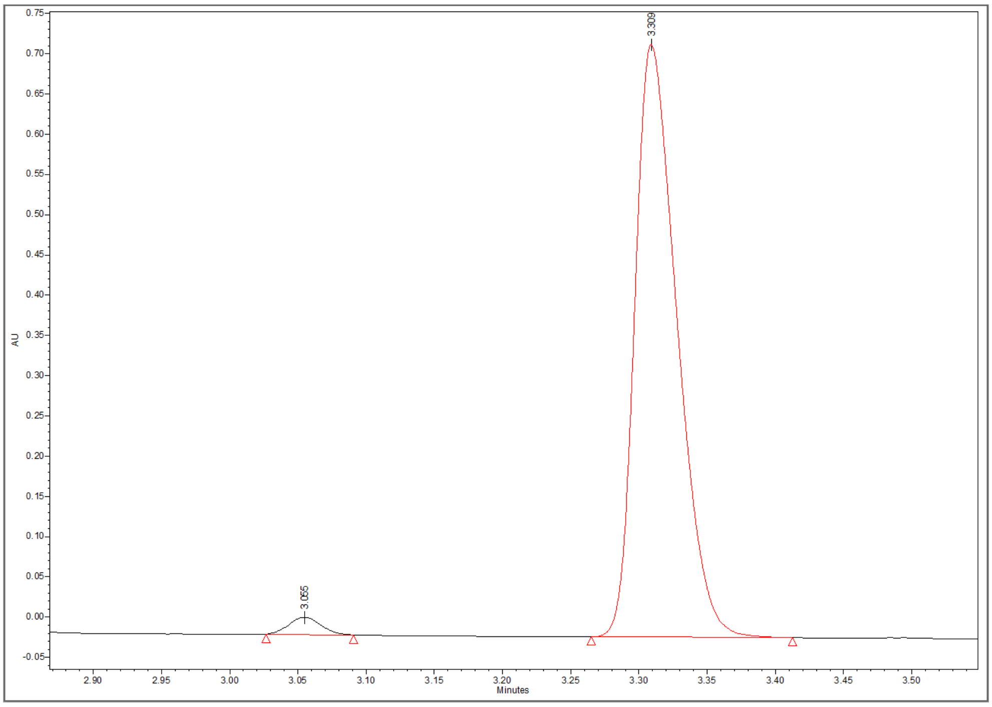

	Name	Retention Time (min)	Purity1 Angle	Purity1 Threshold	PDA/FLR Match1 Spect. Name	PDA/FLR Match1 Angle	PDA/FLR Match1 Threshold	PDA/FLR Match1 Lib. Name	Area (µV*sec)	% Area
1		3.055							34104	2.12
2		3.309							1572564	97.88

Figure 90: SFC traces for compound **(±)-3ba** (top) and enantioenriched **(+)-3ba** (bottom).

SFC traces for compound 3bb

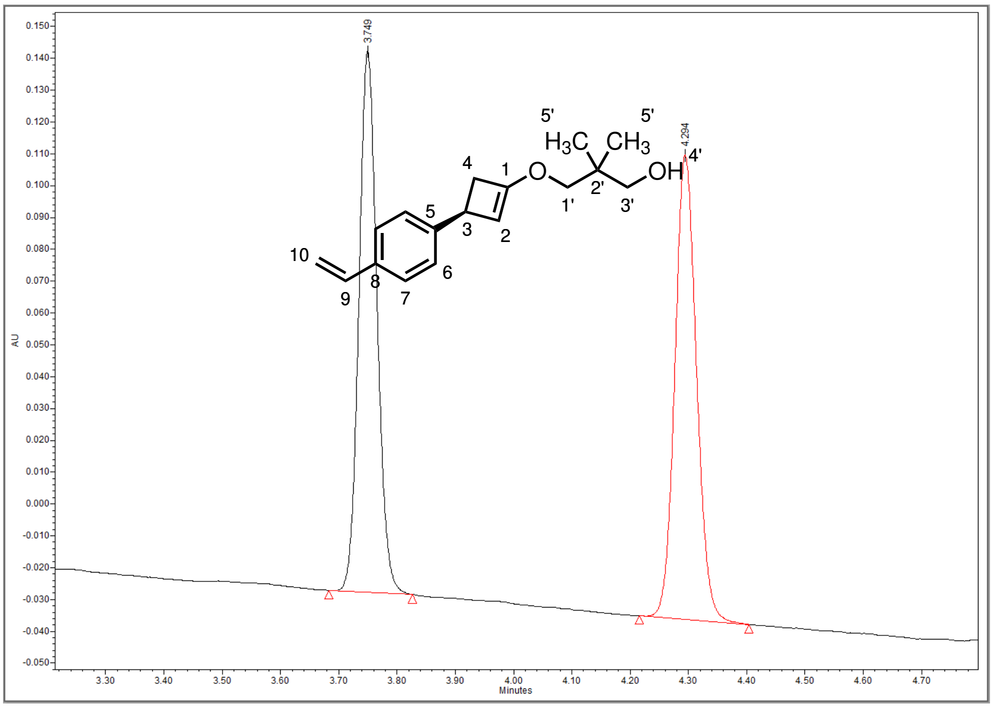

	Name	Retention Time (min)	Purity1 Angle	Purity1 Threshold	PDA/FLR Match1 Spect. Name	PDA/FLR Match1 Angle	PDA/FLR Match1 Threshold	PDA/FLR Match1 Lib. Name	Area (µV*sec)	% Area
1		3.749							366831	49.39
2		4.294							375935	50.61

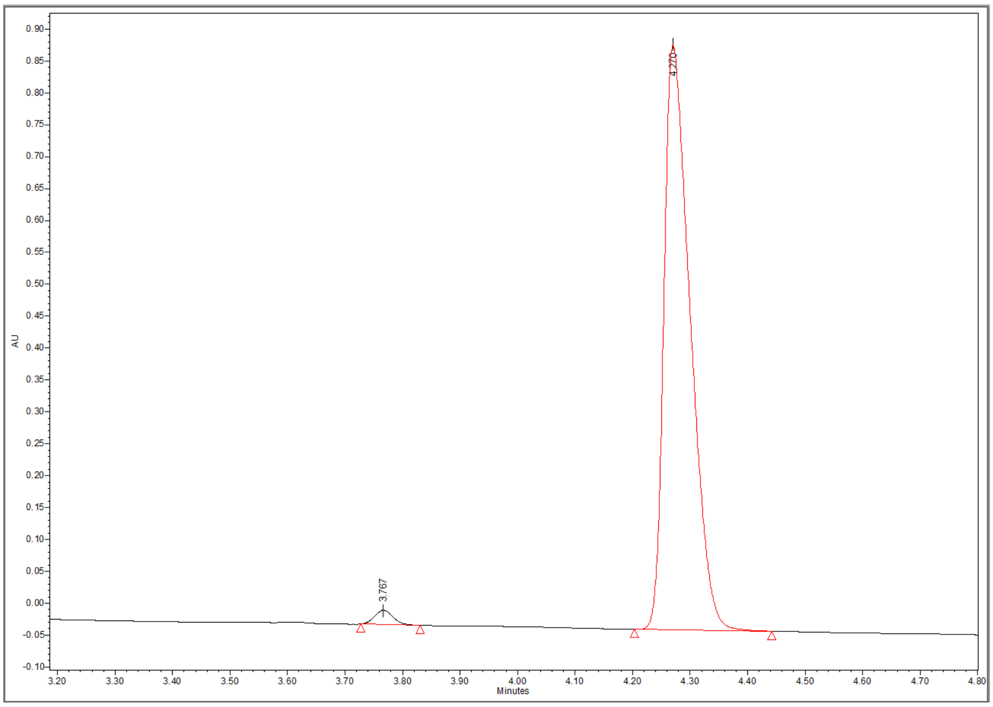

	Name	Retention Time (min)	Purity1 Angle	Purity1 Threshold	PDA/FLR Match1 Spect. Name	PDA/FLR Match1 Angle	PDA/FLR Match1 Threshold	PDA/FLR Match1 Lib. Name	Area (µV*sec)	% Area
1		3.767							49432	1.68
2		4.270							2896705	98.32

Figure 91: SFC traces for compound **(±)-3bb** (top) and enantioenriched **(+)-3bb** (bottom).

SFC traces for compound 3bc

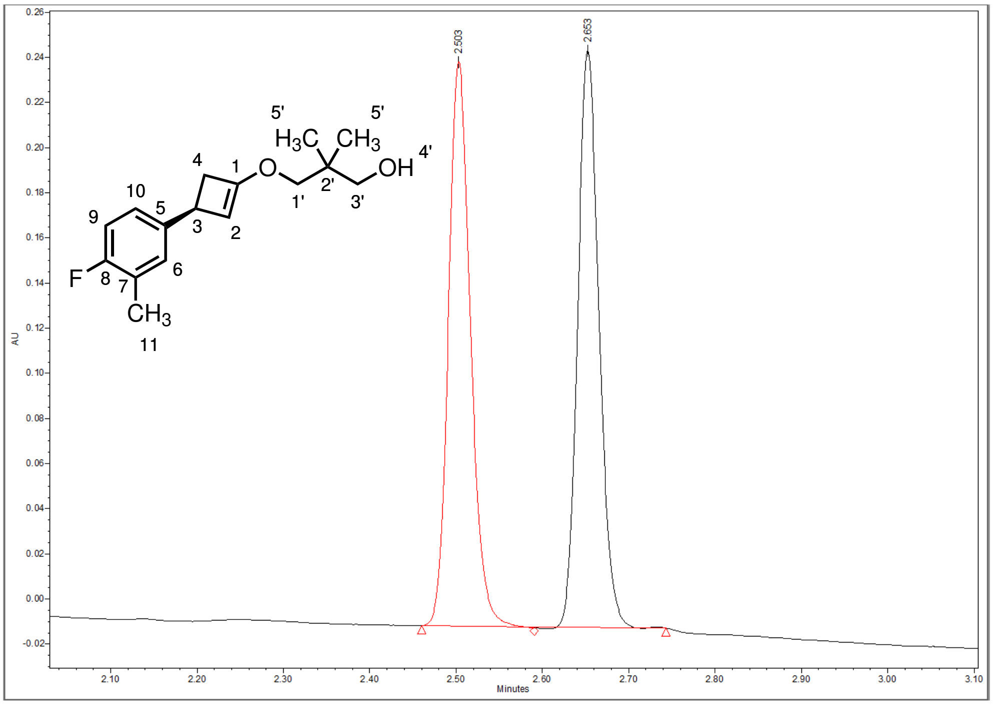

	Name	Retention Time (min)	Purity1 Angle	Purity1 Threshold	PDA/FLR Match1 Spect. Name	PDA/FLR Match1 Angle	PDA/FLR Match1 Threshold	PDA/FLR Match1 Lib. Name	Area (µV*sec)	% Area
1		2.503							446483	49.84
2		2.653							449264	50.16

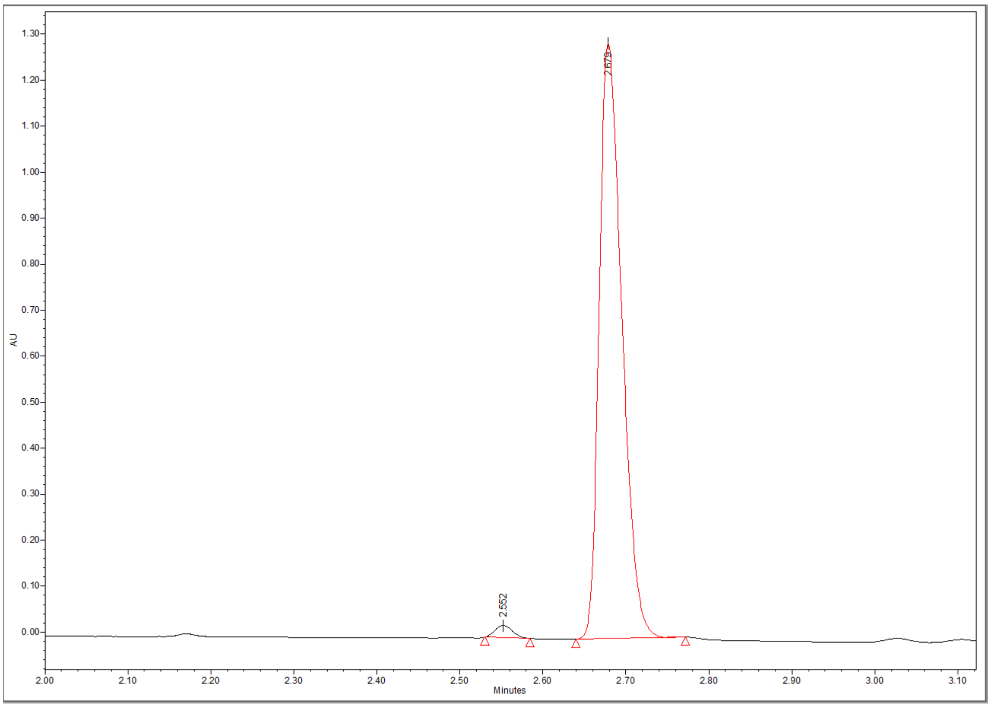

	Name	Retention Time (min)	Purity1 Angle	Purity1 Threshold	PDA/FLR Match1 Spect. Name	PDA/FLR Match1 Angle	PDA/FLR Match1 Threshold	PDA/FLR Match1 Lib. Name	Area (µV*sec)	% Area
1		2.552							39137	1.57
2		2.679							2454121	98.43

Figure 92: SFC traces for compound **(±)-3bc** (top) and enantioenriched **(+)-3bc** (bottom).

SFC traces for compound 3ca

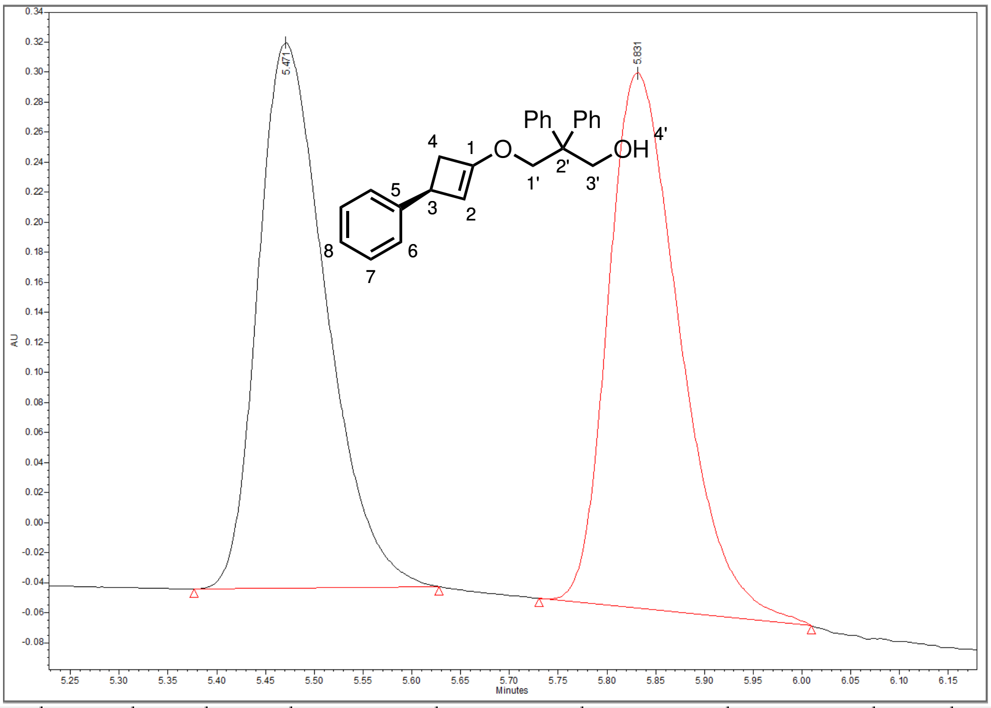

	Name	Retention Time (min)	Purity1 Angle	Purity1 Threshold	PDA/FLR Match1 Spect. Name	PDA/FLR Match1 Angle	PDA/FLR Match1 Threshold	PDA/FLR Match1 Lib. Name	Area (µV*sec)	% Area
1		5.471							1763419	49.10
2		5.831							1827805	50.90

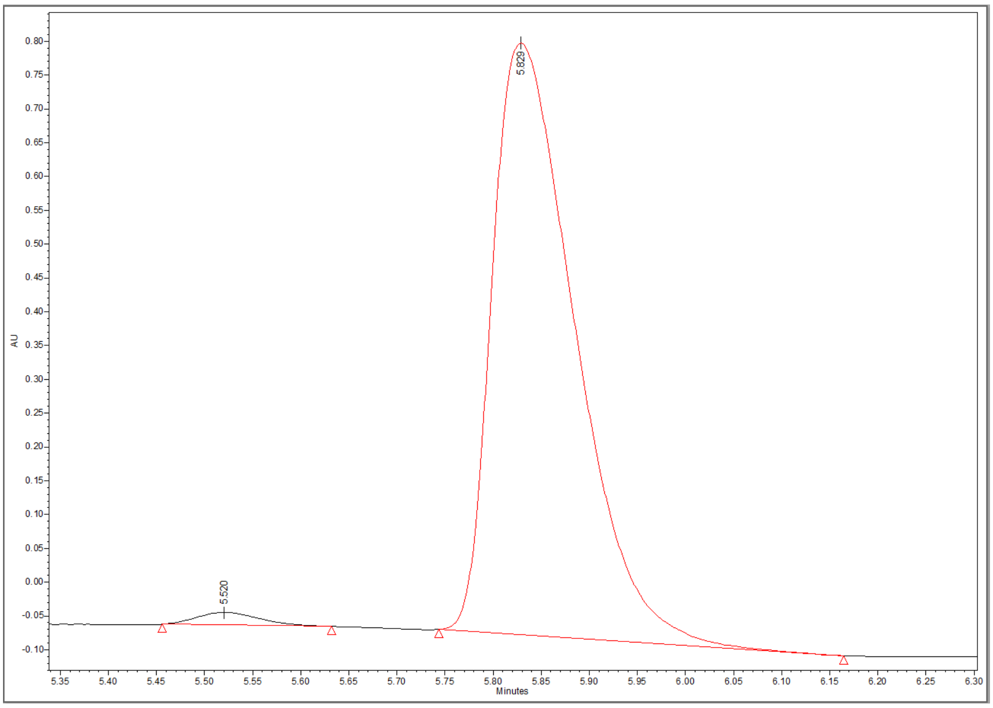

	Name	Retention Time (min)	Purity1 Angle	Purity1 Threshold	PDA/FLR Match1 Spect. Name	PDA/FLR Match1 Angle	PDA/FLR Match1 Threshold	PDA/FLR Match1 Lib. Name	Area (µV*sec)	% Area
1		5.520							82825	1.56
2		5.829							5231720	98.44

Figure 93: SFC traces for compound **(±)-3ca** (top) and enantioenriched **(+)-3ca** (bottom).

SFC traces for compound 3cb

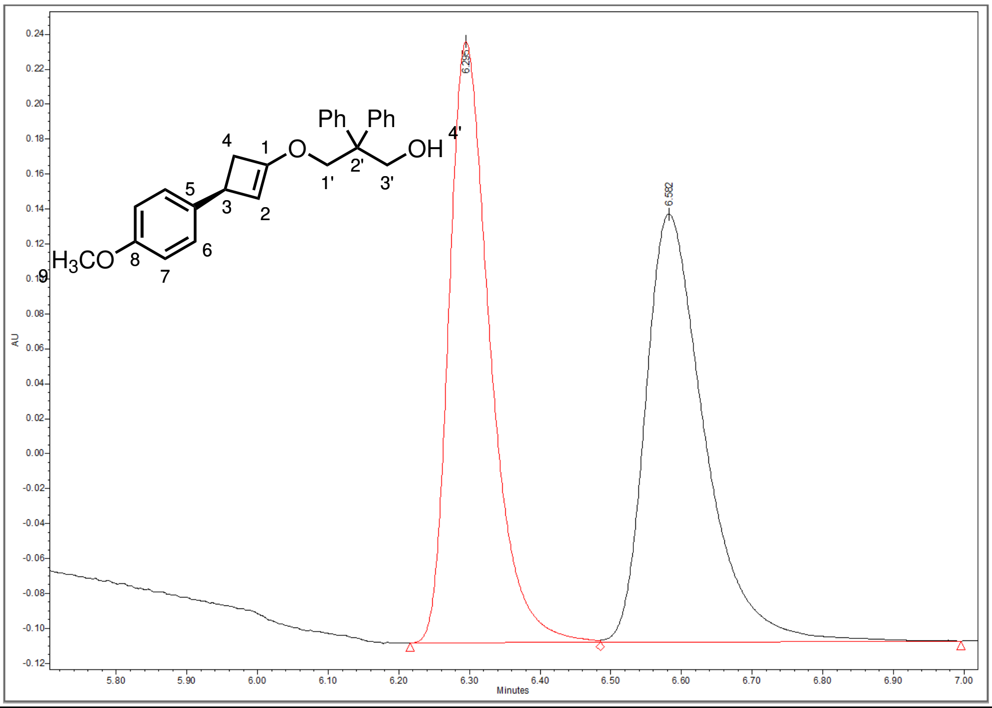

Name	Retention Time (min)	Purity1 Angle	Purity1 Threshold	PDA/FLR Match1 Spect. Name	PDA/FLR Match1 Angle	PDA/FLR Match1 Threshold	PDA/FLR Match1 Lib. Name	Area (µV*sec)	% Area
1	6.295							1390802	49.44
2	6.582							1422142	50.56

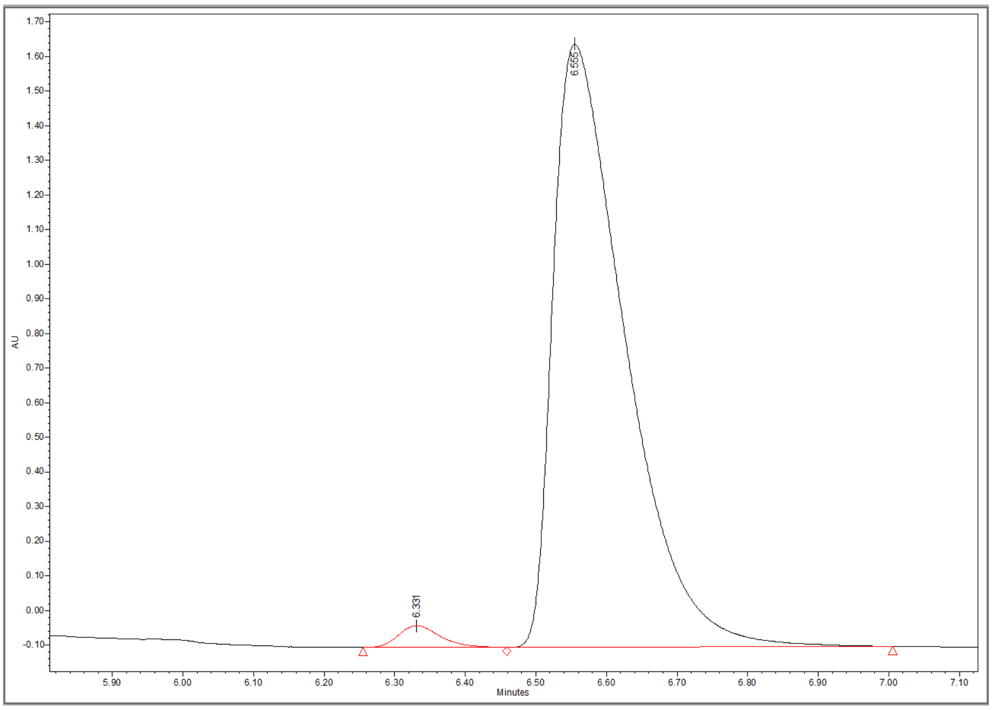

Name	Retention Time (min)	Purity1 Angle	Purity1 Threshold	PDA/FLR Match1 Spect. Name	PDA/FLR Match1 Angle	PDA/FLR Match1 Threshold	PDA/FLR Match1 Lib. Name	Area (µV*sec)	% Area
1	6.331							256191	2.03
2	6.555							12357931	97.97

Figure 94: SFC traces for compound **(±)-3cb** (top) and enantioenriched **(+)-3cb** (bottom).

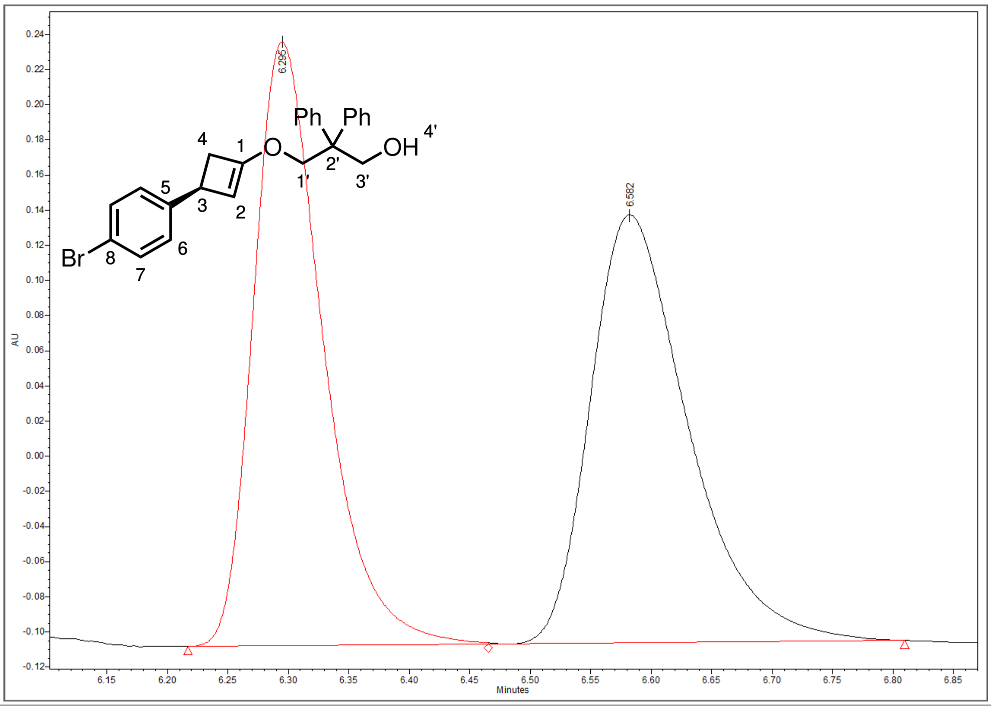

E	Name	Retention Time (min)	Purity1 Angle	Purity1 Threshold	PDA/FLR Match1 Spect. Name	PDA/FLR Match1 Angle	PDA/FLR Match1 Threshold	PDA/FLR Match1 Lib. Name	Area (µV*sec)	% Area
1		6.295							1380600	50.16
2		6.582							1371737	49.84

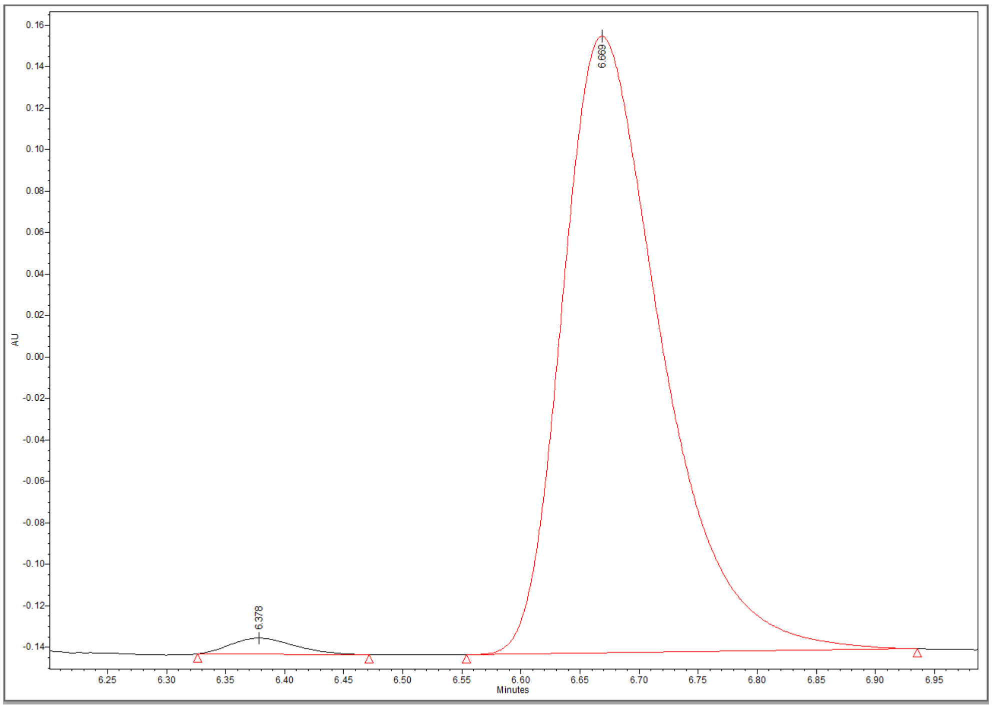

E	Name	Retention Time (min)	Purity1 Angle	Purity1 Threshold	PDA/FLR Match1 Spect. Name	PDA/FLR Match1 Angle	PDA/FLR Match1 Threshold	PDA/FLR Match1 Lib. Name	Area (µV*sec)	% Area
1		6.378							28978	1.64
2		6.669							1736401	98.36

Figure 95: SFC traces for compound **(±)-3cc** (top) and enantioenriched **(+)-3cc** (bottom).

SFC traces for compound 3da

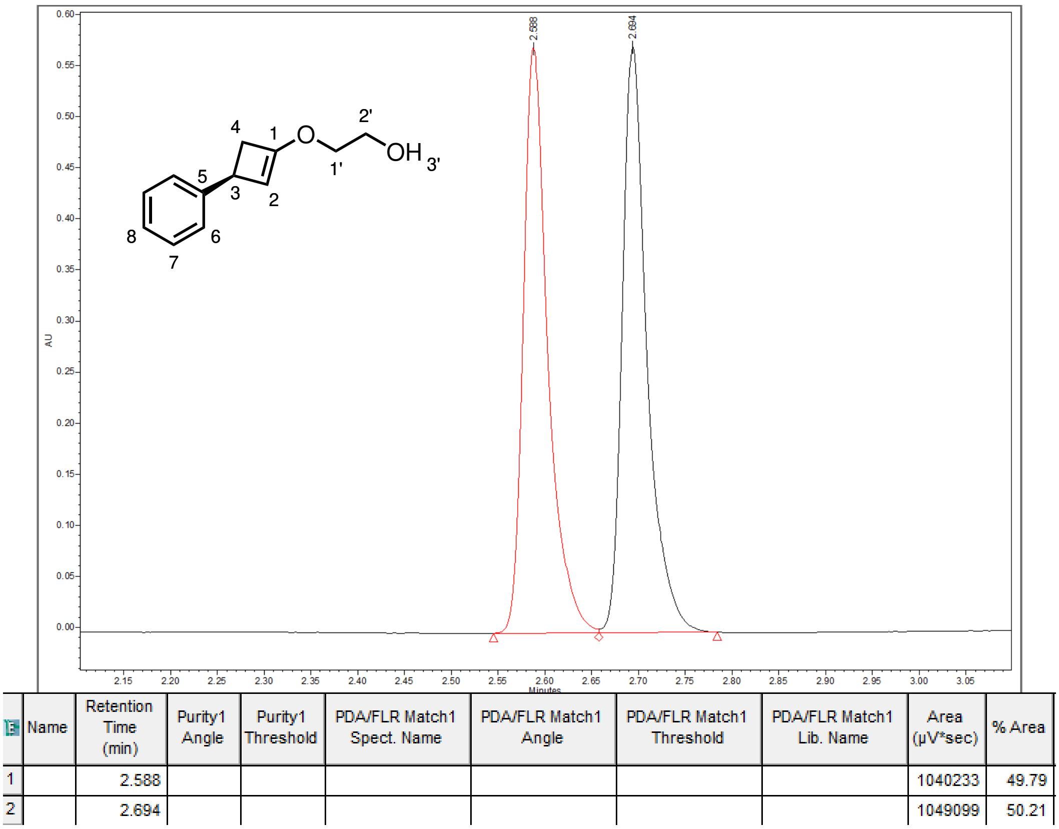

	Name	Retention Time (min)	Purity1 Angle	Purity1 Threshold	PDA/FLR Match1 Spect. Name	PDA/FLR Match1 Angle	PDA/FLR Match1 Threshold	PDA/FLR Match1 Lib. Name	Area (µV*sec)	% Area
1		2.588							1040233	49.79
2		2.694							1049099	50.21

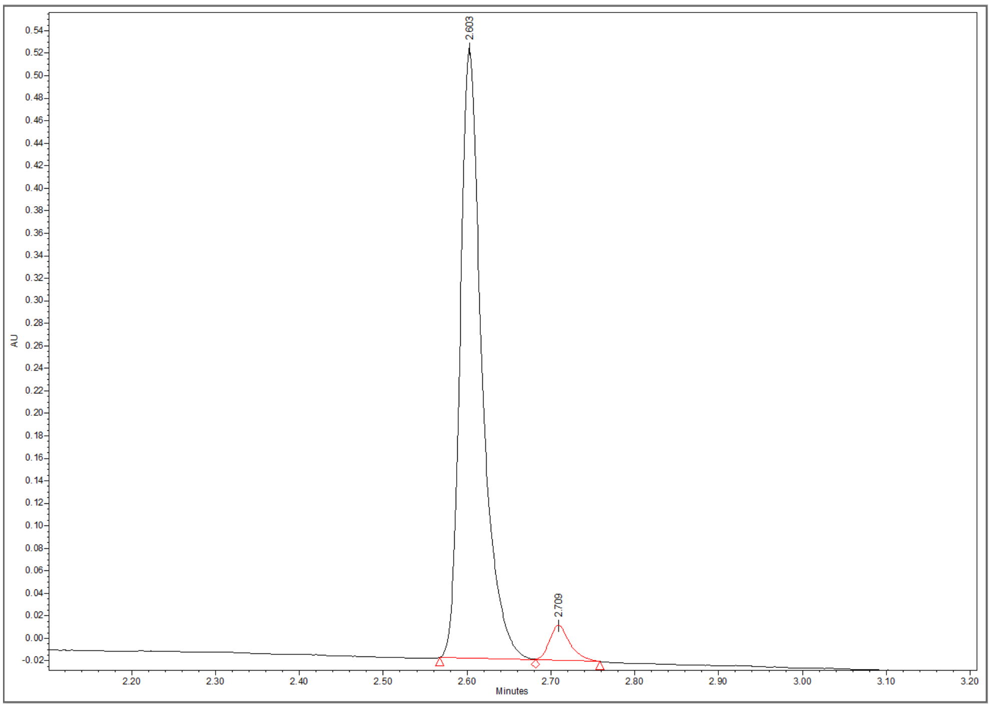

	Name	Retention Time (min)	Purity1 Angle	Purity1 Threshold	PDA/FLR Match1 Spect. Name	PDA/FLR Match1 Angle	PDA/FLR Match1 Threshold	PDA/FLR Match1 Lib. Name	Area (µV*sec)	% Area
1		2.603							979431	94.89
2		2.709							52727	5.11

Figure 96: SFC traces for compound **(±)-3da** (top) and enantioenriched **(+)-3da** (bottom).

SFC traces for compound 3db

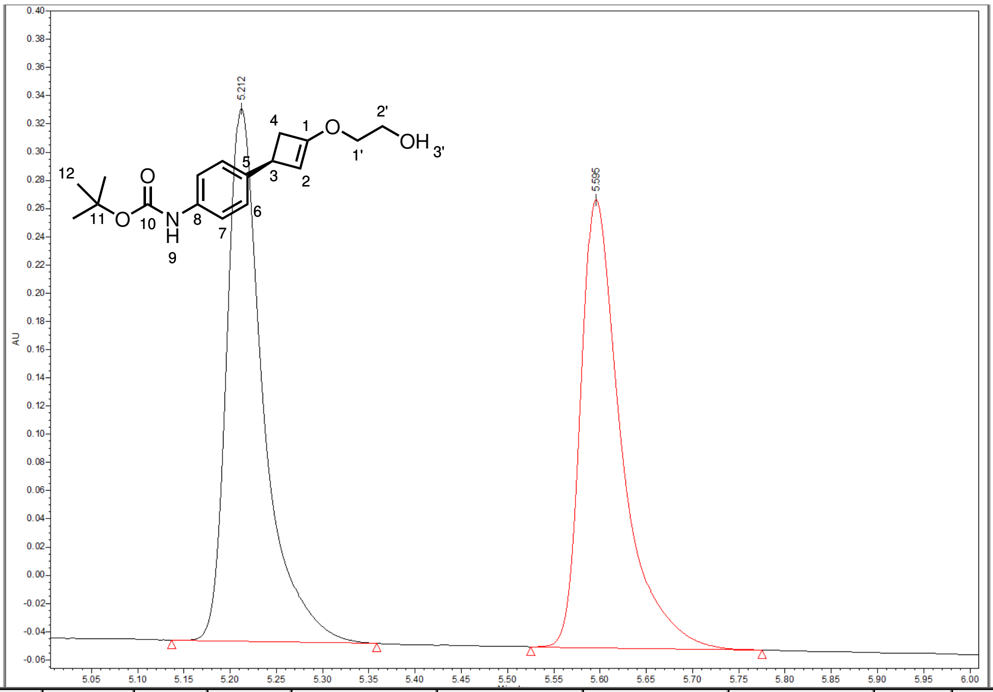

	Name	Retention Time (min)	Purity1 Angle	Purity1 Threshold	PDA/FLR Match1 Spect. Name	PDA/FLR Match1 Angle	PDA/FLR Match1 Threshold	PDA/FLR Match1 Lib. Name	Area (µV*sec)	% Area
1		5.212							1027485	51.41
2		5.595							970937	48.59

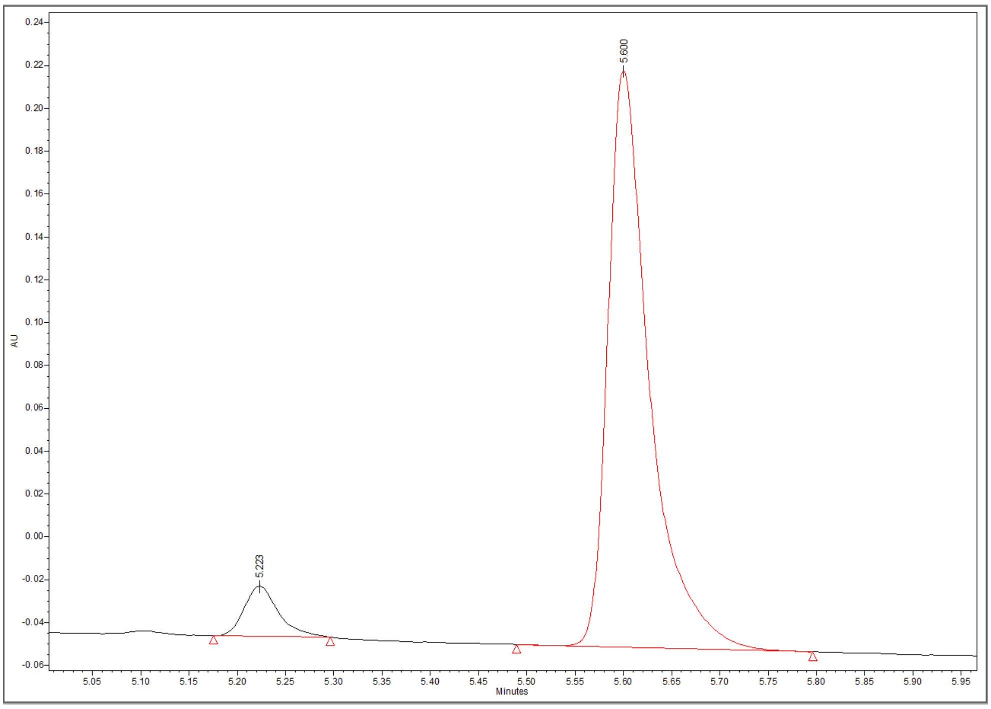

	Name	Retention Time (min)	Purity1 Angle	Purity1 Threshold	PDA/FLR Match1 Spect. Name	PDA/FLR Match1 Angle	PDA/FLR Match1 Threshold	PDA/FLR Match1 Lib. Name	Area (µV*sec)	% Area
1		5.223							57559	6.65
2		5.600							807770	93.35

Figure 97: SFC traces for compound **(±)-3db** (top) and enantioenriched **(+)-3db** (bottom).

SFC traces for compound 3dc

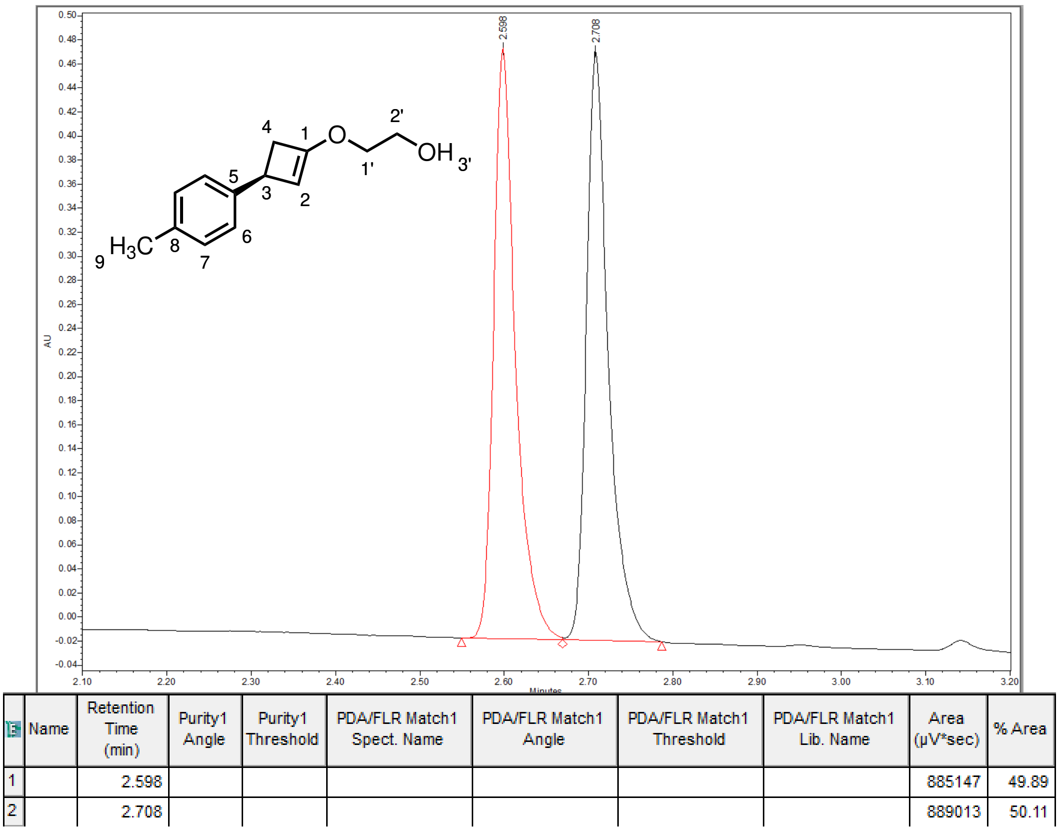

E	Name	Retention Time (min)	Purity1 Angle	Purity1 Threshold	PDA/FLR Match1 Spect. Name	PDA/FLR Match1 Angle	PDA/FLR Match1 Threshold	PDA/FLR Match1 Lib. Name	Area (µV*sec)	% Area
1		2.598							885147	49.89
2		2.708							889013	50.11

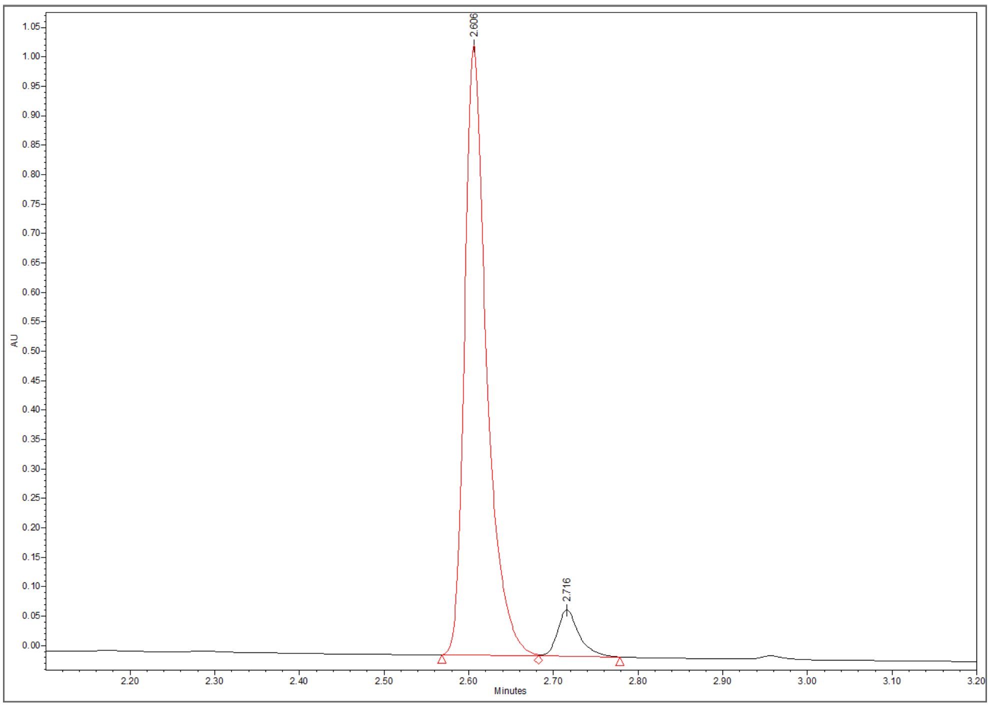

E	Name	Retention Time (min)	Purity1 Angle	Purity1 Threshold	PDA/FLR Match1 Spect. Name	PDA/FLR Match1 Angle	PDA/FLR Match1 Threshold	PDA/FLR Match1 Lib. Name	Area (µV*sec)	% Area
1		2.606							1883850	93.11
2		2.716							139422	6.89

Figure 98: SFC traces for compound **(±)-3dc** (top) and enantioenriched **(+)-3dc** (bottom).

SFC traces for compound 7

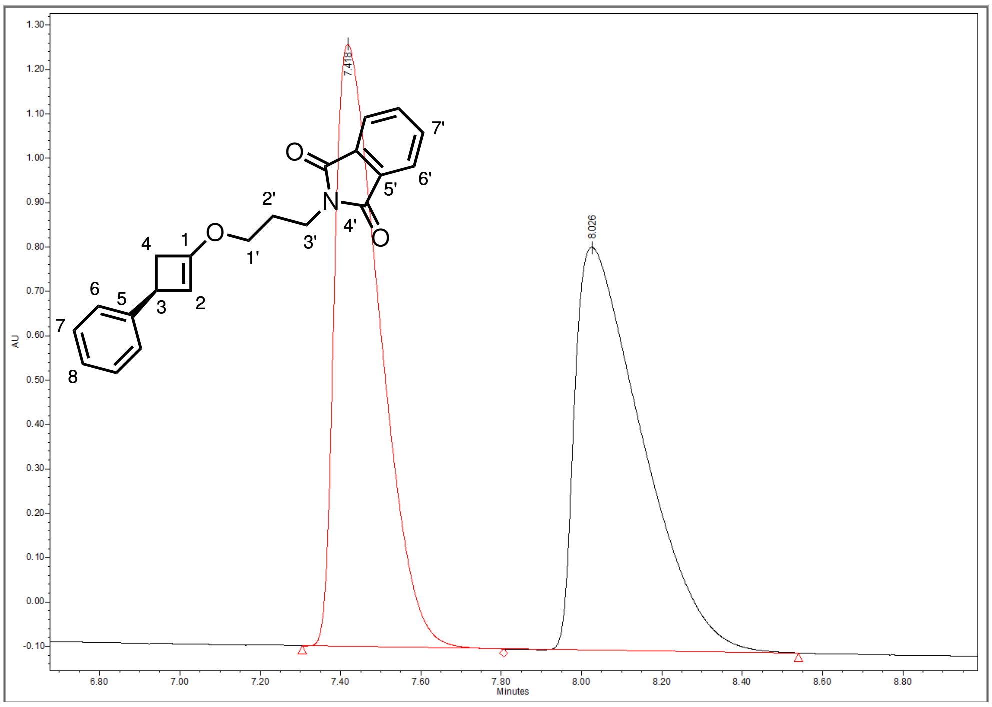

	Name	Retention Time (min)	Purity1 Angle	Purity1 Threshold	PDA/FLR Match1 Spect. Name	PDA/FLR Match1 Angle	PDA/FLR Match1 Threshold	PDA/FLR Match1 Lib. Name	Area (µV*sec)	% Area
1		7.418							10532079	49.95
2		8.026							10551115	50.05

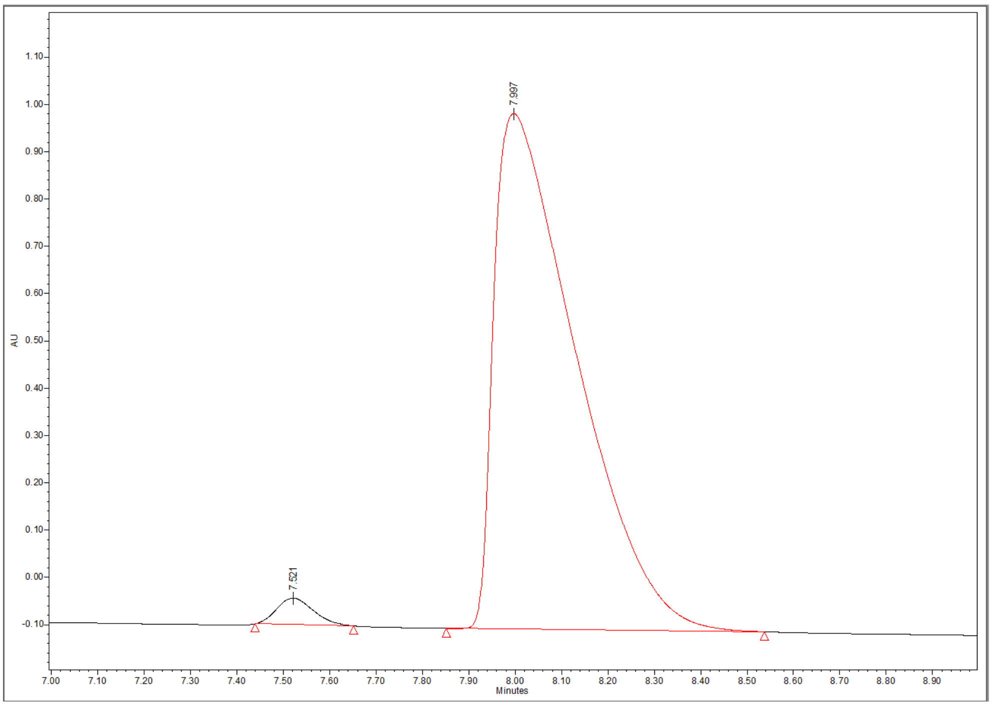

	Name	Retention Time (min)	Purity1 Angle	Purity1 Threshold	PDA/FLR Match1 Spect. Name	PDA/FLR Match1 Angle	PDA/FLR Match1 Threshold	PDA/FLR Match1 Lib. Name	Area (µV*sec)	% Area
1		7.521							308033	2.24
2		7.997							13428460	97.76

Figure 99: SFC traces for compound (±)-**7** (top) and enantioenriched (+)-**7** (bottom).

SFC traces for compound 9

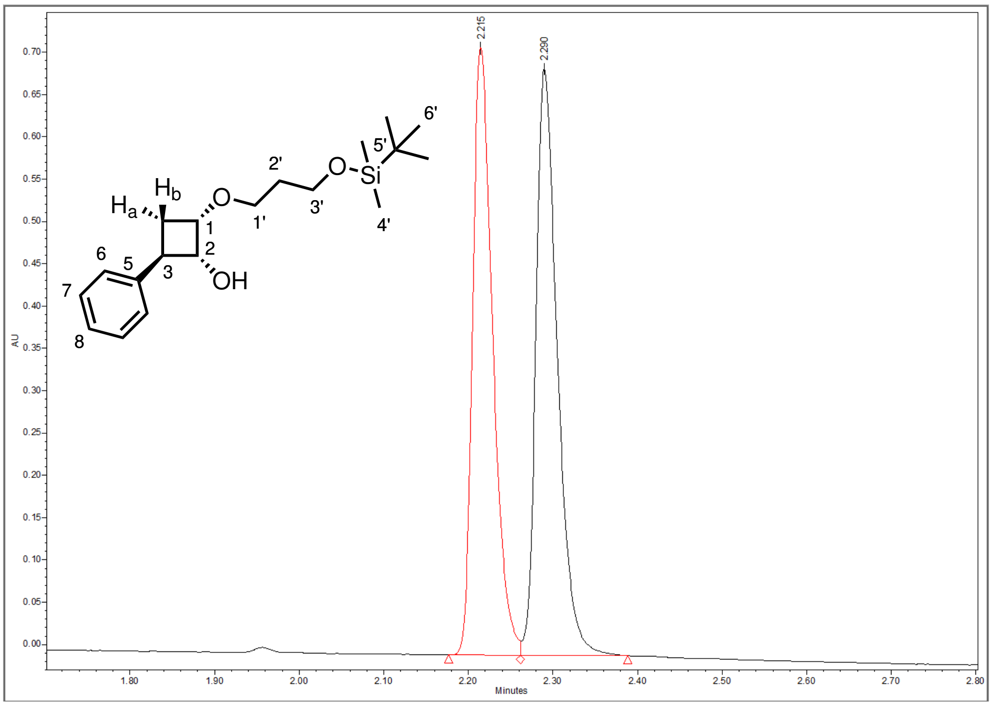

	Name	Retention Time (min)	Purity1 Angle	Purity1 Threshold	PDA/FLR Match1 Spect. Name	PDA/FLR Match1 Angle	PDA/FLR Match1 Threshold	PDA/FLR Match1 Lib. Name	Area (μV*sec)	% Area
1		2.215							1189096	49.32
2		2.290							1221784	50.68

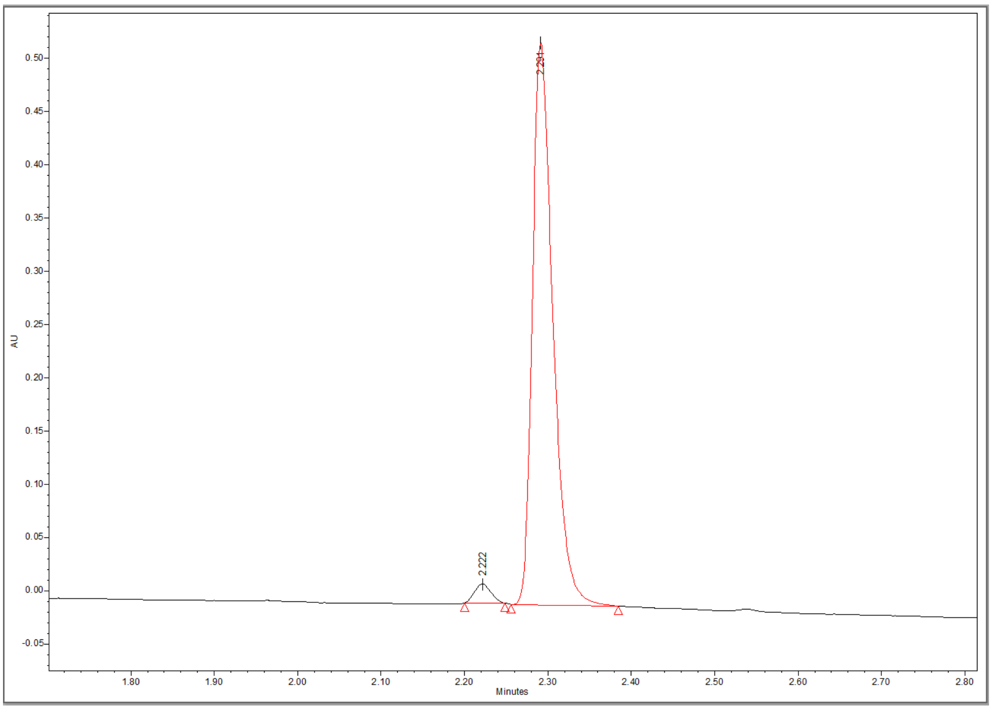

	Name	Retention Time (min)	Purity1 Angle	Purity1 Threshold	PDA/FLR Match1 Spect. Name	PDA/FLR Match1 Angle	PDA/FLR Match1 Threshold	PDA/FLR Match1 Lib. Name	Area (μV*sec)	% Area
1		2.222							24467	2.64
2		2.291							900815	97.36

Figure 100: SFC traces for compound **(±)-9** (top) and enantioenriched **(+)-9** (bottom).

SFC traces for compound 10

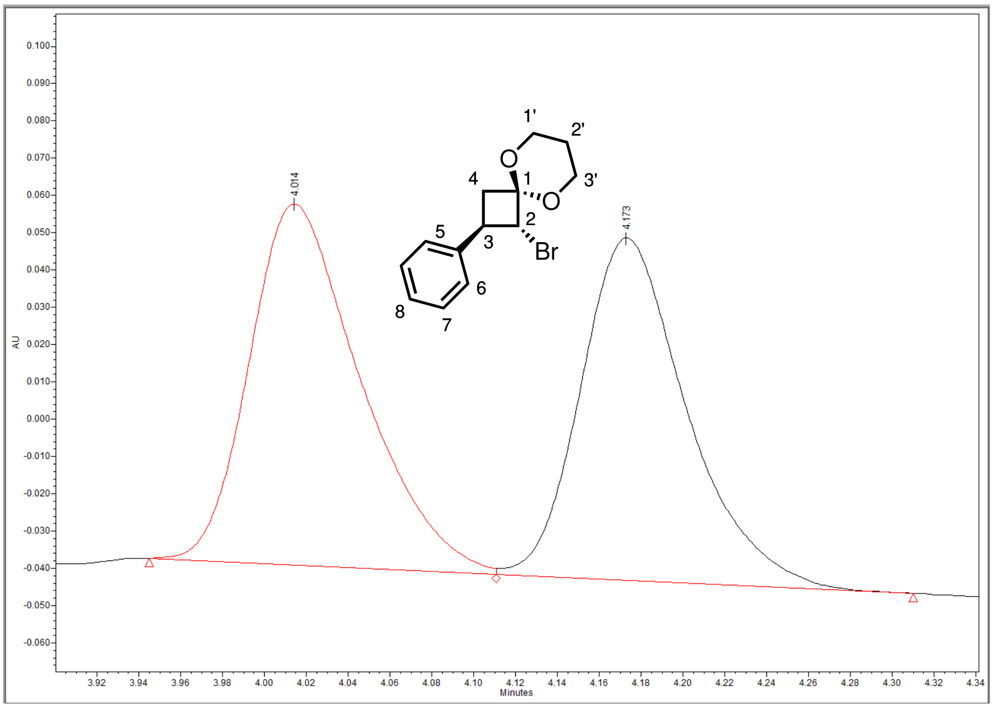

	Name	Retention Time (min)	Purity1 Angle	Purity1 Threshold	PDA/FLR Match1 Spect. Name	PDA/FLR Match1 Angle	PDA/FLR Match1 Threshold	PDA/FLR Match1 Lib. Name	Area (µV*sec)	% Area
1		4.014							356927	52.48
2		4.173							323249	47.52

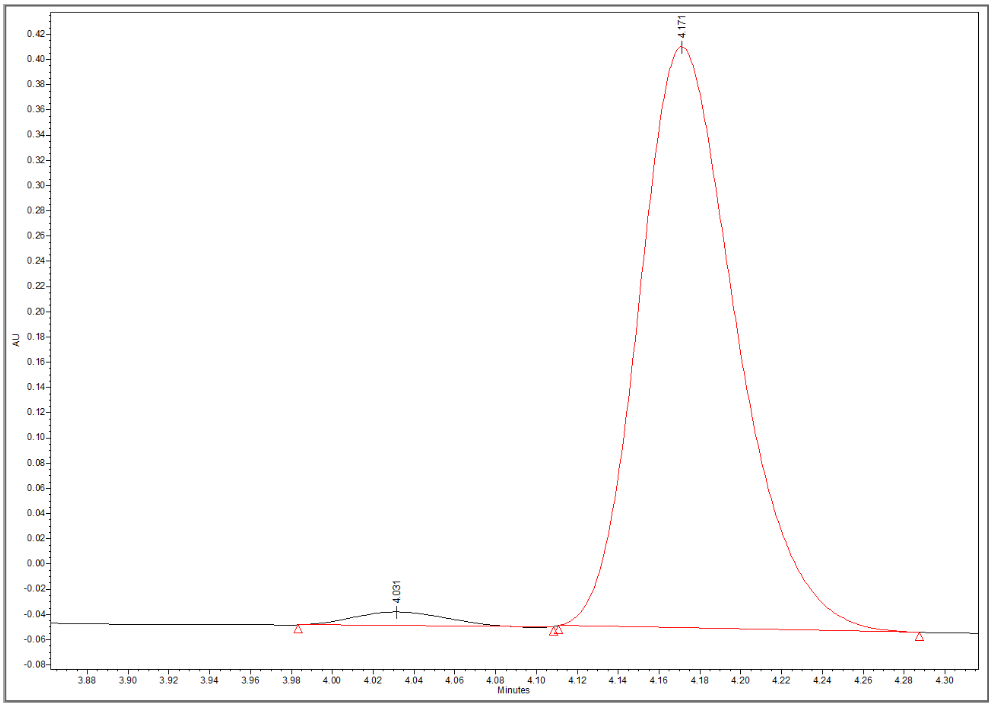

	Name	Retention Time (min)	Purity1 Angle	Purity1 Threshold	PDA/FLR Match1 Spect. Name	PDA/FLR Match1 Angle	PDA/FLR Match1 Threshold	PDA/FLR Match1 Lib. Name	Area (µV*sec)	% Area
1		4.031							33842	2.17
2		4.171							1522597	97.83

Figure 101: SFC traces for compound **(±)-10** (top) and enantioenriched **(+)-10** (bottom).
